# Substituent-Controlled Double Orthometalation and Anticancer Activity in Half-Sandwich Ir(III) Complexes Bearing C^∧^N Ligands

**DOI:** 10.1021/acsorginorgau.6c00026

**Published:** 2026-07-08

**Authors:** Mario Sánchez-Migallón, Carlos Gonzalo-Navarro, Ana María Rodríguez, Cristina Bermejo-Casadesús, Blanca R. Manzano, Anna Massaguer, Gema Durá

**Affiliations:** † 16733 Universidad de Castilla-La Mancha, Departamento de Química Inorgánica, Orgánica y Bioquímica, Facultad de Ciencias y Tecnologías Químicas, Avenida Camilo José Cela 10, 13071 Ciudad Real, Spain; ‡ Instituto Regional de Investigación Científica Aplicada (IRICA), Universidad de Castilla-La Mancha, Avenida Camilo José Cela 1, 13071 Ciudad Real, Spain; § Universidad de Castilla-La Mancha, Departamento de Química Inorgánica, Orgánica y Bioquímica, Escuela Técnica Superior de Ingenieros Industriales, Avenida Camilo José Cela 3, 13071 Ciudad Real, Spain; ∥ 16738 Universitat de Girona, Departament de Biologia, Facultat de Ciències, Maria Aurelia Capmany 40, 17003 Girona, Spain

**Keywords:** Iridium(III) anticancer complexes, half-sandwich complexes, benzo[*h*]imidazoquinoline ligands, mitochondrial dysfunction, redox homeostasis

## Abstract

Platinum-based chemotherapeutic agents remain among the most widely used anticancer drugs; however, they show two main limitations: intrinsic or acquired resistance and severe side effects. To address these limitations, the design of alternative metal-based complexes with tunable properties is of growing interest. In this work, we report the rational design and synthesis of five novel half-sandwich Ir­(III) complexes incorporating benzo­[*h*]­imidazoquinoline ligands with an aryl ring. Two strategic structural modifications were introduced into the ligand: (i) alkylation of the imidazole nitrogen (H or 4-ethylmorpholine) and (ii) variation of the aryl substituent (OMe or CN), which not only induced the formation of a dinuclear complex but also modulated key physicochemical properties, including lipophilicity, cytotoxicity, or stability. The new complexes exhibited cytotoxicity toward different cancer cell lines, with IC_50_ values in the low micromolar range, which are within the same order of magnitude as cisplatin, though operating through a distinct mechanism. Notably, the complexes induced mitochondrial dysfunction, even when morpholine-containing ligands, typically associated with lysosomal targeting, were employed. Additional studies revealed interactions of the complexes with cellular molecules such as NADH and glutathione (GSH), disrupting redox homeostasis and increasing ROS (reactive oxygen species) levels. Furthermore, the complexes exhibited moderate binding affinity for bovine serum albumin (BSA), with molecular docking analyses suggesting a specific interaction near tryptophan residue 134. These Ir­(III) complexes are promising candidates for further development as anticancer agents with distinct mechanisms of action capable of overcoming drug resistance.

## Graphical abstract

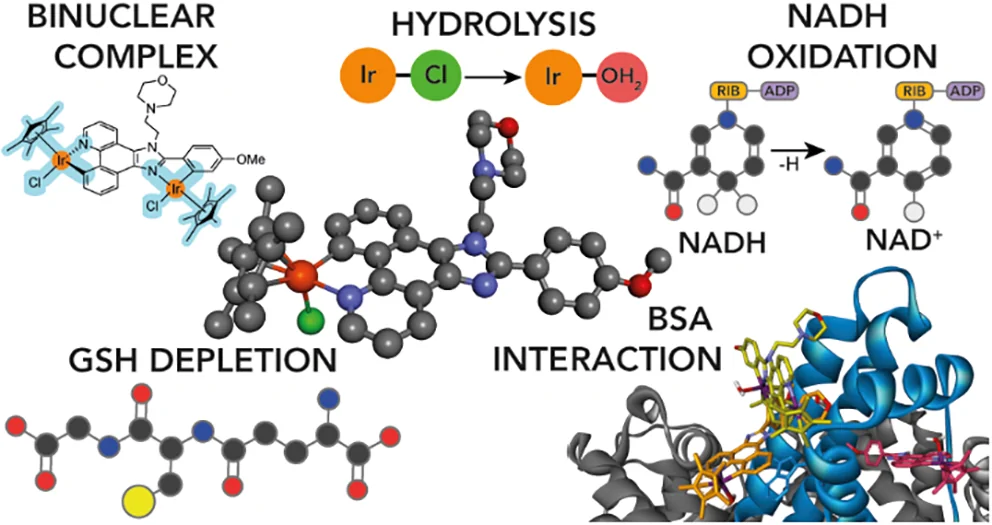

## 1 Introduction

Cancer remains one of the most prevalent and complex diseases affecting humanity, with high mortality rates and increasing global incidence,^1^ and chemotherapy continues to play a central role in its treatment. Platinum-based drugs, such as cisplatin, carboplatin, and oxaliplatin, have long dominated the chemotherapeutic landscape due to their potent cytotoxicity and broad-spectrum efficacy.^2^ However, their clinical application is limited by severe systemic toxicity, poor selectivity toward cancer tissues, and the emergence of drug resistance.^3–9^

These limitations have prompted increasing interest in nonplatinum metallodrugs.^10^ Within this broader field, Ir­(III)-based anticancer compounds are being actively investigated owing to their rich coordination chemistry, favorable photophysical properties, and tunable ligand environments,^11–15^ although none has yet reached clinical use. These systems can operate through alternative mechanisms of action, including redox activity, organelle-specific accumulation, and mitochondrial dysfunction.^16–18^

In the field of Ir­(III) complexes, several structural families have been explored, including tris­(chelate) biscyclometalated and half-sandwich families, among others. The latter, when they have the formula [Cp*Ir­(L^∧^X)­Z]^0/+^, have shown anticancer activity. Sadler and co-workers first reported that half-sandwich Ir­(III) complexes surpass cisplatin in potency,^16^ and that replacing N^∧^N chelating ligand for its C^∧^N analogue significantly enhances cytotoxicity.^19^

Ligand screening and structural modifications of the complexes have proven to be effective strategies for modulating biological activity, solubility, and subcellular localization.^20^ In this context, Paira et al. have extensively explored Cp*Ir systems bearing π-extended 1*H*-imidazo­[4,5-*f*]­[1,10]­phenanthroline ligands, including mononuclear Cp*Ir­(III) complexes designed to promote DNA intercalation, mitochondrial accumulation, or activity under hypoxic environments,^15,21^ including bimetallic systems capable of inducing oxidative stress and apoptosis.^22,23^ Zhe Liu et al. developed cationic/neutral half-sandwich Ir­(III) imidazole-phenanthroline or imidazole-phenanthrene complexes,^24^ which exhibited suitable fluorescence, and a nonenergy-dependent cellular uptake, lysosomal accumulation, and intracellular NADH oxidation, leading to increased ROS levels.

Despite these advances, half-sandwich Ir­(III) complexes bearing benzo­[*h*]­imidazoquinoline ligands remain unexplored. This is particularly noteworthy given the exceptional expected stability of the orthometalated systems, the biological relevance of the imidazole-containing scaffolds,^25,26^ and the tunability of the benzo­[*h*]­imidazoquinoline frameworks for modulating electronic structure and reactivity.

Organelle-specific localization, particularly mitochondrial targeting, is of great interest in cancer treatment due to the central role of mitochondria in cellular metabolism and apoptosis.^27,28^ Disruption of mitochondrial membrane potential (MMP) by Ir­(III) complexes can trigger apoptosis,^29–31^ while modulation of cellular redox homeostasis represents a complementary mechanism. Key cofactors such as nicotinamide adenine dinucleotide (NAD^+^) and its reduced form (NADH)^32^ and nicotinamide adenine dinucleotide phosphate (NADPH) are essential in metabolic and antioxidant pathways, and their perturbation can compromise cellular metabolism and weaken antioxidant defenses, sensitize cancer cells to oxidative stress, and enhance the efficacy of redox-active metallodrugs.^27,33,34^

Beyond the intracellular mechanisms of action, the systemic transport and delivery of therapeutic agents are critical factors in their efficacy. In this regard, interaction with serum proteins is relevant for understanding the pharmacokinetic behavior of metallodrugs.

Herein, we report the design and synthesis of five novel half-sandwich Ir­(III) complexes incorporating benzo­[*h*]­imidazoquinoline as the C^∧^N ligand, including one dinuclear species (Figure 1). Structural modifications include (i) alkylation of the imidazole nitrogen (R^1^ = H or 4-ethylmorpholine) to promote lysosomal targeting, and (ii) variation of the aryl substituent (R^2^ = OMe or CN) to modulate electronic density. All complexes exhibited moderate cytotoxicity against different cell lines and a mechanism of action distinct from cisplatin, involving reactions with NADH and GSH, intracellular ROS generation, and disruption of redox homeostasis. Binding studies and computational molecular docking analysis further revealed interactions with BSA, specifically involving the residue tryptophan 134.

**Figure 1:**
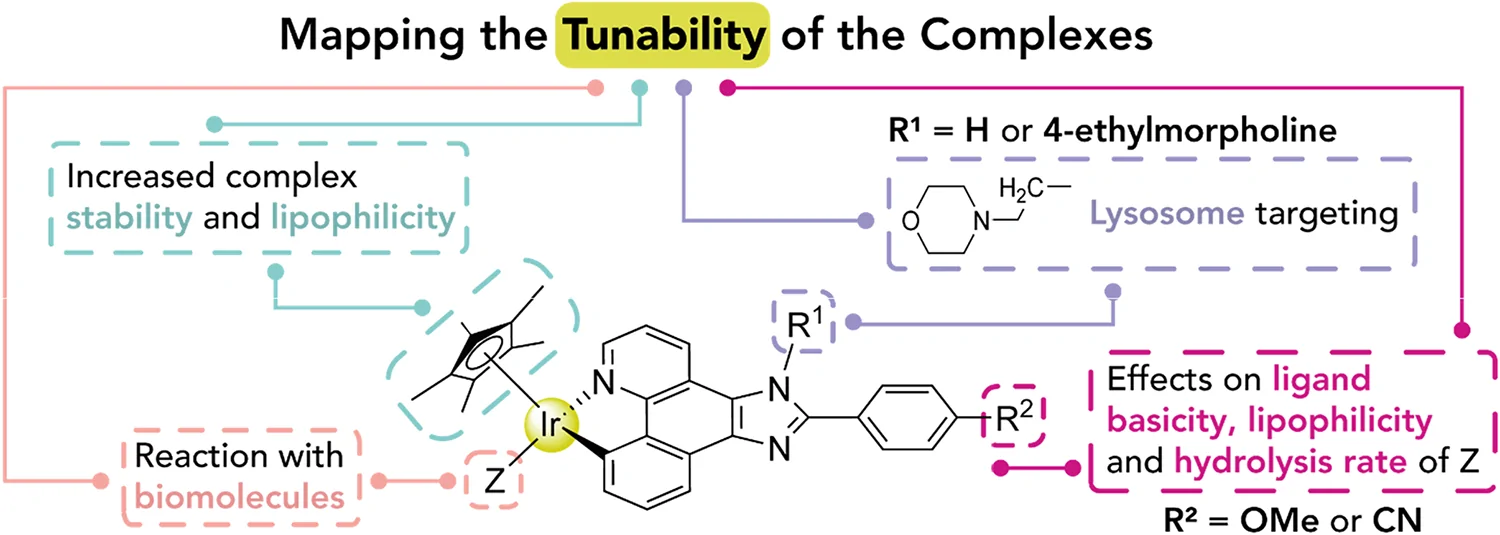
Structure of the complexes presented in this work.

## 2 Results and Discussion

### 2.1 Synthesis and Characterization

The four HC^∧^N proligands prepared contained R^1^ = H or 4-ethylmorpholine and R^2^ = OMe or CN. They are reported in this paper for the first time and were synthesized by adapting a literature procedure (Scheme 1).^35,36^ The two possible orientations of the morpholine fragment led to the formation of two isomers in case of **H-L3** (65:35 ratio) and **H-L4** (64:36 ratio) ligands.

**Scheme 1:**
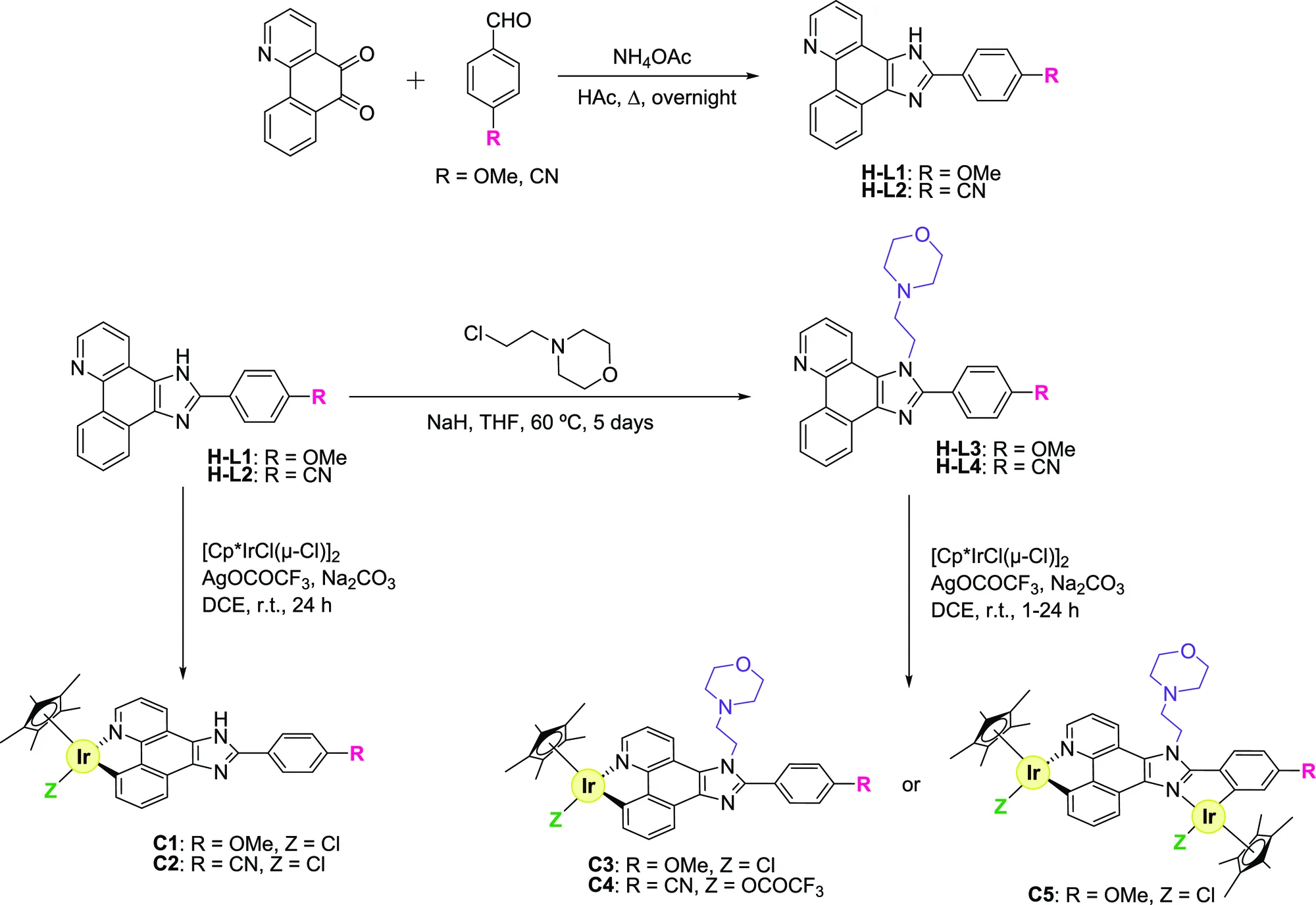
Synthesis of Proligands and Complexes Presented in This Work

Five new cyclometalated half-sandwich Ir­(III) complexes were synthesized, four of the general formula [Cp*Ir­(C^∧^N)­Z] and one bimetallic compound of formula [(Cp*IrCl)_2_ **L4**] (C^∧^N = 2-phenyl-3*H*-benzo­[*h*]­imidazo­[4,5-*f*]­quinoline derivatives, Z = chloride or trifluoroacetate) (Scheme 1, Chart S1). The synthesis of the iridium complexes was attempted by a reported procedure,^37^ by reaction of the HC^∧^N proligand with 0.5 equiv of the precursor dimer [Cp*IrCl­(μ-Cl)]_2_ in the presence of a silver salt (Ag­(OCOCF_3_)) and a base (Na_2_CO_3_) in DCE (Scheme 1). However, only complexes **C1** and **C2** could be obtained as chlorido complexes following this method. In the case of complex **C4**, a larger amount of silver salt was required, likely due to chloride abstraction and subsequent coordination of the trifluoroacetate anion.

For complex **C3**, complete ligand transformation was not achieved, and another half-sandwich Ir­(III) complex was formed as side product. The formation of the latter could be suppressed by shortening the reaction time and lowering the temperature, resulting in the isolation of **C3** as the chlorido complex. However, these conditions also reduced the overall conversion. To identify the nature of the side product, the reaction was conducted using 1.0 equiv of [Cp*IrCl­(μ-Cl)]_2_ at room temperature for a longer time, obtaining the bisorthometalated Ir­(III) complex **C5** with the ligand acting as ditopic. An analogous species was detected as an impurity during the synthesis of **C1** (Figure S1), but this compound could not be obtained in pure form using similar conditions to those of synthesis of **C5**. Efforts to generate the corresponding biscyclometalated Ir­(III) complex related to **C4** were unsuccessful, as no evidence of a second orthometalation was observed. The absence of double orthometalation with proligand **H-L4** points to a definite role of the activating (OMe) or deactivating (CN) groups of the aryl substituents on the reaction of the C–H group with the electrophilic iridium fragment. Besides, in the case of OMe, the contribution of the resonance form of Figure 2 would increase the donor ability of the imidazolyl nitrogen. The difficulty in obtaining a pure bisorthometalated product with **H-L1** reflects an influence of the 4-ethylmorpholine fragment, increasing the basicity of the imidazolyl nitrogen.

**Figure 2:**
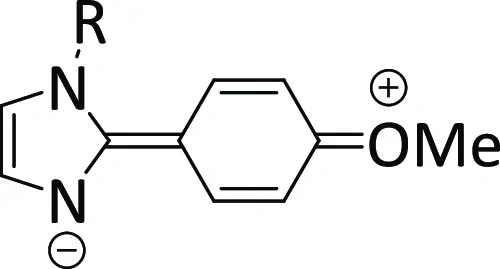
Resonance form that will increase the basicity of the imidazolyl nitrogen in **H-L3**.

The complexes were obtained in moderate yields (31–58%) as pale yellow or orange solids and were fully characterized, along with the ligands, by elemental analysis, ^1^H, ^13^C­{^1^H} and ^19^F­{^1^H} (for **C4**) NMR spectroscopy, including ^1^H–^1^H gCOSY, ^1^H–^13^C gHSQC and ^1^H–^13^C gHMBC (Figures S2–S37) and mass spectrometry (Figures S38–S46). In the case of **C5**, the ^13^C­{^1^H} spectrum could not be obtained. The ^1^H spectra were fully assigned in most cases, by means of ^1^H–^1^H COSY experiments and considering the different coupling constants expected in the pyridyl ring.^38^ It was also possible to assign some of the ^13^C­{^1^H} NMR resonances through ^1^H–^13^C gHSQC and ^1^H–^13^C gHMBC experiments. In addition, the solid structure of complexes **C1**, **C3**, **C4** and **C5** was determined by single crystal X-ray diffraction.

Complexes **C1**-**C5** exhibited good solubility in polar solvents such as dimethyl sulfoxide (DMSO), *N*,*N*-dimethylformamide (DMF) and chlorinated solvents as dichloromethane (DCM) or dichloroethane (DCE). All complexes were poorly soluble in aqueous media but dissolved upon addition of a small amount of DMSO. Thus, nontoxic amounts of DMSO were used in the biological experiments to assist dissolution.

The analysis of the ^1^H NMR spectra allowed us to corroborate the cyclometalation. For all the complexes, the absence of the signal corresponding to H^7^ and the shift of the rest of the protons confirm the cyclometalation through the benzo­[*h*]­quinoline fragment (see Figure 3). In the case of **C3** and **C4**, two isomers were detected, which likely correspond to different orientations of the morpholine fragment with respect to the benzo­[*h*]­quinoline unit. For complex **C5**, the marked modification of the typical pattern of two doublets (each integrating for two protons) of the aryl ring observed for the other complexes, together with the appearance of several peaks in two distinct Cp* regions (one similar to the other complexes and another at higher field), is consistent with the formation of a biscyclometalated species via a second orthometalation at the 2-phenylimidazole unit. In this case, in addition to the two possible orientations of the morpholine fragment, the presence of two stereogenic iridium centers gives rise to two pairs of enantiomers (RR/SS and RS/SR), corresponding to two possible diastereomers. Therefore, a total of four isomers can exist. In the Cp* region, three peaks are observed for each type of Cp*, indicating the presence of at least three of these isomers in solution.

**Figure 3:**
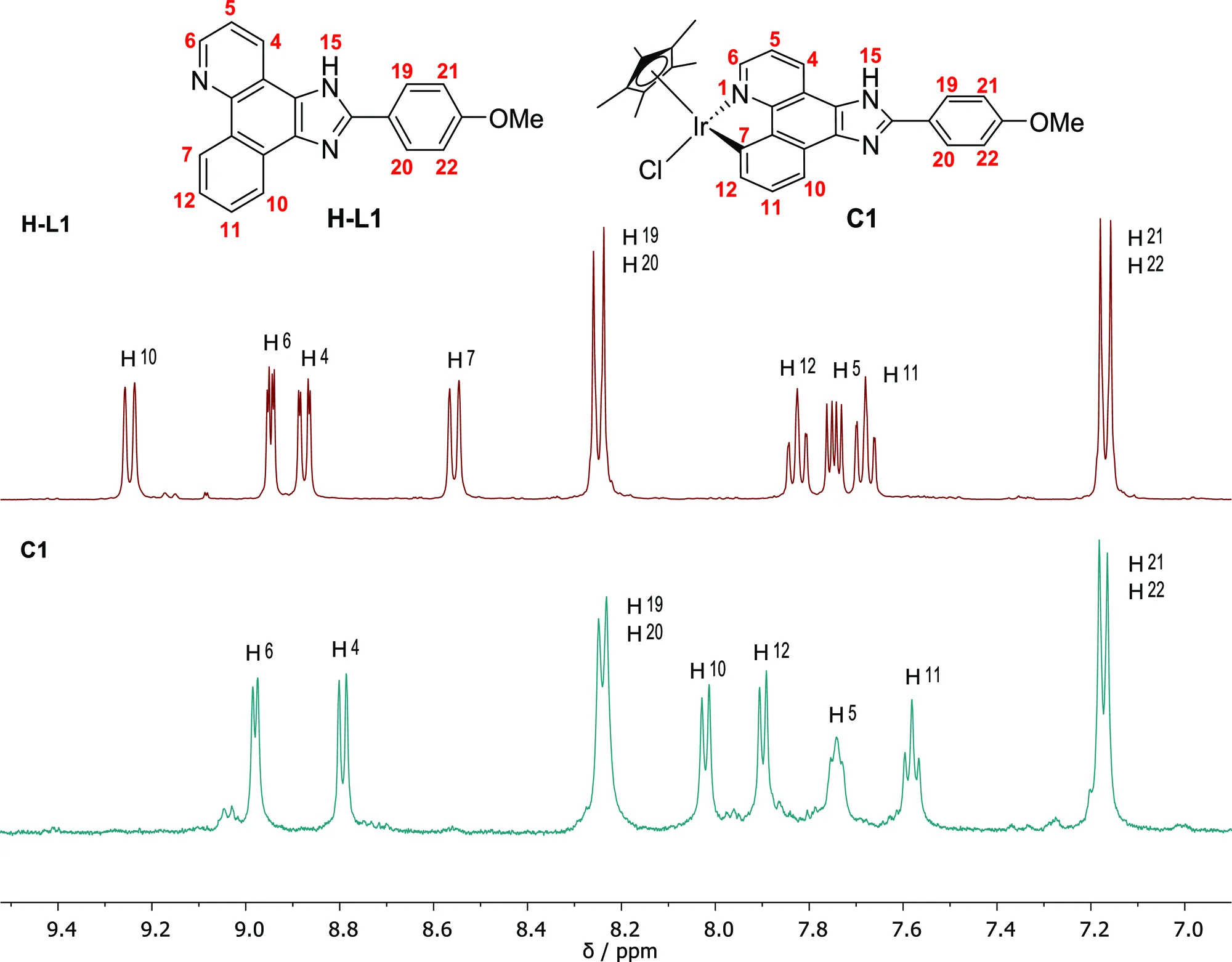
Comparison of the aromatic region of the ^1^H NMR spectra (DMSO-*d*_6_, 298 K) of proligand **H-L1** and complex **C1**.

At this point, it is worth emphasizing that, as far as we are aware, complex **C5** constitutes the first reported case of double orthometalation in complexes bearing benzo­[*h*]­imidazo­[4,5-*f*]­quinoline ligands. A complex notably different from **C5**, containing two “Cp*IrCl” fragments, was obtained with a phenanthroline derived ligand coordinated through two N^∧^N units.^22^ A mono-orthometalation involving imidazo and aryl rings has been described with an imidazole-phenanthrene derived ligand.^24^ These types of dinuclear species may be of interest across various fields. Thus, numerous studies have reported enhanced catalytic efficiency in bimetallic species when compared with mononuclear analogues.^39,40^ Furthermore, dinuclear complexes frequently display noticeable improvements in selected photophysical parameters over their mononuclear counterparts.^41–43^

### 2.2 Solid State Characterization

The molecular and crystal structures of complexes **C1**, **C3**, **C4** and **C5** were determined by X-ray diffraction. Details of the crystallization procedures and space groups are provided in the Supporting information. Selected bond distances and angles are gathered in Table 1, the crystallographic data are provided in Table S1, and the corresponding ORTEP diagrams are shown in Figure 4.

**Figure 4:**
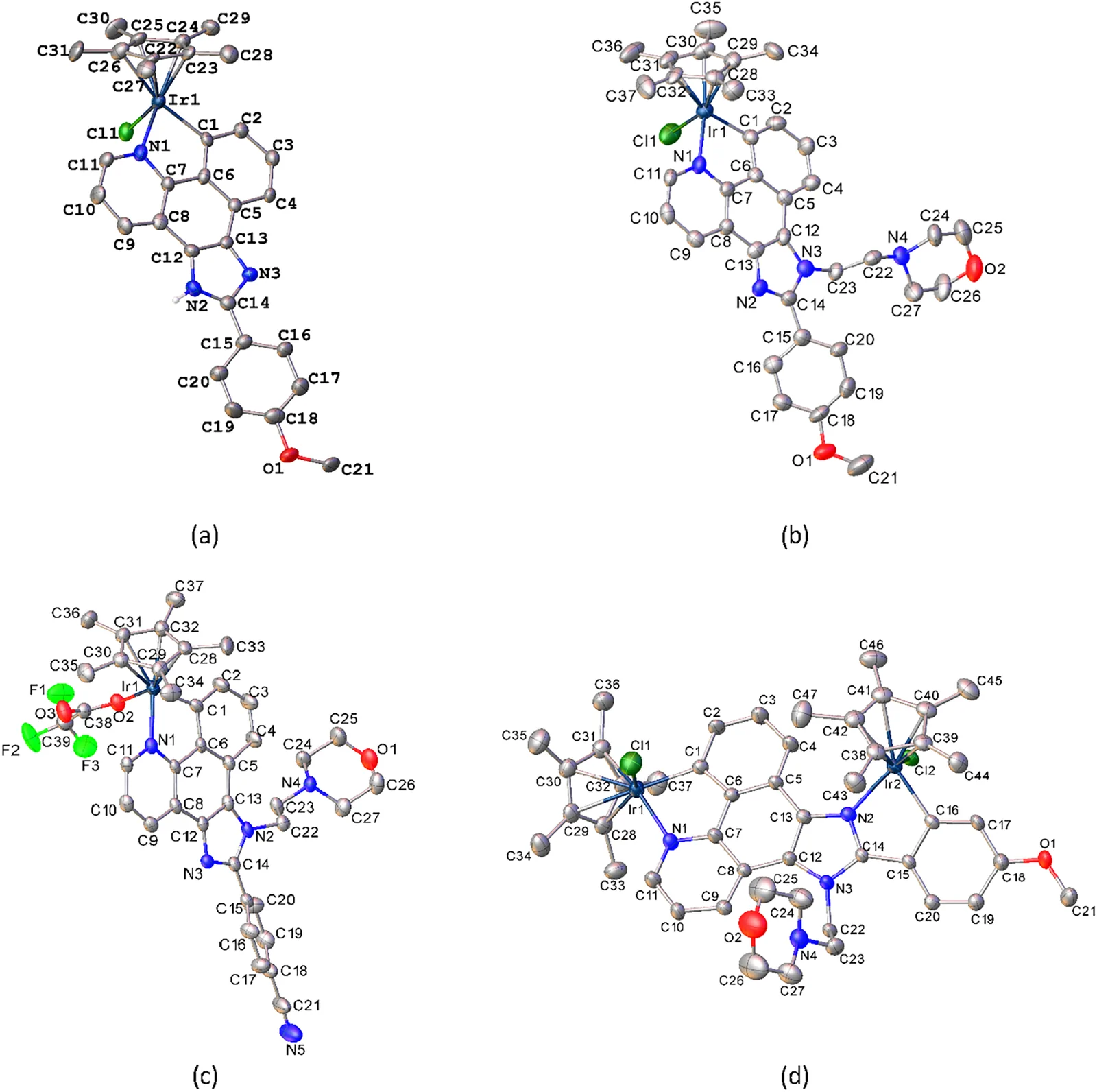
ORTEP diagrams of complexes S_Ir_ **C1** (a), S_Ir_ **C3** (b), S_Ir_ **C4** (c) and R_Ir_,S_Ir_ **C5** (d). Ellipsoids are at the 30% probability level. Hydrogen atoms have been omitted for clarity.

**Table 1:** Selected Bond Distances (Å) and Angles (°) for Complexes **C1**, **C3**, **C4** and **C5**

Comp.	C^∧^N				L		Ir-Cp* (centr.)	Bite angle (C–Ir–N)
**C1**	Ir(1)–C(1)	2.08(1)	Ir(1)–N(1)	2.059(9)	Ir(1)–Cl(1)	2.412(3)	1.812	78.5(3)
**C3**	Ir(1)–C(1)	2.020(1)	Ir(1)–N(1)	2.071(7)	Ir(1)–Cl(1)	2.403(3)	1.827	78.5(3)
**C4**	Ir(1)–C(1)	2.036(9)	Ir(1)–N(1)	2.072(6)	Ir(1)–O(2)	2.143(5)	1.816	78.3(3)
**C5 Ir(1)** ^a^	Ir(1)–C(1)	2.060(2)	Ir(1)–N(1)	2.050(1)	Ir(1)–Cl(1)	2.380(5)	1.823	77.9(6)
**C5 Ir(2)** ^a^	Ir(2)–C(16)	2.050(2)	Ir(2)–N(2)	2.110(1)	Ir(2)–Cl(2)	2.408(4)	1.809	77.8(5)

a Ir­(1) corresponds to the frontal cyclometalation and Ir(2) corresponds to the lateral cyclometalation.

The unit cells of the complexes contain both enantiomers, R_Ir_ and S_Ir_ (**C1**, **C3** and **C4)** or S_Ir_,R_Ir_ and R_Ir_,S_Ir_ (**C5**) arising from the stereogenic nature of the metal center. All the complexes presented a pseudo-octahedral half-sandwich geometry in which the Cp* rings adopt a η^5^-coordination mode and occupy three coordination positions, the C^∧^N ligands present a bidentate-chelate coordination mode (κ^2^-*C,N*) and the Z ligands are coordinated in a monodentate mode, completing the coordination positions. In complexes **C3** and **C4**, the morpholine unit is positioned on the same side as the benzene ring of the C^∧^N ligand, whereas in **C5**, it lies on the same side as the pyridine ring. In addition, in **C5**, the two Cp* rings are orientated opposite to each other with respect to the plane defined by the **L3** ligand.

In the solid structure of the complexes, the C^∧^N ligands retain planarity across the 3*H*-benzo­[*h*]­imidazo­[4,5-*f*]­quinoline moiety, which is illustrated by red planes in Figures S47–S50. In **C1**, the methoxyphenyl ring is slightly deviated (22.4°) regarding to this plane, while larger torsion angles are observed in **C3** (38.3°) and **C4** (39.9°), which can be attributed to steric hindrance involving the 4-ethylmorpholine group. In complex **C5**, the torsion is smaller (22.0°), as a consequence of the lateral orthometalation of the **L3** ligand, which occurs between the 3*H*-benzo­[*h*]­imidazo­[4,5-*f*]­quinoline and the methoxyphenyl moieties.

Concerning noncovalent interactions, π–π interactions involving the C^∧^N ligands were observed only in complex **C1**, leading to the formation of box-like dimers further stabilized by C–H···π interactions (Figure S51). Similar box-like dimeric arrangements stabilized by π–π interactions have been previously reported for other half-sandwich Ir­(III) complexes.^37,44^ The reason for the lack of this type of interactions in the solid structures of complexes **C3**-**C5** must lie in the loss of coplanarity of the 4-substituted phenyl ring, the presence of the 4-ethylmorpholine group and, in the case of **C5**, the presence of the second “Cp*Ir” fragment. For these complexes, only weak C–H···π interactions and hydrogen bonds were found (Figures S52–S54).

### 2.3 Stability in Solution

In many anticancer transition metal complexes, hydrolysis of the M–Cl bond serves as a critical activation step. The resulting aqua complex (M–OH_2_) exhibits enhanced reactivity compared to its chlorido analogue, enabling interactions with biological targets such as DNA and proteins that underlie its therapeutic activity.^16,45^ The solvolysis process of the new five Ir­(III) complexes was studied by ^1^H and ^19^F NMR spectroscopy in DMSO-*d*_6_:D_2_O (8:2) (Figures S55–S60 and Table S2) and in DMSO-*d*_6_ (Figures S61–S66 and Table S3) at room temperature.

The four chlorido complexes (**C1**, **C2**, **C3** and **C5**) underwent solvolysis. The disappearance of the chloride derivatives was relatively fast for **C1**, **C2** and **C3** and moderate for **C5**. The reaction profiles were fitted to a pseudo-first order kinetic model (Figure 5), only as a descriptive tool to compare the apparent solvolysis behavior under identical experimental conditions. The resulting parameters are summarized in Table 2. In DMSO-*d*_6_:D_2_O (8:2), the *t*_1/2_ values estimated from the fitted profiles were in the range of 2–4 h for **C1**, **C2** and **C3** and 8 h for **C5**. The NMR monitoring also indicated that solvolysis was favored by increasing the water content of the solvent mixture. In DMSO-*d*_6_, containing only trace amounts of water, the reaction proceeded much more slowly than in DMSO-*d*_6_:D_2_O (8:2) (see Figure S67), as reflected by the corresponding solvolysis profiles and the apparent half-life times listed in Table 2. On the contrary, complex **C4**, with a trifluoroacetate ligand, exhibited robust stability under these conditions. No evidence for the formation of a solvent adduct was observed, even when the solution was heated at 90 °C for 1 h in DMSO-*d*_6_:D_2_O (8:2) (Figures S68–S69).

**Figure 5:**
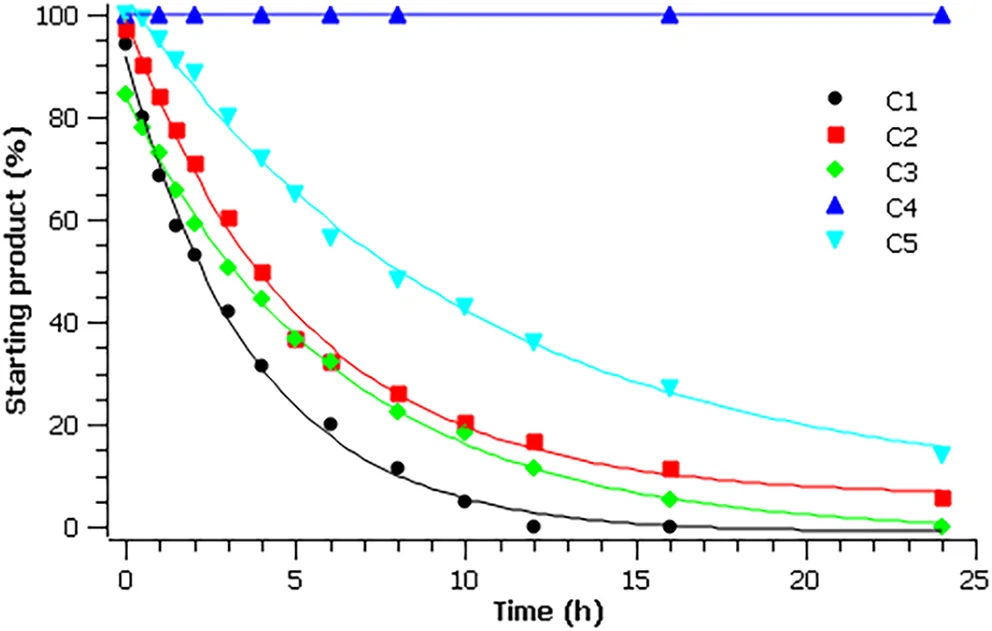
Solvolysis of the Ir­(III) complexes in DMSO-*d*_6_:D_2_O (8:2) by ^1^H NMR.

**Table 2:** Apparent Solvolysis Parameters Estimated from ^1^H NMR Spectra for Complexes **C1**-**C5** in DMSO-*d*_6_ and DMSO-*d*_6_:D_2_O (8:2)

	DMSO-*d*_*6*_:D_2_O (8:2)		DMSO-*d*_*6*_	
Complex	*k* (h^–1^)	*t*1/2 (h)	*k* (h^–1^)	*t*_1/2_ (h)
C1	0.279 ± 0.009	2.48 ± 0.08	0.085 ± 0.006	8.1 ± 0.6
C2	0.178 ± 0.005	3.9 ± 0.1	0.065 ± 0.004	10.6 ± 0.6
C3	0.168 ± 0.003	4.12 ± 0.07	0.025 ± 0.004	13 ± 4
C4	0	-	0	-
C5	0.088 ± 0.005	7.8 ± 0.4	0.0072 ± 0.0002	96 ± 3

The hydrolysis of complex **C1** was evaluated by UV–vis spectroscopy in aqueous solution (4% DMSO). However, the equilibrium was reached before the acquisition of the first spectrum and no changes were observed after 24 h (Figure S70), indicating that the hydrolysis process leading to the aqua adduct **C1w** occurs almost immediately when the water fraction is close to 100%.

The reversibility of the solvolysis process was evaluated by ^1^H NMR upon addition of 150 equiv of *n*-Bu_4_NCl to a solution of **C3w** in DMSO-*d*_6_:D_2_O (8:2) after 48 h, mimicking the high concentration of chlorides typically present in biological environment.^46^ No evidence of regeneration of the chlorido complex was observed at room temperature (Figure S71), indicating that solvolysis is irreversible under these conditions.

Additional NMR experiments were performed in the presence of increasing chloride concentrations (23 mM, representative of cytoplasmic conditions; 104 mM, representative of blood plasma chloride levels; and up to 100 equiv) for complex **C4** in DMSO-*d*_6_:D_2_O (8:2) in order to evaluate the stability of the trifluoroacetate ligand toward chloride attack. However, no significant spectral changes were observed in any case, even after thermal treatment (Figure S72). Therefore, the trifluoroacetate ligand is expected to remain coordinated in aqueous media.

Taken together, these results suggest that complexes **C1–C3** and **C5** are able to generate the corresponding cationic aqua species under aqueous conditions, whereas **C4** remains comparatively stable. The aqua species (**C1w**, **C2w**, **C3w**, and **C5w**), are likely to contribute to the speciation profile relevant to biological media. The generation of these aqua adducts are expected to enhance both solubility and cellular uptake. Consequently, any observed cytotoxic activity is attributed to the aqua complexes rather than the chlorido precursors of these complexes.

### 2.4 p*K*_a_ Determination

The reactivity of the aqua adducts is strongly influenced by the p*K*_a_ of the Ir–OH_2_ bond. Previous studies have demonstrated that the biological activity of the classical half-sandwich Ir­(III) complexes comes from the aqua adduct Ir–OH_2_ rather than the chlorido complexes.^45,46^ Furthermore, the presence of morpholine units in the C^∧^N ligands can modulate their behavior in solution, as the N atom is capable of undergoing protonation/deprotonation equilibria, which may, in turn, influence their biological properties.^47^ The p*K*_a_ of complexes **C1w**, **C2w** and **C3w** was determined by UV–vis spectroscopy. Changes in absorbance at different wavelengths were monitored as a function of pH (Figures S73–75).

The titration curves were analyzed using the Henderson–Hasselbalch equation to determine the p*K*_a_ values. Complexes **C1w** (p*K*_a1_ = 7.04 and p*K*_a2_ = 9.55) and **C2w** (p*K*_a1_ = 7.00 and p*K*_a2_ = 8.94) exhibited two equilibria in the same pH ranges. In contrast, **C3w** exhibited two peaks at p*K*_a1_ = 5.55 and p*K*_a2_ = 7.14. The common feature observed for all complexes is a deprotonation process occurring around p*K*_a_ ≈ 7, which can be attributed to the deprotonation of the coordinated water molecule to the Ir­(III) atom. This assignment is consistent with previous reports on half-sandwich Ir­(III) aqua complexes exhibiting similar proton dissociation behavior.^45,46^

In addition to the deprotonation of the Ir–OH_2_ moiety, other p*K*_a_ values, corresponding to different protonation–deprotonation equilibria occurring at different fragments of the molecules, were obtained. In **C1w** and **C2w**, the p*K*_a_ ≈ 9 value is assigned to the deprotonation of the NH group on the imidazole ring of the C^∧^N ligand (Scheme 2). This value is consistent with those reported for other N^∧^N-type ligands bearing an analogous NH group.^26,48^

**Scheme 2:**
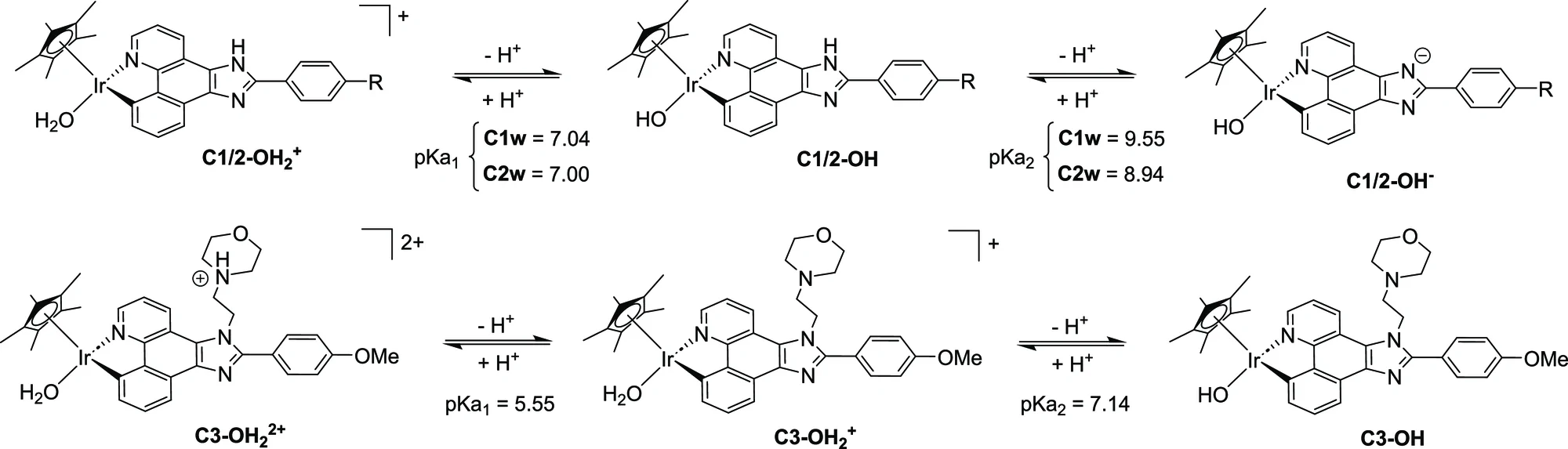
Protonation/Deprotonation Processes in Complexes C1w, C2w and C3w

Conversely, the p*K*_a_ value of 5.55 observed for **C3w** is attributed to the protonation of the nitrogen atom in the morpholine moiety. This finding is consistent with those reported for other compounds containing a morpholine unit.^25,47^ Therefore, the nitrogen atom of the morpholine group in **C3w** would be expected to remain predominantly unprotonated under physiological conditions (pH = 7.4), but could become protonated within the acidic environment of lysosomes.

### 2.5 Photophysical Properties

The UV–vis spectra of complexes **C1w**-**C3w**, **C4** and **C5w** and the proligands **H-L1**-**H-L4** were recorded in aqueous solution (4% DMSO, pH 7.0) at 25 °C (Figures 6 and S76). Table 3 provides a summary of all collected data. The UV–vis absorption spectra of the complexes exhibited very intense bands in the UV region and less intense bands in the vis region up to 450 nm. The strong bands observed at λ ≤ 300 nm can be attributed to the spin-allowed ligand-centered (LC) π–π* transitions combined with metal-to-ligand charge-transfer (MLCT) transitions, while the bands of lower energy may be due to MLCT transitions, as it has been reported for other similar half-sandwich Ir­(III) complexes with C^∧^N ligands.^37,44^ In the case of **C5w**, the absorption spectrum displays additional bands, which are attributed to an increased number of MLCT transitions arising from the second Cp*Ir fragment, in comparison with **C3w**.

**Figure 6:**
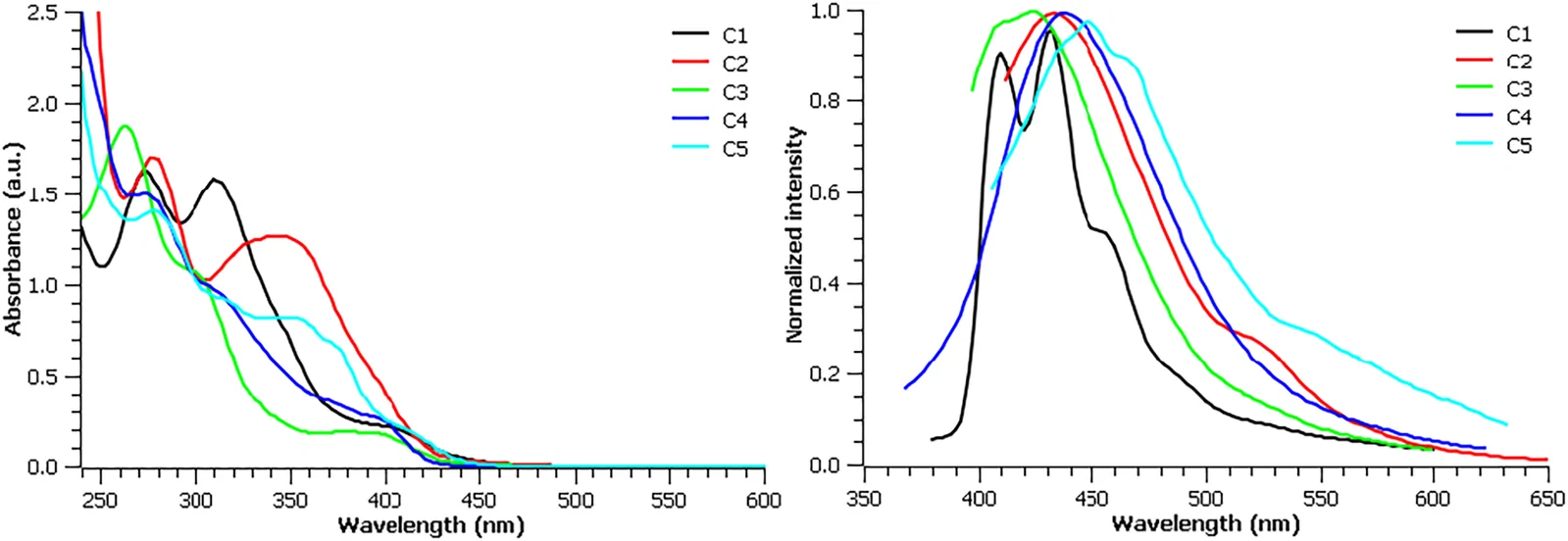
UV–vis absorption (left) and emission spectra (right) of complexes **C1w**-**C3w**, **C4** and **C5w** at 5.0 × 10^–5^ M in water (4% DMSO, pH 7.0).

**Table 3:** Photophysical Properties of Complexes **C1w-C3w**, **C4** and **C5w** in Aqueous Solution (4% DMSO, pH 7.0)

Compound	λ/nm (ε/M^–1^·cm^–1^)	λ_em_ (λ_exc_)/nm
**H-L1**	260 (19100), 274 (19600), 314 (14400)	448 (326)
**H-L2**	246 (14800), 274 (10600), 350 (9200)	442 (350)
**H-L3**	252 (24900), 286 (16800), 362 (1100)	440 (290)
**H-L4**	242 (38200), 276 (19900), 360 (6100)	442 (326)
**C1w**	276 (32600), 312 (32400), 408 (4000)	432 (355)
**C2w**	278 (34600), 346 (25300)	432 (352)
**C3w**	264 (38100), 298 (21600), 384 (3800)	425 (345)
**C4**	274 (30100), 312 (19400), 398 (5400)	438 (344)
**C5w**	280 (28400), 354 (16500), 372 (13600), 412 (4000)	450 (330)

The photoluminescence emission spectra of the compounds (Figure 6) and the proligands (Figure S76) in aqueous solution (4% DMSO, pH 7.0) were recorded at 5.0 × 10^–5^ M at 25 °C. Overall, the emission intensity of the complexes was markedly weak, and vibronically structured bands could be observed in some cases. The emission band is centered at around the 425–450 nm range, closely resembling the emission profiles of the corresponding proligands. This similarity suggests that the emission originates predominantly from the C^∧^N ligand. Additionally, complex **C5w** exhibited the longest wavelength emission at 450 nm. Nevertheless, since the emission is notably weak across all complexes, it was not possible to determine their emission quantum yields.

Given the presence of the morpholine moiety, the effect of the pH on the emission properties of complex **C3w** was monitored. Previous studies have reported enhanced emission intensity under acidic conditions for morpholine derivatives.^25,47^ Accordingly, the emission behavior of **C3w** was monitored across a pH range of 2 to 11. The results revealed that the emission intensity remained largely invariant throughout the range, indicating minimal sensitivity to proton concentration under the employed conditions. A slight spectral variation was observed at pH values above 6, which could be attributed to the deprotonation of the fragment Ir–OH_2_ (Figure S77).

### 2.6 Lipophilicity

The lipophilicity of complexes **C1w**-**C3w**, **C4** and **C5w** was assessed by determining their log *P* values, using the “shake-flask” method, to examine a possible correlation with their biological activity. The results are shown in Table 4. Focusing on complexes bearing R^2^ = OMe, a notable decrease in lipophilicity was observed upon the introduction of the morpholine moiety into the C^∧^N ligand (as shown by the log *P* values of **C1w** and **C3w**). This reduction could be attributed to hydrogen bonding interactions between the heteroatoms in the rings and water molecules. Complex **C5w**, which features two Cp*IrCl fragments capable of undergoing hydrolysis to generate a dicationic species, exhibited the highest hydrophilicity in the series. Complexes with R^2^ = CN (**C2w** and **C4**) were more hydrophilic and interestingly, the presence of the morpholine group had a negligible effect on lipophilicity, suggesting that the electron-withdrawing nature of the CN substituent may counterbalance the hydrophilic contribution of the morpholine unit.

**Table 4:** Lipophilicity (Log *P* Values) of Complexes **C1w**-**C3w**, **C4** and **C5w** Determined by the “Shake-Flask” Method

	**C1w**	**C2w**	**C3w**	**C4**	**C5w**
log *P*	0.38 ± 0.12	–0.339 ± 0.079	0.053 ± 0.010	–0.350 ± 0.040	–0.97 ± 0.19

### 2.7 Oxidation of NADH

Based on the central role of NAD^+^ and NADPH in cellular metabolism and redox homeostasis, the catalytic oxidation of NADH by complexes **C1w**-**C3w**, **C4** and **C5w** was studied over 24 h by UV–vis spectroscopy. The evolution of the reaction was monitored by the decrease of the characteristic absorption band of NADH at 340 nm (Figure S78). Overall, all complexes demonstrated moderate catalytic activity toward the oxidation of NADH. Among them, complexes **C2w** and **C4** exhibited the highest catalytic activities, as evidenced by their turnover numbers (TONs) and turnover frequencies (TOFs), presented in Figure 7 and Table S4. These values are comparable to those reported for some cyclometalated half-sandwich Ir­(III) complexes under similar experimental conditions (Table S5), although they remain lower than those of the most active systems described in the literature.^37,49,50^

**Figure 7:**
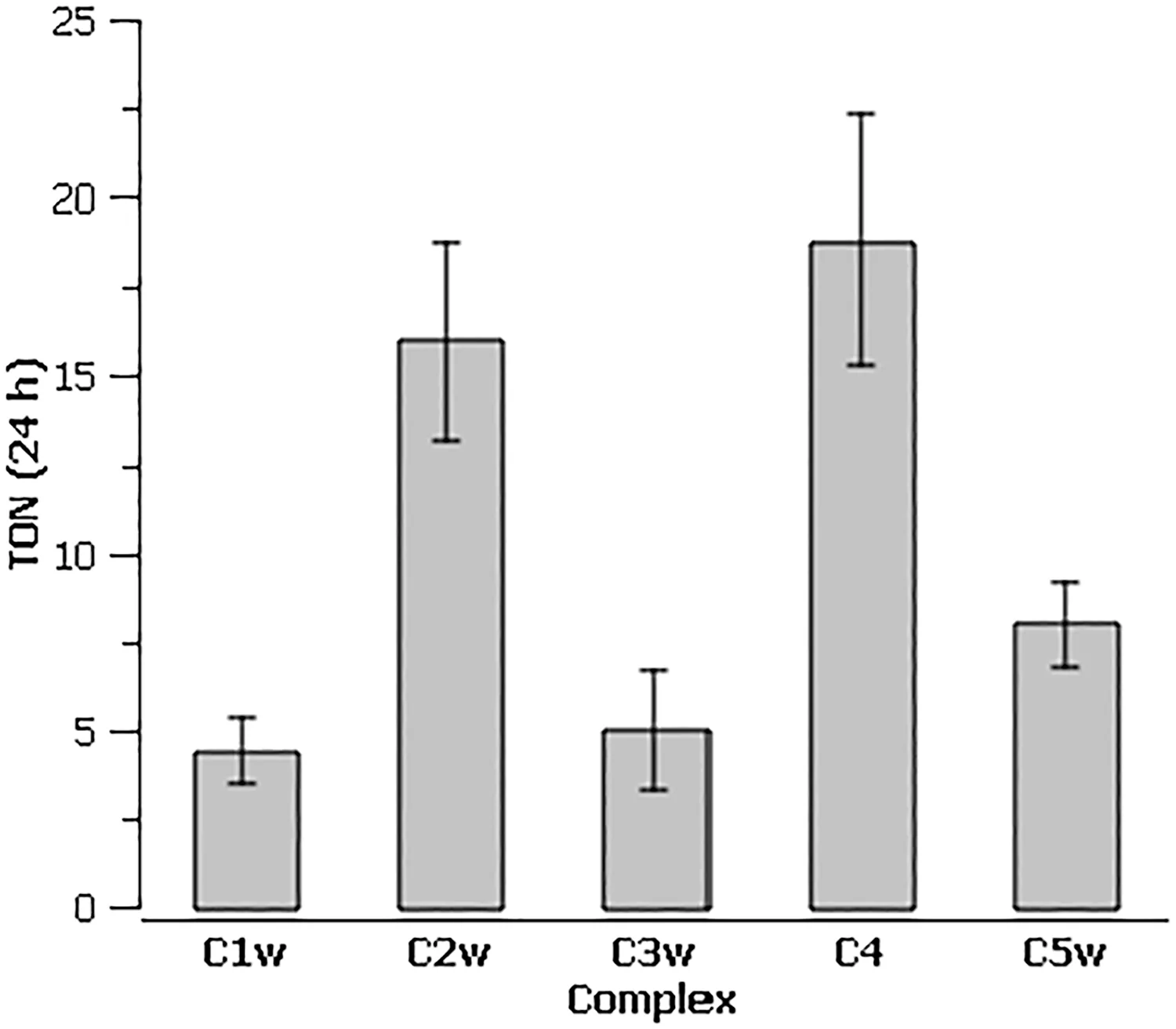
TON values from the catalytic oxidation of NADH (100 μM) with complexes **C1w**-**C3w**, **C4** and **C5w** (2.5 μM). The experiments were performed by triplicates.

The catalytic mechanism for NADH oxidation by half-sandwich Ir­(III) complexes has been reported to proceed via the formation of transient iridium hydride (Ir–H) intermediates, which can transfer a hydride equivalent to molecular oxygen, thereby generating H_2_O_2_.^49,51^ To examine whether a similar pathway operates in the presented Ir­(III) systems, the catalytic oxidation of NADH was investigated by ^1^H NMR spectroscopy. A solution containing complex **C2w** (2 mM) and NADH (5 equiv) in DMSO-*d*_6_:H_2_O (1:1) was monitored over time. As shown in Figure S79, the characteristic resonances of NADH at 8.43 and 8.11 ppm progressively decreased, accompanied by the concomitant appearance of signals attributable to NAD^+^ at 8.37 and 8.09 ppm. However, in the present study, neither Ir–H species nor H_2_O_2_ could be detected under the experimental conditions.

### 2.8 GSH Depletion

Considering the pivotal role of NADPH and reduced GSH in the regulation of intracellular oxidative stress,^27,33^ and bearing in mind the demonstrated ability of the presented Ir­(III) complexes to oxidize NADH (employed here as a structural and functional analogue of NADPH) their ability to deplete GSH levels was subsequently investigated. The experiments were performed using Ellman’s reagent (5,5′-dithiobis­(2-nitrobenzoic acid), DTNB), a classical probe used for quantifying free thiol groups. In this assay, the colorless DTNB reacts with thiols to produce 2-nitro-5-thiobenzoate (TNB), which exhibits a characteristic yellow color that can be easily monitored by UV–vis spectroscopy.

First, a calibration curve was established using increasing concentrations of GSH (Figures S80–S81). Subsequently, solutions of the Ir­(III) complexes (10 μM) and GSH (50 μM) in Tris-HCl buffer (pH 7.2) were incubated for 4 h at room temperature, and the UV–vis spectra were recorded after the addition of DTNB. The residual GSH concentration was quantified from the calibration curve, showing that approximately 20 μM of GSH was consumed in the presence of each complex (Table S6). These results indicate that all five Ir­(III) complexes can deplete GSH in physiologically relevant conditions.

Taken together with the observed NADH oxidation, these findings suggest that these Ir­(III) complexes are capable of disrupting the biochemical regeneration pathways of two key antioxidant species, NADH/NADPH and GSH, both of which play essential roles in neutralizing ROS within cancer cells. This dual interference is anticipated to compromise the cellular antioxidant defense system of cancer cells, thereby enhancing their susceptibility to oxidative stress.^34^

### 2.9 Interaction with BSA

In the rational design of effective anticancer agents, the investigation of their interactions with serum proteins is essential for evaluating their potential therapeutic application. In this context, human serum albumin (HSA) is often considered since it is the main protein in the blood of mammals and is involved in the transport and distribution of a wide range of endogenous and exogenous molecules.^52^ To perform this study, bovine serum albumin (BSA) was selected as a model of albumin protein, as it enables the assessment of this interaction by spectroscopic methods.

The interaction between the half-sandwich Ir­(III) complexes bearing the R^2^ = OMe substituent (**C1w**, **C3w** and **C5w**) and BSA was examined by fluorescence emission spectroscopy. The intrinsic fluorescence of BSA, centered at 340 nm (λ_exc_ = 285 nm), was recorded upon incremental addition of the complexes (0–60 μM). A progressive, concentration-dependent quenching of the BSA emission was observed, consistent with interactions involving the protein’s emissive tryptophan residues.^53,54^ As shown in Figure S82, the stepwise decrease in fluorescence intensity reflects a moderate binding affinity of the complexes for BSA.

To gain further insight into the quenching mechanism, the data were evaluated using the Stern–Volmer eq (eq 4, *Experimental Section* and Figure S83). At high concentrations of the complexes, the Stern–Volmer plots deviated from linearity, indicating that the model cannot fully describe the system. Such deviation excludes a purely dynamic quenching process and points instead to a more complex mechanism. As a result, the Lineweaver–Burk eq (eq 5, *Experimental Section*), commonly applied to static quenching, was therefore used. Analysis with this model yielded a linear correlation (Figure S84), confirming the predominance of such a mechanism. The static quenching constants (*K*_LB_) derived from this approach are summarized in Table 5 and lie in the range of 8.68 × 10^4^ to 1.11 × 10^5^.

**Table 5:** Binding Parameters for the interaction of the complexes **C1w**, **C3w** and **C5w** with BSA^a^

Compound	*K*_LB_ (M^–1^)	*K*_b_ (M^–1^)	*n*
**C1w**	1.11 × 10^5^	1.97 × 10^5^	1.032
**C3w**	4.46 × 10^4^	2.19 × 10^5^	1.119
**C5w**	8.68 × 10^4^	2.74 × 10^6^	1.265

a *K*_LB_ = static quenching constant (M^–1^), *K*_b_ = binding constant, *n* = number of binding sites.

Finally, the Scatchard eq (eq 6, *Experimental Section*) was applied to further evaluate the binding parameters (Figure S85 and Table 5). The calculated binding constants (*K*_b_) for the interaction of the complexes with BSA fall within the range of 1.97 × 10^5^ to 2.74 × 10^6^ M^–1^. These values are comparable to those reported in the literature for other Ir­(III) complexes and suggest a moderate interaction with the protein.^55–59^ In all cases, the binding site number (*n*) was close to 1.0.

### 2.10 Molecular Docking

The emission quenching of BSA upon increasing concentrations of the complexes provides experimental confirmation of their interaction. However, to gain further insight into the most favorable binding sites and the nature of these interactions, molecular docking studies were performed.

BSA features three main ligand-binding regions: Sudlow site I, Sudlow site II and site III. Interestingly, the two tryptophan residues responsible for the proteins’ intrinsic fluorescence are located within these regions: TRP134 resides on subdomain IA at the hydrophilic surface of site III, whereas TRP213 is buried within the hydrophobic cavity of subdomain IIA (Sudlow site I).^60^ Based on these features, these sites were anticipated to serve as the primary docking regions for the complexes. Therefore, the preferred binding site was identified by comparing the predicted binding energies obtained for each TRP region and by evaluating the consistency of the binding modes across the poses generated in the docking runs, with the lowest-energy representative conformation selected for further analysis (Table S7).

Once the calculations were completed, site III was identified as the preferential binding site (Figures 8 and S86). Complex **C1w** fits neatly within the cavity, whereas the steric hindrance from the morpholine moiety in **C3w** and the second Ir­(III) orthometalation in **C5w** push them toward the outer region of the site, which also coincides with the protein surface. The reliability of the method to produce meaningful binding poses was supported by a redocking test of the cocrystallized triethylene glycol (TEG) molecule within the BSA structure (RMSD = 2.56, Figure S87). A summary of the interactions between the complexes and the protein residues is presented in Table S8. In general, all complexes form hydrogen bonds with nearby polar residues, particularly glutamic acid (GLU), via their aqua ligands or the O atoms of the anisole or morpholine units. Moreover, these GLU residues play a key role in establishing electrostatic attractions with the positively charged Ir­(III) centers, as their negatively charged carboxylate groups interact favorably with the metal cores. However, notable differences arise among the complexes when considering the interactions between their planar biquem units and the residues of site III. The similar orientation of **C3w** and **C5w** leads to a combination of electrostatic π–anion, π–cation, and hydrophobic π–alkyl interactions with their local environment. In contrast, complex **C1w** is stabilized within the site III cavity mainly through hydrophobic contacts, including π–σ, π–π T-shaped, π–alkyl, and pure alkyl interactions, in which the contribution of the anisole unit is also significant. Detailed 3D and 2D representations of the interactions are shown in Figure S86.

**Figure 8:**
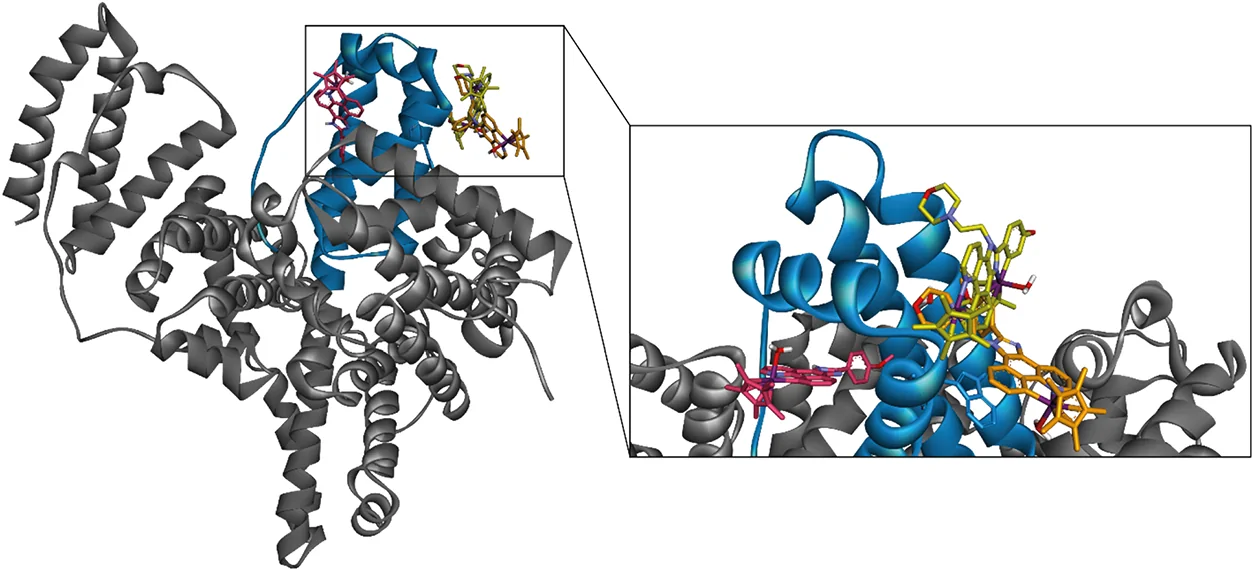
Superposition of complexes **C1w**, **C3w**, and **C5w** within BSA. Inset: zoom highlighting the arrangement of the complexes in site III (blue), with **C1w** (pink), **C3w** (orange), and **C5w** (yellow). TRP134 residue is displayed on sticks style.

Given the predicted absence of direct interactions with TRP134, the observed experimental fluorescence quenching is likely mediated by a distance-dependent, through-space mechanism. This behavior is characteristic of Förster Resonance Energy Transfer (FRET), a nonradiative energy transfer process that depends strongly on the distance between the fluorescent donor (TRP134) and the acceptor (the complex).^60^

### 2.11 Cytotoxic Activity

The cytotoxic activity of the complexes was determined in human lung carcinoma (A549), pancreatic carcinoma (BxPC-3), and cervical carcinoma (HeLa) cell lines, as well as in nonmalignant lung fibroblasts (MRC-5), using the standard MTT (3-[4,5-dimethylthiazol-2-yl]-2,5-diphenyl tetrazolium bromide) assay. As summarized in Table 6, all five Ir­(III) complexes displayed cytotoxicity across all tested cell lines after 48 h of treatment, with IC_50_ values in the low micromolar range. Comparable IC_50_ values were obtained for malignant and nonmalignant cells, indicating a lack of intrinsic cytotoxic selectivity toward cancer cells, a behavior comparable to that of cisplatin. Notably, the IC_50_ values determined for these complexes were also in the same range as those of the cyclometalated Ir­(III) complex [Cp*Ir­(ppy)­Cl] (ppy = 2-phenylpyridine), reported by Sadler, which is structurally and mechanistically more closely related to the new Ir­(III) complexes.^19^

**Table 6:** Cytotoxicity of the Ir­(III) Complexes on Cancer and Non-Malignant Cells

	IC_50_ (μM)^a^			
Cell line	A549	BxPC-3	HeLa	MRC-5
**C1w**	19.8 ± 3.2	18.8 ± 3.7	11.4 ± 1.1	15.4 ± 0.6
**C2w**	28.1 ± 1.8	32.7 ± 4.1	12.4 ± 0.9	15.7 ± 9.6
**C3w**	13.0 ± 1.3	49.2 ± 0.5	8.8 ± 0.7	13.4 ± 1.9
**C4**	10.8 ± 3.2	29.4 ± 9.2	3.4 ± 2.4	31.8 ± 12.2
**C5w**	9.2 ± 1.8	19.6 ± 1.0	28.2 ± 2.8	35.0 ± 0.1
[Cp*Ir(ppy)Cl] ^b^	8.7 ± 2.1	12.9 ± 2.1	7.7 ± 1.4	n.d.
**Cisplatin**	5.9 ± 1.2	1.0 ± 0.1	1.5 ± 0.3	5.3 ± 0.4

a Cell viability was assessed after 48 h of treatment by MTT assays. Data represents mean ± standard deviation (SD) of at least three independent experiments, each performed in triplicate.

b From ref 19 n.d.: not determined.

Overall, no significant differences in cytotoxicity were observed among the complexes, suggesting that neither morpholine functionalization nor the presence of the trifluoroacetate ligand has a major impact on the cytotoxic response under the conditions tested.

Prompted by previous reports describing remarkable light-induced anticancer activity of half-sandwich Ir­(III) complexes,^37,61–63^ the biological response of the new Ir­(III) complexes under light irradiation was next assessed using A549 cells. Blue light irradiation (460 nm, 1 h, 24.1 J cm^–2^) was selected based on the UV–vis absorption spectra of the compounds. Under these conditions only a slight increase in cytotoxicity, of approximately 2-fold, was obtained for methoxy-containing complexes **C1w** and **C3w**, as shown in Table S9.

### 2.12 Intracellular ROS Generation

Intracellular ROS levels were analyzed using the dichlorodihydrofluorescein diacetate (H_2_DCFDA) probe, a nonfluorescent molecule that is oxidized by intracellular ROS to yield the green fluorescent dichlorofluorescein (DCF). A549 cells were treated with the complexes at an equimolar concentration of 25 μM for 4 h, and the intracellular fluorescence intensity was measured by flow cytometry. As shown in Figure 9, treatment with all complexes resulted in a significant increase of fluorescence intensity compared to untreated control cells, indicating elevated intracellular ROS levels, with increases of up to 3.4-fold. These results support the hypothesis that the complexes disrupt the redox balance inside cancer cells, weakening the antioxidant defense system and leading intracellular ROS levels elevation, which may contribute to their cytotoxic mechanism.

**Figure 9:**
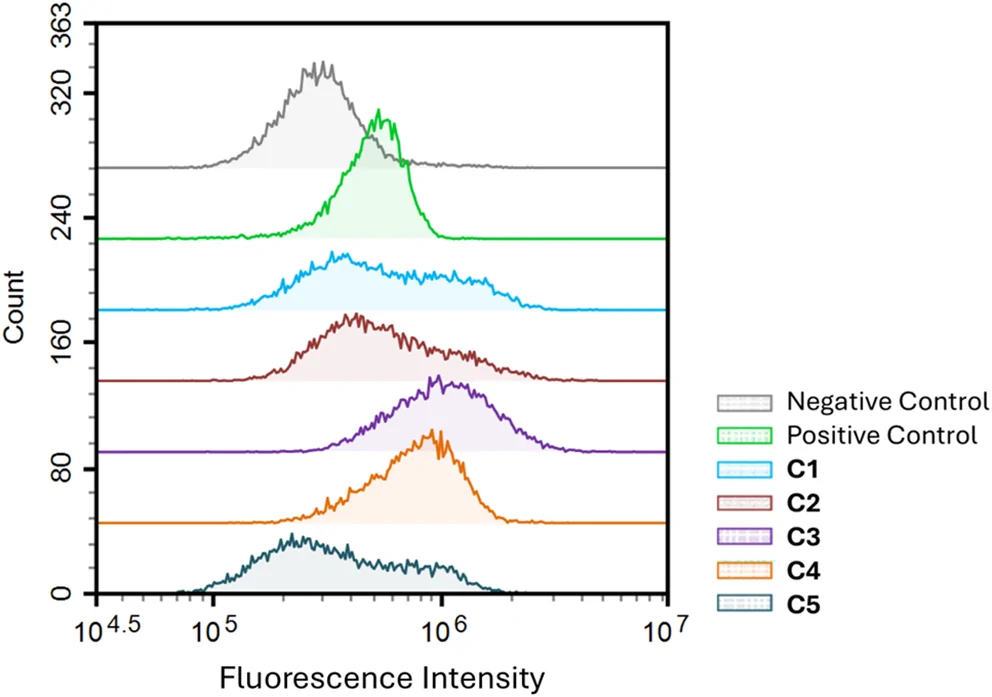
Flow cytometry histograms of intracellular ROS levels in A549 cells following 4 h treatment with the Ir­(III) complexes at 25 μM. Cells incubated with culture medium alone were used as the negative control, and with 20 μM FeCl_2_ and 1.75% H_2_O_2_ as the positive control. ROS were detected using the H_2_DCFDA probe, and fluorescence intensity of 10,000 cells was measured per sample.

### 2.13 Organelle Damage

Next, the specific organelle damage induced by the complexes was evaluated. Mitochondria and lysosomes are among the major intracellular targets of Ir­(III) complexes.^64–68^ In accordance, the effects of the compounds on these organelles were investigated using confocal fluorescence microscopy and specific probes. Mitochondria were first examined since cationic Ir­(III) compounds tend to accumulate preferentially in this organelle due to the negative potential across the inner mitochondrial membrane. The integrity of mitochondria was assessed after 24 h of treatment with the complexes at their IC_50_ values using MitoTracker Red CMXRos, a fluorescent red dye that selectively accumulates in healthy mitochondria.^69^ As illustrated in Figure 10, control cells displayed intense red fluorescence, indicative of functional mitochondria. Similar staining was observed in cells treated with cisplatin, consistent with its established mechanism of action, which targets nuclear DNA rather than mitochondria. In contrast, treatment with the Ir­(III) complexes markedly reduced the red fluorescent staining, revealing that they significantly disrupt mitochondrial integrity.

**Figure 10:**
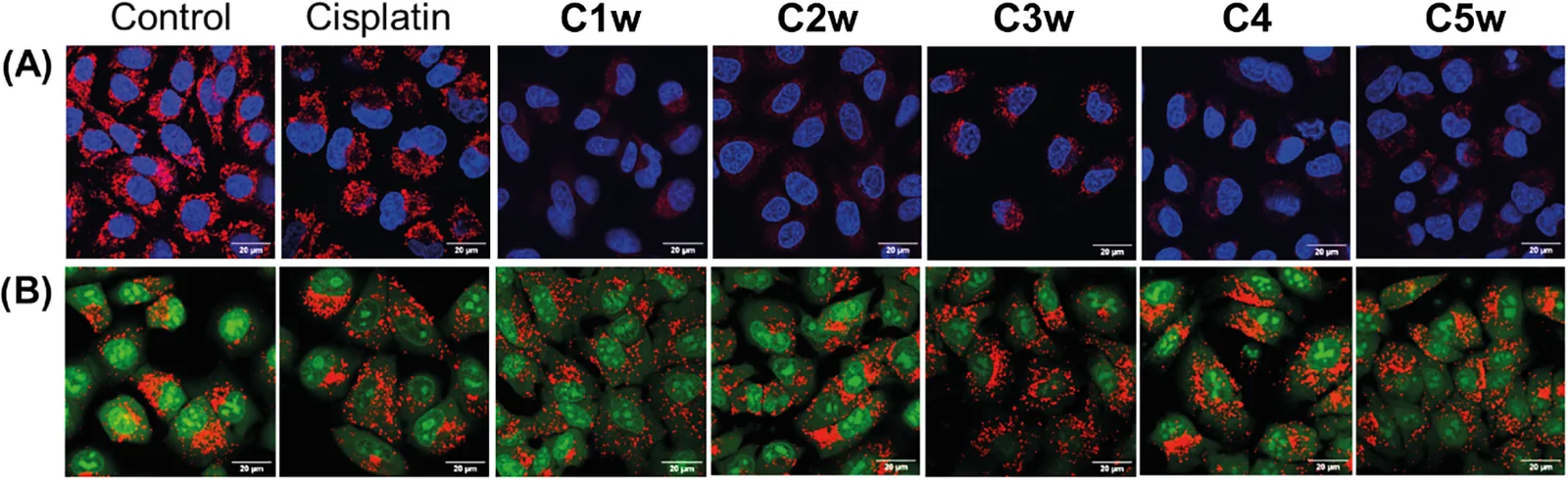
Evaluation of mitochondrial and lysosomal damage induced by the new Ir­(III) complexes. A549 cells were treated with the indicated complexes at their corresponding IC_50_ values or with cisplatin (5 μM) for 24 h at 37 °C, followed by staining with organelle-specific fluorescent dyes. (A) Confocal microscopy images of cells stained with Hoechst (λ_exc_ = 400 nm, λ_em_ = 450 nm) to visualize cell nuclei, and with MitoTracker Red CMXRos (λ_exc_ = 543 nm, λ_em_ = 595 nm) to label functional mitochondria. (B) Confocal microscopy images of cells stained with Acridine Orange (λ_ex_ = 488 nm). Cell cytoplasm and nucleoli were visualized in green (λ_em_ = 510 nm) while acidic cellular compartments, such as lysosomes, were visualized in red (λ_em_ = 625 nm).

Similarly, lysosomal integrity was assessed using Acridine Orange (AO), a cationic fluorescent dye whose emission depends on the environment. In acidic compartments as lysosomes, it accumulates at high concentrations, emitting a red fluorescent signal. In contrast, when AO is distributed in the cytosol or nucleus, where the pH is neutral, it intercalates into DNA and RNA and emits green fluorescence. Thus, the loss of red signal indicates lysosomal damage and membrane permeabilization. Microscopy experiments showed a similar punctate red fluorescence staining in both control and all treated cells (Figure 10), indicating that lysosomes remained unaffected upon the treatment with the Ir­(III) complexes. Consequently, lysosomes do not appear to be the primary targets of these compounds.

Overall, these results indicate that the new Ir­(III) complexes localize to mitochondria regardless of the presence of the morpholine moiety. This behavior stands in contrast to earlier Ir based systems incorporating morpholine units,^20,25,70,71^ even those bearing analogous N^∧^N ligands that have been reported as long-lived probes for lysosomal tracking,^36^ suggesting that the organelle specificity in the systems presented in this work is primarily determined by the Cp*Ir fragment. The observed mitochondrial-targeted mode of action exhibited by the new Ir­(III) complexes is clearly distinct from that of conventional chemotherapeutic drugs, such as cisplatin. It should be noted that the mitochondrial damage appears to result from a direct interaction between the complexes and the mitochondria rather than from secondary apoptotic processes associated with apoptosis, since no mitochondrial damage was observed following cisplatin treatment, which induces cell apoptosis. Thus, this different mode of action may be particularly advantageous for the treatment of cancer cells resistant to conventional DNA-targeting drugs.

## 3 Conclusions

In this study, four benzo­[*h*]­imidazoquinoline derivatives and five new half-sandwich Ir­(III) complexes incorporating such ligands were successfully synthesized and characterized. Different substituents were incorporated into the ligands to fine-tune the structural and physicochemical properties of the resulting complexes. The substituents on the aryl ring modulated key chemical parameters such as reactivity, ligand basicity, p*K*_a_ and hydrolysis kinetics at the Z-position reflecting the importance of the push–pull of electronic density on the 2-carbon of the imidazole ring. The presence of a morpholine substituent on the imidazole ring, and particularly a methoxy group on the aryl ring, promoted double orthometalation, yielding the complex with the highest hydrophilicity and the greatest stability among the chloride derivatives. The observation of this unprecedented double orthometalation in this class of ligands opens the way for synthesizing other types of bimetallic complexes, with the possibility of enhancing the second C–H activation by placing an OMe substituent in the *para* position of the group to be activated. Furthermore, the incorporation of two emissive units, such as biscyclometalated Ir­(III) fragments, in this asymmetric ligand, could yield complexes with intriguing photophysical properties.

All five Ir­(III) complexes displayed cytotoxicity against three different cancer cell lines with IC_50_ values in the low micromolar range. However, despite the inclusion of morpholine, none of the complexes accumulated in lysosomes, indicating that the Cp*Ir­(III) fragment predominantly governs organelle targeting. The complexes disrupted mitochondrial function, suggesting a mechanism of action distinct from that of cisplatin. These findings highlight their potential as alternative agents for the treatment of tumors exhibiting cisplatin resistance. Furthermore, the complexes displayed reactivity with biomolecules such as NADH and GSH, thereby impairing the cellular antioxidant defense system and sensitizing tumor cells to chemotherapy. The observed increase in intracellular ROS levels supports this mechanism. In addition, molecular docking studies revealed moderate interactions between the complexes and serum albumin, particularly involving the tryptophan-134 residue. Such moderate binding prevents the complexes from being sequestered by serum proteins, thereby maintaining their availability for biological activity. Overall, these findings indicate that these Ir­(III) complexes are promising candidates for further development as anticancer agents with distinct mechanisms of action capable of overcoming drug resistance.

## 4 Materials and Methods

### 4.1 General Information

All synthetic manipulations were performed under an inert, oxygen-free, dry nitrogen atmosphere using standard Schlenk techniques. All metal complexes were synthesized in dark conditions and protected from light using aluminum foil throughout each step of synthesis, isolation and characterization. Solvents were dried and distilled under nitrogen atmosphere before use. Flash chromatography was carried out using neutral alumina (Acros, Brockmann I, 60A, 40–300 μM). Elemental analyses were performed on a FlashEA112 microanalyzer (ThermoFinnigan). ESI+ or APCI+ MS (mass spectrometry) data were recorded on a Thermo MAT95XP mass spectrometer using DCM or DMF:DCM (1:9) as the sample solvent. UV–vis absorption spectra were recorded on a Secomam Uvikon XS spectrophotometer using the LabPower Junior program. Fluorescence excitation and emission spectra were recorded on a PTI Quanta Master TM spectrofluorometer from Photon Technology International (PTI) equipped with a 75 W xenon short arc lamp and a model 814PTM detection system. Multinuclear NMR (nuclear magnetic resonance) spectra were recorded at 298 K on a Varian Unity Inova 400 or on a Varian Inova 500 spectrometer. Chemical shift values (δ) are reported in ppm (parts per million) and coupling constants (*J*) in Hertz. The splitting of proton resonances is defined as follows: s = singlet, d = doublet, t = triplet, q = quadruplet, m = multiplet, bs = broad singlet. 2D-NMR spectra such as ^1^H–^1^H gCOSY, ^1^H–^13^C gHSQC and ^1^H–^13^C gHMBC were recorded using standard pulse–pulse sequences. ^1^H NMR chemical shifts were internally referenced to (C*H*D_2_)­(CD_3_)­SO (2.50 ppm) for DMSO-*d*_6_ via the residual proton solvent resonances. The probe temperature (±1 K) was controlled by a standard unit calibrated with a methanol reference. All NMR data processing was performed using MestReNova version 12.0.0. The atom-numbering scheme for the ligands and complexes is shown in Figure 11.

**Figure 11:**
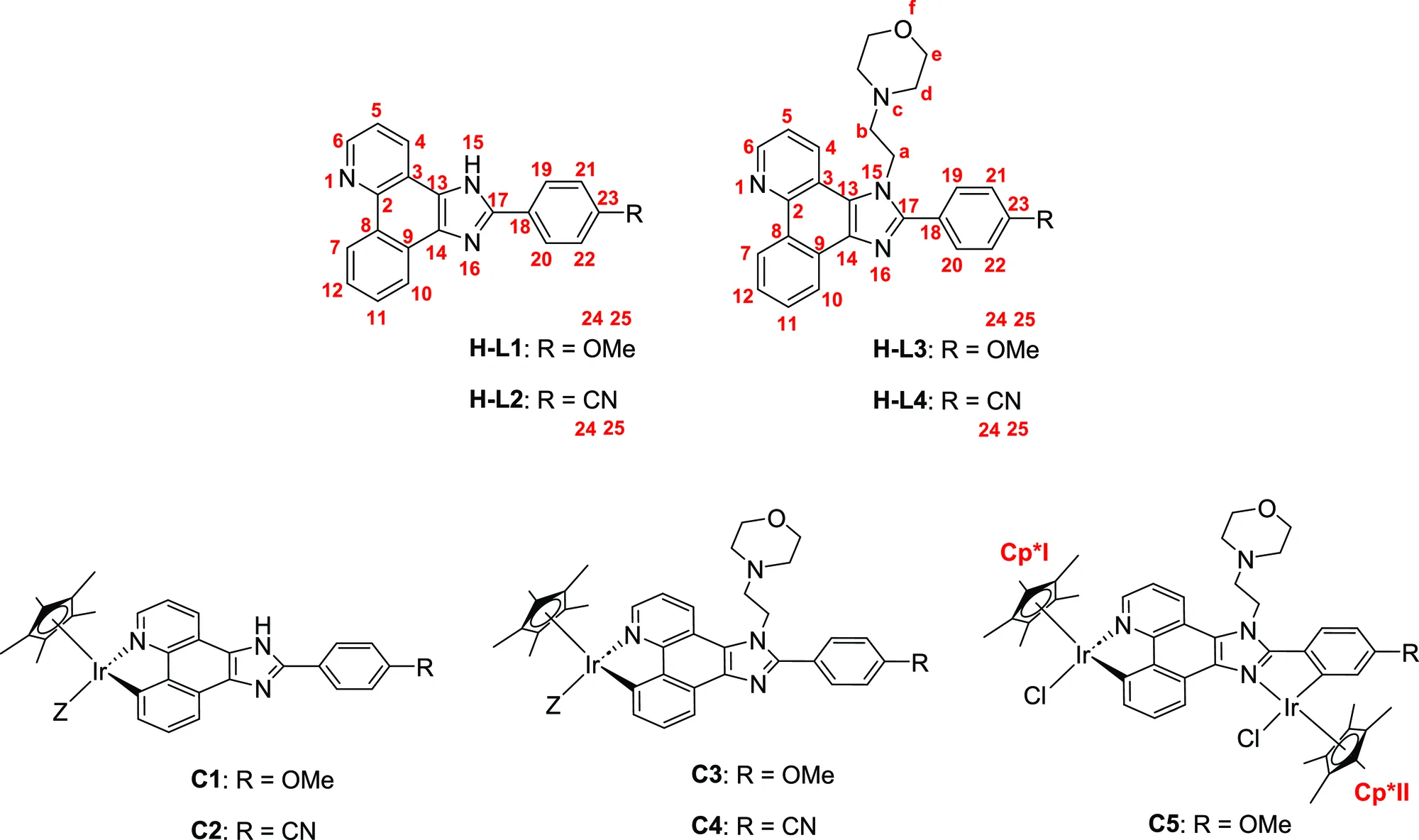
Atom numbering of the new ligands and complexes.

Unless otherwise stated, reagents and solvents were reagent quality and commercially available. Benzo­[*h*]­quinoline-5,6-dione was prepared as reported.^38^ Proligands **H-L1** and **H-L2** were prepared by adapting the methodology described by Solís et al. for other similar C^∧^N ligands.^35^ The modifications of **H-L1** and **H-L2** to prepare **H-L3** and **H-L4** were carried out by adapting a literature procedure.^20^ The precursor dimer [Cp*IrCl­(μ-Cl)]_2_ was purchased from Tokyo Chemical Industry (TCI); ammonium acetate, silver trifluoroacetate and triethylamine were purchased from Alfa Aesar; 4-benzonitrile and 4-methoxibenzaldehyde were purchased from Acros; 4-(2-chloroethyl)­morpholine was purchased from Thermo Scientific; sodium carbonate was purchased from Panreac and sodium hydride was purchased from Sigma-Aldrich. All of them were used without further purification. Deuterated solvents were purchased from Eurisotop. Conventional solvents such as DCM (Scharlab), DCE (Scharlab), diethyl ether (Fisher Scientific), acetone (Fisher Scientific), pentane (Scharlab), DMSO (Scharlab), water (Scharlab) and acetic acid (Scharlab) were degassed and, in some cases, distilled prior to use. Sodium hydroxide and hydrochloride acid were purchased from Panreac.

No unexpected or unusually high safety hazards were encountered in this work.

### 4.2 Synthesis and Characterization of the New Complexes

#### 4.2.1 2-(4-Methoxyphenyl)-3*H*-benzo­[*h*]­imidazo­[4,5-*f*]­quinoline, H-L1

In a 100 mL Schlenk, benzo­[*h*]­quinoline-5,6-dione (0.64 g, 3.1 mmol, 1.2 equiv), 4-methoxybenzaldehyde (0.35 g, 2.6 mmol, 1.0 equiv) and ammonium acetate (4.84 g, 63 mmol, 24 equiv) were stirred overnight under reflux in glacial acetic acid (30 mL) under inert atmosphere, using an oil bath as the heart source. After cooling to room temperature, water (50 mL) was added to the mixture and a brown solid was obtained. The excess of acetic acid was neutralized with ammonia. The solid was filtered and washed with aqueous NaHCO_3_ (3 × 50 mL) and water (3 × 50 mL) and dried under vacuum. The product was obtained as a brown solid (0.82 g, 2.5 mmol, 96%). ^**1**^ **H NMR (400 MHz, DMSO-** ***d***_***6***_ **)**: δ 9.25 (dd, ^3^ *J* = 8.3 Hz, ^4^ *J* = 1.3 Hz, 1H, H^10^), 8.95 (dd, ^3^ *J* = 4.4 Hz, ^4^ *J* = 1.8 Hz, 1H, H^6^), 8.88 (dd, ^3^ *J* = 8.1 Hz, ^4^ *J* = 1.8 Hz, 1H, H^4^), 8.56 (dd, ^3^ *J* = 8.1 Hz, ^4^ *J* = 1.3 Hz, 1H, H^7^), 8.27–8.22 (m, 2H, H^19^ and H^20^), 7.83 (ddd, ^3^ *J* = 8.1, 7.0 Hz, ^4^ *J* = 1.4 Hz, 1H, H^12^), 7.75 (dd, ^3^ *J* = 8.1, 4.4 Hz, 1H, H^5^), 7.68 (ddd, ^3^ *J* = 8.3, 7.0 Hz, ^4^ *J* = 1.3 Hz, 1H, H^11^), 7.19–7.14 (m, 2H, H^21^ and H^22^), 3.86 (s, 3H, H^25^). ^**13**^ **C­{** ^**1**^ **H} NMR (126 MHz, DMSO-** ***d***_**6**_ **):** δ 160.8 (C, C^23^), 150.5 (C, C^17^), 147.4 (CH, C^6^), 144.2 (C, C^2^), 130.0 (CH, C^4^), 129.1 (CH, C^11^), 128.9 (C, C^8^), 128.3 (CH, C^19^ and C^20^), 125.6 (CH, C^7^ and C^12^), 123.3 (C, C^18^), 122.8 (CH, C^5^), 122.0 (CH, C^10^), 114.9 (CH, C^21^ and C^22^), 55.8 (C, C^25^). **Elemental Analysis** calculated for C_21_H_15_N_3_O·(CH_2_Cl_2_)_0.7_ (384.83 g/mol): C 67.73, H 4.30, N 10.92; found: C 68.02, H 4.21, N 10.8. **MS (ESI+, DMF:DCM 1:9)**: *m*/*z* calculated for C_21_H_16_N_3_O [M + H]^+^: 326.1288, found: 326.1281; *m*/*z* calculated for C_42_H_31_N_6_O_2_ [M_2_+H]^+^: 651.2503, found: 651.2488.

#### 4.2.2 4-(3*H*-Benzo­[*h*]­imidazo­[4,5-*f*]­quinolin-2-yl)­benzonitrile, H-L2

Proligand **H-L2** was prepared in a similar way than proligand **H-L1** using benzo­[*h*]­quinoline-5,6-dione (0.62 g, 3.0 mmol, 1.2 equiv), benzonitrile (0.33 g, 2.5 mmol, 1.0 equiv) and ammonium acetate (4.64 g, 60 mmol, 24 equiv). The compound was obtained as a pale yellow solid (0.74 g, 2.3 mmol, 93%). ^**1**^ **H NMR (500 MHz, DMSO-** ***d***_**6**_ **):** δ 13.83 (bs, 1 H, H^16^), 9.23 (d, ^3^ *J* = 7.1 Hz, 1H, H^10^), 8.96 (dd, ^3^ *J* = 4.2 Hz, ^4^ *J* = 1.5 Hz, 1H, H^6^), 8.84 (d, ^3^ *J* = 7.6 Hz, 1H, H^4^), 8.53 (d, ^3^ *J* = 7.1 Hz, 1H, H^7^), 8.43 (d, ^3^ *J* = 8.2 Hz, 2H, H^19^ and H^20^), 8.05 (d, ^3^ *J* = 8.4 Hz, 2H, H^21^ and H^22^), 7.84 (t, ^3^ *J* = 6.3 Hz, 1H, H^12^), 7.75 (m, 1H, H^5^), 7.71 (t, ^3^ *J* = 7.6 Hz, 1H, H^11^). ^**13**^ **C­{** ^**1**^ **H} NMR (126 MHz, DMSO-** ***d***_**6**_ **):** δ 147.8 (C, C^17^), 147.5 (CH, C^6^), 144.1 (C, C^2^), 134.2 (C, C^18^), 133.0 (CH, C^21^ and C^22^), 129.7 (CH, C^4^), 128.8 (CH, C^11^), 126.6 (CH, C^19^ and C^20^), 125.9 (CH and C, C^12^, C^13^, and C^14^), 125.3 (C, C^7^ and C^9^), 122.5 (CH and C, C^5^ and C^8^), 121.7 (C and CH, C^3^ and C^10^), 118.8 (C, C^24^), 111.3 (C, C^23^). **Elemental Analysis** calculated for C_21_H_12_N_4_·(H_2_O)_2_ (356.39 g/mol): C 70.78, H 4.53, N 15.72; found: C 70.68, H 4.47, N 15.69. **MS (ESI+, DMF:DCM 1:9)**: *m*/*z* calculated for C_21_H_13_N_4_ [M + H]^+^: 321.1135, found: 321.1125.

#### 4.2.3 4-(2-(2-(4-Methoxyphenyl)-3*H*-benzo­[*h*]­imidazo­[4,5-*f*]­quinolin-3-yl)­ethyl)­morpholine, H-L3

Proligand **H-L1** (0.2004 g, 0.6 mmol, 1.0 equiv) was dissolved in anhydride THF (50 mL) in a 500 mL round-bottom flask under inert atmosphere and heated at 60 °C in an oil bath. Then, NaH was added (0.3710 g, 9.3 mmol, 15.0 equiv) and the mixture was stirred. When the effervescence is over, a solution of 4-(2-chloroethyl) morpholine (0.2890 g, 1.2 mmol, 2.0 equiv) in THF was added dropwise. The mixture was stirred for 5 days. Then, the solvent was evaporated, and the residue was redissolved in EtOAc (200 mL) and filtered. The obtained solution was concentrated to give an oil, which is precipitated with ether:pentane (1:1, 10 mL). The precipitate was filtered and dried under vacuum to obtain a pale orange solid (0.1320 g, 0.3 mmol, 49%). Two isomers, A and B, were observed in a 65:35 (A:B) ratio. ^**1**^ **H NMR (500 MHz, DMSO-** ***d***_***6***_ **)**: δ 9.43 (dd, ^3^ *J* = 8.3 Hz, ^4^ *J* = 1.5 Hz, 1H, H^10,A^), 9.25 (dd, ^3^ *J* = 8.5 Hz, ^4^ *J =* 1.3 Hz, 1H, H^10,B^), 8.98 (m, 2H, H^6^), 8.87 (dd, ^3^ *J* = 8.0 Hz, ^4^ *J* = 1.8 Hz, 1H, H^4,A^), 8.83 (dd, ^3^ *J* = 8.5 Hz, ^4^ *J* = 1.6 Hz, 1H, H^4,B^), 8.56 (dd, ^3^ *J* = 8.0 Hz, ^4^ *J* = 1.4 Hz, 1H, H^7,B^), 8.46 (d, ^3^ *J* = 8.2 Hz, 1H, H^7,A^), 7.87 (ddd, ^3^ *J* = 8.4, 6.9 Hz, ^4^ *J* = 1.5 Hz, 1H, H^12,A^), 7.81 (m, 1H, H^12,B^), 7.80–7.67 (m, 8H, H^11^, H^5^, H^20^, and H^19^), 7.19–7.14 (m, 4H, H^22^ and H^21^), 4.81 (m, 4H, H^a^), 3.87 (s, 6H, H^25^), 3.37 (m, 8H, H^e^), 2.75 (t, ^3^ *J* = 6.3 Hz, 2H, H^b,A^), 2.71 (t, ^3^ *J* = 6.2 Hz, 2H, H^b,B^) 2.12 (m, 8H, H^d^). ^**13**^ **C­{** ^**1**^ **H} NMR (126 MHz, DMSO-** ***d***_***6***_ **)**: δ 160.2, 153.49, 153.46, 147.6, 146.5, 143.9, 143.7, 137.0, 135.8, 131.41, 131.38, 129.7, 129.0, 128.87, 128.82, 128.80, 128.64, 128.0, 125.79, 125.78, 125.53, 125.1, 124.40, 123.94, 122.79, 122.68, 122.1, 121.63, 121.44, 120.7, 118.0, 114.11, 114.09, 66.0, 57.69, 57.52, 55.4, 53.3, 44.07, 43.90. **Elemental Analysis** calculated for C_27_H_26_N_4_O_2_·(H_2_O)_0.4_ (445.74 g/mol): C 72.76, H 6.06, N 12.57; found: C 72.56, H 5.99, N 12.49. **MS (ESI+, DMF:DCM 1:9)**: *m*/*z* calculated for C_27_H_27_N_4_O_2_ [M + H]^+^: 439.2129, found: 439.2131.

#### 4.2.4 4-(3-(2-Morpholinoethyl)-3*H*-benzo­[*h*]­imidazo­[4,5-*f*]­quinolin-2-yl)­benzonitrile, H-L4

Proligand **H-L4** was prepared in a similar way than **H-L3** employing **H-PL2** (0.3000 g, 0.9 mmol, 1.0 equiv), NaH (0.3430 g, 8.6 mmol, 9.6 equiv) and 4-(2-chloroethyl) morpholine (0.3530 g, 1.4 mmol, 1.5 equiv). The compound was obtained as a pale yellow solid (0.2780 g, 0.6 mmol, 69%). Two isomers, A and B, were observed in a 64:36 (A:B) ratio. ^**1**^ **H NMR (400 MHz, DMSO-** ***d***_***6***_ **)**: δ 9.43 (dd, ^3^ *J* = 8.3 Hz, ^4^ *J* = 1.5 Hz, 1H, H^10,A^), 9.25 (d, ^3^ *J* = 8.3 Hz, 1H, H^10,B^), 8.99 (m, 2H, H^6,A^ and H^6,B^), 8.88 (dd, ^3^ *J* = 8.1 Hz, ^4^ *J* = 1.9 Hz, 1H, H^4,A^), 8.85 (m, 1H, H^4,B^), 8.57 (dd, ^3^ *J* = 8.1 Hz, ^4^ *J* = 1.3 Hz, 1H, H^7,B^), 8.47 (d, ^3^ *J* = 8.3 Hz, 1H, H^7,A^), 8.10 (s, 8H, H^20^, H^19^, H^22^, and H^21^), 7.96–7.67 (m, 6H, H^12^, H^11^, and H^5^), 4.83 (m, 4H, H^a^), 3.30 (m, 8H, H^e^), 2.75 (t, ^3^ *J* = 5.8 Hz, 2H, H^b,A^), 2.72 (t, ^3^ *J* = 5.7 Hz, 2H, H^b,B^) 2.08 (m, 8H, H^d^). ^**13**^ **C­{** ^**1**^ **H} NMR (101 MHz, DMSO-** ***d***_***6***_ **):** δ 151.8, 147.9, 147.0, 144.3, 143.8, 137.3, 136.2, 135.19, 135.17, 132.6, 130.85, 130.81, 129.7, 129.32, 129.13, 129.04, 128.99, 128.82, 127.91, 127.76, 126.4, 125.88, 125.85, 125.55, 125.15, 125.04, 123.8, 122.8, 122.3, 121.53, 121.46, 120.97, 118.6, 118.0, 112.0, 65.9, 57.68, 57.51, 53.4, 44.37, 44.19. **Elemental Analysis** calculated for C_27_H_23_N_5_O·(H_2_O)_0.4_ (440.72 g/mol): C 73.58, H 5.44, N 15.89; found: C 73.36, H 5.23, N 15.80. **MS (ESI+, DMF:DCM 1:9)**: *m*/*z* calculated for C_27_H_24_N_5_O [M + H]^+^: 434.1976, found: 434.1975.

#### 4.2.5 [(η^5^-C_5_Me_5_)­Ir­(L1)­Cl], (C1)

In a 50 mL Schlenk flask, a solution of [(η^5^-C_5_Me_5_)­Ir­(μ-Cl)­Cl]_2_ (0.07 g, 0.08 mmol, 1.0 equiv), 2-(4-methoxyphenyl)-3*H*-benzo­[*h*]­imidazo­[4,5-*f*]­quinoline (**H-L1**, 0.05 g, 0.16 mmol, 2.0 equiv), Na_2_CO_3_ (0.02 g, 0.16 mmol, 2.0 equiv) and Ag­(OCOCF_3_) (0.03 g, 0.16 mmol, 2.0 equiv) in degassed DCE (15 mL) was stirred at ambient temperature for 24 h under inert atmosphere. The resulting precipitate (AgCl) was removed by filtration over Celite. To recover all the product, the residue was washed with DCM until the filtrate was colorless. The filtrate was concentrated in vacuo until dryness. Then, the yellow solid was washed with diethyl ether (3 × 10 mL). The product was purified by a flash chromatography with Celite as stationary phase and DCM as mobile phase. The filtrate was concentrated in vacuo until dryness and washed with pentane to give a yellow solid (0.03 g, 0.04 mmol, 49%). The following ^1^H NMR data correspond to the chlorido complex, whereas ^13^C NMR signals pertain to the solvolysis product in DMSO-*d*_6_. ^**1**^ **H NMR (500 MHz, DMSO-** ***d***_**6**_ **):** δ 13.45 (s, 1H, H^15^), 8.98 (d, *J* = 5.3 Hz, 1H, H^6^), 8.79 (d, *J* = 7.9 Hz, 1H, H^4^), 8.24 (m, 2H, H^19^ and H^20^), 8.02 (t, *J* = 7.8 Hz, 1H, H^10^), 7.90 (d, *J* = 7.2 Hz, 1H, H^12^), 7.75 (t, *J* = 7.4 Hz, 1H, H^5^), 7.58 (t, *J* = 7.6 Hz, 1H, H^11^), 7.17 (m, 2H, H^21^ and H^22^), 3.87 (s, 3H), 1.68 (s, 15H, H^27^). ^**13**^ **C NMR (126 MHz, DMSO-** ***d***_**6**_ **):** δ 160.5, 154.4, 154.1, 151.7, 151.6, 151.5, 151.1, 150.7, 150.4, 139.3, 139.1, 138.8, 135.1, 133.2, 132.6, 131.7, 131.4, 130.9, 130.6, 129.7, 128.8, 128.1, 125.7, 124.1, 124.05, 123.95, 123.7, 122.9, 122.7, 118.7, 117.3, 116.7, 114.4, 96.2, 55.4, 8.3. For elemental and MS analysis, the chlorido complex was employed. **Elemental Analysis** calculated for IrC_31_H_29_N_3_OCl·(C_2_H_4_Cl_2_)_0.4_ (726.83 g/mol): C 52.55, H 4.24, N 5.78; found C 52.78, H 3.82, N 5.43. **MS (ESI+, DMF:DCM 1:9):** *m*/*z* calculated for C_31_H_29_IrN_3_O [M-Cl]^+^: 652.1935, found: 652.1935.

#### 4.2.6 [(η^5^-C_5_Me_5_)­Ir­(L2)­Cl], (C2)

In a 50 mL Schlenk flask, a solution of [(η^5^-C_5_Me_5_)­Ir­(μ-Cl)­Cl]_2_ (0.06 g, 0.07 mmol, 0.5 equiv), 4-(3*H*-benzo­[*h*]­imidazo­[4,5-*f*]­quinolin-2-yl)­benzonitrile (**H-L2**, 0.047 g, 0.14 mmol, 1.0 equiv), Na_2_CO_3_ (0.02 g, 0.17 mmol, 1.1 equiv) and Ag­(OCOCF_3_) (0.04 g, 0.16 mmol, 1.1 equiv) in degassed DCE (15 mL) was stirred under reflux for 4.5 h, using an oil bath as the heat source. The resulting precipitate (AgCl) was removed by filtration over Celite. To recover all the product, the residue was resuspended in DCM and filtered. The filtrate was concentrated in vacuo until dryness. Then, the orange solid was washed with acetone (3 × 50 mL) until the filtrate was colorless. The product was washed with pentane and dried under vacuum to give a yellow solid (0.06 g, 0.09 mmol, 58%). The following ^1^H NMR data correspond to the chlorido complex, whereas ^13^C­{^1^H} NMR signals pertain to the solvolysis product in DMSO-*d*_6_. ^**1**^ **H NMR (500 MHz, DMSO-** ***d***_***6***_ **)**: δ 13.89 (s, 1H, H^15^), 9.01 (dd, *J* = 5.5, 1.4 Hz, 1H, H^6^), 8.82 (dd, *J* = 7.9, 1.3 Hz, 1H, H^4^), 8.47 (d, *J* = 8.1 Hz, 2H, H^19^ and H^20^), 8.10 (m, 2H, H^21^ and H^22^), 8.04 (d, *J* = 7.8 Hz, 1H, H^10^), 7.94 (d, *J* = 7.2 Hz, 1H, H^12^), 7.77 (t, *J* = 6.7 Hz, 1H, H^5^), 7.61 (d, *J* = 7.6 Hz, 1H, H^11*l*^), 1.68 (s, 15H, H^27^). ^**13**^ **C­{** ^**1**^ **H} NMR (126 MHz, DMSO-** ***d***_**6**_ **):** δ 154.8, 151.8, 151.2, 149.0, 139.4, 134.2, 133.0, 130.9, 127.0, 124.2, 118.7, 111.5, 96.2, 8.3. For elemental and MS analysis, the chlorido complex was employed. **Elemental Analysis** calculated for IrC_31_H_26_N_4_Cl·(CH_2_Cl_2_)_0.7_ (741.69 g/mol): C 51.34, H 3.72, N 7.55; found C 51.69, H 3.52, N 7.20. **MS (ESI+, DMF:DCM 1:9):** *m*/*z* calculated for C_31_H_26_IrN_4_ [M-Cl]^+^: 647.1782, found: 647.1781.

#### 4.2.7 [(η^5^-C_5_Me_5_)­Ir­(L3)­Cl], (C3)

In a 100 mL Schlenk flask, a solution of [(η^5^-C_5_Me_5_)­Ir­(μ-Cl)­Cl]_2_ (0.05 g, 0.06 mmol, 0.5 equiv), **H-L3** (0.06 g, 0.13 mmol, 1.0 equiv), Na_2_CO_3_ (0.01 g, 0.12 mmol, 1.0 equiv) and Ag­(OCOCF_3_) (0.03 g, 0.13 mmol, 1.0 equiv) in degassed DCE (25 mL) was stirred for 1 h at 0 °C. The resulting precipitate (AgCl) was removed by filtration over Celite. To recover all the product, the residue was resuspended in DCM and filtered. The filtrate was concentrated in vacuo until dryness. The product was purified by preparative aluminum oxide TLC with DCM:MeOH (99.5:0.5). The product was washed with diethyl ether and pentane and dried under vacuum to give a yellow solid (0.03 g, 0.04 mmol, 31%). The following ^1^H NMR data correspond to the chlorido complex, whereas ^13^C­{^1^H} NMR signals pertain to the solvolysis product in DMSO-*d*_6_. Two isomers, A and B, were observed in a 7:3 (A:B) ratio. ^**1**^ **H NMR of isomer A (500 MHz, DMSO** ***-d***_***6***_ **)**: δ 9.01 (dd, ^3^ *J* = 5.3 Hz, ^4^ *J* = 1.0 Hz, 1H, H^6^), 8.79 (dd, ^3^ *J* = 8.0 Hz, ^4^ *J* = 1.4 Hz, 1H, H^4^), 7.99 (d, ^3^ *J* = 8.5 Hz, 1H, H^10^ or H^12^), 7.91 (d, ^3^ *J* = 7.7 Hz, 1H, H^10^ or H^12^), 7.76 (m, 3H, H^5^, H^19^ and H^20^), 7.57 (t, ^3^ *J* = 7.7 Hz, 1H, H^11^), 7.16 (m, 2H, H^21^ and H^22^), 4.80 (m, 2H, H^a^), 3.87 (s, 3H, H^25^), 3.40 (m, 4H, H^e^), 2.75 (t, ^3^ *J* = 5.9 Hz, 2H, H^b^), 2.15 (m, 4H, H^d^), 1.68 (s, 15H, H^27^). ^**1**^ **H NMR of isomer B (500 MHz, DMSO** ***-d***_***6***_ **)**: δ 9.01 (dd, ^3^ *J* = 5.2 Hz, ^4^ *J* = 1.1 Hz, 1H, H^6^), 8.79 (dd, ^3^ *J* = 8.0 Hz, ^4^ *J* = 1.4 Hz, 1H, H^4^), 7.95 (2 × d, ^3^ *J* = 7.7 Hz, ^3^ *J* = 7.3 Hz, 2H, H^10^ and H^12^), 7.76 (m, 3H, H^5^, H^21^, and H^22^), 7.61 (t, ^3^ *J* = 7.8 Hz, 1H, H^11^), 7.16 (m, 2H, H^19^ and H^20^), 4.80 (m, 2H, H^a^), 3.87 (s, 3H, H^25^), 3.40 (m, 4H, H^e^), 2.80 (t, ^3^ *J* = 6.0 Hz, 2H, H^b^), 2.18 (m, 4H, H^d^), 1.68 (s, 15H, H^27^). ^**13**^ **C­{** ^**1**^ **H} NMR (126 MHz, DMSO-** ***d***_***6***_ **)**: δ 160.3, 154.5, 154.3, 154.1, 152.8, 151.4, 139.2, 138.9, 135.2, 132.8, 132.1, 131.4, 130.9, 128.1, 124.4, 124.3, 123.8, 122.8, 122.5, 116.1, 114.2, 96.3, 66.0, 57.8, 55.5, 53.3, 43.8, 8.4. For elemental and MS analysis, the chlorido complex was employed. **Elemental Analysis** calculated for C_37_H_40_ClIrN_4_O_2_·(H_2_O)_2_ (836.44 g/mol): C 53.13, H 5.30, N 6.70; found: C 52.98, H 5.20, N 6.62. **MS (ESI+, DCM)**: *m*/*z* calculated for C_37_H_40_IrN_4_O_2_ [M-Cl]^+^: 765.2775, found: 765.2758.

#### 4.2.8 [(η^5^-C_5_Me_5_)­Ir­(L4)­(OCOCF_3_)], (C4)

In a 100 mL Schlenk flask, a solution of [(η^5^-C_5_Me_5_)­Ir­(μ-Cl)­Cl]_2_ (0.08 g, 0.1 mmol, 0.5 equiv), **H-L4** (0.09 g, 0.2 mmol, 1.0 equiv), Na_2_CO_3_ (0.04 g, 0.4 mmol, 2.0 equiv) and Ag­(OCOCF_3_) (0.09 g, 0.4 mmol, 2.0 equiv) in degassed DCE (25 mL) was stirred at room temperature for 6 days. The resulting precipitate (AgCl) was removed by filtration over Celite. To recover all the product, the residue was resuspended in DCM and filtered. The filtrate was concentrated in vacuo until dryness. Then, the product was filtered through a short pad of Celite with DCM, concentrated under vacuum and washed with diethyl ether and pentane. The product was dried under vacuum to give an orange solid (0.08 g, 0.09 mmol, 46%). Two isomers, A and B, were observed in a 65:35 (A:B) ratio. ^**1**^ **H NMR of isomer A (400 MHz, DMSO** ***-d***_***6***_ **)**: δ 9.10 (dd, ^3^ *J* = 5.5 Hz, ^4^ *J* = 1.2 Hz, 1H, H^6^), 9.06 (dd, ^3^ *J* = 8.1 Hz, ^4^ *J* = 1.2 Hz, 1H, H^4^), 8.25 (d, ^3^ *J* = 8.3 Hz, 1H, H^10^), 8.12 (m, 2H, H^19^ and H^20^ or H^21^ and H^22^), 8.07 (m, 2H, H^19^ and H^20^ or H^21^ and H^22^), 7.96 (m, 1H, H^5^), 7.91 (m, 1H, H^12^), 7.80 (t, ^3^ *J* = 7.9 Hz, 1H, H^11^), 4.85 (m, 2H, H^a^), 3.29 (m, 4H, H^e^), 2.78 (m, 2H, H^b^), 2.12 (m, 4H, H^d^), 1.74 (s, 15H, H^26^). ^**1**^ **H NMR of isomer B (400 MHz, DMSO** ***-d***_***6***_ **)**: δ 9.12 (d, ^3^ *J* = 5.5 Hz, 1H, H^6^), 9.06 (dd, ^3^ *J* = 8.1 Hz, ^4^ *J* = 1.2 Hz, 1H, H^4^), 8.27 (d, ^3^ *J* = 8.3 Hz, 1H, H^10^), 8.12 (m, 2H, H^19^ and H^20^ or H^21^ and H^22^), 8.07 (m, 2H, H^19^ and H^20^ or H^21^ and H^22^), 7.96 (m, 2H, H^5^ and H^12^), 7.77 (t, ^3^ *J* = 7.5 Hz, 1H, H^11^), 4.85 (m, 2H, H^a^), 3.29 (m, 4H, H^e^), 2.78 (m, 2H, H^b^), 2.12 (m, 4H, H^d^), 1.74 (s, 15H, H^26^). ^**13**^ **C­{** ^**1**^ **H} NMR (101 MHz, DMSO-** ***d***_***6***_ **)**: δ 154.9, 154.5, 152.9, 152.7, 152.34, 151.7, 151.5, 139.4, 139.2, 135.5, 135.0, 132.7, 132.6, 131.0, 130.8, 128.6, 124.9, 124.6, 124.1, 122.7, 119.0, 118.5, 116.4, 112.2, 96.3, 88.4, 65.9, 57.9, 57.6, 53.4, 44.1, 8.3. ^**19**^ **F NMR (471 MHz, DMSO-** ***d***_***6***_ **)**: δ −73.40 (s, 3F, CF_3_). **Elemental Analysis** calculated for C_39_H_37_F_3_IrN_5_O_3_·(C_2_H_4_Cl_2_)_0.7_ (932.41 g/mol): C 51.14, H 4.15, N 7.15; found: C 51.38, H 3.71, N 6.97. **MS (ESI+, DCM)**: *m*/*z* calculated for C_37_H_37_IrN_5_O [M-Cl]^+^: 760.2622, found: 760.2632.

#### 4.2.9 [(η^5^-C_5_Me_5_)_2_Ir_2_(L3)­Cl_2_], (C5)

In a 100 mL Schlenk flask, a solution of [(η^5^-C_5_Me_5_)­Ir­(μ-Cl)­Cl]_2_ (0.08 g, 0.1 mmol, 1.0 equiv), **H-L3** (0.04 g, 0.1 mmol, 1.0 equiv), Na_2_CO_3_ (0.02 g, 0.2 mmol, 2.0 equiv) and Ag­(OCOCF_3_) (0.04 g, 0.2 mmol, 2.0 equiv) in degassed DCE (25 mL) was stirred at room temperature for 5 days. The resulting precipitate (AgCl) was removed by filtration over Celite. To recover all the product, the residue was resuspended in DCM and filtered. The filtrate was concentrated in vacuo until dryness and purified by column chromatography using silica gel and DCM:MeOH:NEt_3_ (99:0:1 → 98.2:0.8:1). The pure fraction was washed with water (3 × 10 mL), diethyl ether:pentane (6 × 10 mL) and pentane (3 × 10 mL). The product was dried under vacuum to obtain a yellow solid (0.04 g, 0.04 mmol, 38%). The following ^1^H NMR data correspond to the chlorido species. Three isomers were observed in the Cp* region. ^**1**^ **H NMR (400 MHz, DMSO** ***-d***_***6***_ **)**: A list of all the resonances is indicated. δ 9.75 (d, ^3^ *J* = 8.8 Hz), 9.02 (m), 8.99 (m), 8.91 (m), 8.05 (m), 7.97 (m), 7.83 (m), 7.79 (m), 7.64 (t, ^3^ *J* = 7.7 Hz), 7.57 (t, ^3^ *J* = 7.4 Hz), 7.32 (m), 6.72 (m), 5.24 (m, H^a^), 3.87 (s, H^25^), 3.56 (m, H^e^) 3.03 (m, H^b^), 2.60 (m, H^d^), 1.68 (s, Cp*I), 1.67 (s, Cp*I), 1.66 (s, Cp*I), 1.44 (s, Cp*II), 1.43 (s, Cp*II), 1.40 (s, Cp*II). **Elemental Analysis** calculated for C_47_H_54_Cl_2_Ir_2_N_4_O_2_·(H_2_O) (1180.30 g/mol): C 47.83, H 4.78, N 4.75; found: C 47.9, H 4.73, N 4.63. **MS (ESI+, DCM)**: *m*/*z* calculated for C_37_H_40_IrN_4_O_2_ [M-Cp*IrCl_2_]^+^: 765.2775, found: 765.2740.

### 4.3 Experiments and Methods

#### 4.3.1 X-ray Crystallographic Structure Determination

Suitable single crystals for X-ray diffraction studies of complexes **C1 × 0.4CH**_**2**_ **Cl**_**2**_, **C3 × 0.5CH**_**2**_ **Cl**_**2**_, **C4 × 0.7C**_**2**_ **H**_**4**_ **Cl**_**2**_, and **C5 × 0.5H**_**2**_ **Ox0.25C**_**3**_ **H**_**6**_ **O** were obtained by the following methods: slow diffusion of pentane into a solution of the complex in DCM (**C1**, **C3**, and **C5**), and slow diffusion of diethyl ether into a solution of the complex in DCE (**C4**). Single crystals were transferred to a Bruker APEX II CCD-based diffractometer equipped with a graphite-monochromated Mo Kα radiation source (λ = 0.71073 Å). The data sets were integrated with Saint^72^ and corrected for Lorentzian and polarization effects. A semiempirical absorption correction was applied to the diffraction data.^73^ The software package OLEX2 ^74,75^ was used for space group determination, structure solution, and refinement by full-matrix least-squares methods based on F^2^. A successful solution by direct methods provided most non-hydrogen atoms from the E map. The remaining non-hydrogen atoms were located in an alternating series of least-squares cycles and difference Fourier maps. The non-hydrogen atoms were refined with anisotropic displacement coefficients and hydrogen atoms were placed by using a riding model and included in the refinement at calculated positions. Deposition numbers in the Cambridge database are 2534831 (**C1**), 2534832 (**C3**), 2534833 (**C4**), and 2534834 (**C5**).

#### 4.3.2 Stability Studies in DMSO-*d*_6_ and DMSO-*d*_6_:D_2_O (8:2) (NMR Spectroscopy)

Solutions of all complexes at 2 mM in DMSO-*d*_6_ and DMSO-*d*_6_:D_2_O (8:2) were studied by ^1^H NMR spectroscopy over time at room temperature to evaluate their stability.

#### 4.3.3 Stability Studies in Water (UV–Vis Spectroscopy)

The stability of complex **C1** at 5.0 × 10^–5^ M in water (4% DMSO) was monitored by UV–vis spectroscopy over time at room temperature.

#### 4.3.4 Reactivity of **C3w** with *n*-Bu_4_NCl

A solution of **C3w** (2.0 mM) with 150 equiv of *n*-Bu_4_NCl in a DMSO-*d*_6_:D_2_O (8:2) mixture was monitored by ^1^H NMR spectroscopy at room temperature.

#### 4.3.5 p*K*_a_ Determination

Solutions of complexes (5 × 10^–5^ M) in aqueous solution (4% DMSO) with *n*-Bu_4_NBF_4_ (5 mM) were prepared. The presence of the salt ensured the conductivity of the media. Evolution of the peaks were studied at different pH by recording the absorbance spectrum. Aliquots of 10 μL of HCl (0.002–0.020 M) or NaOH (0.002–0.020 M) were added to a solution of 2.4 mL of complex to vary the pH. The absorbance versus pH plot enables to determine one constant according to equation from the Wilson and Lester method (eq 1)^76^

1 $$\log K=\log\frac{A_{m}-A_{a}}{A_{b}-A_{m}}+\mathrm{pH}$$

where *A*_a_ is the absorbance for the acid species, *A*_b_ is the absorbance for the basic species and *A*_m_ is the absorbance of the mixture. The intersection obtained by representing log­(*A*_m_ – *A*_a_)/(*A*_b_ – *A*_m_) versus pH provides the p*K*_a_.

#### 4.3.6 Photophysical Properties

For photophysical measurements, aqueous solutions (4% DMSO) of the complexes were prepared. All solvents used were spectroscopic grade. UV–vis absorption spectra were recorded on a Secomam Uvikon XS spectrophotometer at room temperature using the LabPower Junior program. Quartz cuvettes with 1 cm optical path length were used for the measurements. Molar absorption coefficients (ε) were determined by direct application of the Beer–Lambert law, using solutions of the compounds with concentrations ranging from 1.0 × 10^–6^ to 1.0 × 10^–5^ M. Photoluminescence excitation and emission spectra of solutions at 5.0 × 10^–5^ M were recorded on a PTI Quanta Master TM spectrofluorometer from Photon Technology International (PTI) equipped with a Xenon short arc lamp (75 W) and an 814PTM detector. Hellma quartz cuvettes with 1 cm optical path length were used. Felix32 software was used to collect and process luminescence data.

#### 4.3.7 Lipophilicity

The partition coefficients of Ir­(III) complexes were determined by standard “shake flask” method as previously described.^37^ Octanol-saturated water (OSW) and water-saturated octanol (WSO) were prepared using analytical grade 1-octanol and ultrapure water. Then, 2 mL of 25 μg/mL of complex in WSO were shaken with 2 mL of OSW at 1500 rpm for 6 h at room temperature. The organic phases were collected by centrifugation (4000 rpm, 10 min, r.t.). The absorbance of WSO phase before and after shaking was measured. The distribution coefficients (log *D*_o/w_) of the complexes were obtained from the ratio of the complexes present in 1-octanol before and after shaking (eq 2). Each experiment was repeated three times.

2 $$\log P=\log\frac{C_{1‐\mathrm{oct}}}{C_{\mathrm{water}}}=\log\frac{C_{1‐\mathrm{oct}}}{C_{\mathrm{total}}-C_{1‐\mathrm{oct}}}=\log\frac{A_{1‐\mathrm{oct}}}{A_{\mathrm{total}}-A_{1‐\mathrm{oct}}}$$

where *A*_1‑oct_ corresponds to the absorbance of WSO phase and *A*_total_ the actual concentration of the WSO before shaking.

#### 4.3.8 NADH Oxidation (UV–Vis Spectroscopy)

Fresh stock solutions of complexes at 7.5 mM in water (0.3% DMSO) and NADH at 300 mM in water were prepared. In a quartz cell, 1 mL of complex solution, 1 mL of NADH solution and 1 mL of water were mixed to prepare a final solution with 2.5 μM of complex and 100 μM of NADH (complex/NADH rate = 1/40) in aqueous solution (0.1% DMSO). The solutions were studied by recording UV–vis absorption spectra at room temperature for 24 h. The stability of the NADH at 100 μM was also confirmed.

The catalytic rate of the complexes was evaluated by monitoring its absorbance decrease at 340 nm, which corresponds to NADH oxidation. The turnover numbers (TONs) of complexes were calculated by measuring the absorption difference at 340 nm after 24 h of reaction. TON was calculated from the difference in NADH concentration after 24 h divided by the concentration of catalyst (eq 3), where b is the optical path length (1 cm). The concentration of NADH was obtained using the extinction coefficient ε_340_ = 5751 M^–1^ cm^–1^. The experiment was performed in triplicate.

3 $$\mathrm{TON}=\frac{\left[\mathrm{NADH consumed in}\;24\;\mathrm{hours}\right]}{\left[\mathrm{Catalyst}\right]}=\frac{\frac{A_{0h}-A_{24h}}{b\cdot \varepsilon_{340\mathrm{nm}}}}{\left[\mathrm{Catalyst}\right]}$$

#### 4.3.9 Oxidation of NADH (^1^H NMR Spectroscopy)

A solution of complex **C2w** (2 mM) and NADH (5 equiv) in DMSO-*d*_6_:H_2_O (1:1) was monitored by ^1^H NMR spectroscopy at room temperature over time.

#### 4.3.10 Depletion of GSH

A calibration curve to quantify free l-glutathione (GSH) was performed by using the Ellman’s reagent (5,5-dithiobis­(2-nitrobenzoic acid), DTNB). For this purpose, a solution of DTNB (60 μM) in Tris-HCl buffer (pH 7.2) was monitored by UV–vis spectroscopy upon addition of increasing concentrations of GSH (0–60 μM). The absorbance at 412 nm, corresponding to the formation of TNB, was used to obtain the calibration curve. Subsequently, solutions of the Ir­(III) complexes (10 μM) and GSH (50 μM) were kept in the dark for 4 h. After the indicated times, DTNB (60 μM) was added to the solutions, and the UV–vis spectra were recorded. The absorbance at 412 nm was used to determine the concentration of free GSH remaining in solution. The amount of depleted GSH was calculated by subtracting the concentration of free GSH from the initial GSH concentration.

#### 4.3.11 Interaction with BSA

A 2 μM solution of bovine serum albumin (BSA) was prepared in Tris-HCl buffer. The Ir­(III) complexes were added from a 10 mM DMSO stock solution to obtain final concentrations ranging from 0 to 60 μM. The resulting mixtures were stirred for 5 min at 80 rpm prior to measurement. Emission spectra were recorded on a PTI Quanta Master spectrofluorometer (Photon Technology International) equipped with a 75 W xenon short-arc lamp and an 814PTM detector, using Hellma quartz cuvettes with a 1 cm optical path length. Data were acquired with Felix32 software. The excitation wavelength was fixed at 285 nm, and emission spectra were collected in the 300–450 nm range. The excitation and emission monochromator slits were set to 0.75 mm, corresponding to a spectral bandwidth of 3 nm.

The interaction between BSA and the complexes was evaluated using the Stern–Volmer eq (eq 4) to assess dynamic quenching, the Lineweaver–Burk eq (eq 5) to characterize static quenching, and the Scatchard eq (eq 6) to determine binding parameters.

4 $$\frac{I_{0}}{I}=1+K_{\mathrm{SV}}\left[Q\right]$$

where *I*_0_ is the emission intensity of pure BSA, *I* the emission intensity of BSA in the presence of the complex at any given concentration, *K*_SV_ the Stern–Volmer quenching constant, and [*Q*] the concentration of the quencher.

5 $$\frac{1}{I_{0}-I}=\frac{1}{I_{0}}+\frac{1}{K_{\mathrm{LB}}\cdot I_{0}\cdot \left[Q\right]}$$

where *I*_0_, *I* and [*Q*] are defined as above and *K*_LB_ is the static quenching constant.

6 $$\log\left(\frac{I_{0}-I}{I}\right)=\log K_{b}+n\log\left[Q\right]$$

where *K*_b_ is the binding constant and *n* is the number of sites.

#### 4.3.12 Molecular Docking

The binding modes of complexes **C1w**, **C3w** and **C5w** with BSA were investigated through molecular docking calculations using Autodock 4.2.^77^ The geometries of the complexes were optimized in Gaussian 09 ^78^ employing the DFT method with the B3LYP functional.^79,80^ The 6–31G­(d,p) basis set was used for C, N, and H atoms, while Ir atoms were described using the SDD effective core potential (ECP) basis set. The effects of water as solvent were taken into account through the self-consistent reaction field (SCRF) approach employing the SMD solvation model.^81^ Atomic charges of the optimized geometries were obtained using the electrostatic potential fitted (CHELPG) method^82^ at the same level of theory. The CHELPG scheme was selected because it provides a reliable representation of the molecular electrostatic potential around transition metal complexes while remaining numerically stable for systems including ECP.^83^ The resulting charges were incorporated into the docking input file of the complexes to achieve an accurate description of the electrostatic interactions between the complexes and the protein. The crystal structure of BSA (PDB ID 4F5S) was obtained from the Research Collaboratory for Structural Bioinformatics Protein Data Bank (RCSB PDB).^84^ The AutoDockTools 1.5.7 program, provided by MGLTools software suite,^77^ was used to prepare the BSA structure for the docking studies. All crystallographic water molecules were removed, polar hydrogens were added, and nonpolar hydrogens were merged. Finally, Gasteiger charges were assigned. Two independent docking simulations were performed for each complex to evaluate which binding site (around TRP134 or TRP213) was energetically preferred. The grid size was set to 80 × 80 × 80, with a spacing of 0.375 Å. The grid center *xyz*-coordinates were 19.639, 35.691, and 90.174 for TRP134 and −4.896, 23.661, and 106.964 for TRP213. Docking calculations were carried out with Lamarckian Genetic Algorithm (LGA). The maximum number of energy evaluations was set to 25,000,000 and for each of the 10 independent runs, a maximum number of 27,000 generations was performed on a population size of 150 individuals. The lowest-energy docked conformation, according to the Autodock predicted binding energy, was selected as the most favorable binding mode of each complex. To assess the reliability of the docking protocol, a redocking study was performed using the cocrystallized triethylene glycol (TEG) molecule present in the BSA structure. The ligand was extracted from the crystal structure and prepared following the same protocol used for the studied complexes, including the assignment of CHELPG charges while preserving the experimental geometry. Docking calculations were carried out under the same conditions described above. The agreement between the docked and crystallographic poses, evaluated over the heavy atoms of the ligand, yielded an RMSD value of 2.56 Å, consistent with a reasonable reproduction of the experimental binding mode for a flexible ligand.^85^ The analysis of the interactions between the complexes and the protein was carried out using Discovery Studio 2025 software.

#### 4.3.13 Effect of Cell Viability

Cells were seeded in 96-well plates at densities of 3000 cells per well for A549 and HeLa cells and 5000 cells per well for BxPC-3 cells and MRC-5 fibroblats, and allowed to attach for 24 h. Complexes were dissolved in DMSO to a concentration of 5 mM, which was then diluted with sterile water to obtain a 1 mM stock solution. Subsequently, the stock solutions were diluted in culture medium to prepare working solutions ranging from 100 to 1 μM. The maxim percentage of DMSO in the working solutions was 2%. Nontreated cells were used as negative control. Cisplatin (1 mg/mL; Accord Healthcare) was used as a positive control. Cells were treated with the different solutions of the compounds for a total of 48 h. For photoactivation experiments, cells were incubated with the compounds for 4 h, to allow cellular internalization, followed by 1 h irradiation with blue light (460 nm) using a light-emitting diode (LED) system (LuxLight), providing a total light dose of 24.1 J cm^–2^. Following the treatment, cells were washed with phosphate buffer saline (PBS) and the cell viability was assessed using an MTT assay. Briefly, fresh culture medium containing 10% MTT (0.5 mg/mL, Sigma-Aldrich) was added to each well. Following a 2 h incubation at 37 °C, the medium was removed, and the precipitates were dissolved in DMSO. The absorbance of each well was measured at 570 nm in a Multiscan Plate Reader (Synergy 4, Biotek, Winooski, USA) and the concentration that reduces cell viability by 50% (IC_50_) was calculated with the Gen5 Data Analysis Software (BioTeck). In photoactivation experiments, the phototoxic indexes (PIs) of the complexes were established by dividing the IC_50_ value obtained in dark (IC_50,dark_) by the value obtained in photoactivation conditions (IC_50,light_). All experiments were performed at least three times with triplicates per each condition.

#### 4.3.14 Intracellular ROS Generation

A549 cells were seeded on 12-well plates at a density of 100,000 cells/well. 24h later, cells were treated with the complexes at 25 μM for 4h. In parallel, cells were incubated with culture medium alone as a negative control, or treated with 20 μM FeCl_2_ in the presence of 1.75% H_2_O_2_ for ROS generation, as positive control. All treatments were carried out in the dark at 37 °C. After washing with PBS, intracellular ROS were detected using the dichlorodihydrofluorescein diacetate (H_2_DCFDA) probe (Sigma-Aldrich). Cells were immediately collected by trypsinization, and the intracellular fluorescence was measured by flow cytometry using a Novocyte flow cytometer (Agilent Technologies) equipped with the NovoExpress software. For each treatment, the fluorescence intensity of 10,000 cells was analyzed.

#### 4.3.15 Microscopy Experiments

For confocal microscopy experiments, A549 cells were seeded onto glass-bottomed 8 well chamber slides (Ibidi) at a density of 50,000 cells per well and allowed to attach overnight. Cells were then treated with the complexes at their respective IC_50_ for 24 h at 37 °C and washed with PBS before staining. For mitochondria visualization, cells were incubated with the fluorescent dye MitoTracker Red CMXRos (Molecular Probes) at 200 nM in serum free culture medium for 15 min at 37 °C. Cell nuclei were counterstained in blue with Hoechst 33342 (Invitrogen) diluted 1:4000 in DMEM without phenol red. To analyze lysosomal damage, cells were incubated with AO at 5 μM at 37 °C for 15 min. After washing with PBS, cell images were immediately obtained by a Nikon A1R confocal microscope and images were analyzed using the NIS-Elements AR (Nikon, Japan) and the Fiji/ImageJ softwares.

## Supplementary Material



## Data Availability

The data underlying this study are available in the published article and its Supporting Information.

## Notes

1H NMR spectra of characterization and stability studies; X-ray diffraction data; UV–vis absorption and emission spectra; interaction with biomolecules assays; molecular docking; and MTT data assays under light (PDF)
