# Supplementary material for: Substituent-Controlled Double Orthometalation and Anticancer Activity in Half-Sandwich Ir(III) Complexes Bearing C∧N Ligands
Source: ACS Org Inorg Au. 2026 Jul 8;6(5):580–98. doi: 10.1021/acsorginorgau.6c00026 (PMC13650956; doi:10.1021/acsorginorgau.6c00026)
Supplement: Supplementary file 1 [file gg6c00026_si_001.pdf]

Substituent-Controlled Double Orthometalation and Anticancer Activity in Half-Sandwich Ir(III) Complexes Bearing C^N Ligands.

Mario Sánchez-Migallón,[1,2] Carlos Gonzalo-Navarro,*[1,2] Ana María Rodríguez,[2,3] Cristina Bermejo-Casadesús,[4] Blanca R. Manzano,[1,2] Anna Massaguer,*[4] Gema Durá*[1,2]

[1] Universidad de Castilla-La Mancha, Departamento de Química Inorgánica, Orgánica y Bioquímica, Facultad de Ciencias y Tecnologías Químicas, Avenida Camilo José Cela 10, 13071 Ciudad Real, Spain.

[2] Instituto Regional de Investigación Científica Aplicada (IRICA), Universidad de Castilla-La Mancha, Avenida Camilo José Cela 1, 13071 Ciudad Real, Spain.

[3] Universidad de Castilla-La Mancha, Departamento de Química Inorgánica, Orgánica y Bioquímica, Escuela Técnica Superior de Ingenieros Industriales, Avenida Camilo José Cela 3, 13071 Ciudad Real, Spain.

[4] Universitat de Girona, Departament de Biologia, Facultat de Ciències, Maria Aurelia Capmany 40, 17003 Girona, Spain.

Chart S1. Complexes presented in this work.

Complex	Ir	R^1	R^2	Z
**C1**	Mononuclear	H	OMe	Cl
**C2**	Mononuclear	H	CN	Cl
**C3**	Mononuclear	CH_2CH_2-morpholine	OMe	Cl
**C4**	Mononuclear	CH_2CH_2-morpholine	CN	$OCOCF_3$
**C5**	Binuclear	CH_2CH_2-morpholine	OMe	Cl

1. NMR Characterization

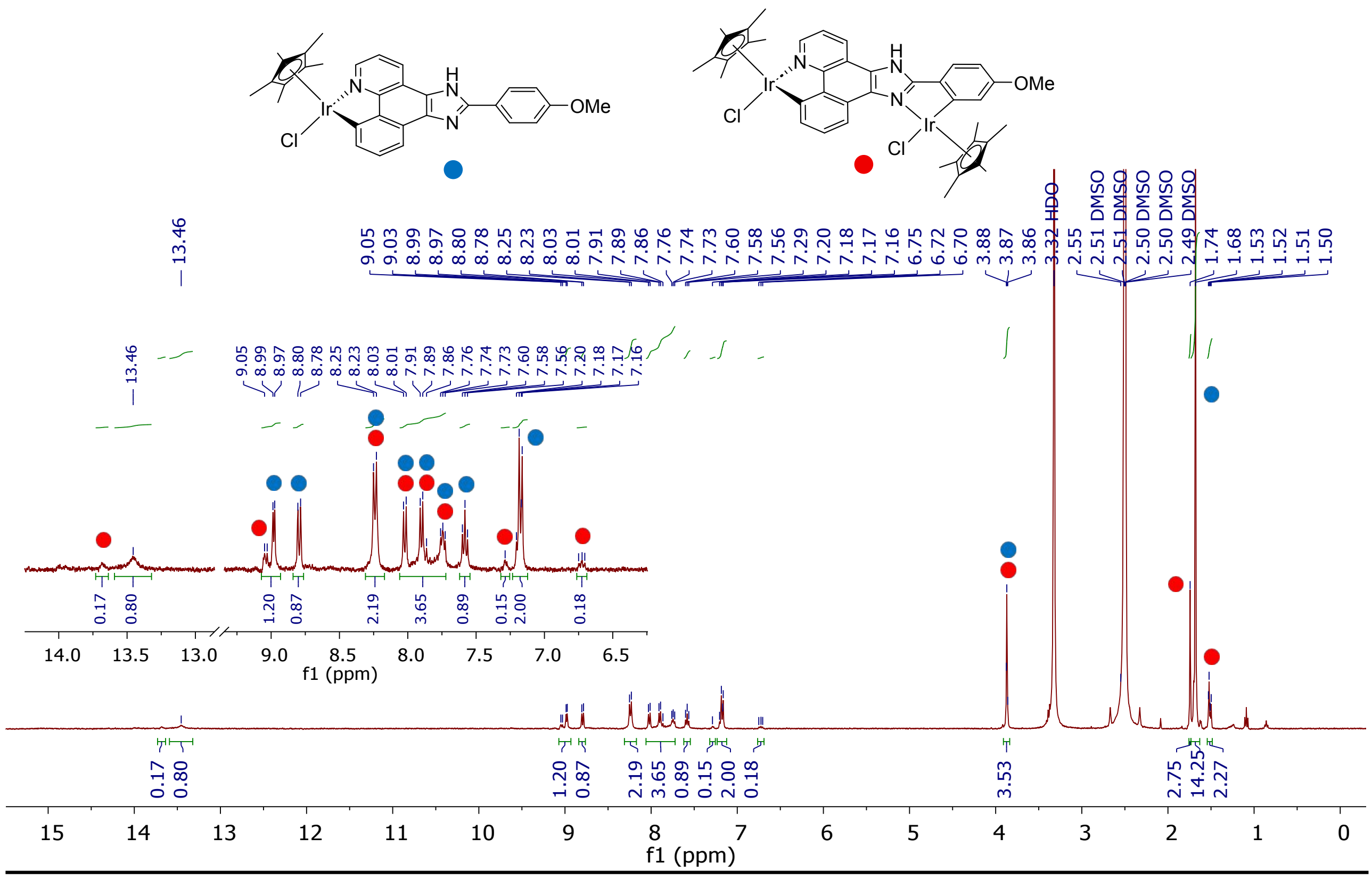

Figure S1. ^{1}H NMR (500 MHz, DMSO-d_6, 298 K) of the reaction crude of **C1** synthesis. Two compounds are obtained: **C1** (blue circles) and complex $[(Cp^*IrCl)_2(\mathbf{L1})]$ (red circles).

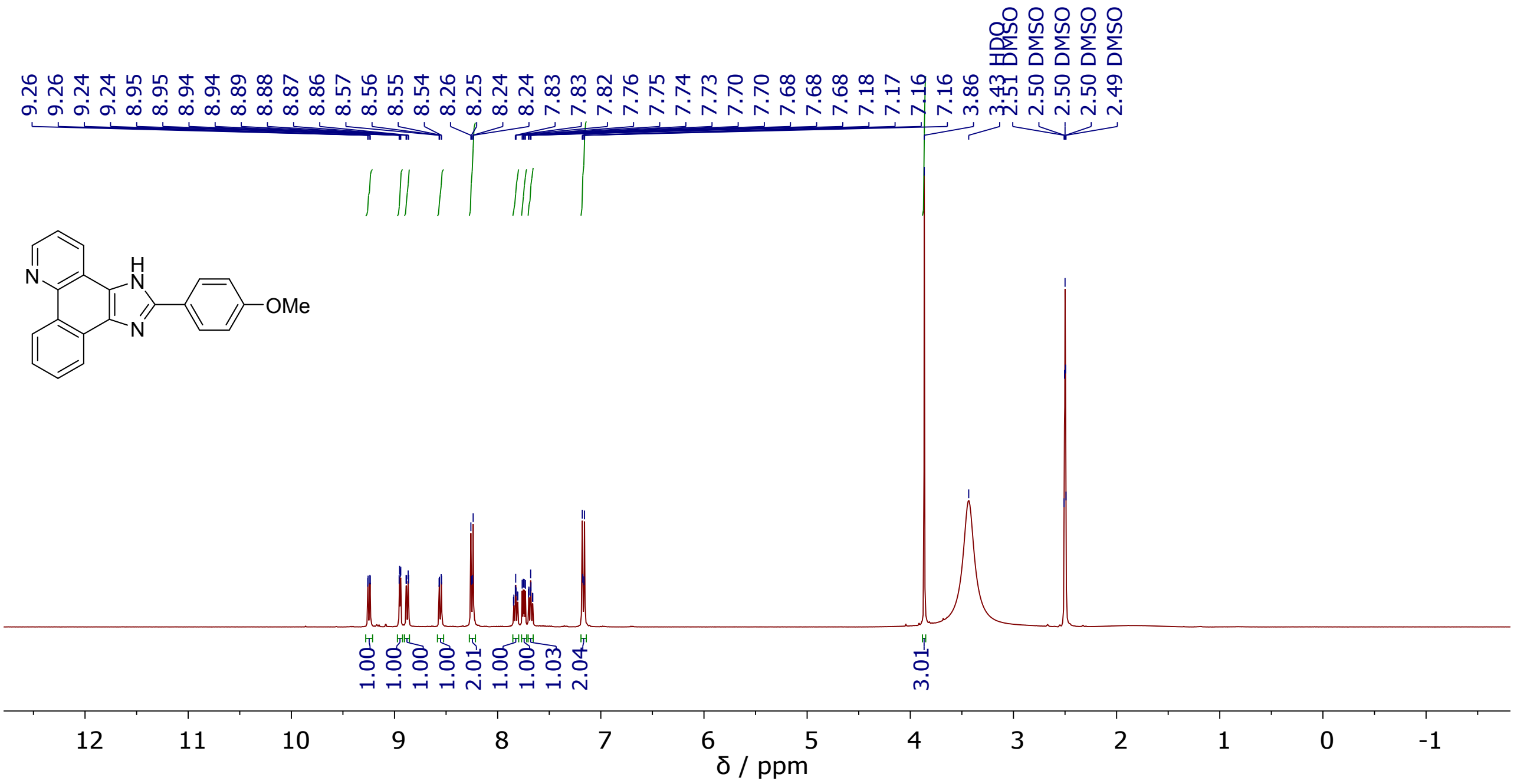

Figure S2. ^{1}H NMR (400 MHz, DMSO-d_6, 298 K) spectrum of **H-L1**.

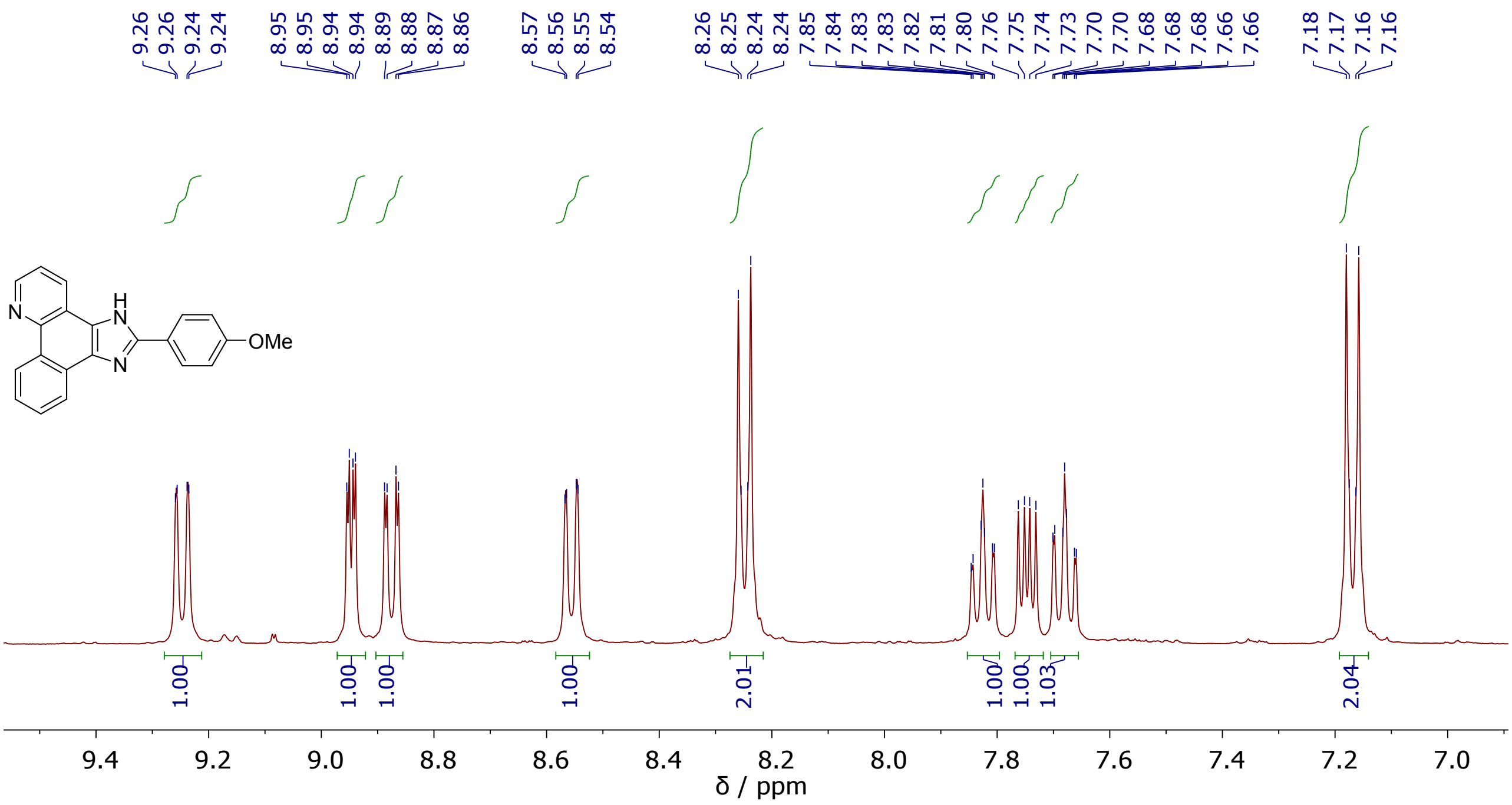

Figure S3. ^{1}H NMR (400 MHz, DMSO-d_6, 298 K) spectrum of **H-L1** in the aromatic region.

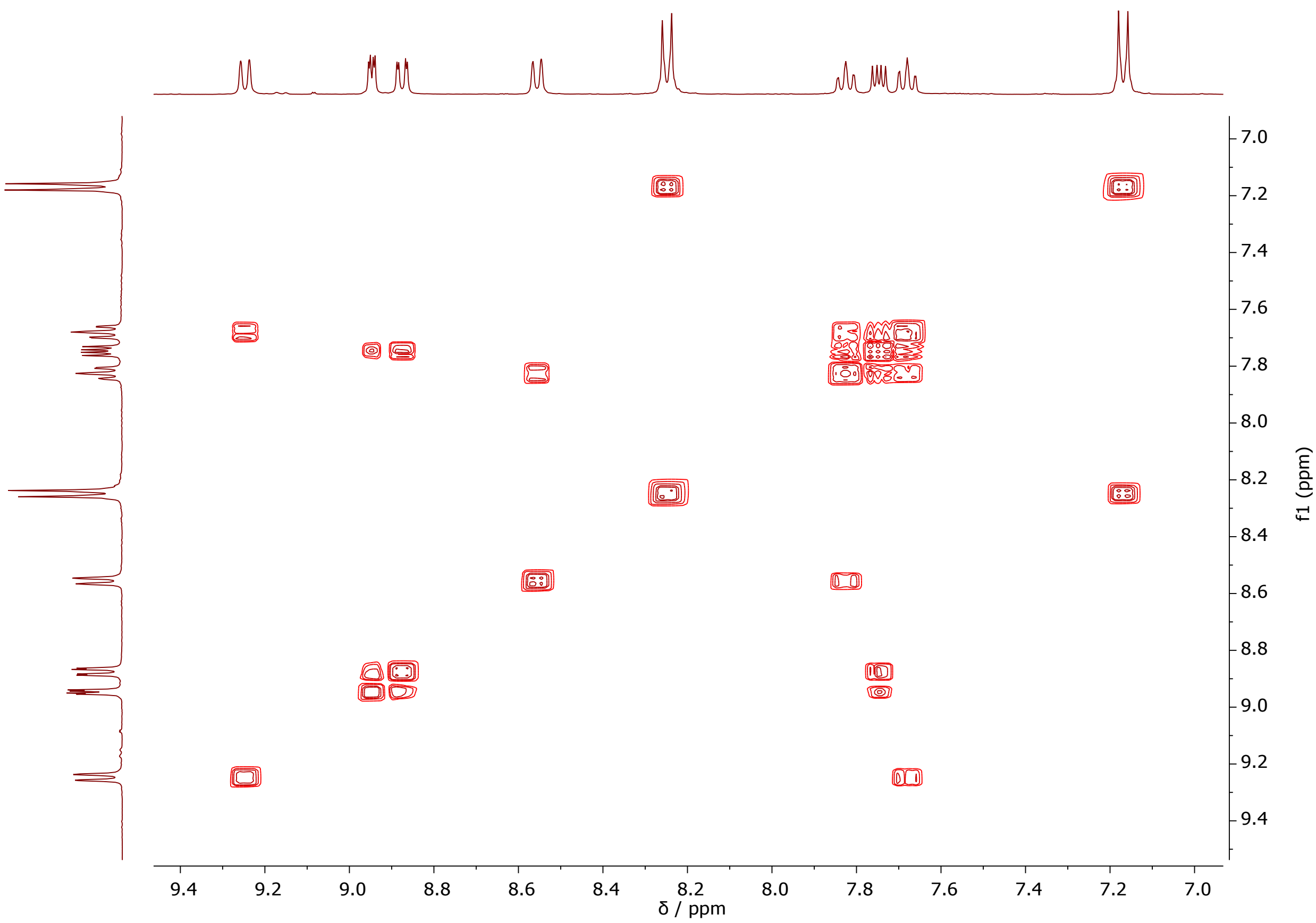

Figure S4. ^{1}H-^{1}H COSY (400 MHz, DMSO-d_6, 298 K) spectrum of **H-L1**.

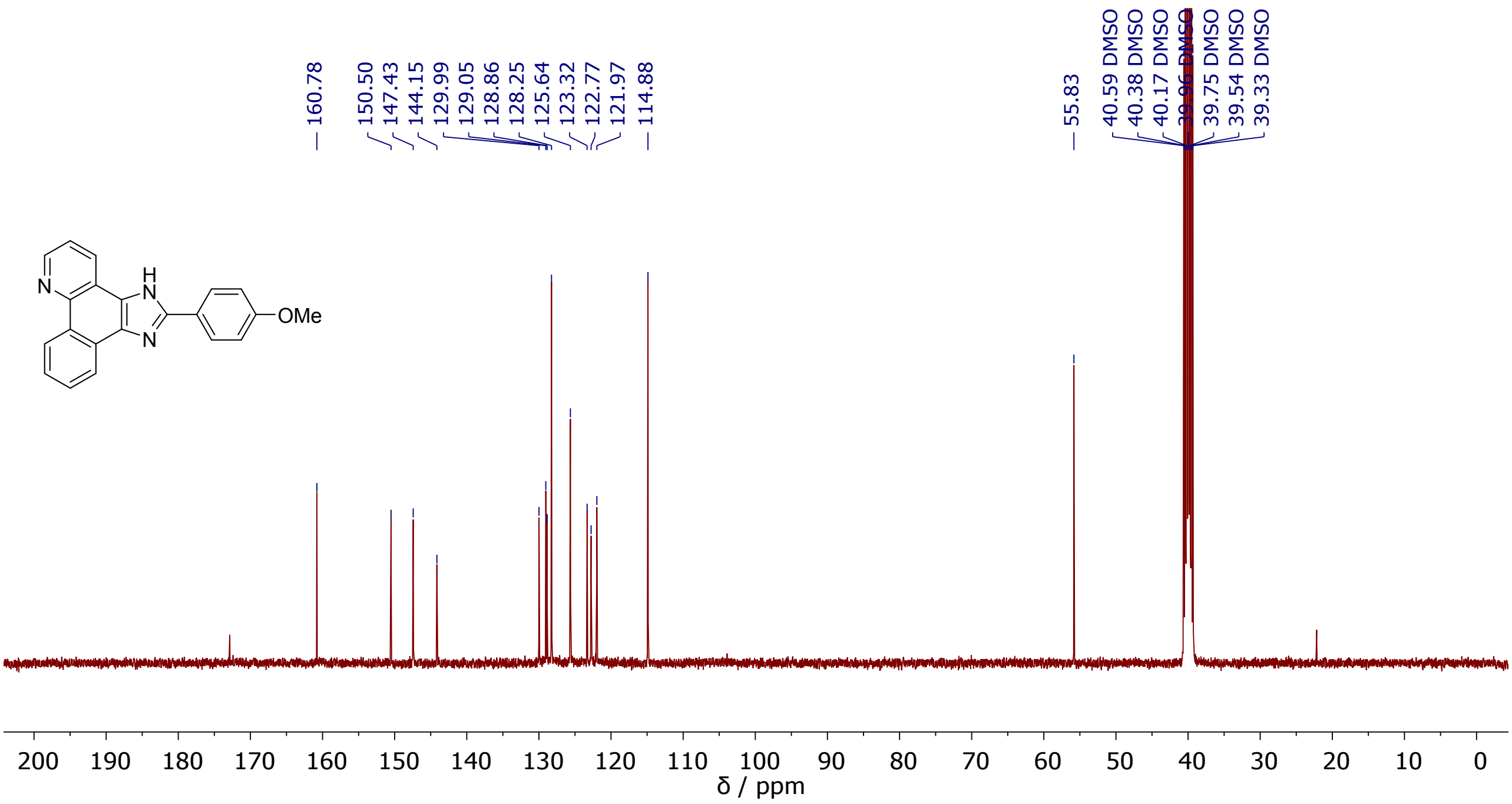

Figure S5. $^{13}C\{^{1}H\}$ NMR (101 MHz, DMSO-d_6, 298 K) spectrum of **H-L1**.

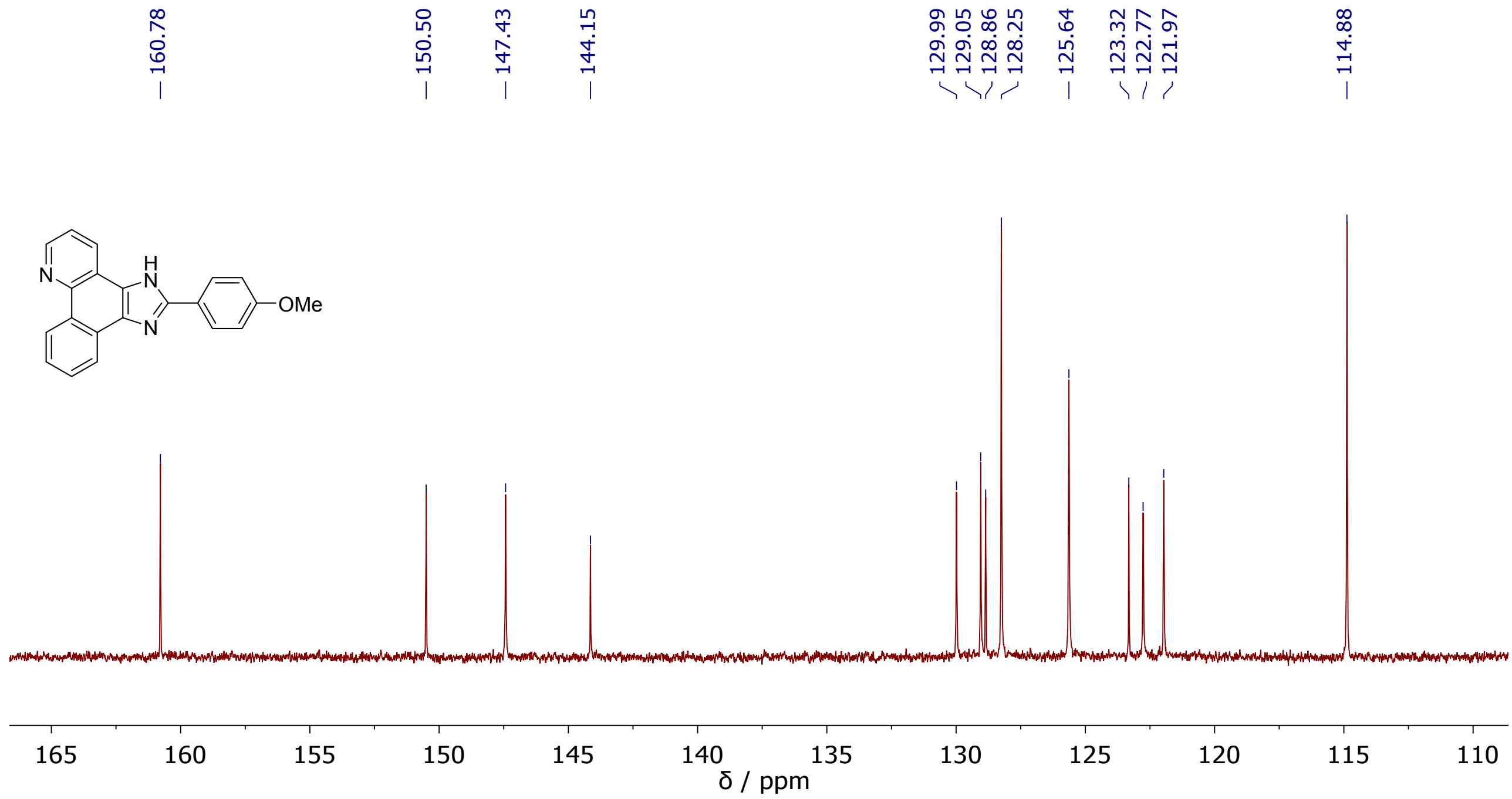

Figure S6. $^{13}C\{^{1}H\}$ NMR (101 MHz, DMSO-d_6, 298 K) spectrum of **H-L1** in the aromatic region.

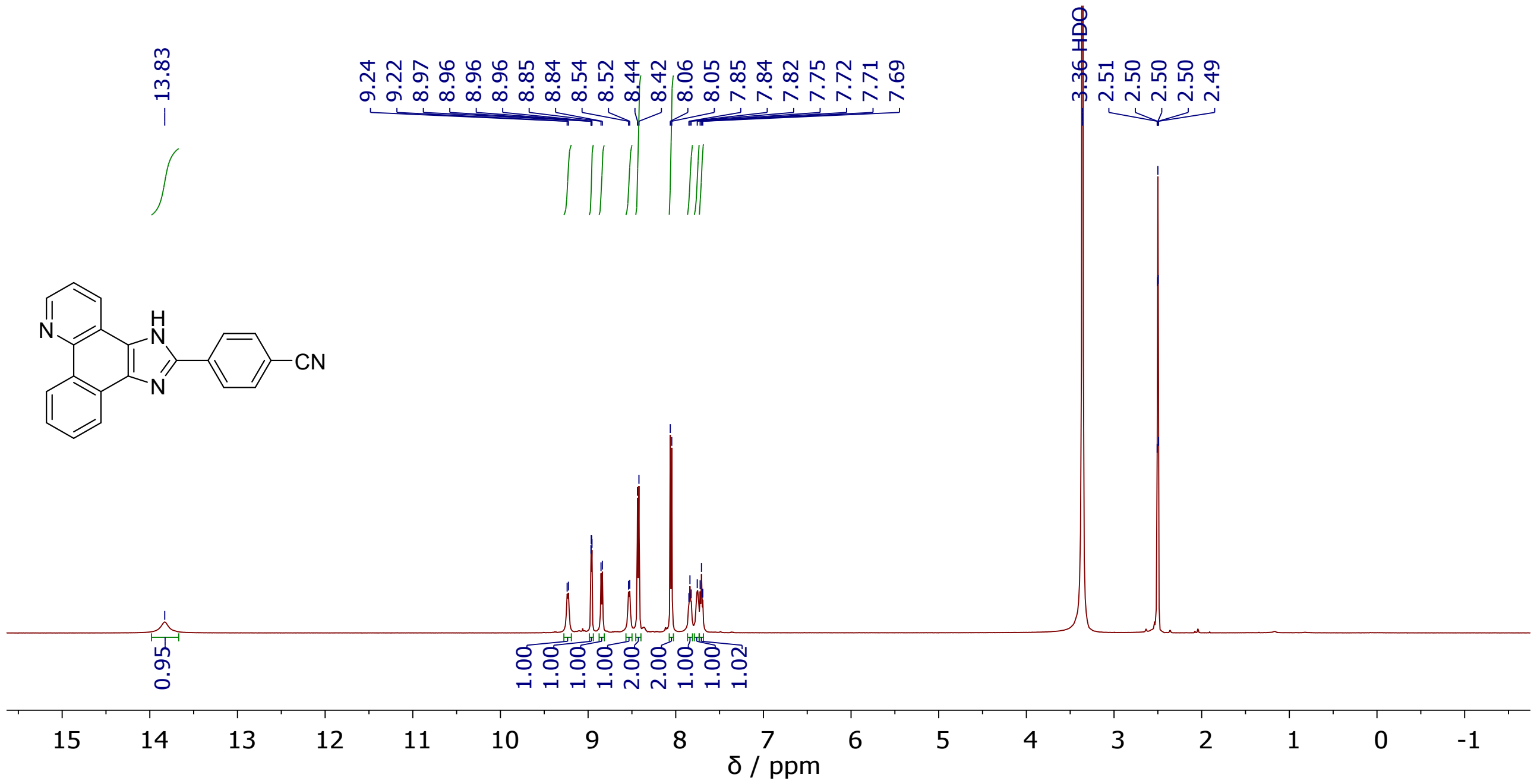

Figure S7. ^{1}H NMR (500 MHz, DMSO-d_6, 298 K) spectrum of **H-L2**.

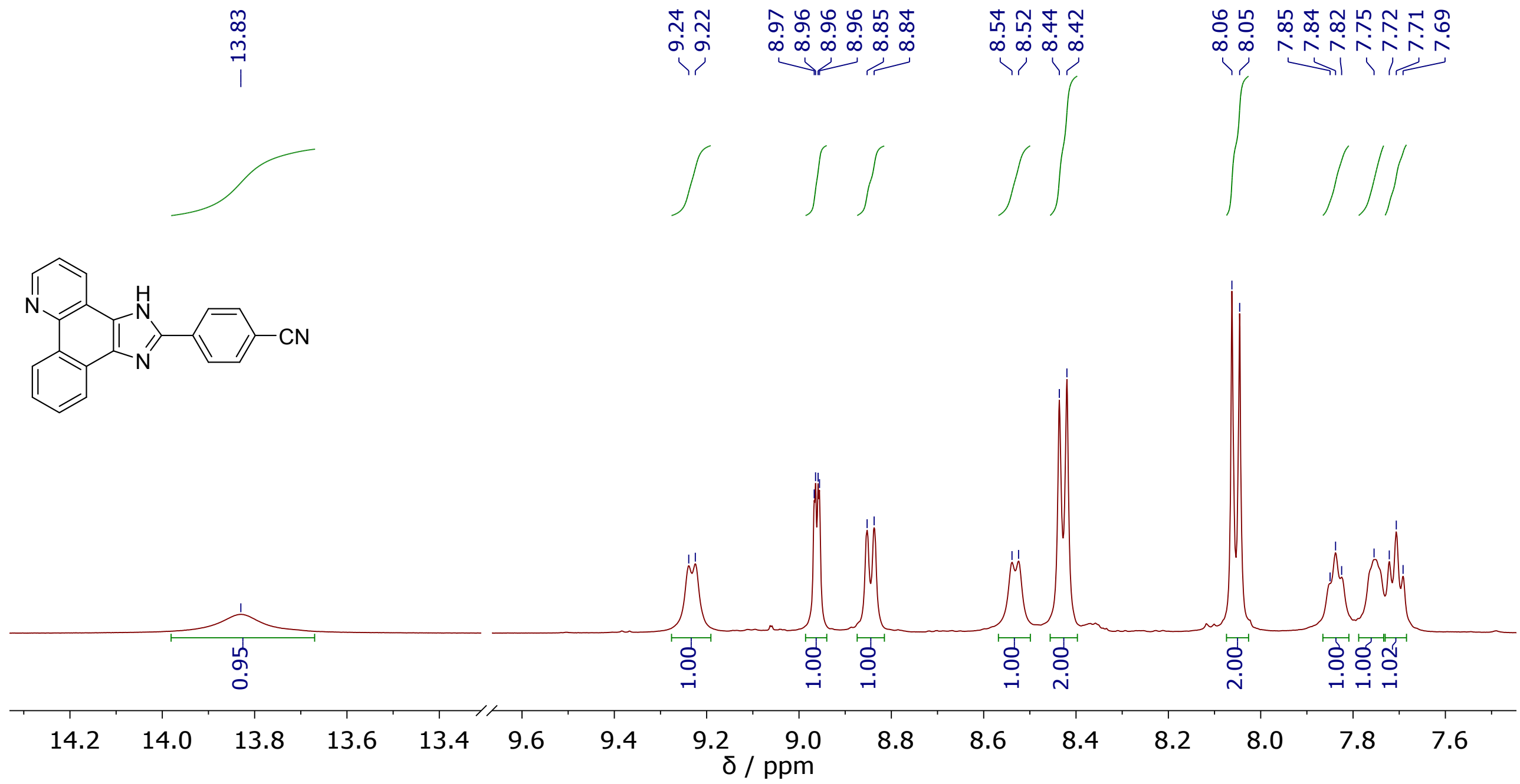

Figure S8. ^{1}H NMR (500 MHz, DMSO-d_6, 298 K) spectrum of **H-L2** in the aromatic region.

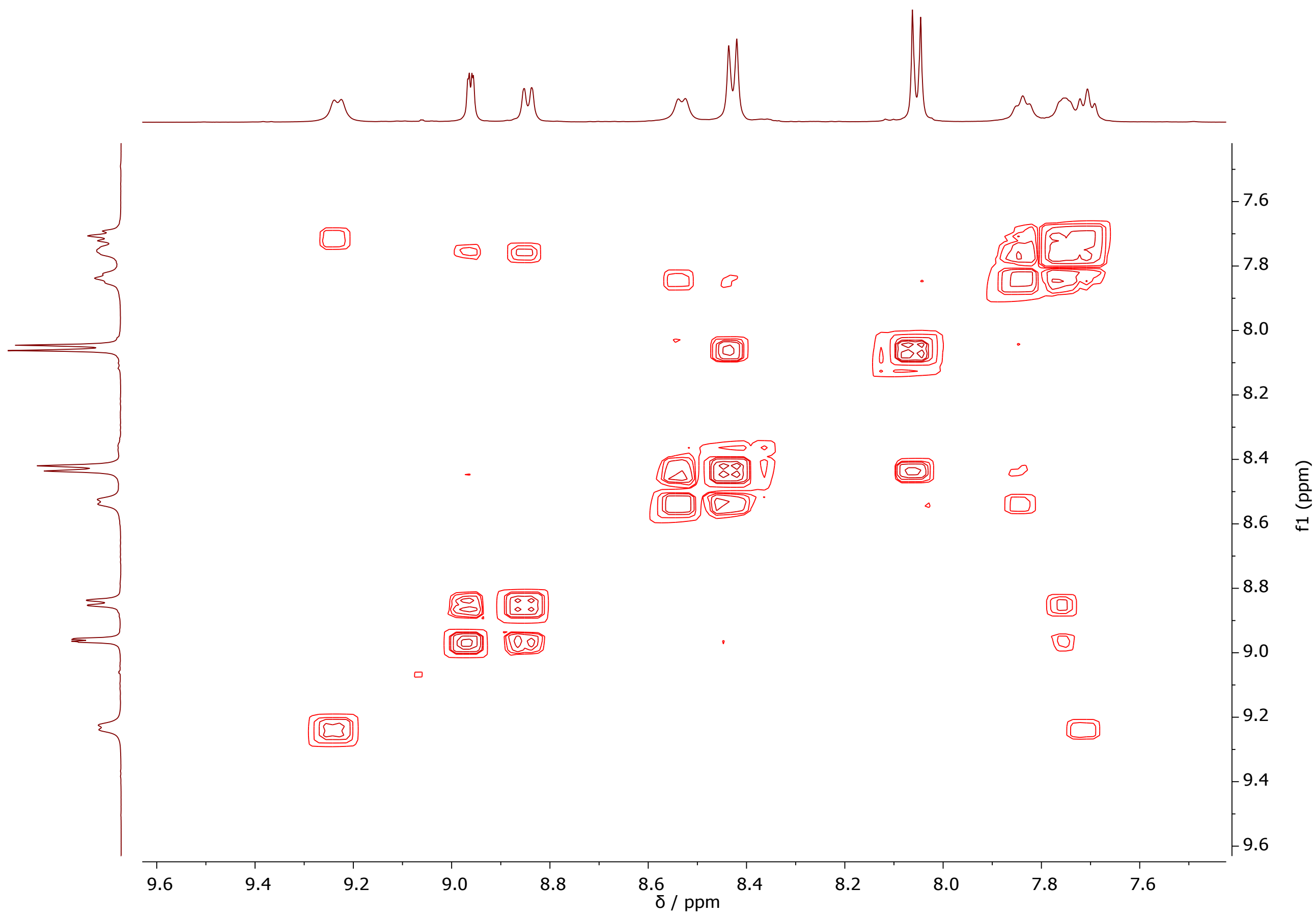

Figure S9. ^{1}H-^{1}H COSY (500 MHz, DMSO-d_6, 298 K) spectrum of **H-L2**.

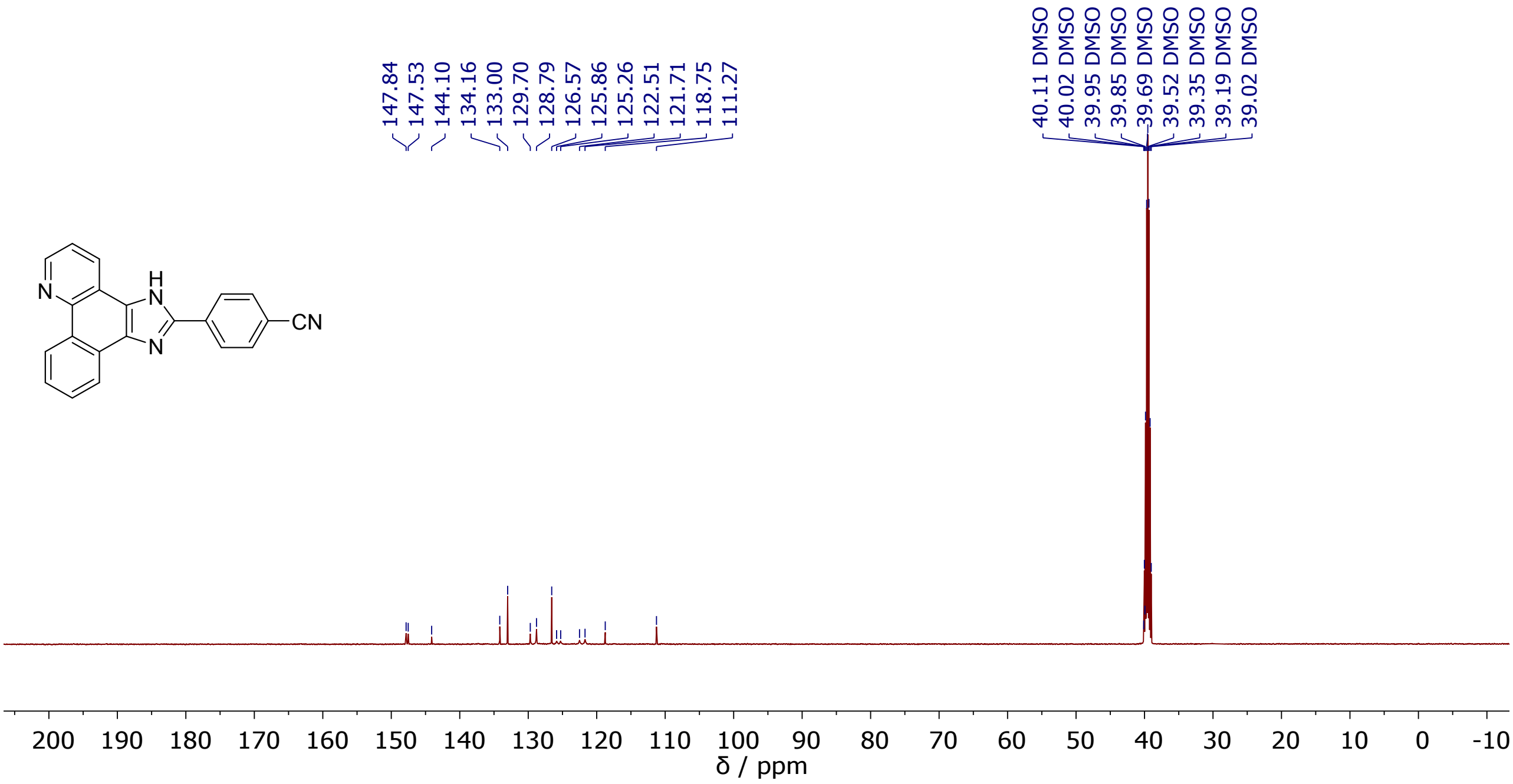

Figure S10. $^{13}C\{^{1}H\}$ NMR (126 MHz, DMSO-d_6, 298 K) spectrum of **H-L2**.

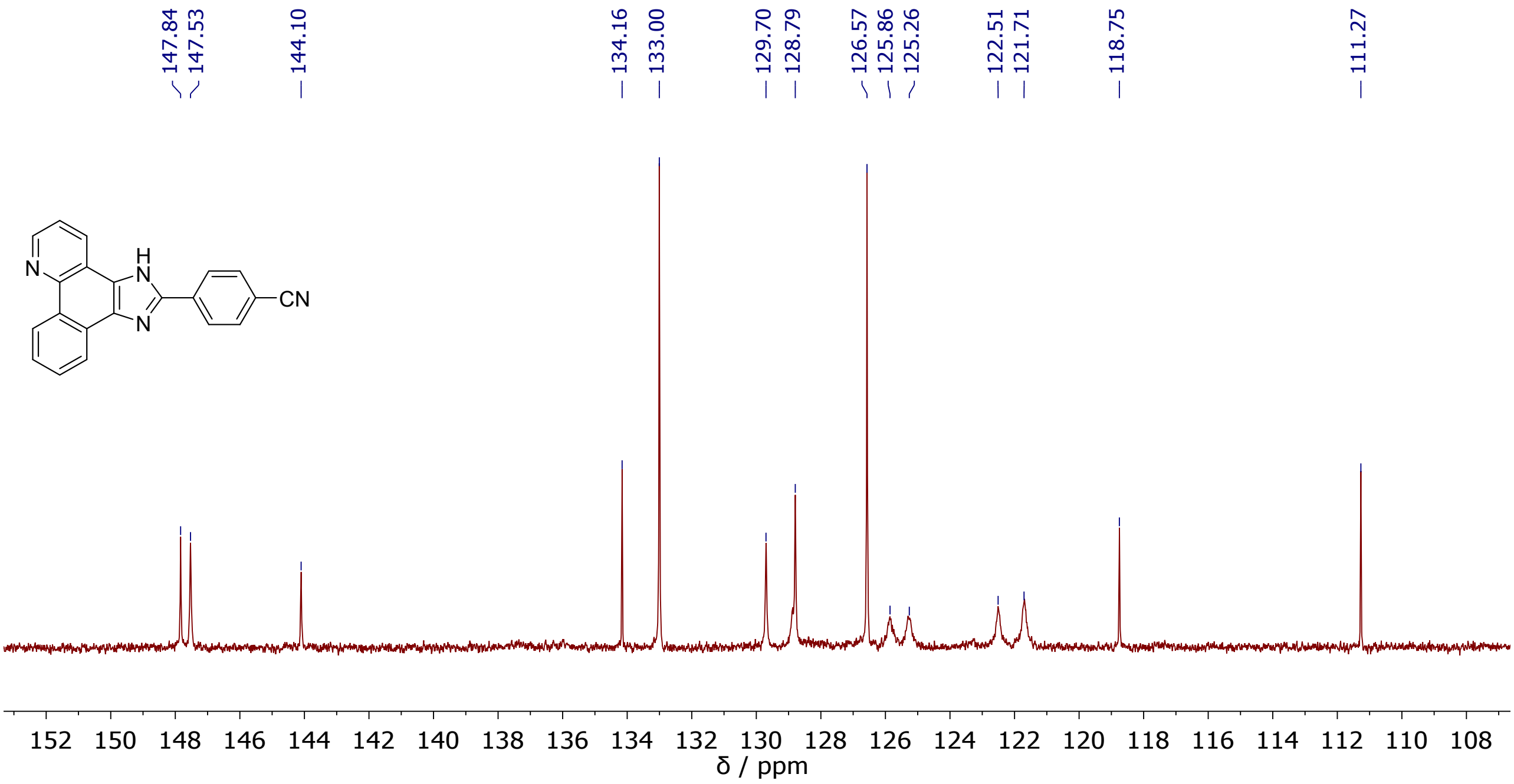

Figure S11. $^{13}C\{^{1}H\}$ NMR (126 MHz, DMSO-d_6, 298 K) spectrum of **H-L2** in the aromatic region.

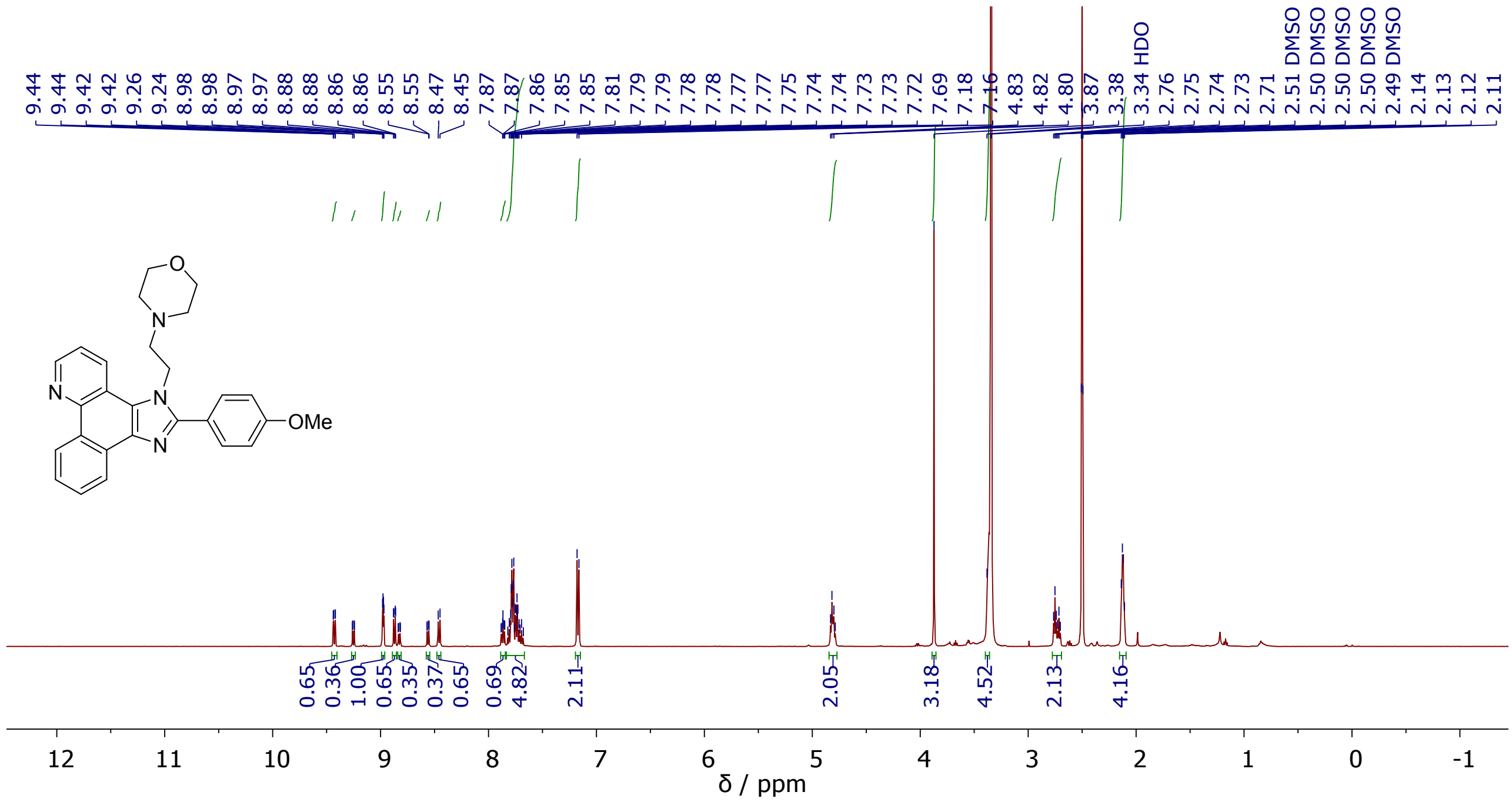

Figure S12. ^{1}H NMR (500 MHz, DMSO-d_6, 298 K) spectrum of **H-L3**.

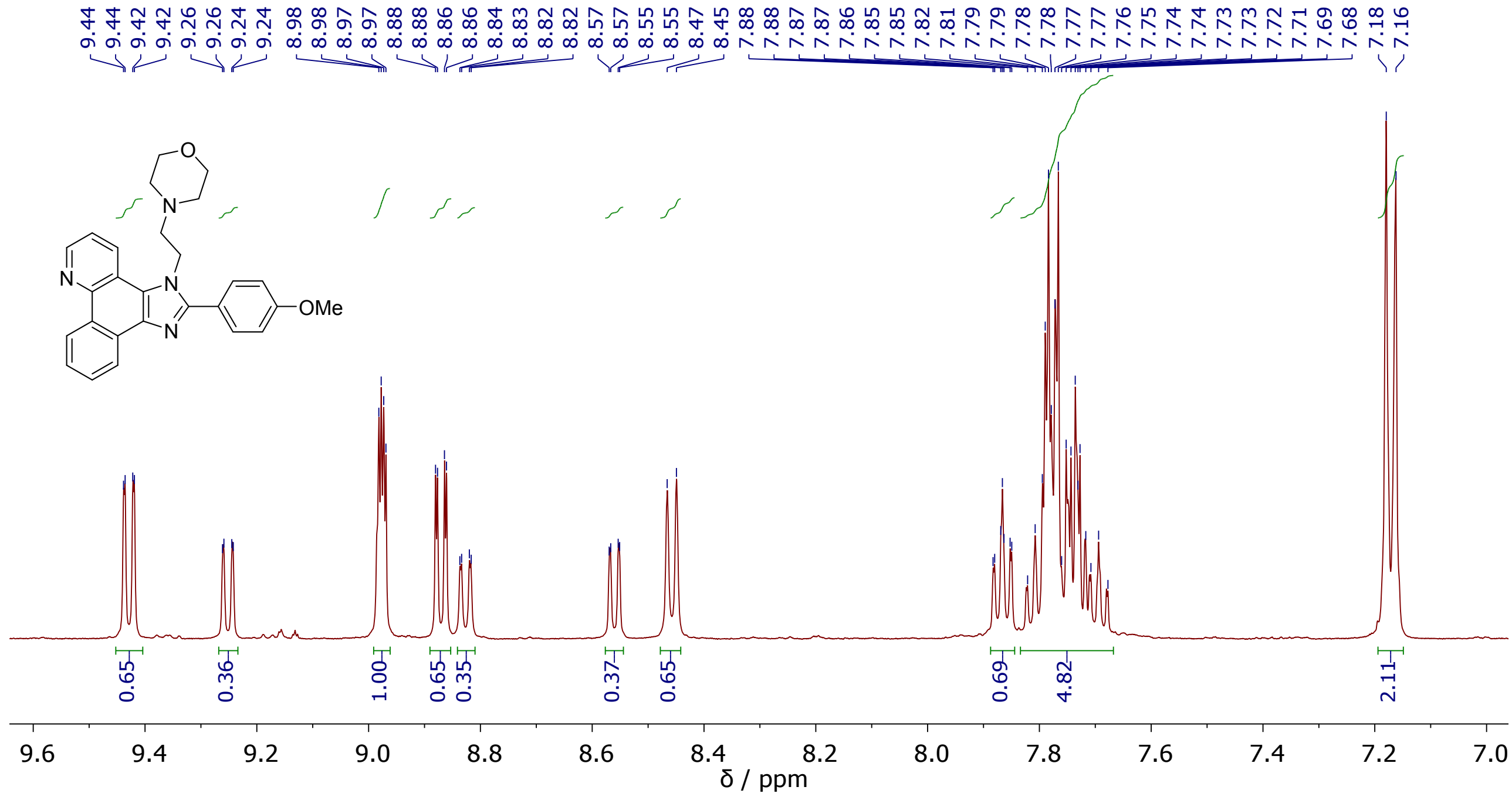

Figure S13. ^{1}H NMR (500 MHz, DMSO-d_6, 298 K) spectrum of **H-L3** in the aromatic region.

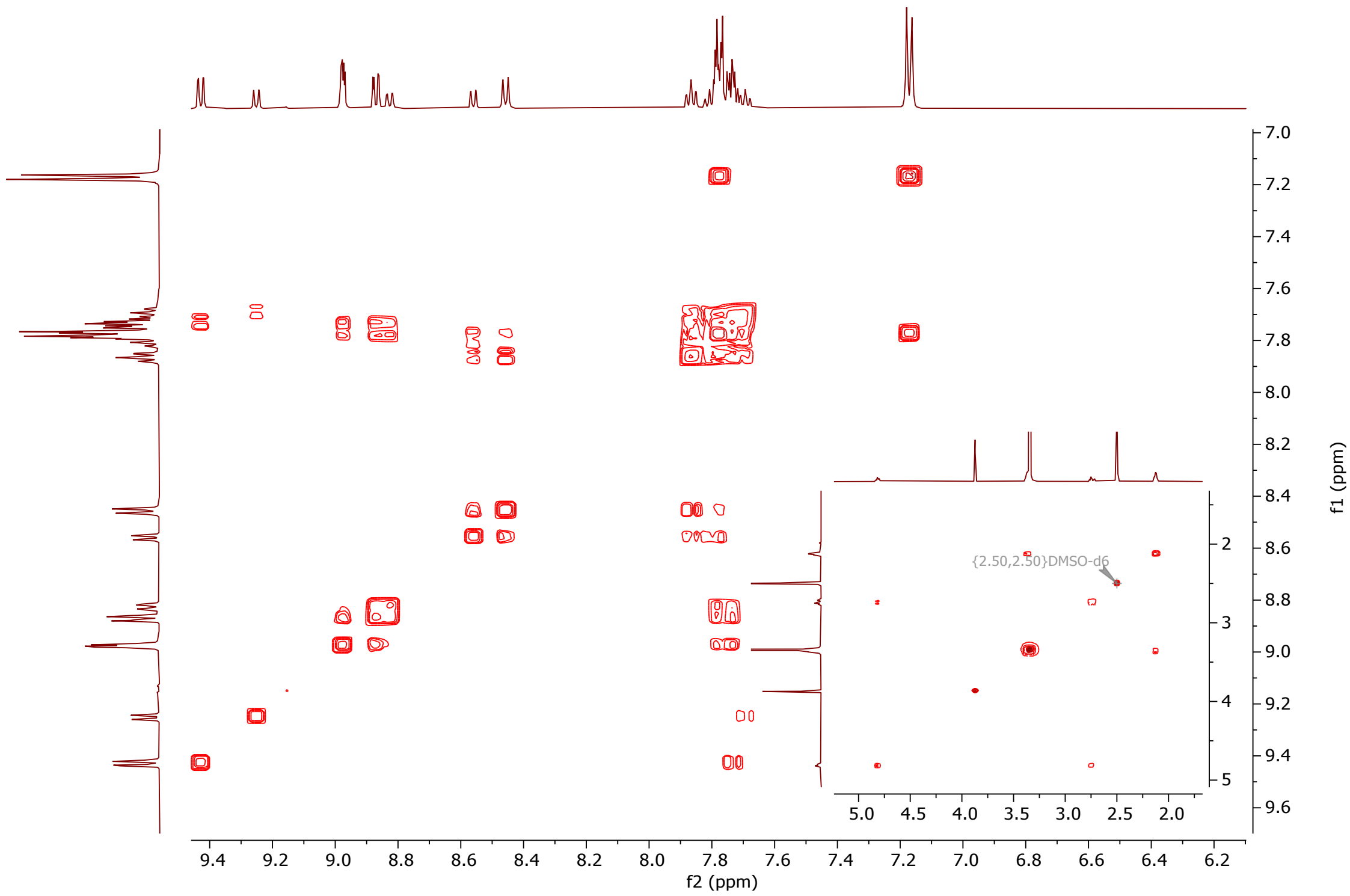

Figure S14. ^{1}H-^{1}H COSY (500 MHz, DMSO-d_6, 298 K) spectrum of **H-L3**.

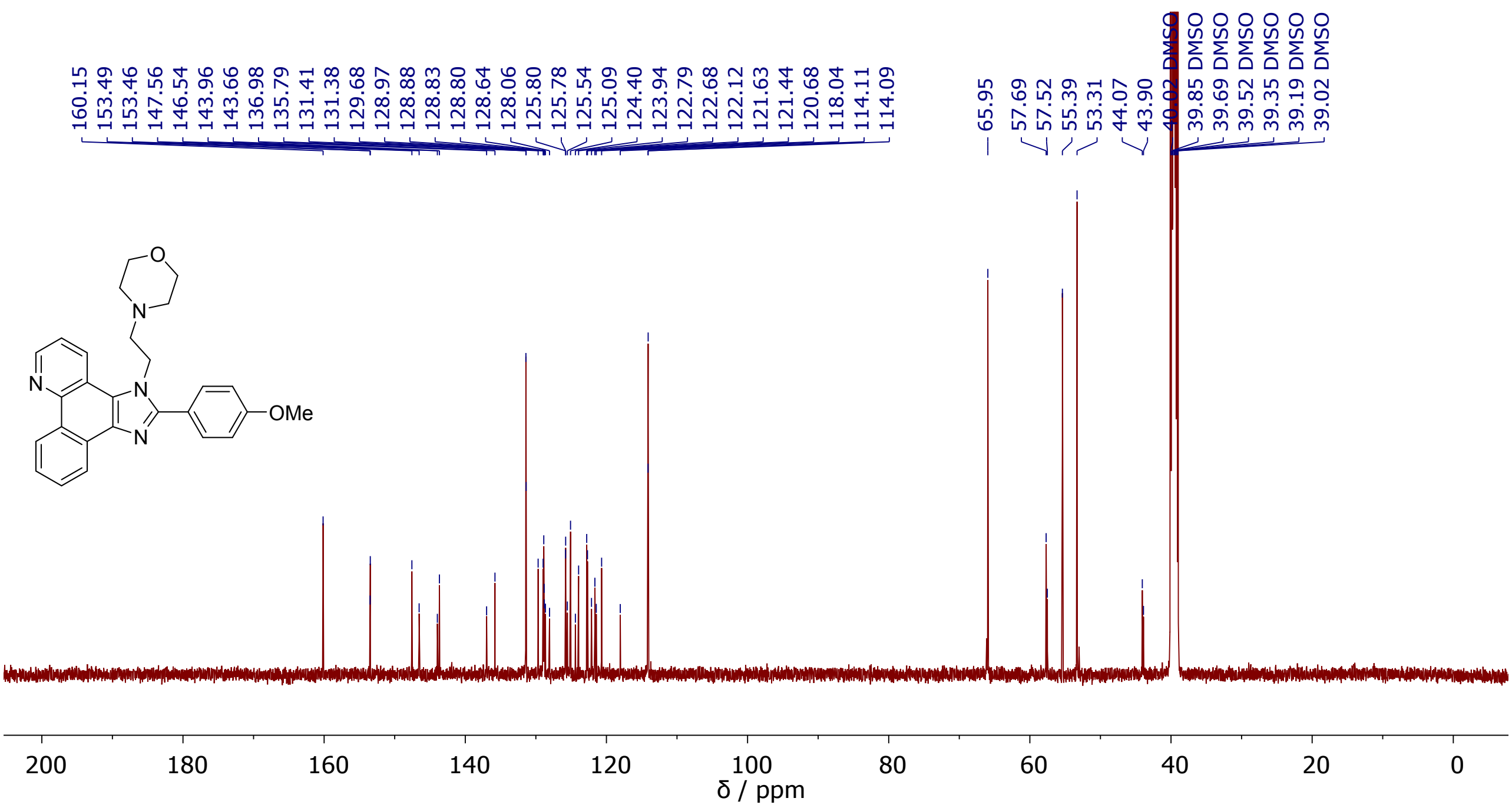

Figure S15. $^{13}C\{^{1}H\}$ NMR (126 MHz, DMSO-d_6, 298 K) spectrum of **H-L3**.

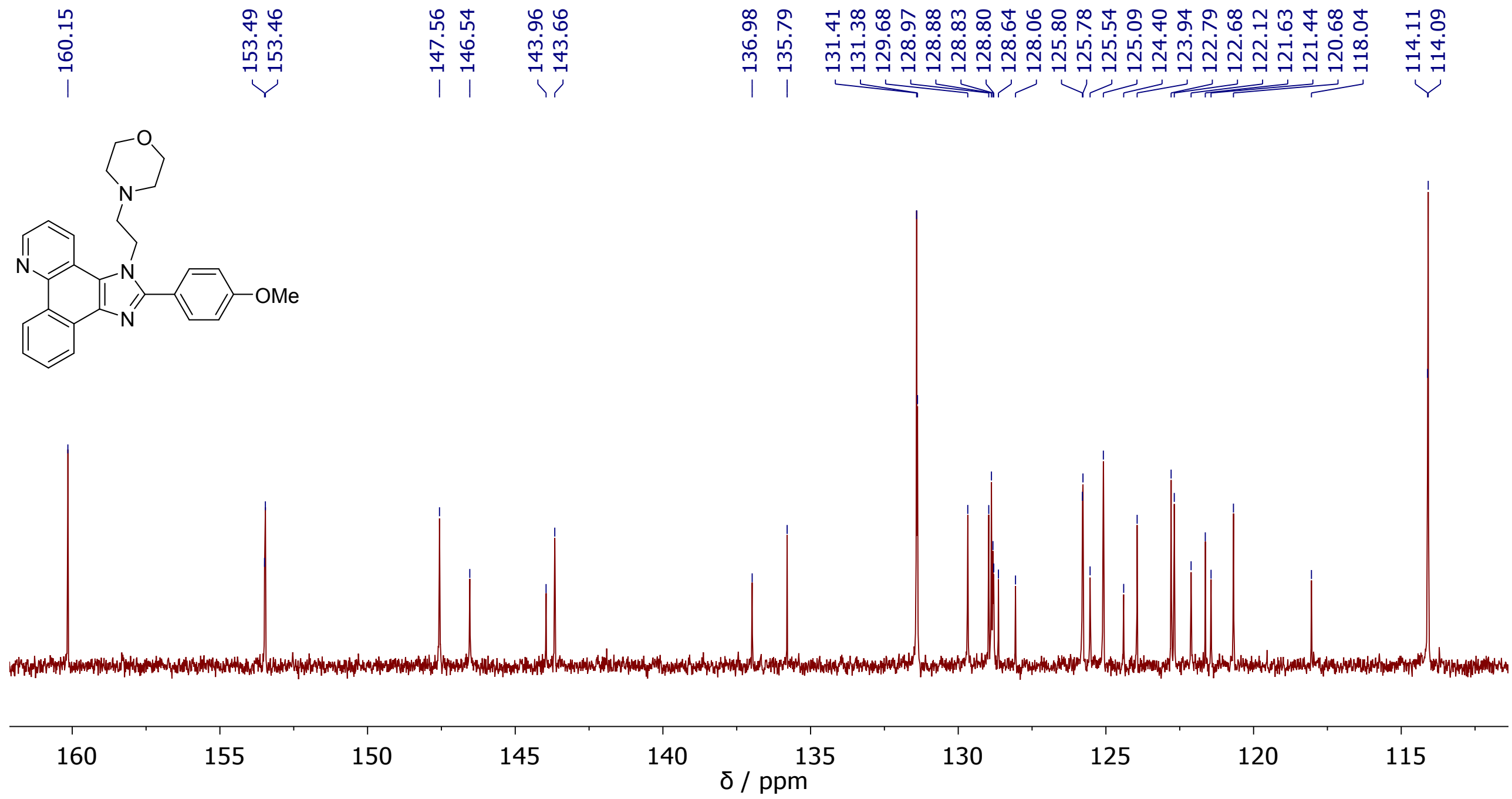

Figure S16. $^{13}C\{^1H\}$ NMR (126 MHz, DMSO-d_6, 298 K) spectrum of **H-L3** in the aromatic region.

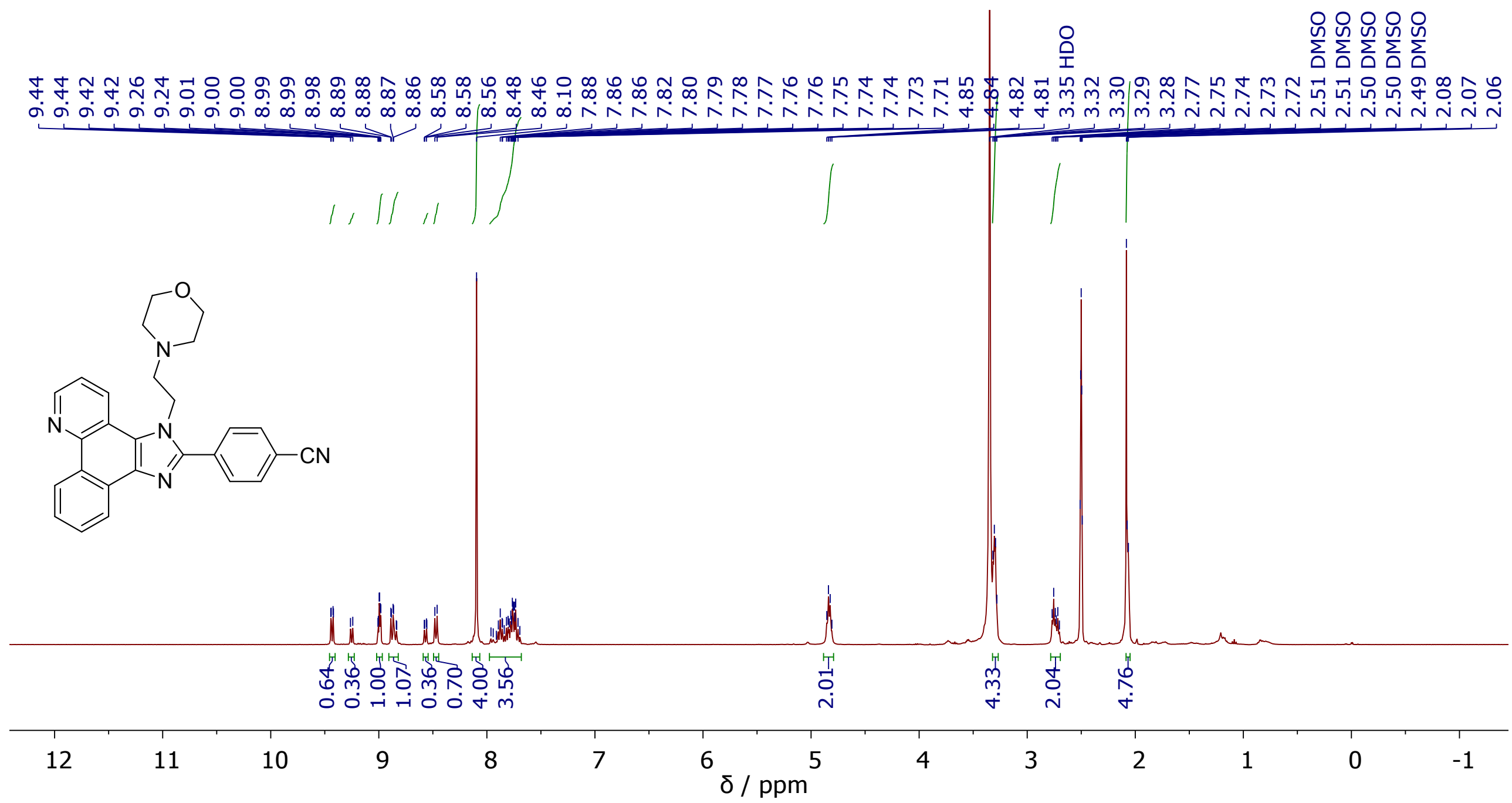

Figure S17. ^1H NMR (400 MHz, DMSO-d_6, 298 K) spectrum of **H-L4**.

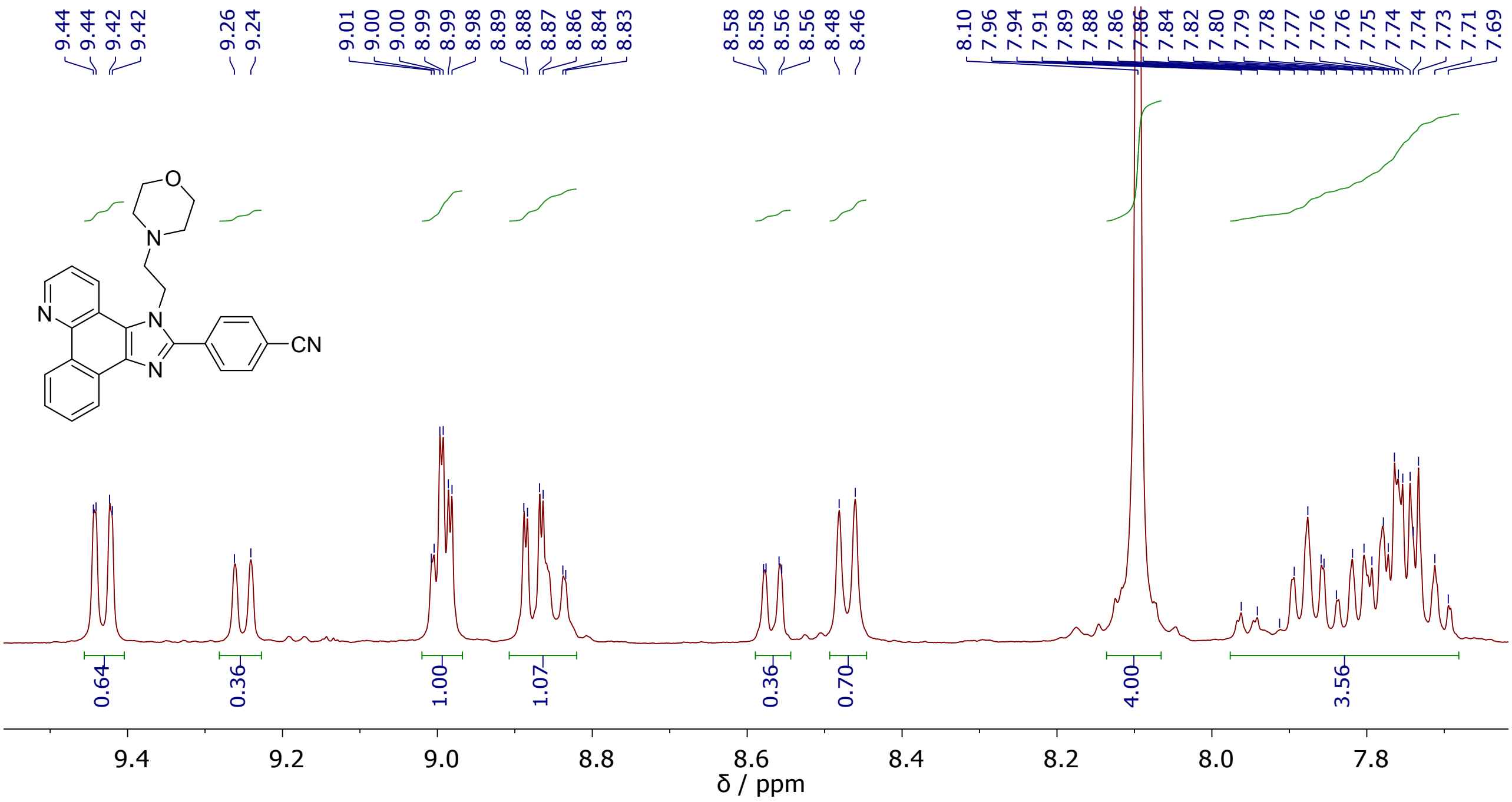

Figure S18. ^{1}H NMR (400 MHz, DMSO-d_6, 298 K) spectrum of **H-L4** in the aromatic region.

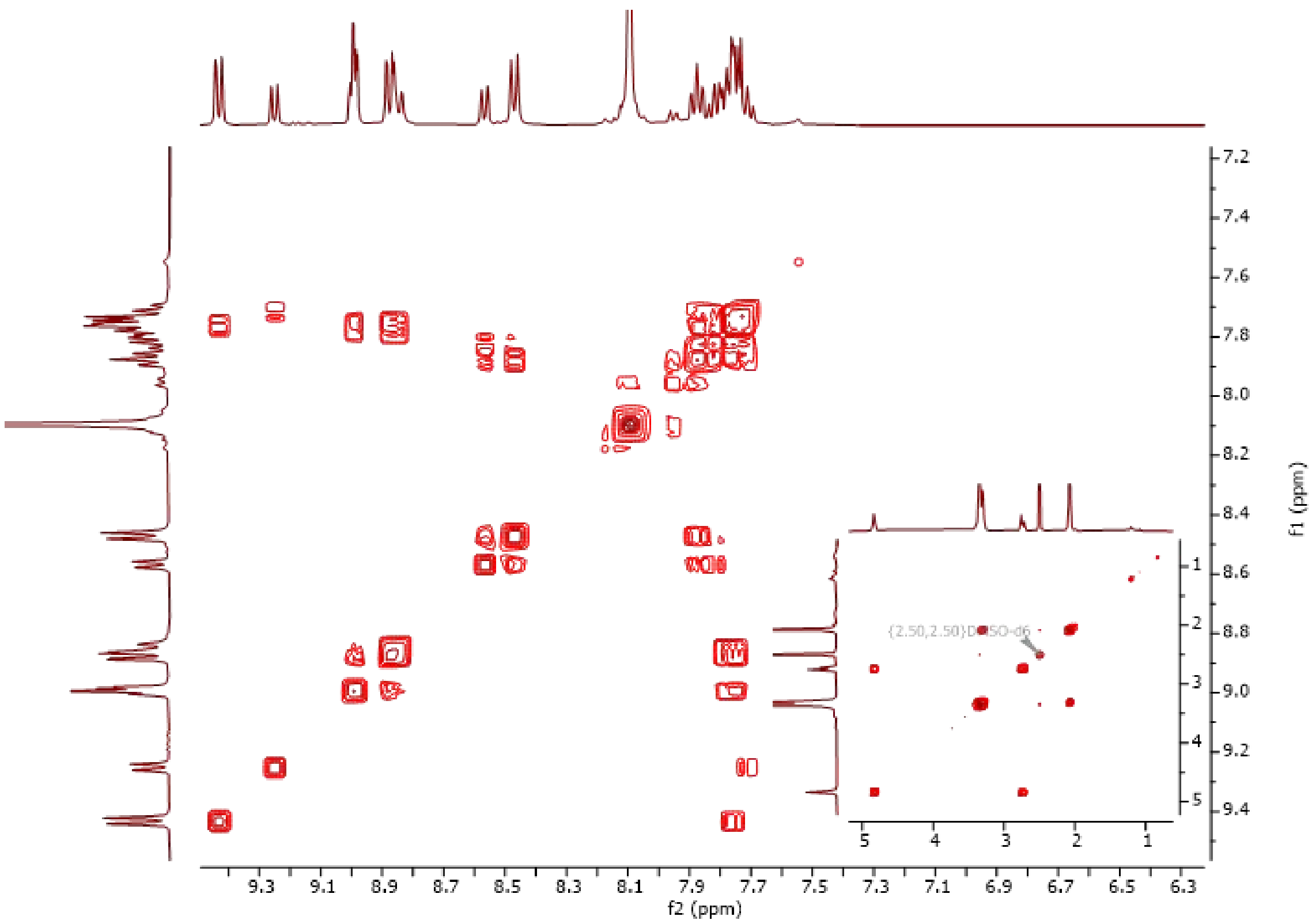

Figure S19. ^{1}H-^{1}H COSY (400 MHz, DMSO-d_6, 298 K) spectrum of **H-L4**.

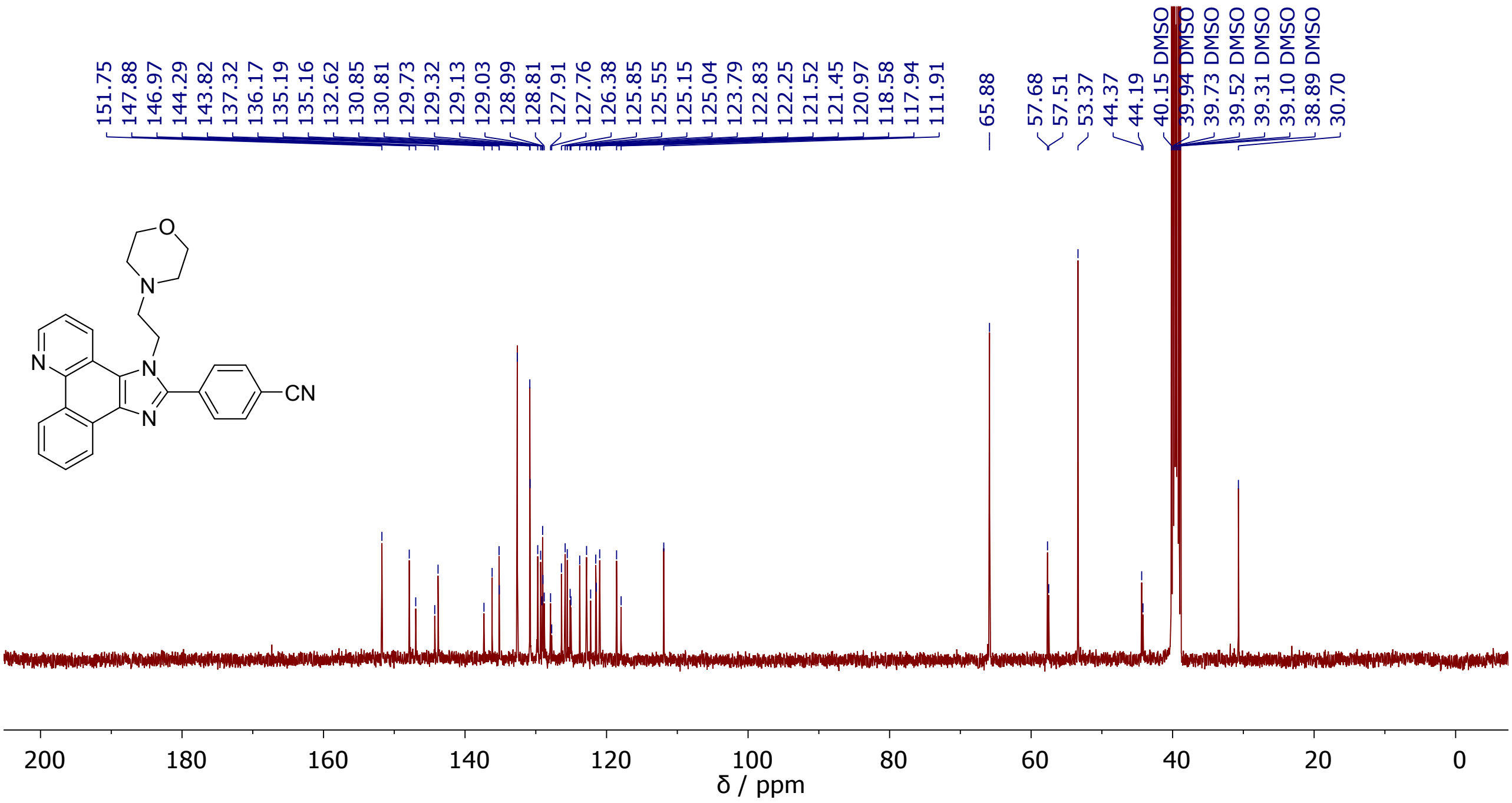

Figure S20. $^{13}C\{^{1}H\}$ NMR (101 MHz, DMSO-d_6, 298 K) spectrum of **H-L4**.

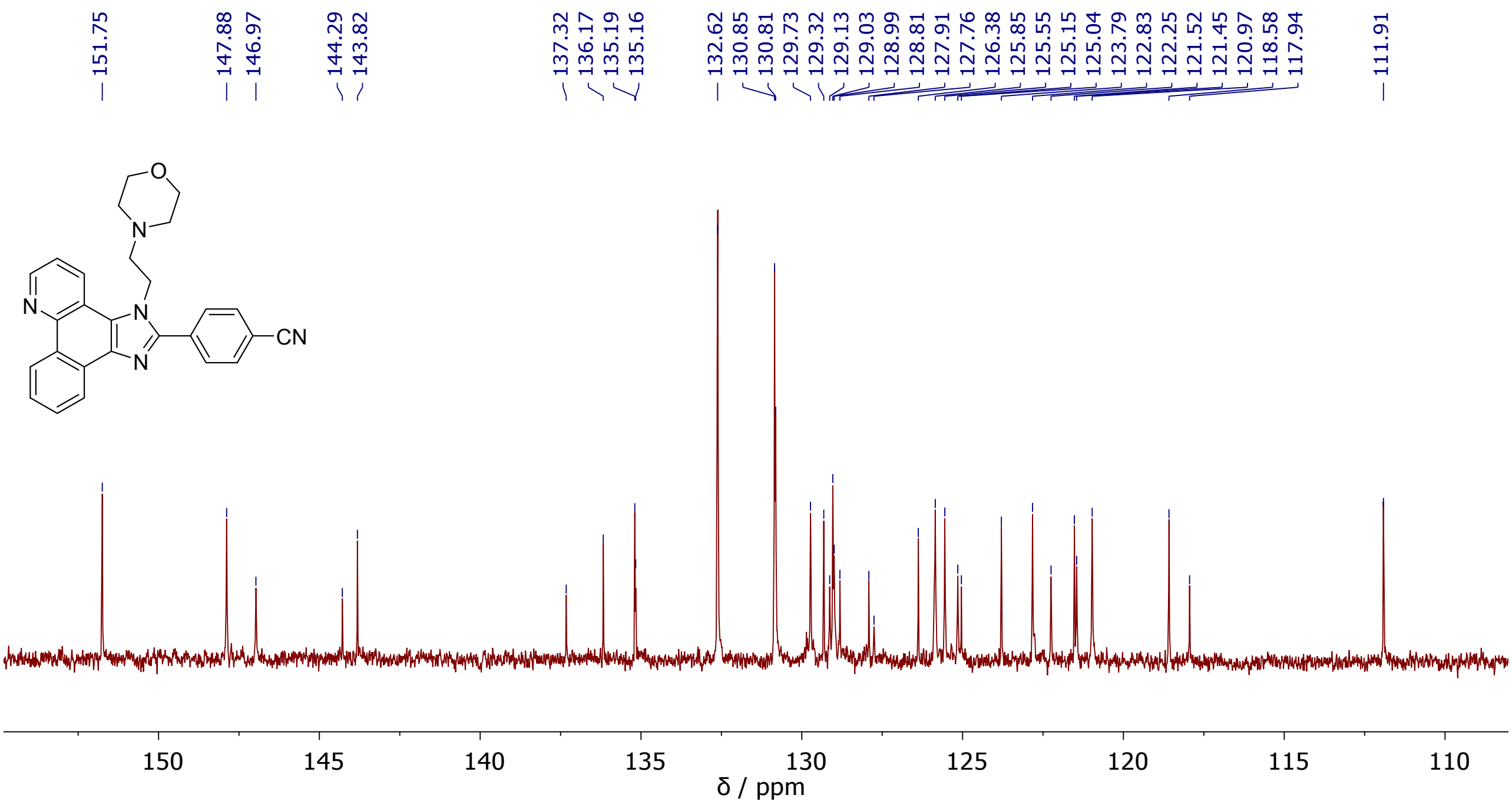

Figure S21. $^{13}C\{^{1}H\}$ NMR (101 MHz, DMSO-d_6, 298 K) spectrum of **H-L4** in the aromatic region.

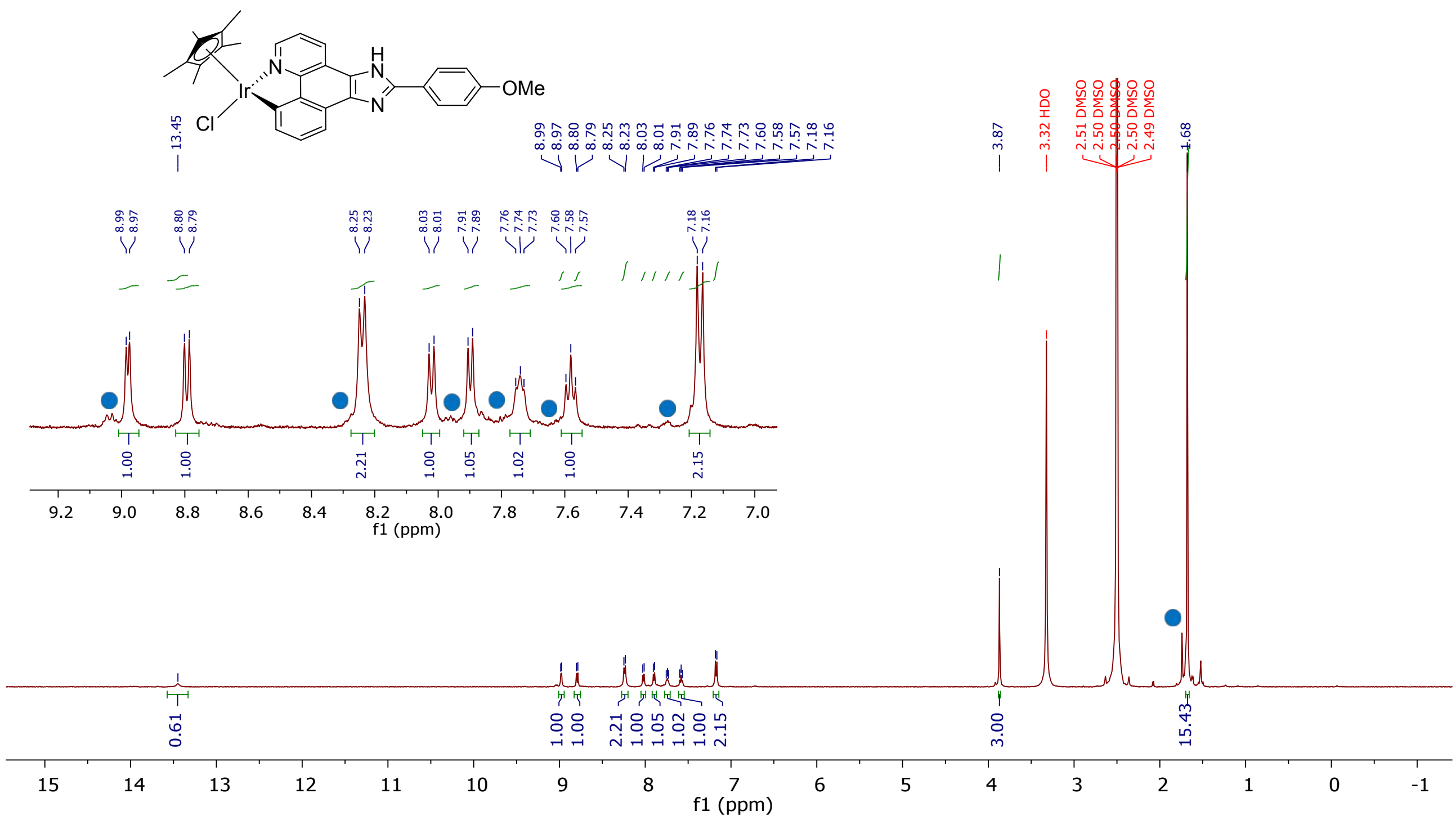

Figure S22. ^{1}H NMR (500 MHz, DMSO-d_6, 298 K) spectrum of **C1**. Blue circles correspond to the solvolysis product.

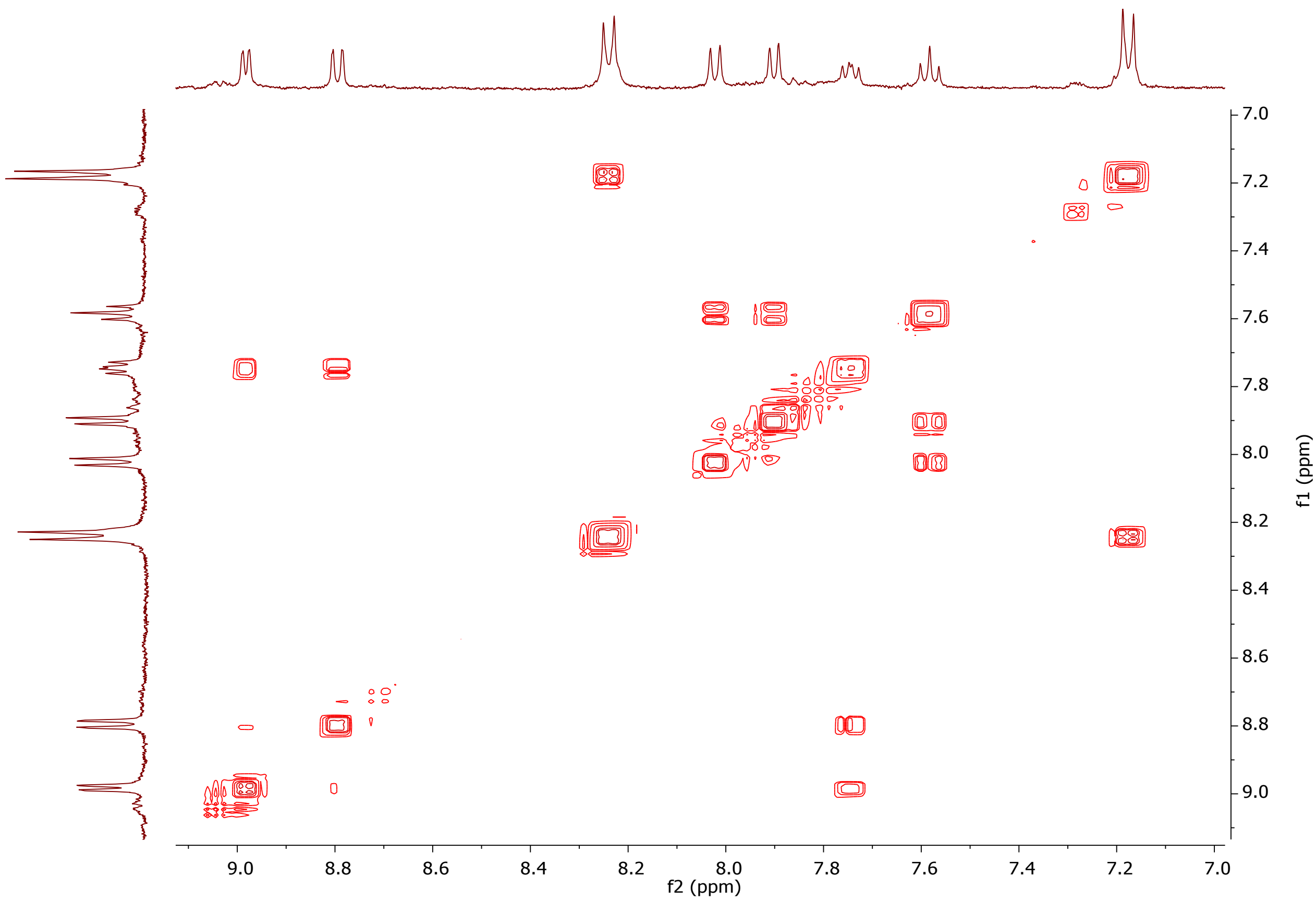

Figure S23. ^{1}H-^{1}H COSY (500 MHz, DMSO-d_6, 298 K) spectrum of **C1**.

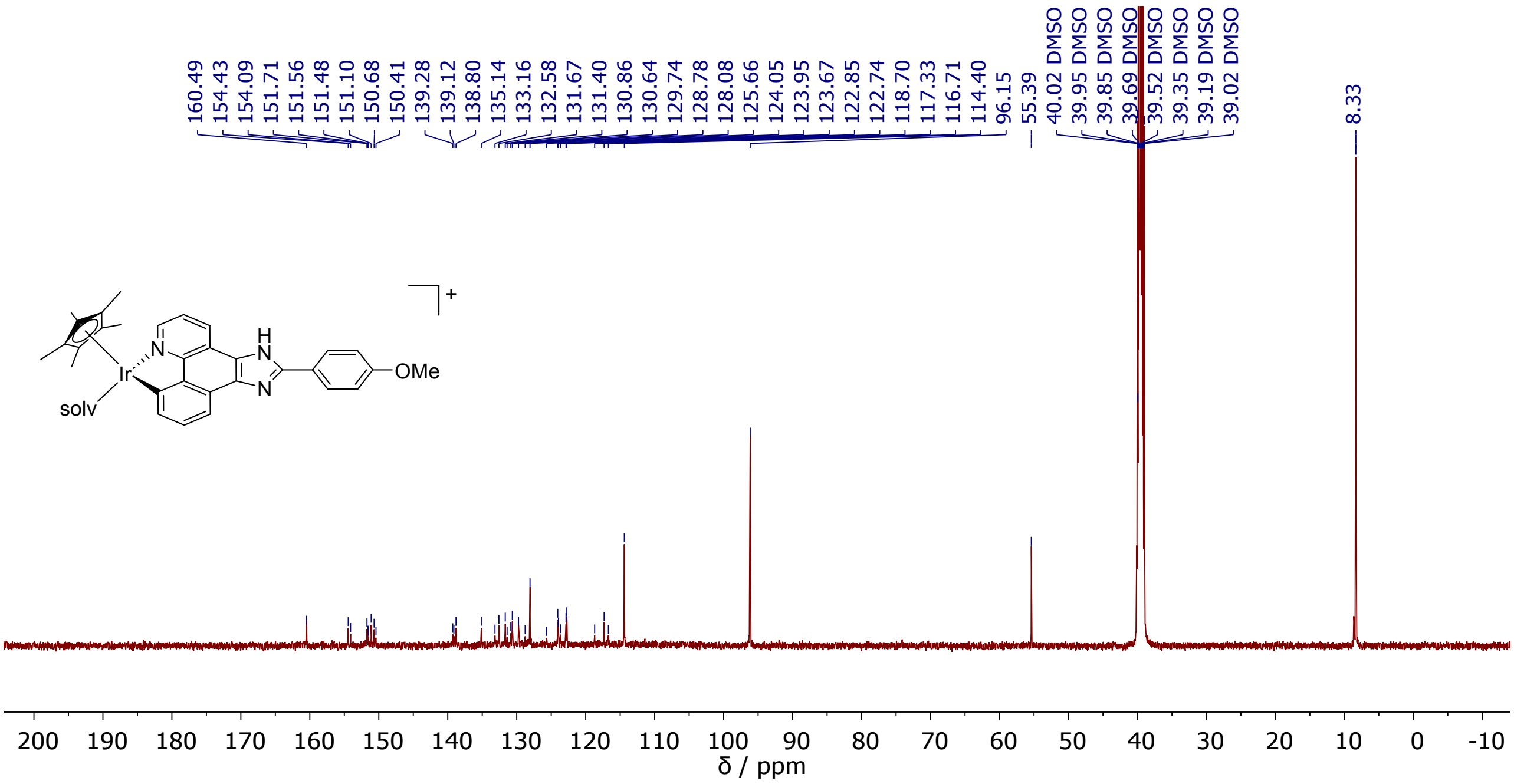

Figure S24. $^{13}C\{^{1}H\}$ NMR (126 MHz, DMSO-d_6, 298 K) spectrum of solvolysis product of **C1**.

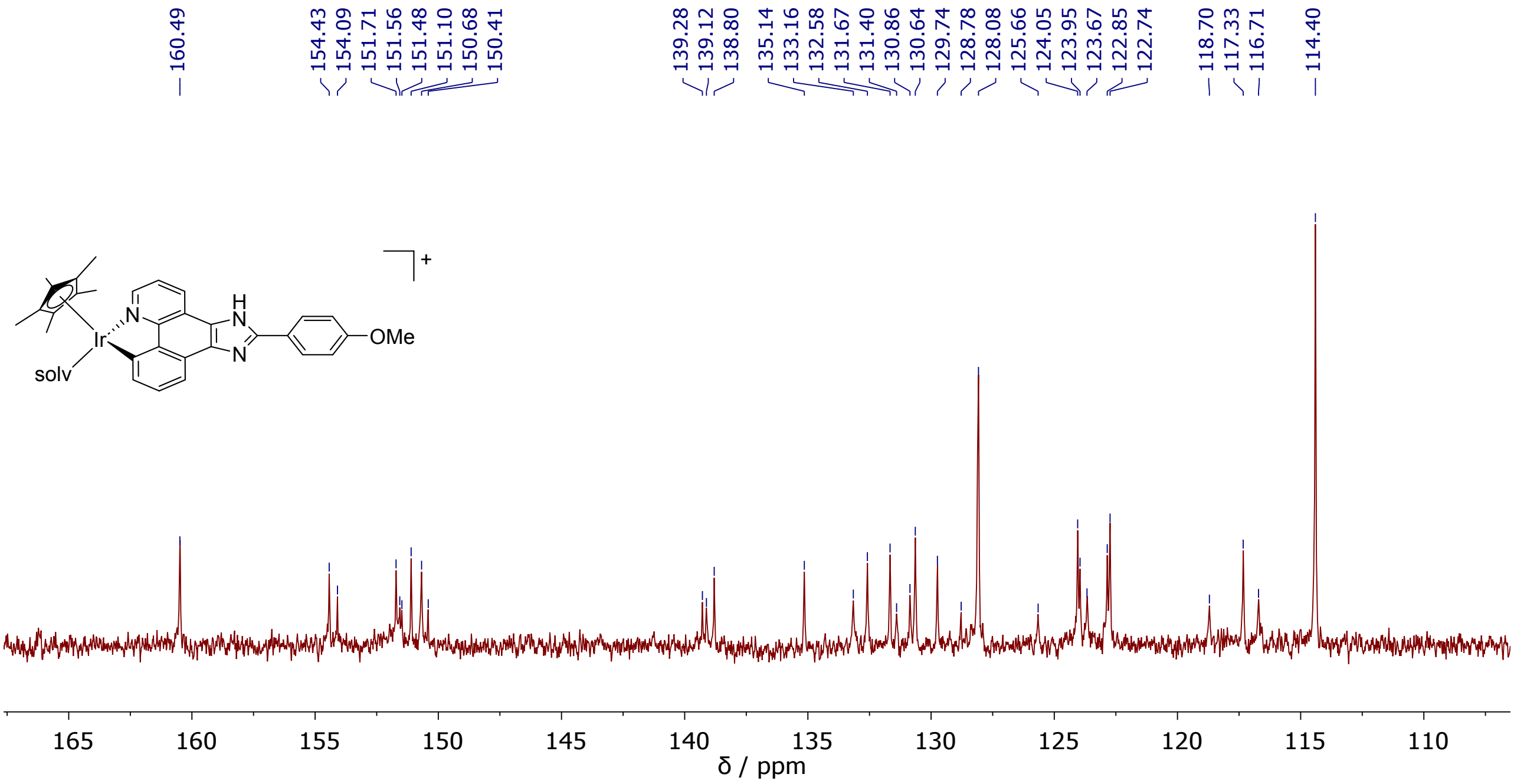

Figure S25. $^{13}C\{^{1}H\}$ NMR (126 MHz, DMSO-d_6, 298 K) spectrum of solvolysis product of **C1** in the aromatic region.

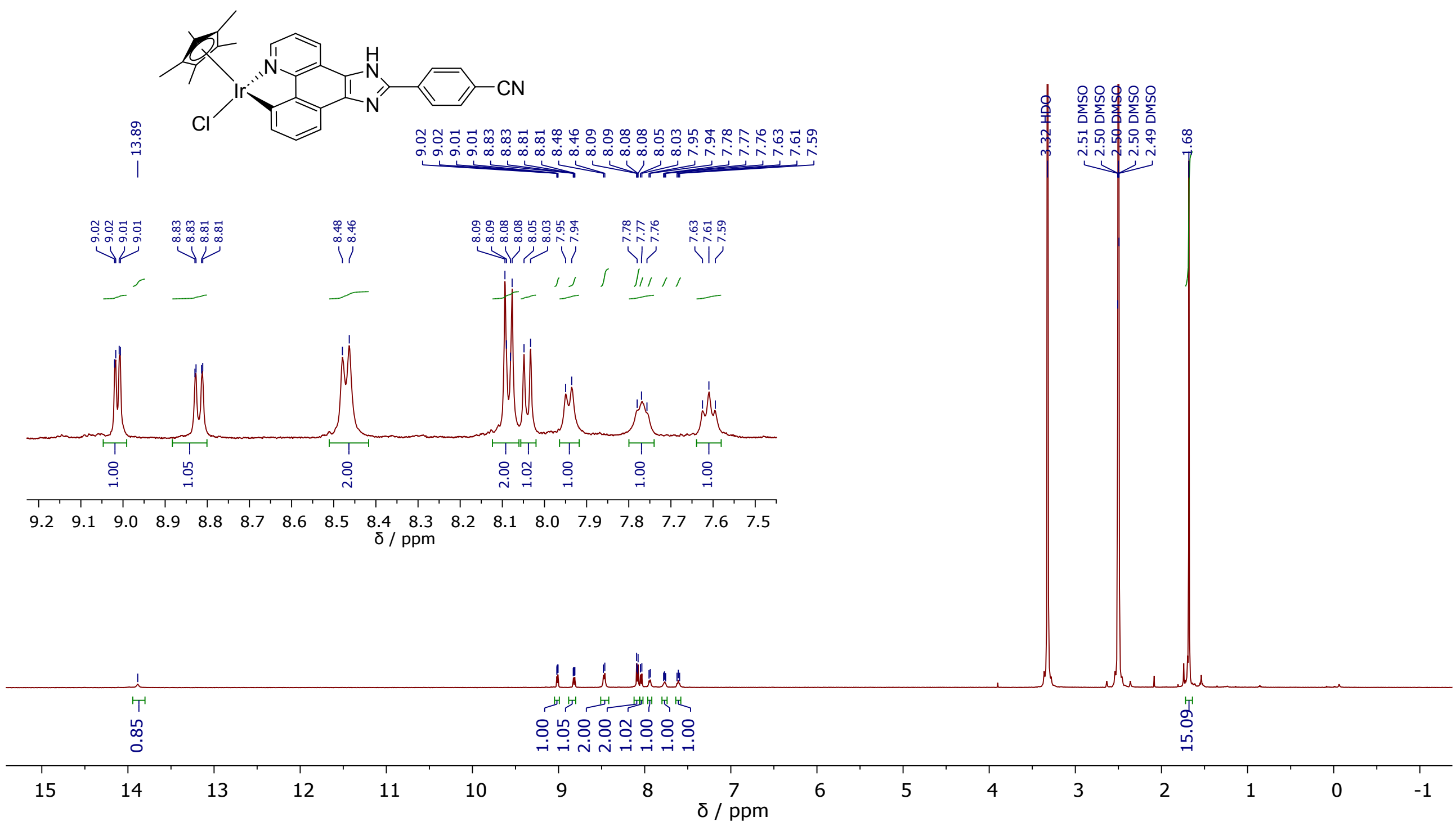

Figure S26. ^{1}H NMR (500 MHz, DMSO-d_6, 298 K) spectrum of **C2**.

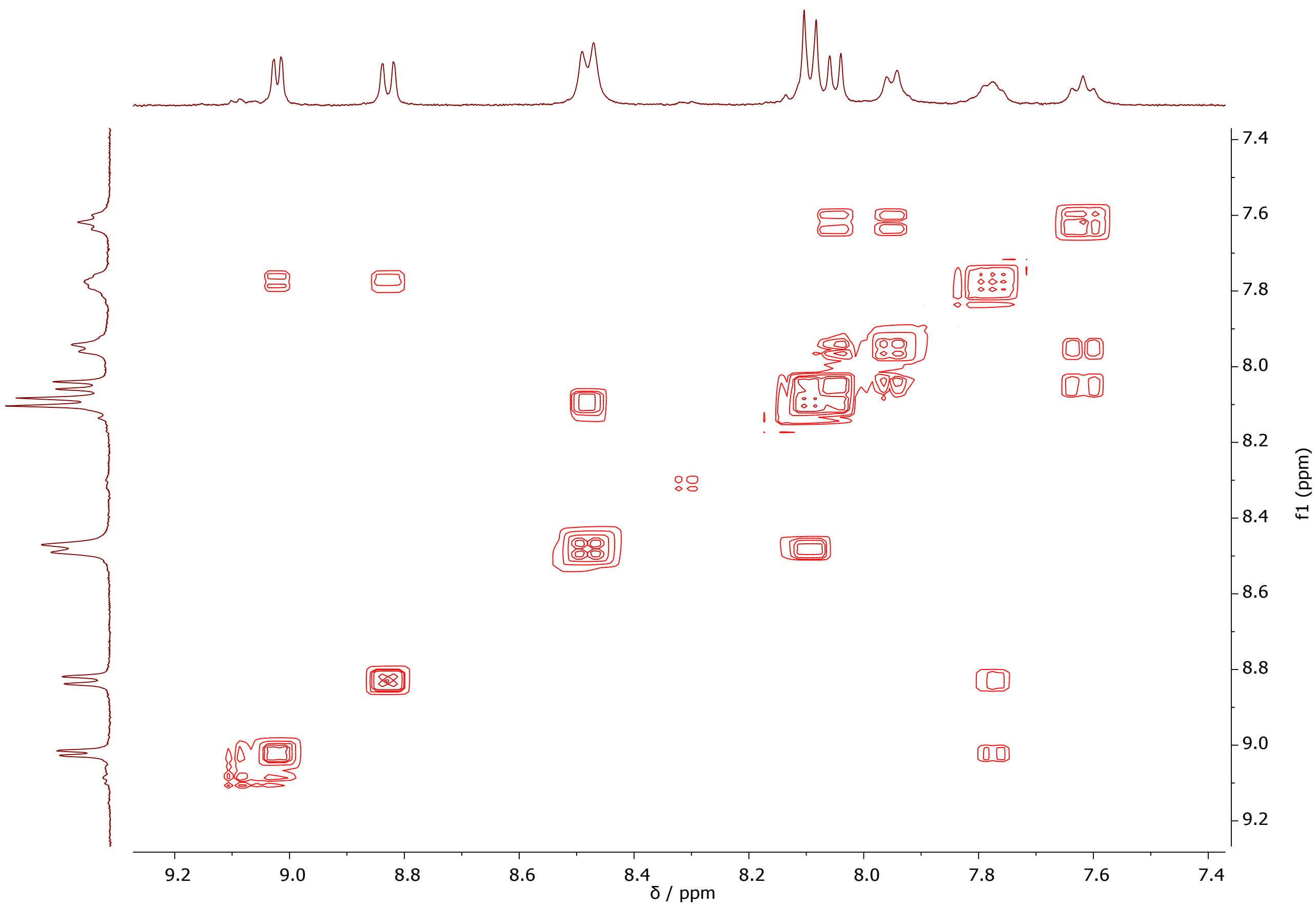

Figure S27. ^{1}H-^{1}H COSY (500 MHz, DMSO-d_6, 298 K) spectrum of **C2**.

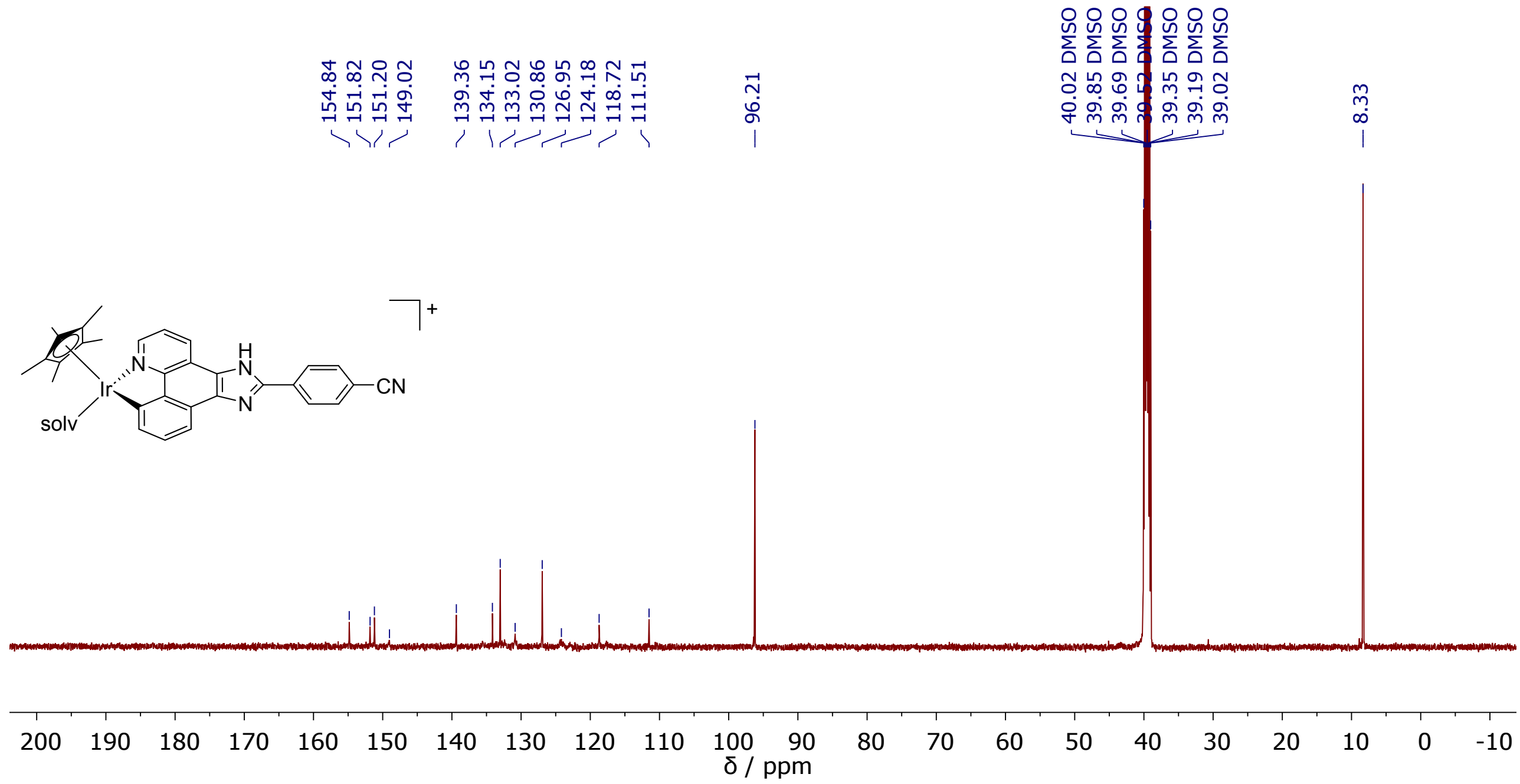

Figure S28. $^{13}C\{^{1}H\}$ NMR (126 MHz, DMSO-d_6, 298 K) spectrum of solvolysis product of **C2**.

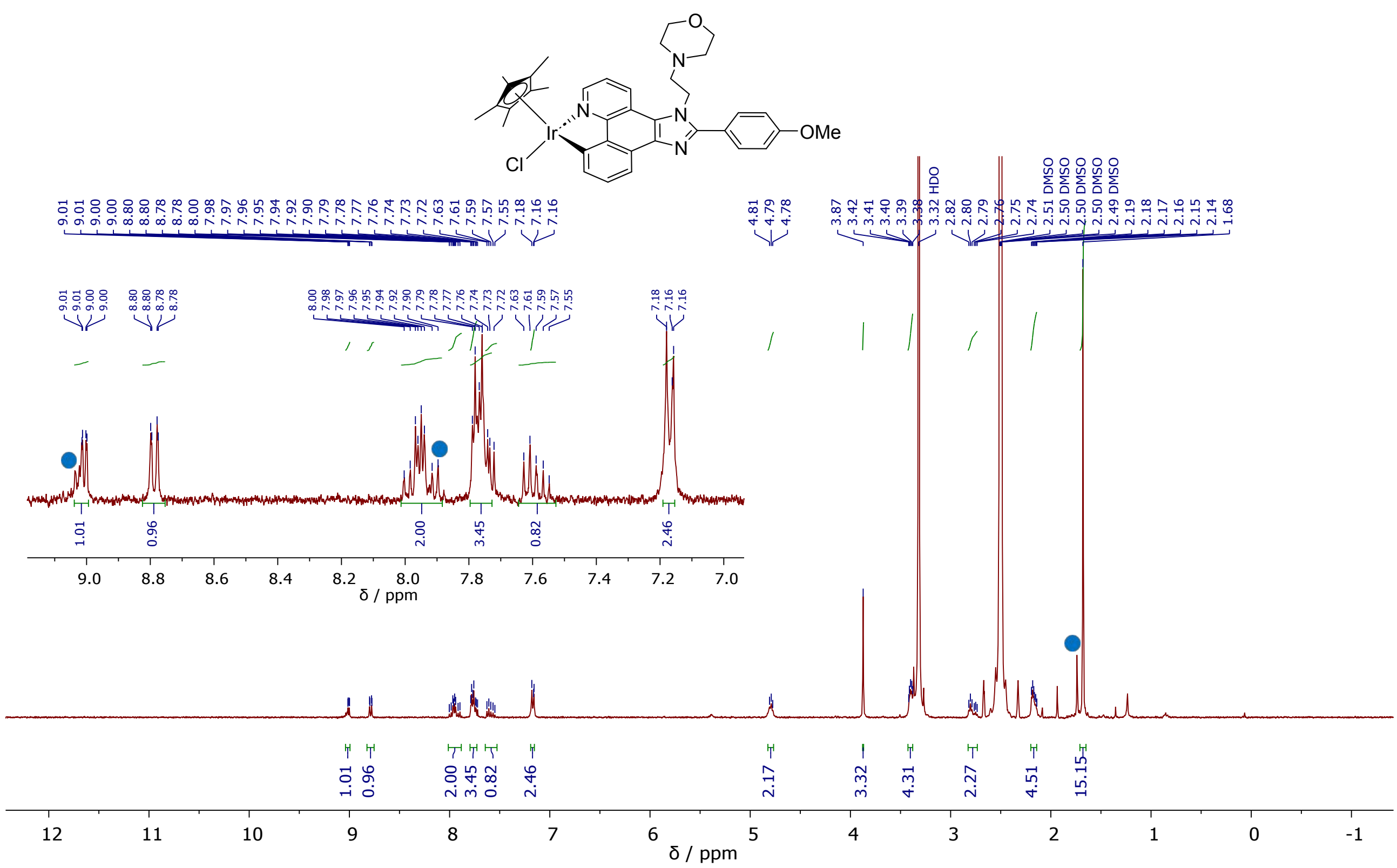

Figure S29. ^{1}H NMR (500 MHz, DMSO-d_6, 298 K) spectrum of **C3**. Blue circles correspond to the solvolysis product.

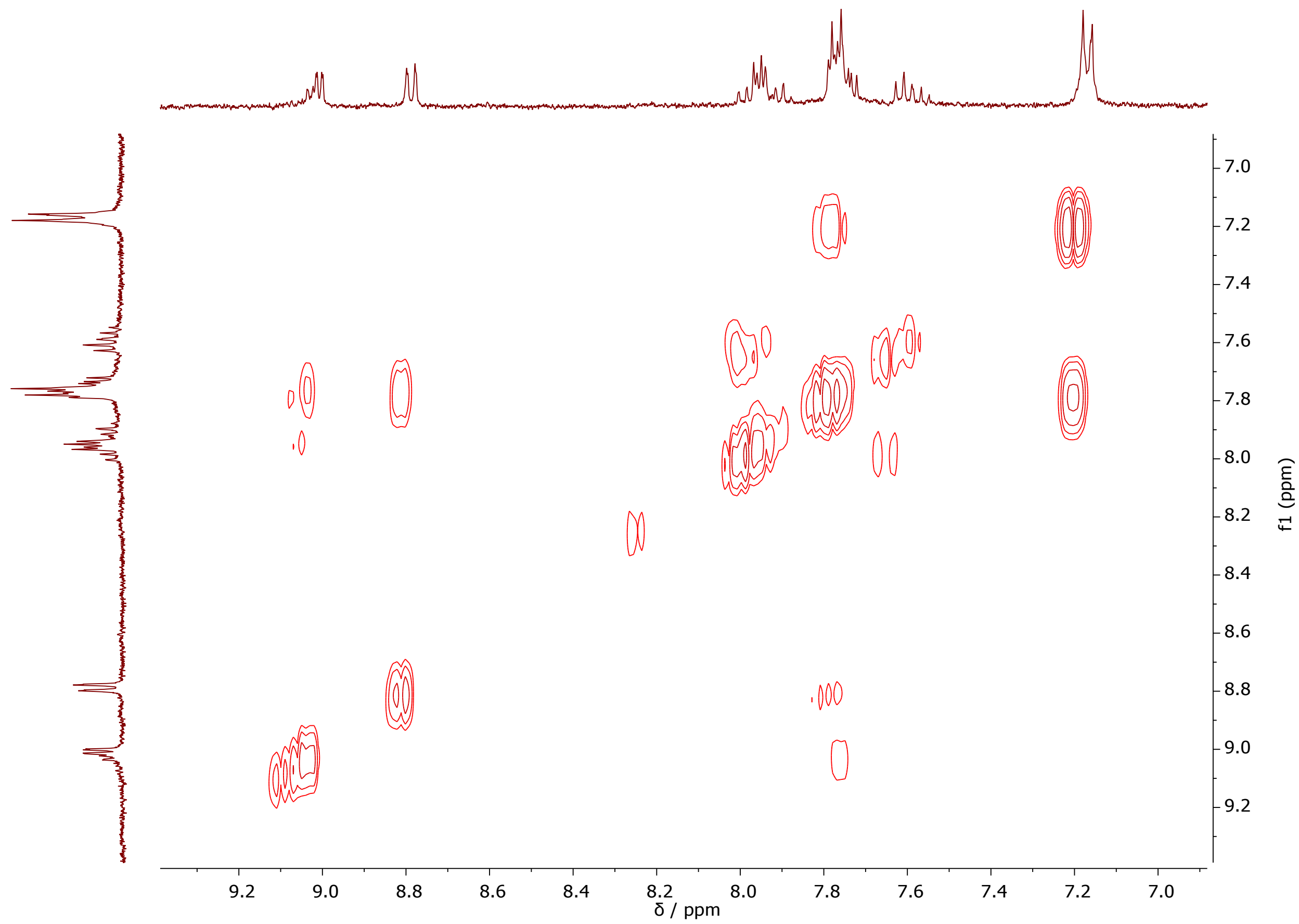

Figure S30. ^{1}H-^{1}H COSY (500 MHz, DMSO-d_6, 298 K) spectrum of **C3**.

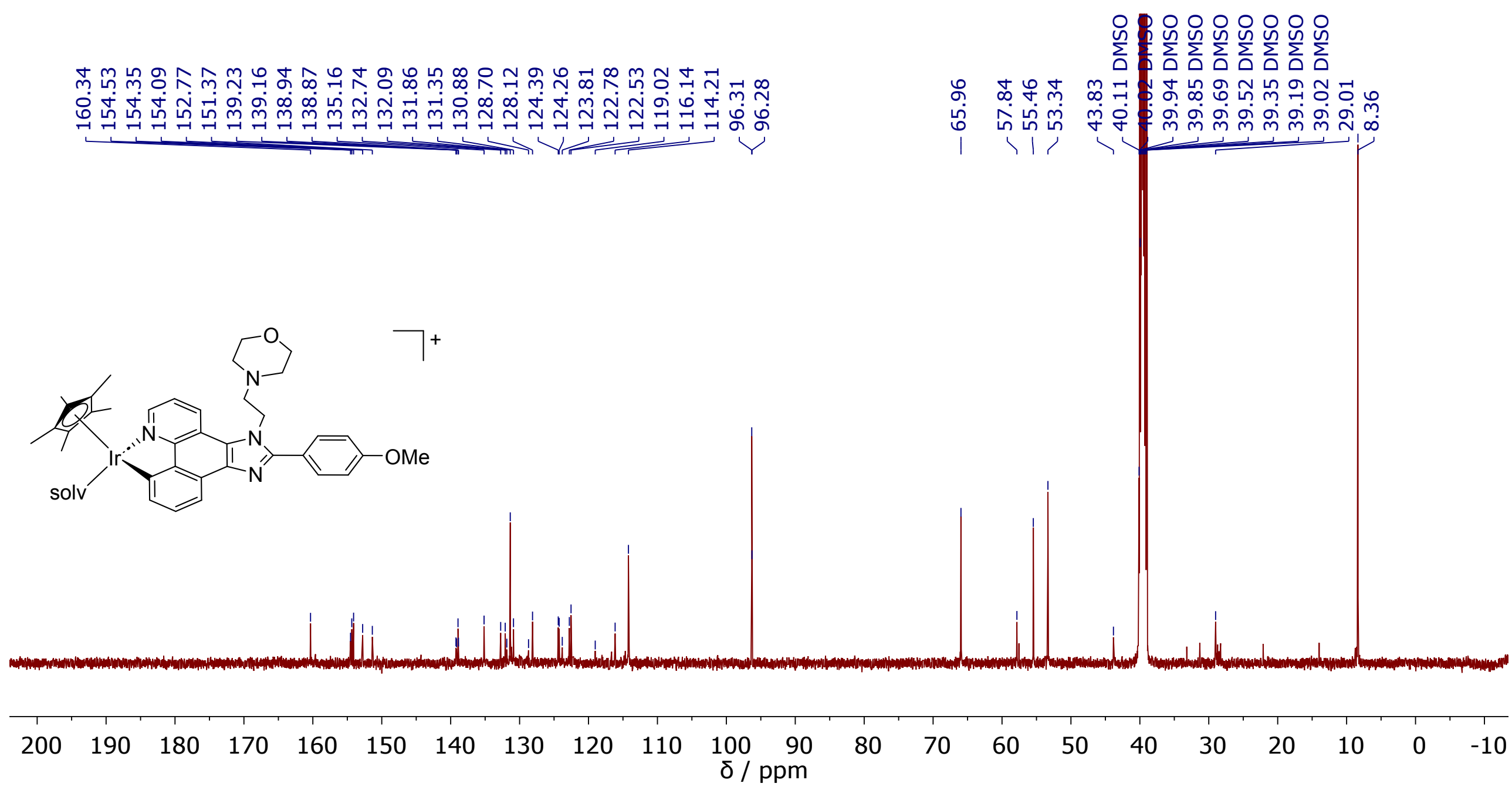

Figure S31. $^{13}C\{^{1}H\}$ NMR (126 MHz, DMSO-d_6, 298 K) spectrum of solvolysis product of **C3**.

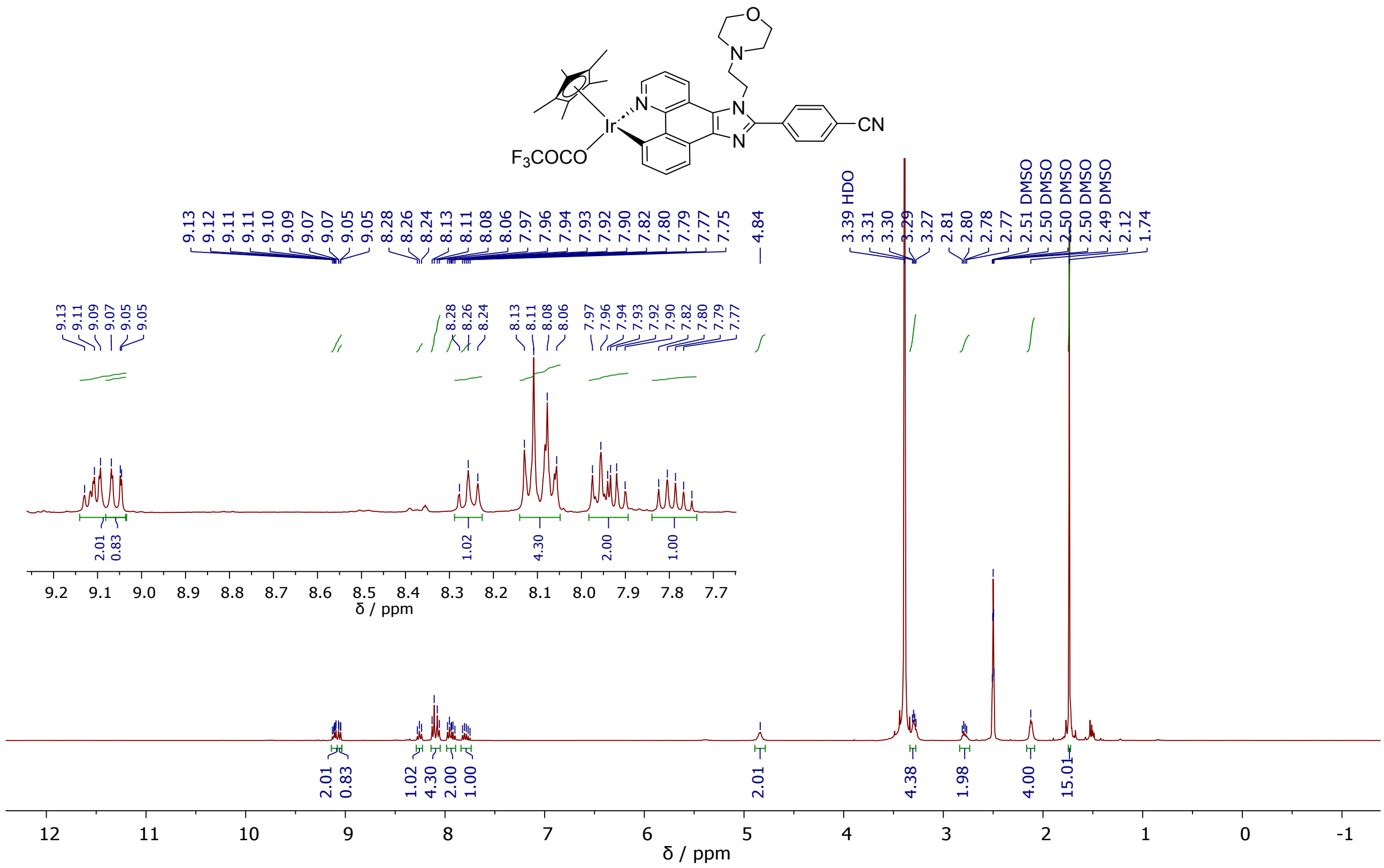

Figure S32. ^{1}H NMR (400 MHz, DMSO-d_6, 298 K) spectrum of **C4**.

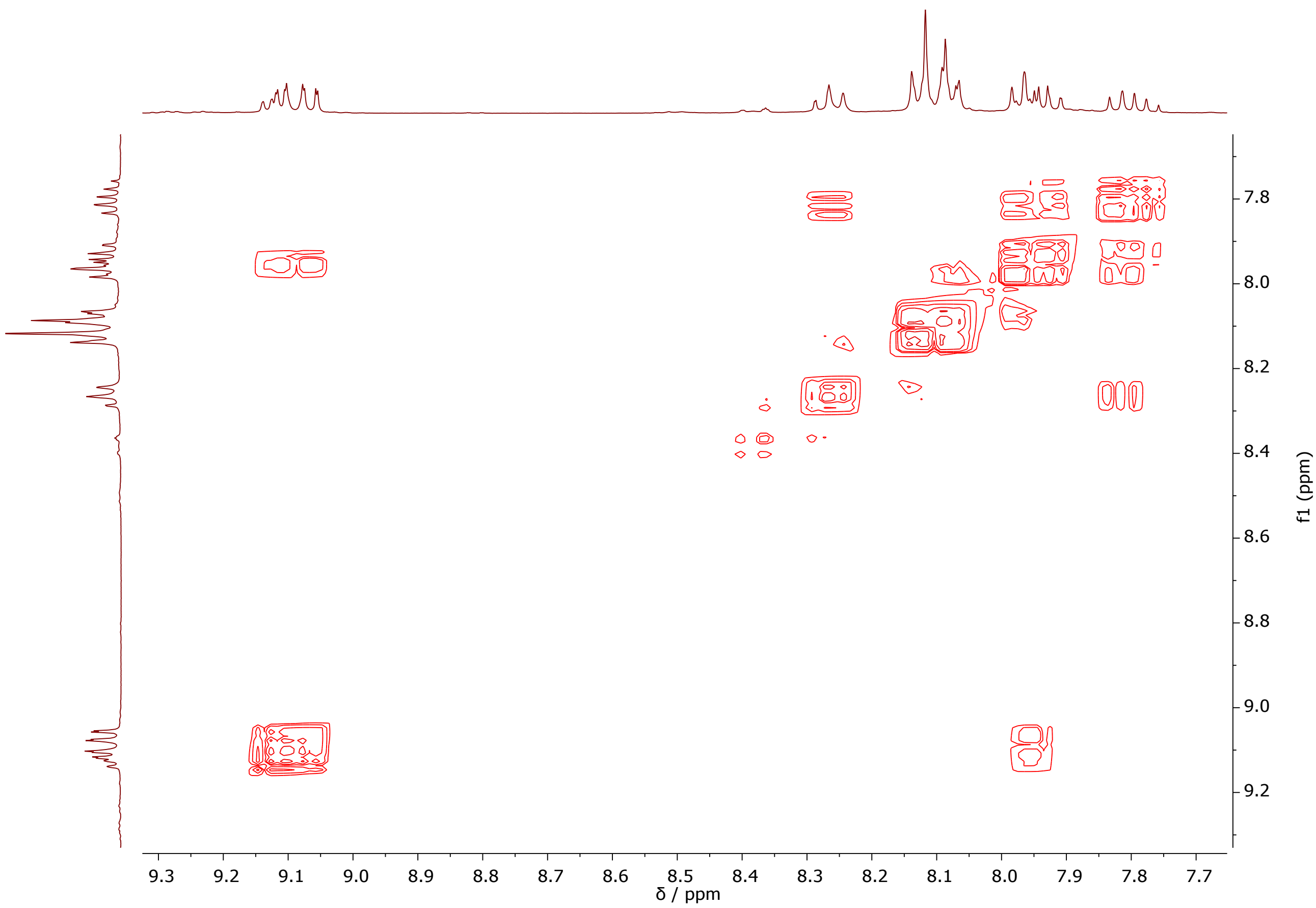

Figure S33. ^{1}H-^{1}H COSY (400 MHz, DMSO-d_6, 298 K) spectrum of **C4**.

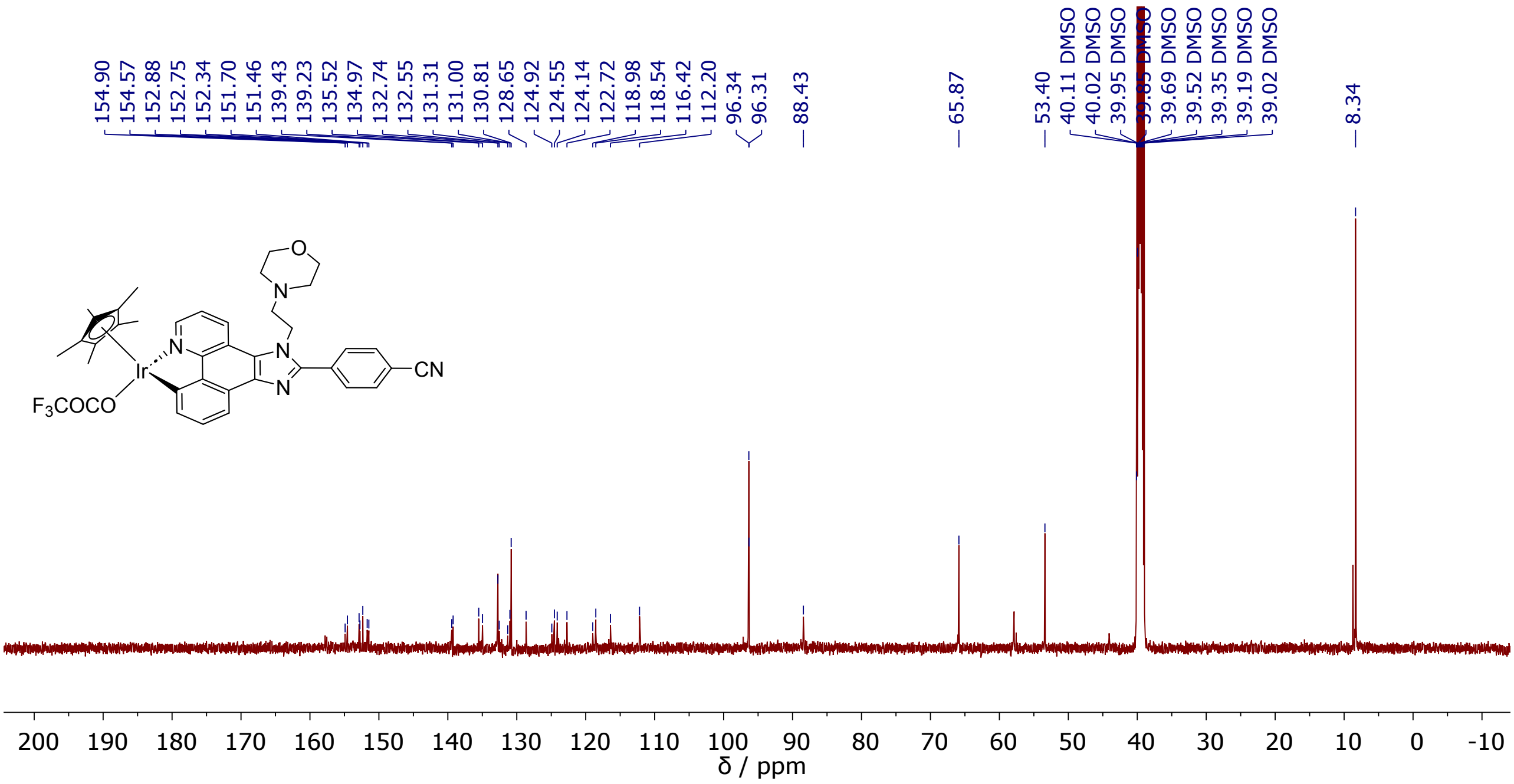

Figure S34. $^{13}C\{^{1}H\}$ NMR (126 MHz, DMSO-d_6, 298 K) spectrum of **C4**.

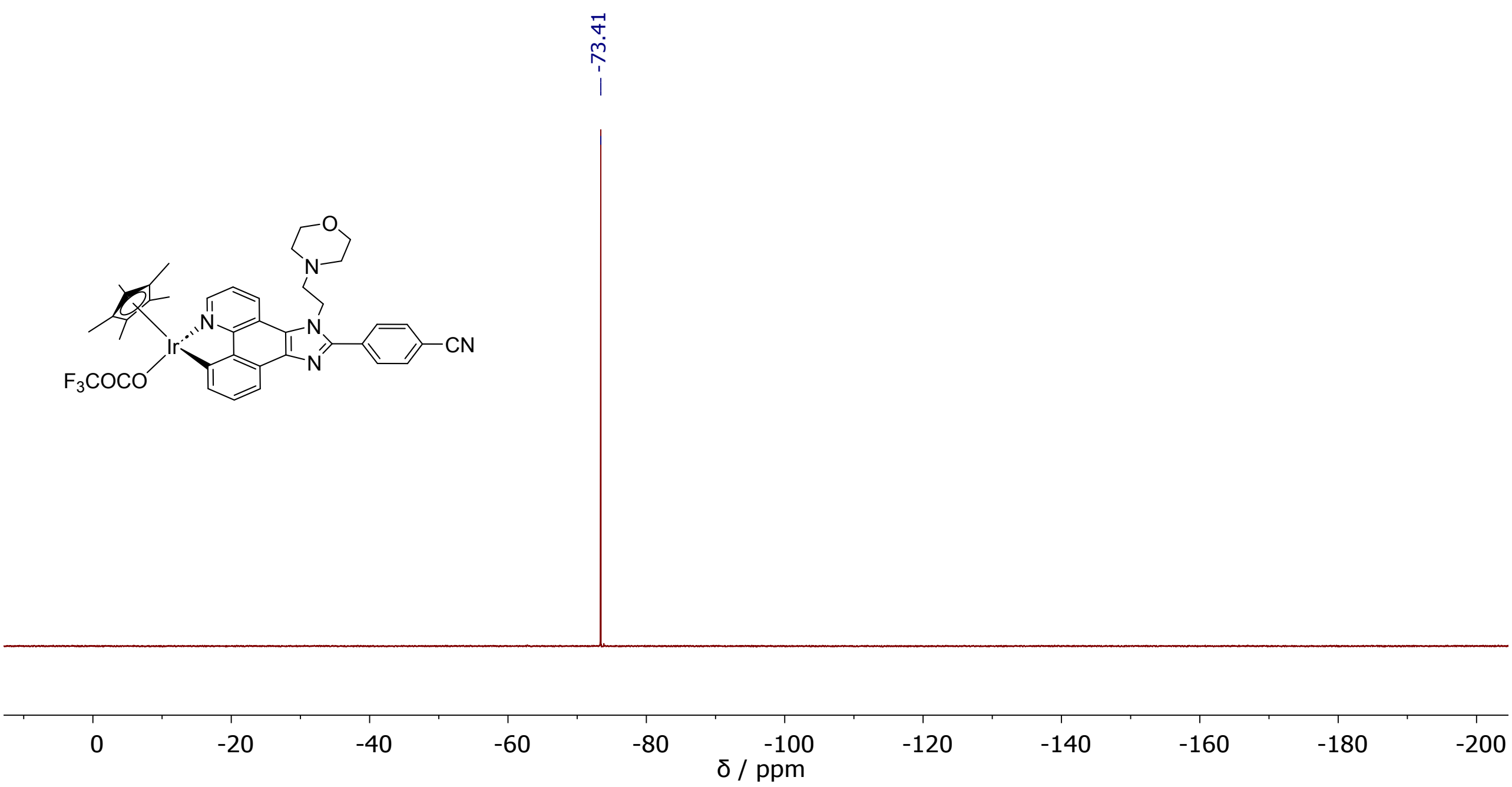

Figure S35. ^{19}F NMR (471 MHz, DMSO-d_6, 298 K) spectrum of **C4**.

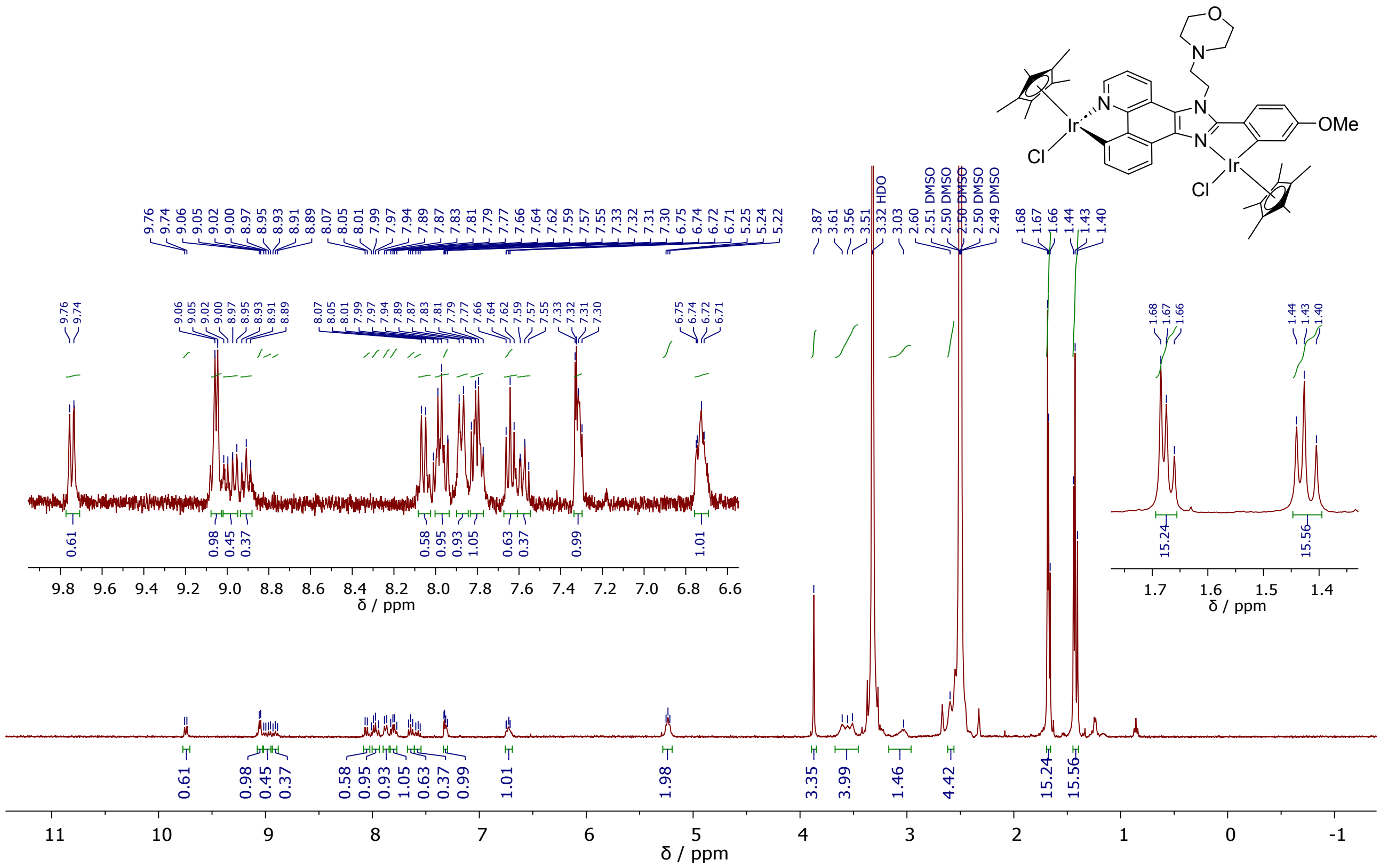

Figure S36. ^{1}H NMR (400 MHz, DMSO-d_6, 298 K) spectrum of **C5**.

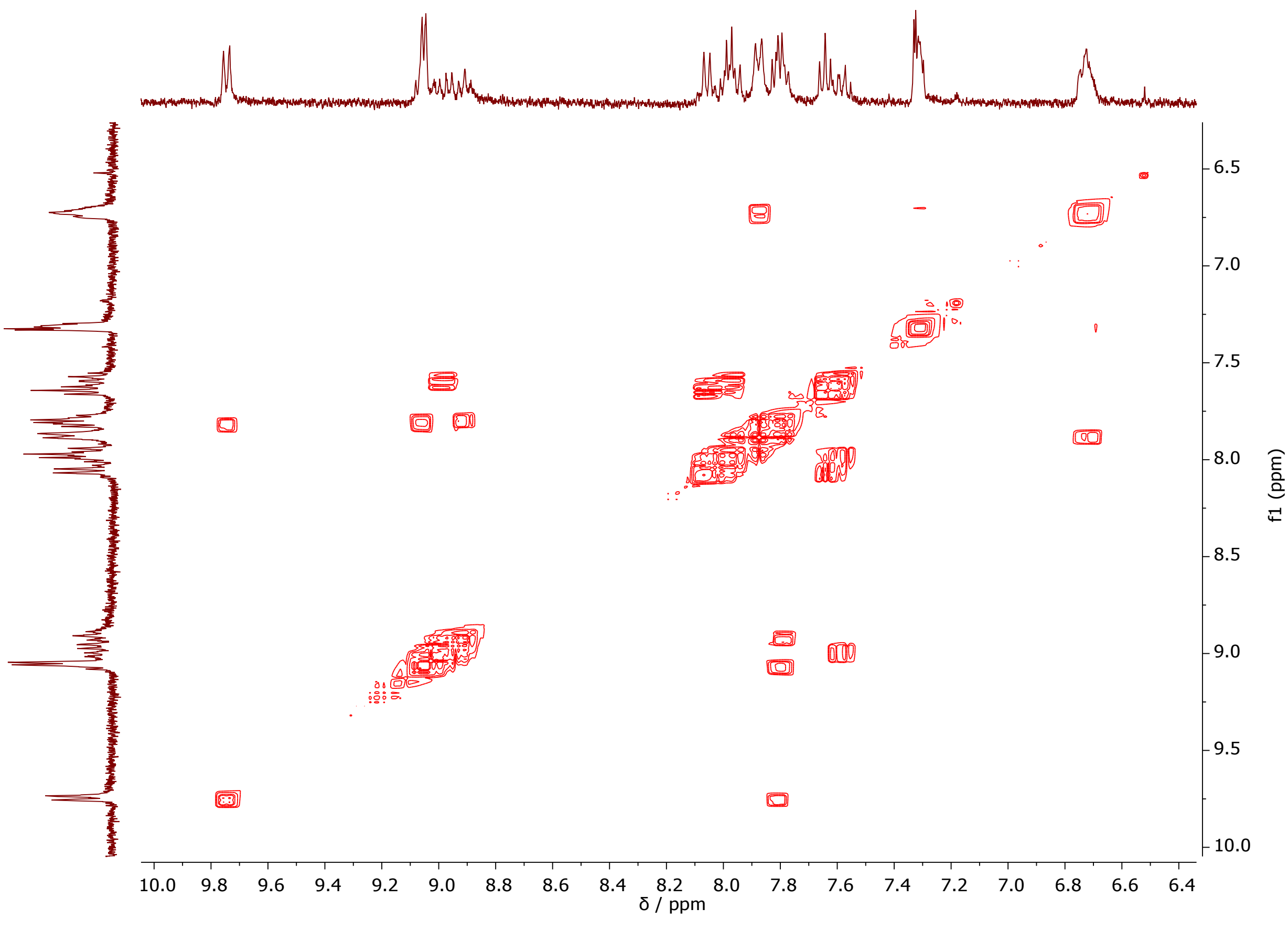

Figure S37. ^{1}H-^{1}H COSY (400 MHz, DMSO-d_6, 298 K) spectrum of **C5**.

2. MS Characterization

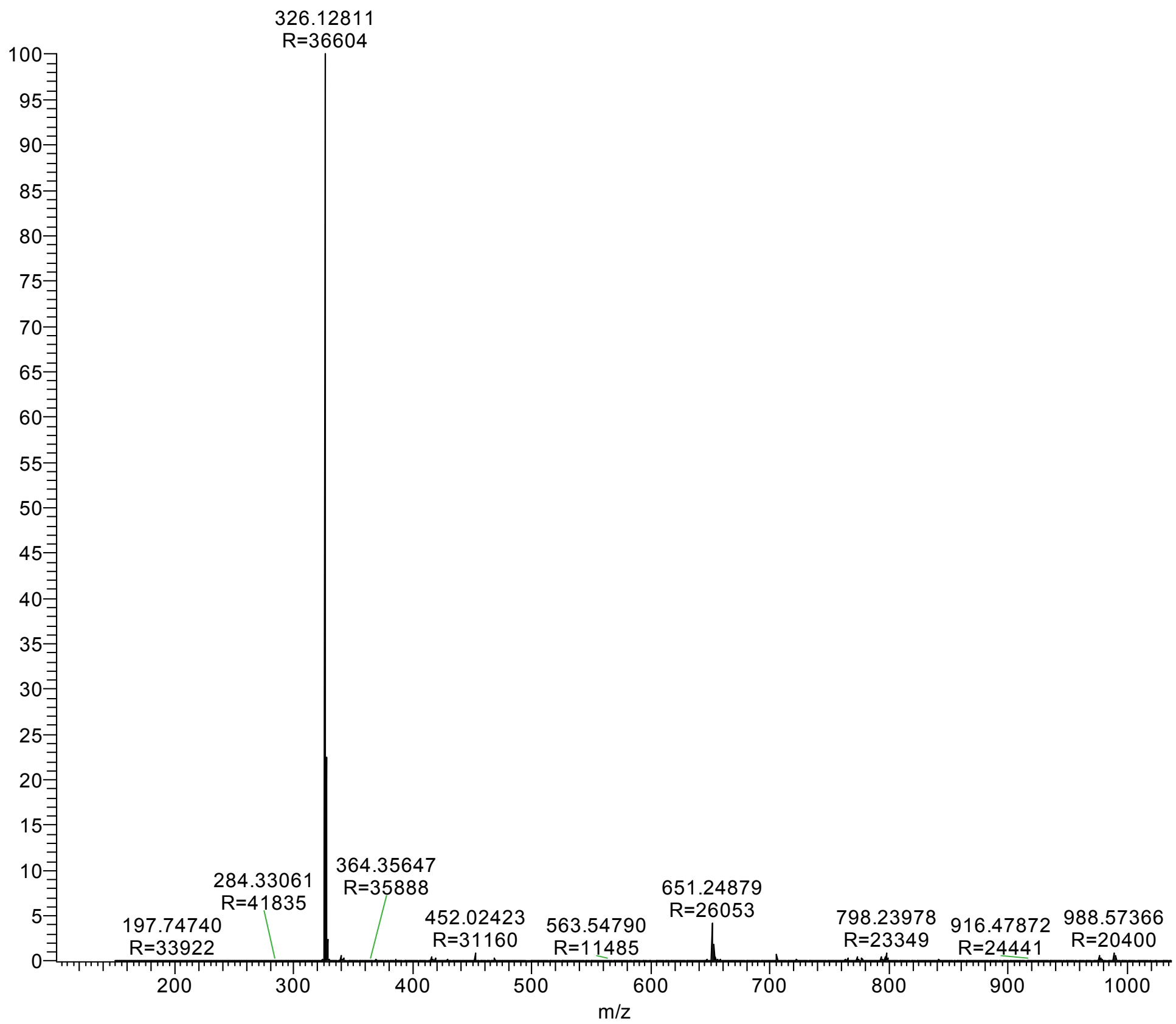

Figure S38. ESI+ mass of proligand **H-L1**.

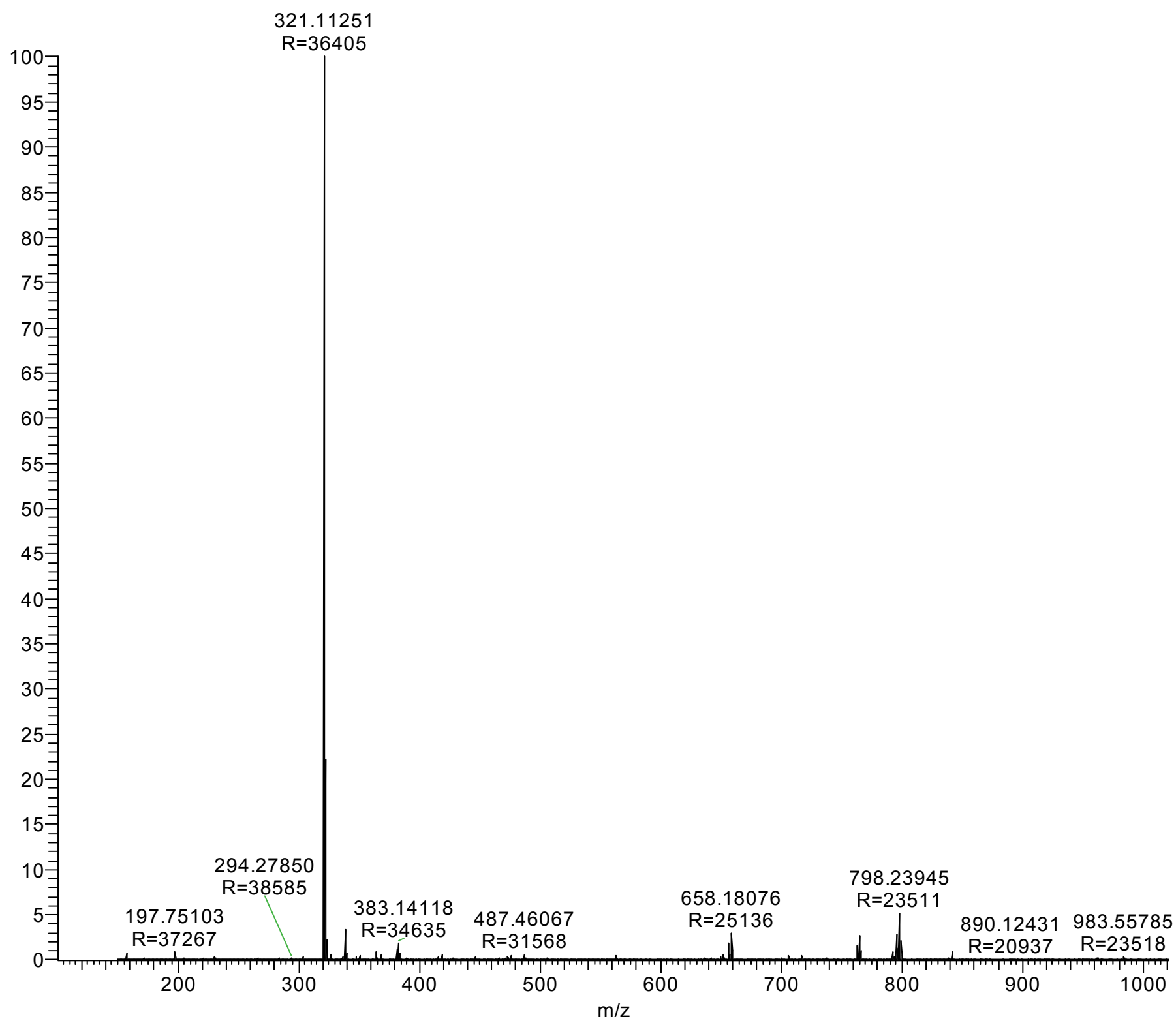

Figure S39. ESI+ mass of proligand **H-L2**.

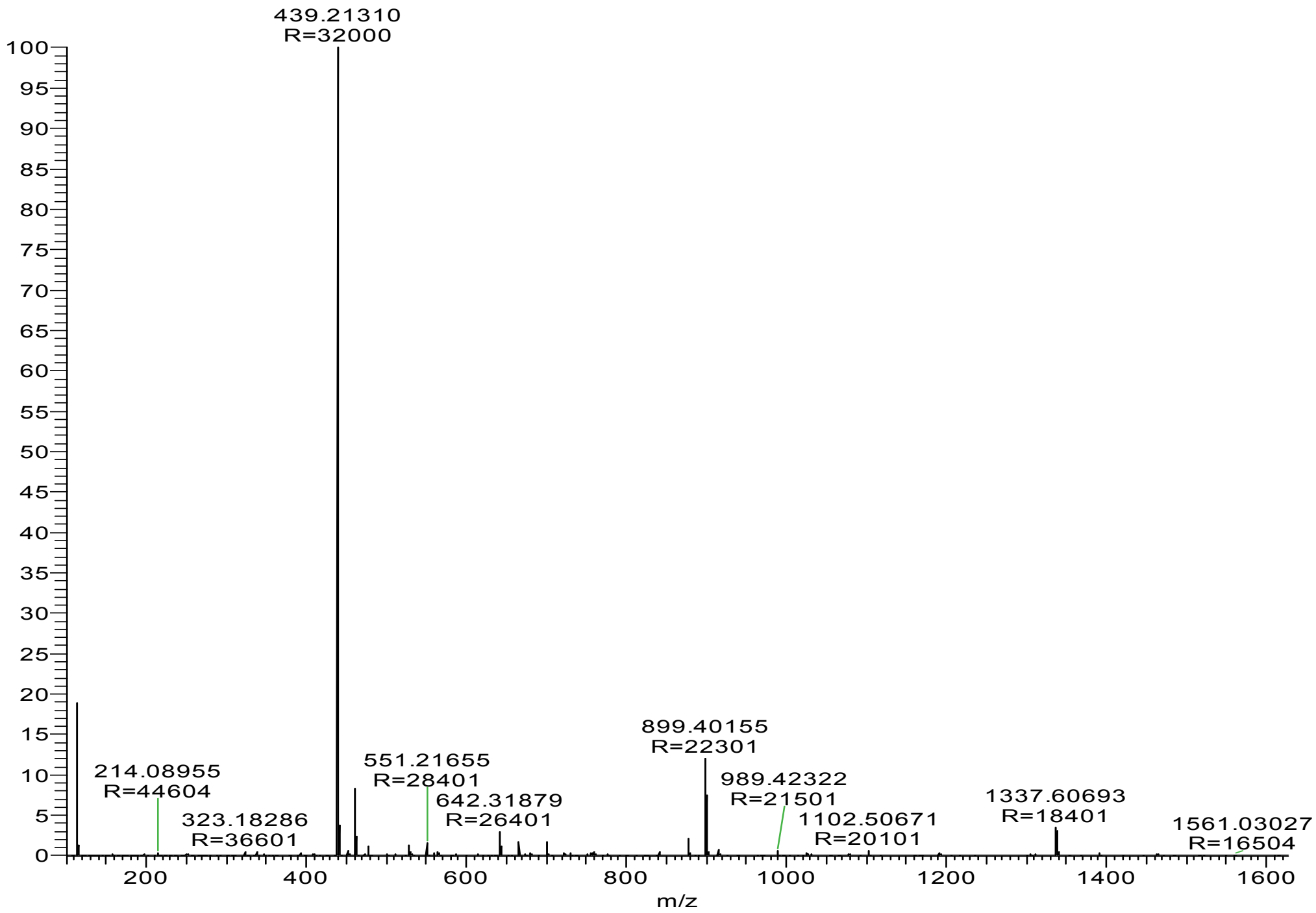

Figure S40. ESI+ mass of proligand **H-L3**.

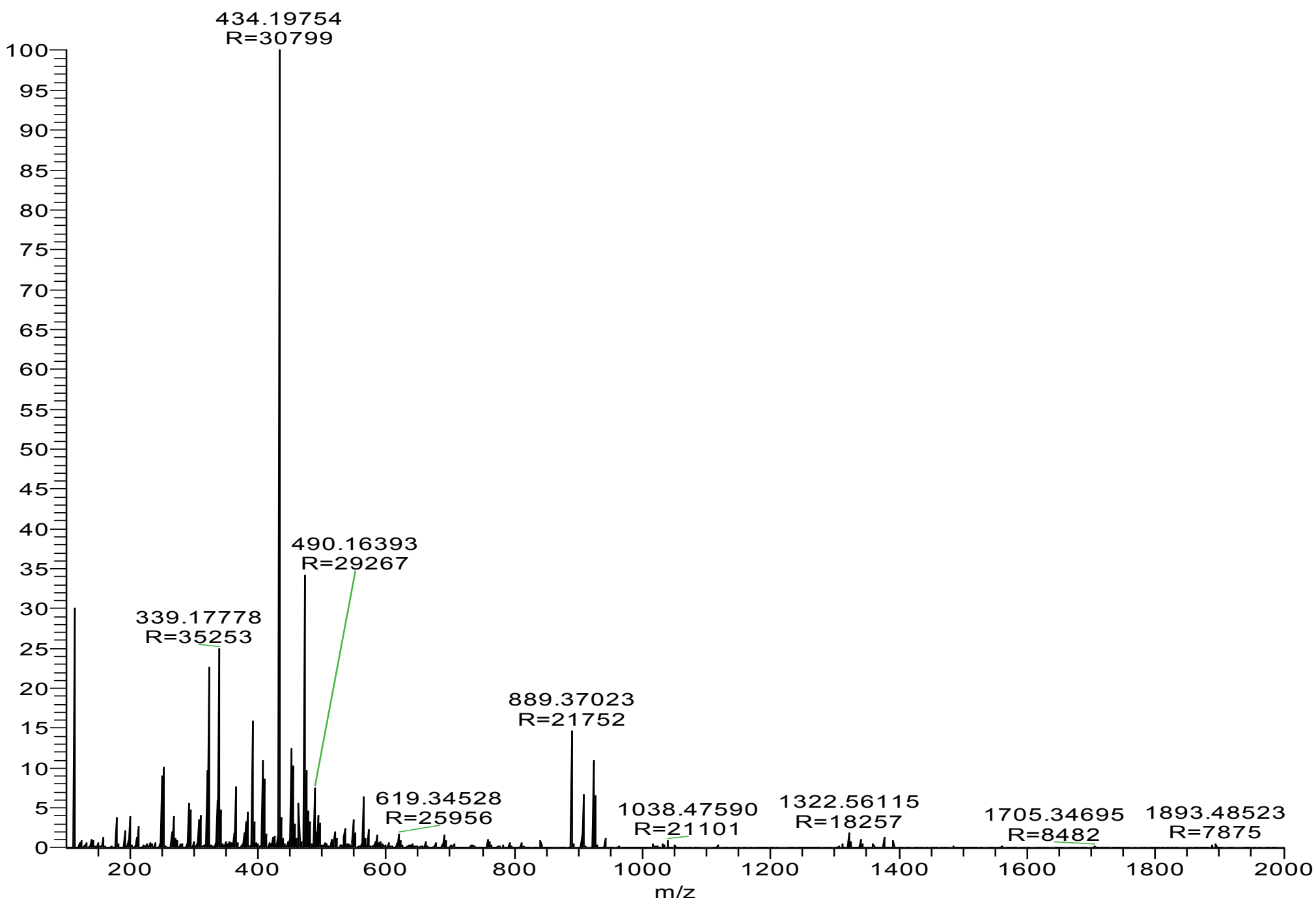

Figure S41. ESI+ mass of proligand **H-L4**.

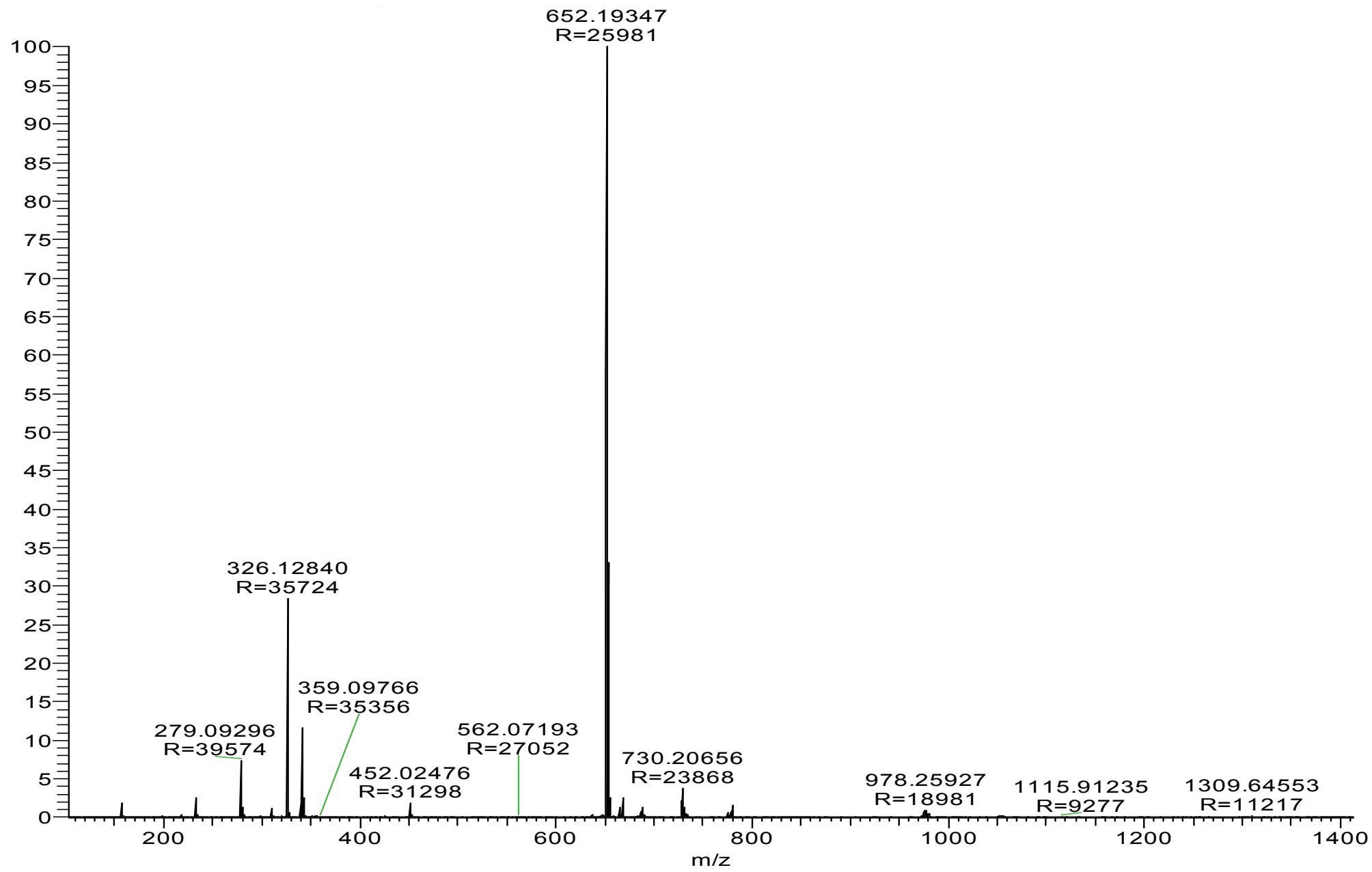

Figure S42. APCI+ mass of complex **C1**.

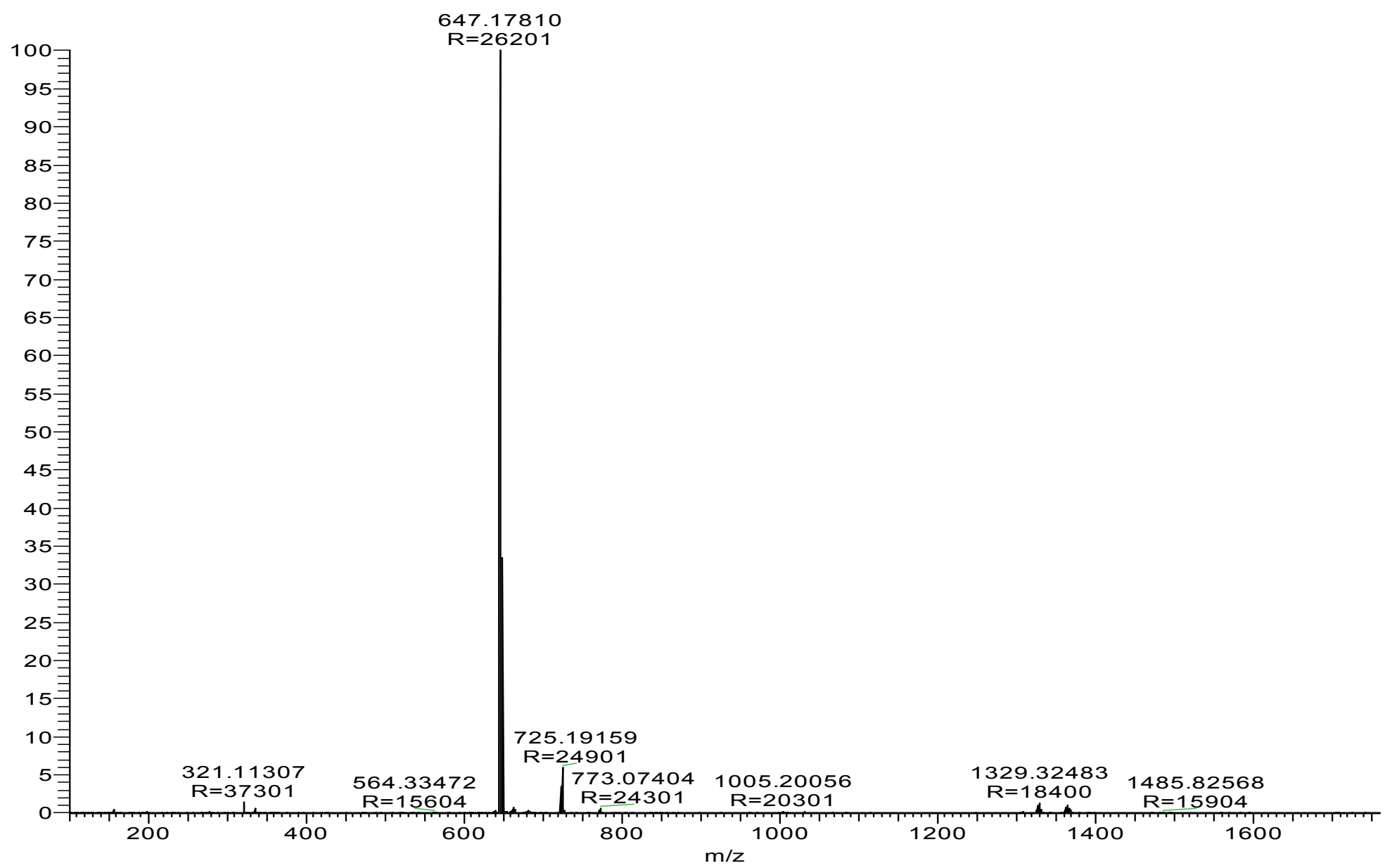

Figure S43. APCI+ mass of complex **C2**.

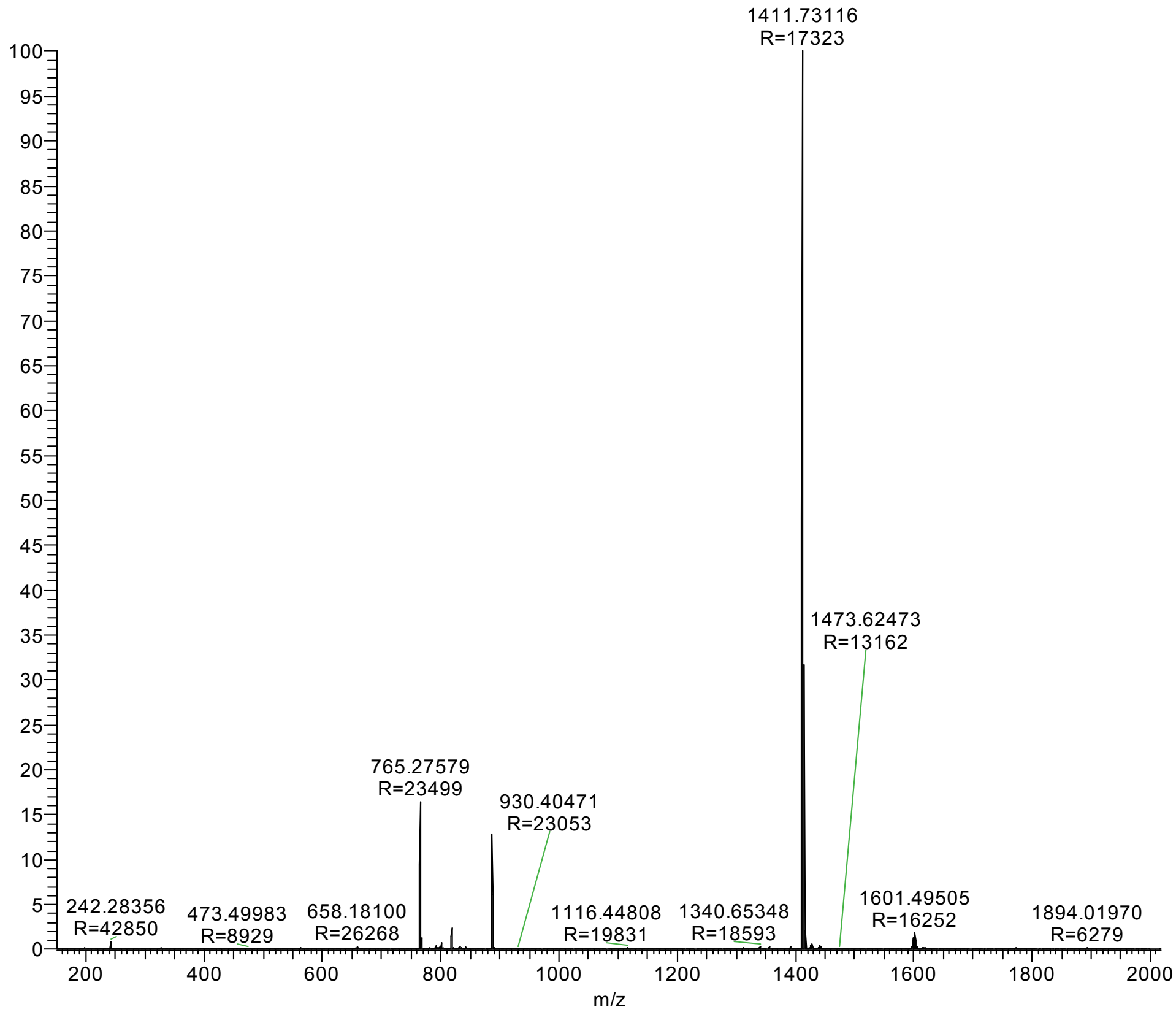

Figure S44. ESI+ mass of complex **C3**.

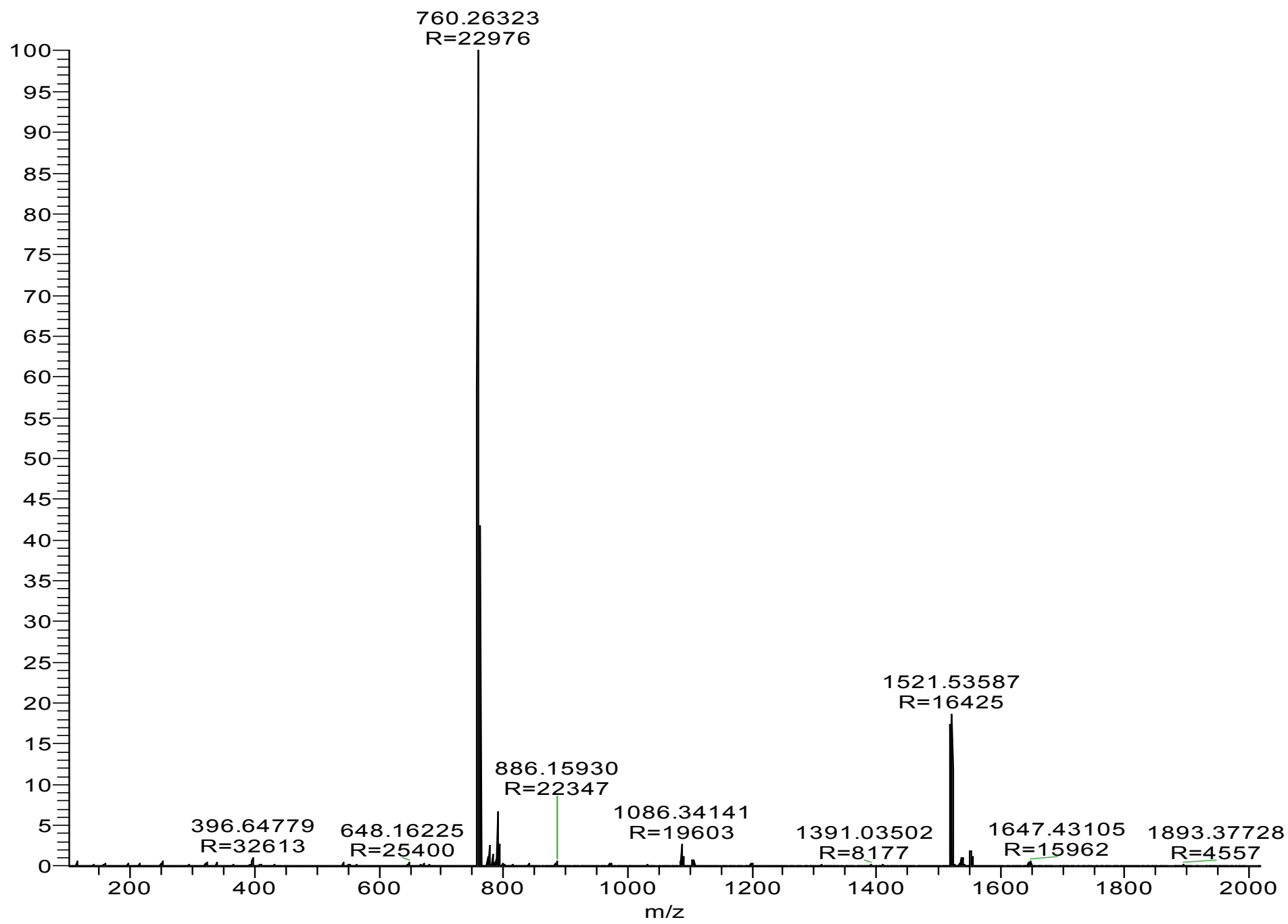

Figure S45. ESI+ mass of complex **C4**.

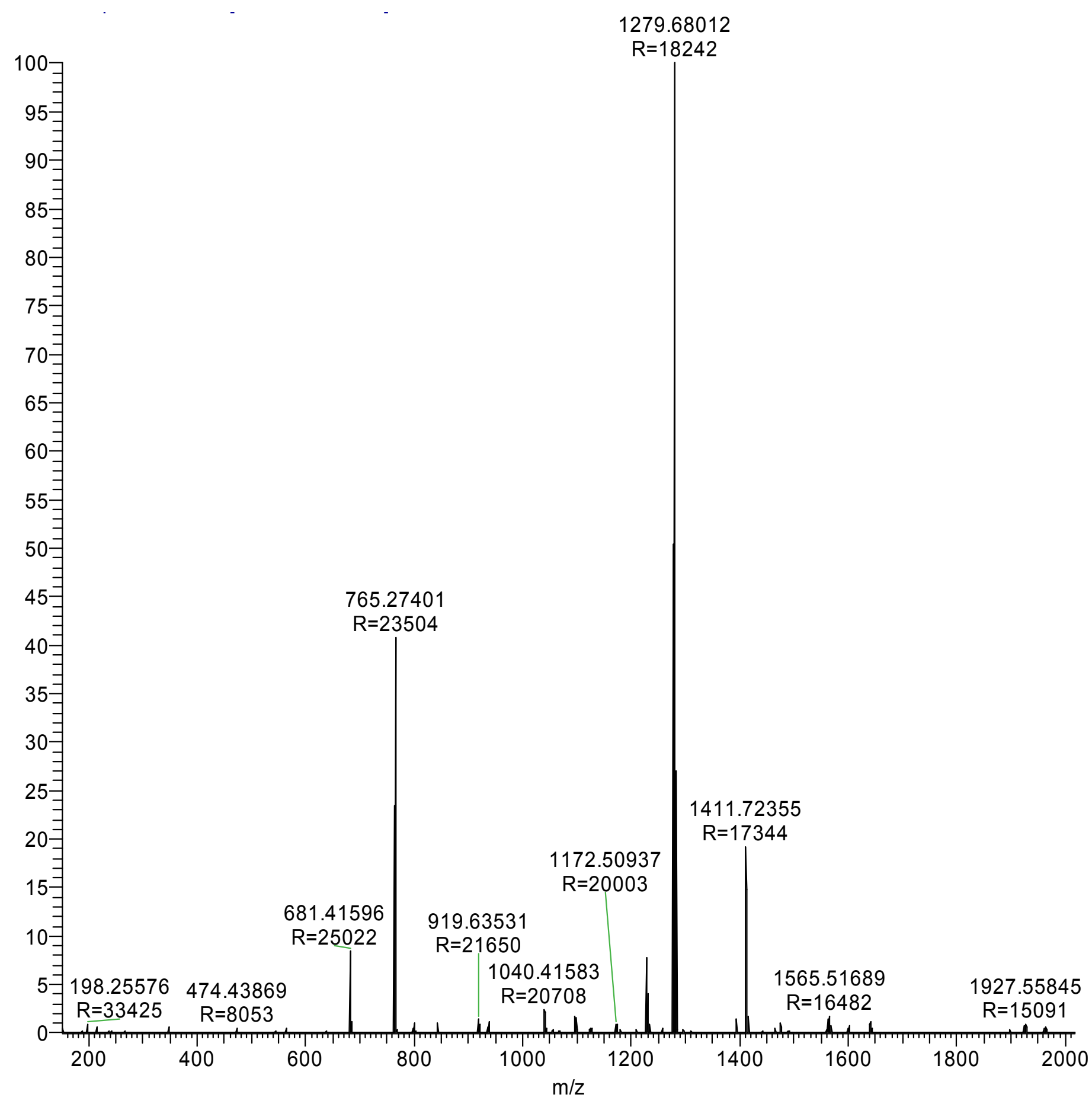

Figure S46. ESI+ mass of complex **C5**.

3. X-ray Difraction

Table S1. Crystal data and structure refinement for complexes **C1**, **C3**, **C4** and **C5**.

	**C1**x0.4CH_2Cl_2	**C3**x0.5CH_2Cl_2	**C4**x0.7$C_2H_4Cl_2$	**C5**x0.5H_2Ox0.25C_3H_6O
Empirical formula	$C_{31}H_{29}ClIrN_3O$	$C_{37.5}H_{41}Cl_2IrN_4O_2$	$C_{40}H_{39}ClF_3IrN_5O_3$	$C_{47.75}H_{56.5}Cl_2Ir_2N_4O_{2.75}$
Formula weight	687.22	842.84	922.41	1185.77
Temperature (K)	296.15	296.15	298.15	293(2)
Wavelength (Å)	0.71073	0.71073	0.71073	0.71073
Crystal system	Monoclinic	Orthorhombic	Triclinic	Monoclinic
Space group	$P2_1/c$	Pbca	$P\overline{1}$	C2/c
a(Å)	10.637(3)	23.427(4)	8.5385(5)	29.521(4)
b(Å)	20.264(5)	8.6400(14)	13.6395(8)	12.2386(14)
c(Å)	13.119(3)	35.775(6)	17.1290(10)	26.394(3)
α(°)	90	90	72.274(2)	90
β(°)	110.454(3)	90	82.507(2)	103.347(4)
γ(°)	90	90	82.469(2)	90
Volume($Å^3$)	2649.7(11)	7241(2)	1875.09(19)	9278(2)
Z	4	8	2	8
Density (calculated) (g/cm^3)	1.723	1.546	1.634	1.698
Absorption coefficient (mm^{-1})	5.169	3.873	3.692	5.890
F(000)	1352.0	3368.0	918.0	4648.0
Crystal size (mm^3)	0.28 × 0.11 × 0.09	0.36 × 0.28 × 0.22	0.12 × 0.07 × 0.03	0.17×0.1×0.05
Index ranges	-13 ≤ h ≤ 13, -25 ≤ k ≤ 23, -16 ≤ l ≤ 16	-26 ≤ h ≤ 27, -10 ≤ k ≤ 10, -40 ≤ l ≤ 42	-10 ≤ h ≤ 10, -16 ≤ k ≤ 16, -20 ≤ l ≤ 16	-35 ≤ h ≤ 21 -14 ≤ k ≤ 14 -31 ≤ l ≤ 31
Reflections collected	18897	42718	20588	17868
Independent reflections	5416 [R_{int} = 0.0864, R_{sigma} = 0.0898]	6374 [R_{int} = 0.1240, R_{sigma} = 0.0790]	6600 [R_{int} = 0.0595, R_{sigma} = 0.0803]	8037 [R_{int} = 0.0872]
Data / restraints / parameters	5416/18/354	6374/0/439	6600/4/491	8037/639/533
Goodness-of-fit on F^2 *c*	1.020	1.047	1.028	0.983
Final R indexes *a,b* [$I>2\sigma(I)$]	R_1 = 0.0548, wR_2 = 0.1175	R_1 = 0.0623, wR_2 = 0.1330	R_1 = 0.0511, wR_2 = 0.0773	R_1 = 0.0717 wR_2 = 0.1440
Final R indexes *a,b* (all data)	R_1 = 0.1008, wR_2 = 0.1439	R_1 = 0.1111, wR_2 = 0.1556	R_1 = 0.0837, wR_2 = 0.0892	R_1 = 0.1668 wR_2 = 0.1865

Largest diff. peak / hole, e.Å^{-3}	1.00/-1.73	1.57 / -0.84	0.69/-1.04	1.71/-1.37

^{a} $R = \Sigma||F_o|–|F_c|/\Sigma|F_o|$. ^{b} $wR = \{\Sigma w(F_o^2–F_c^2)^2/\Sigma w(F_o^2)^2\}^{1/2}$. ^{c} GOF = $\{\Sigma [w((F_o^2–F_c^2)^2)/(n-p)\}^{1/2}$, where n = number of reflections and p = total number of parameters refined.

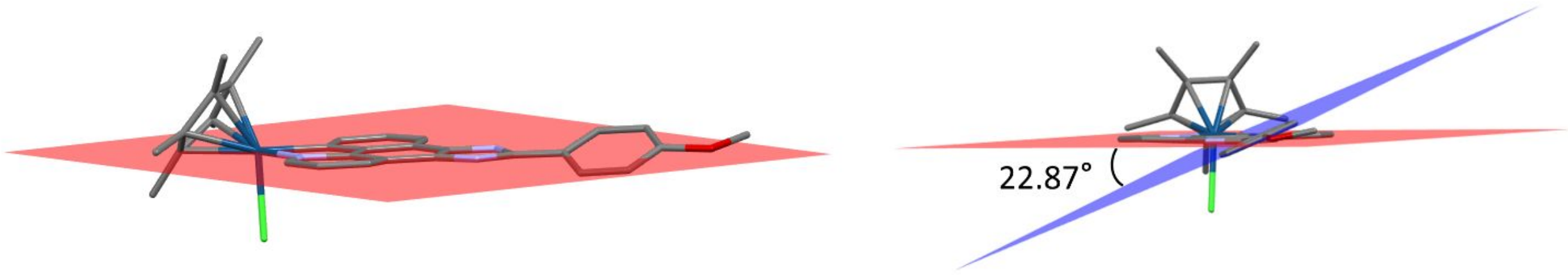

Figure S47. Deviation of the planarity of the C^N ligand in the solid structure of complex **C1**.

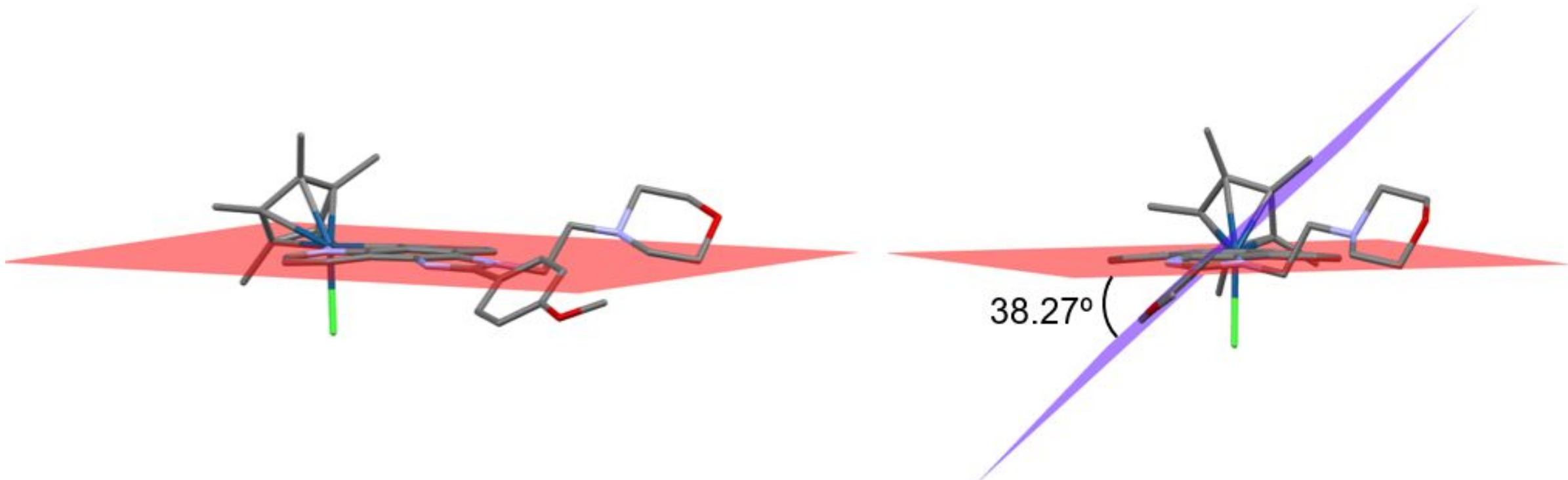

Figure S48. Deviation of the planarity of the C^N ligand in the solid structure of complex **C3**.

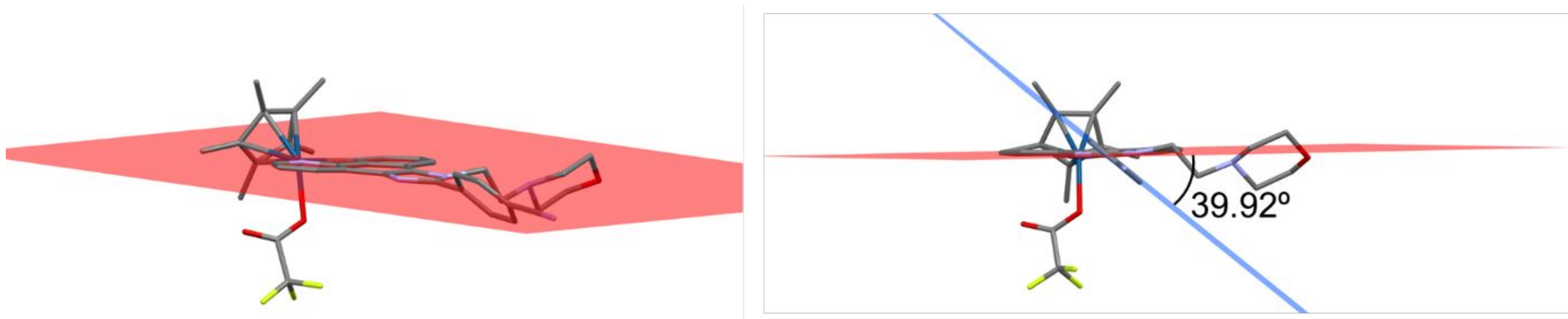

Figure S49. Deviation of the planarity of the C^N ligand in the solid structure of complex **C4**.

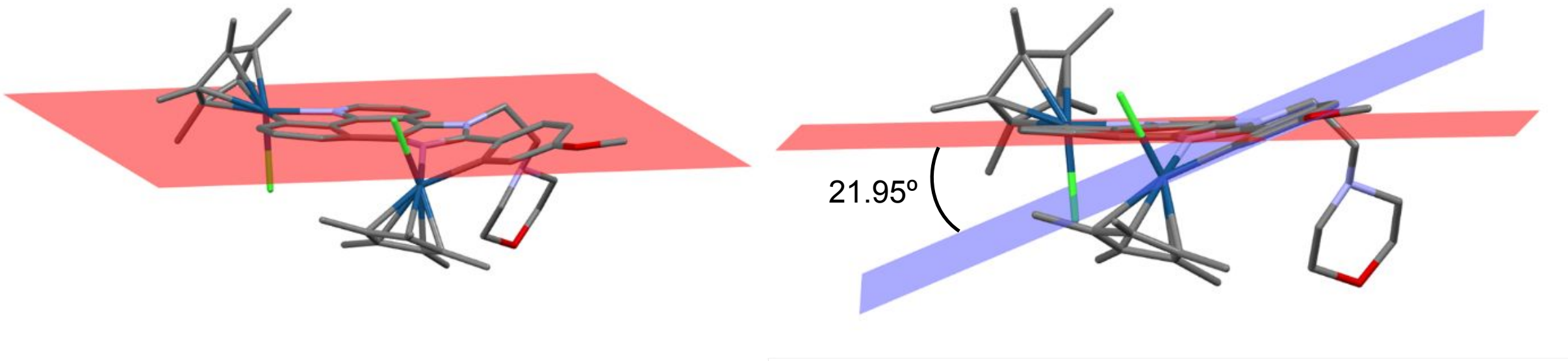

Figure S50. Deviation of the planarity of the C^N ligand in the solid structure of complex **C5**.

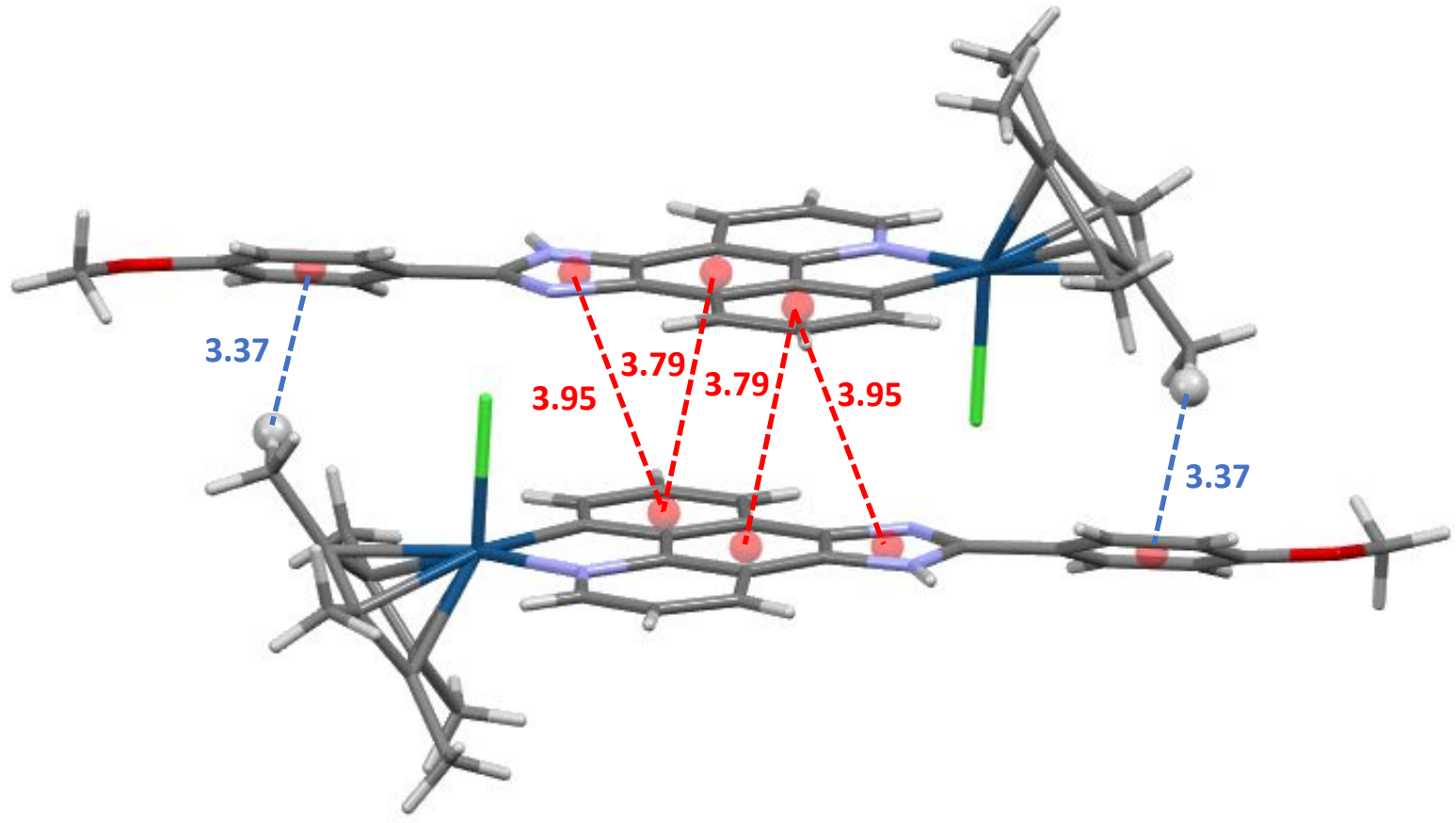

Figure S51. Box-like dimer of complex **C1** formed through π-π interactions (red, centroid–centroid distances indicated, Å) and CH-π interactions (blue, centroid-H distance indicated, Å). The H atoms that participate in CH-π interactions are marked as balls.

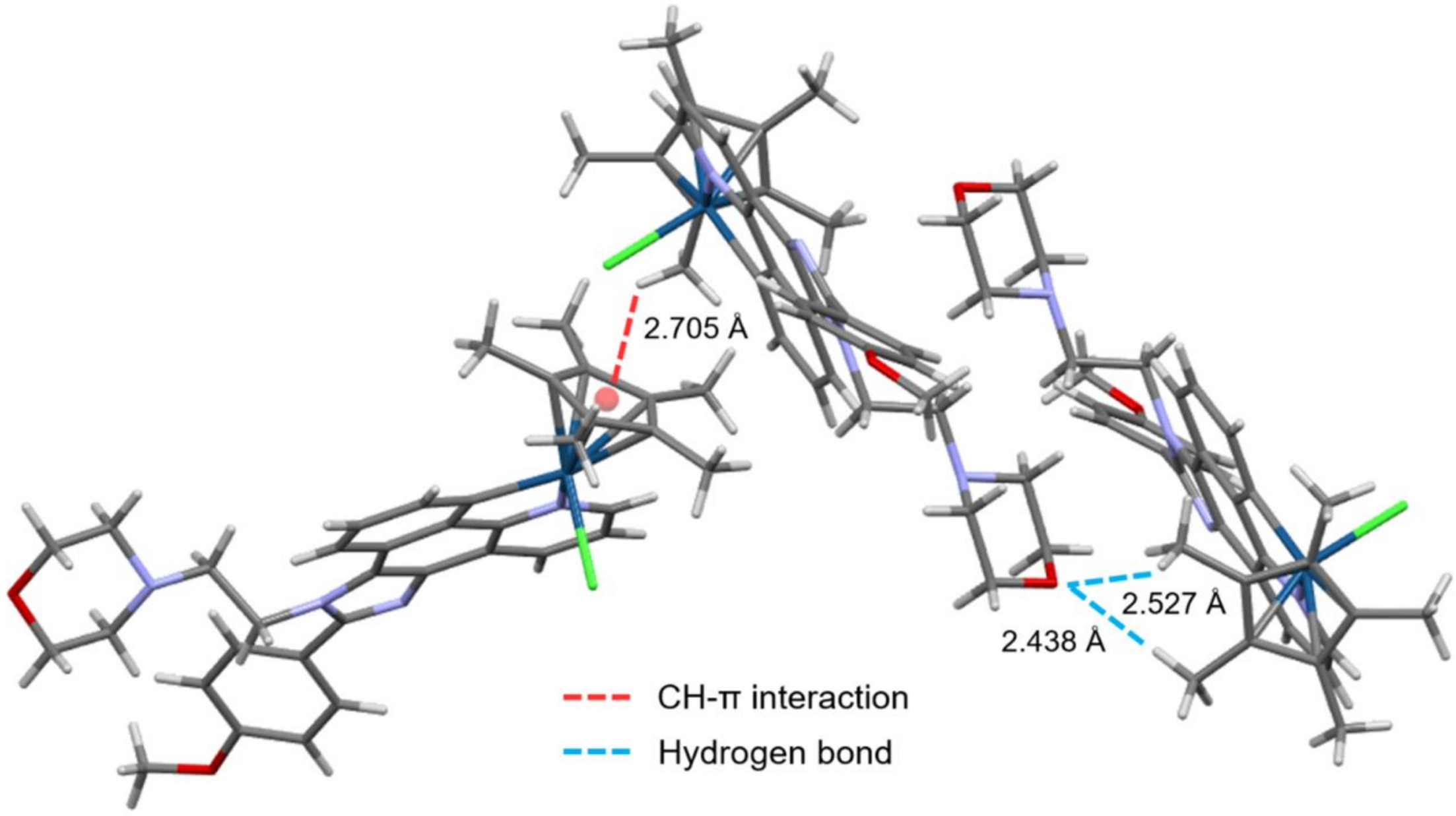

Figure S52. Intramolecular interactions of complex **C3** in the solid state. CH-π interactions (red, centroid–H distances indicated, Å) and hydrogen bonds (blue, H-X distances indicated, Å).

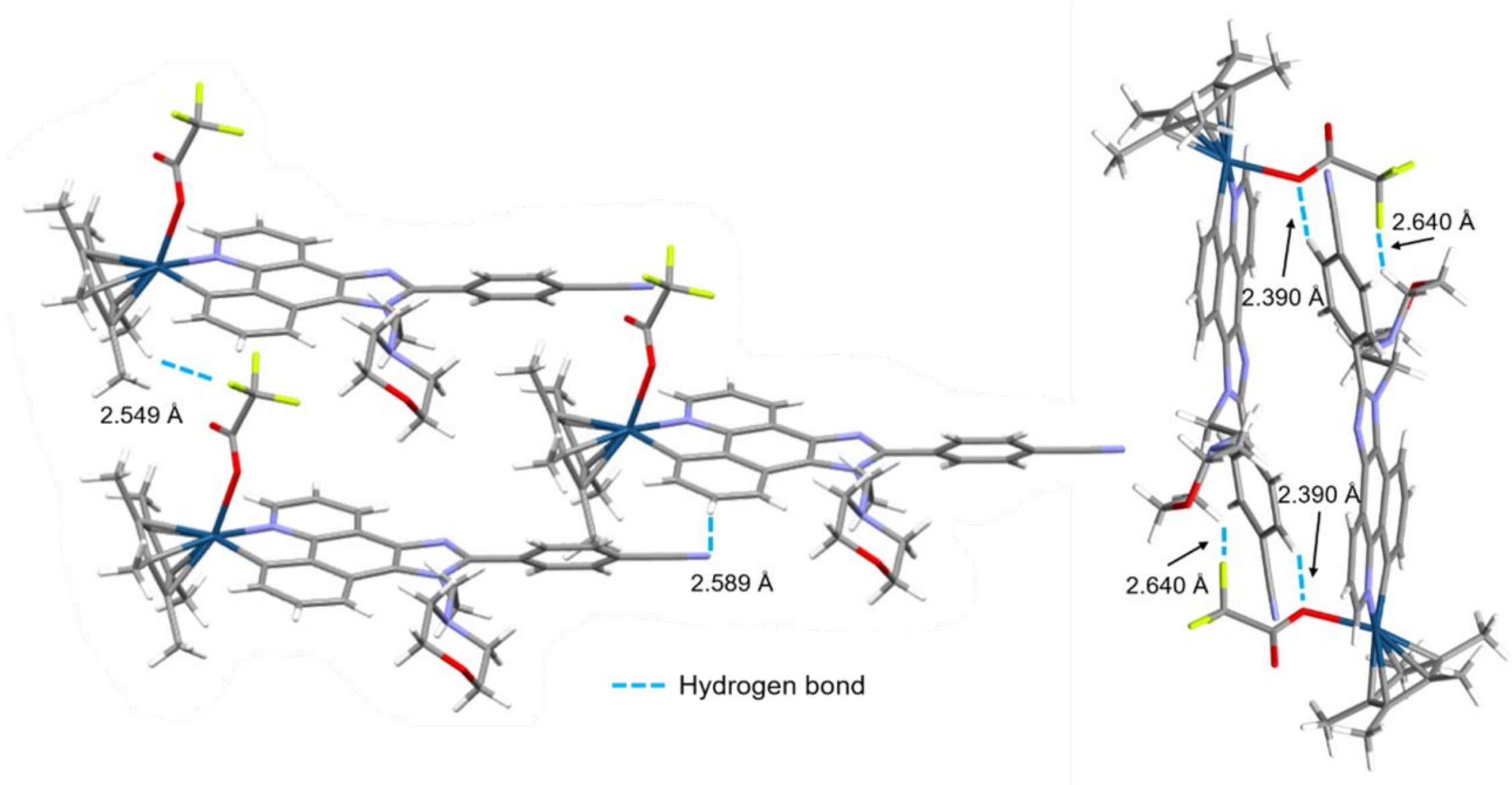

Figure S53. Intramolecular interactions of complex **C4** in the solid state. Hydrogen bonds (blue, H-X distances indicated, Å).

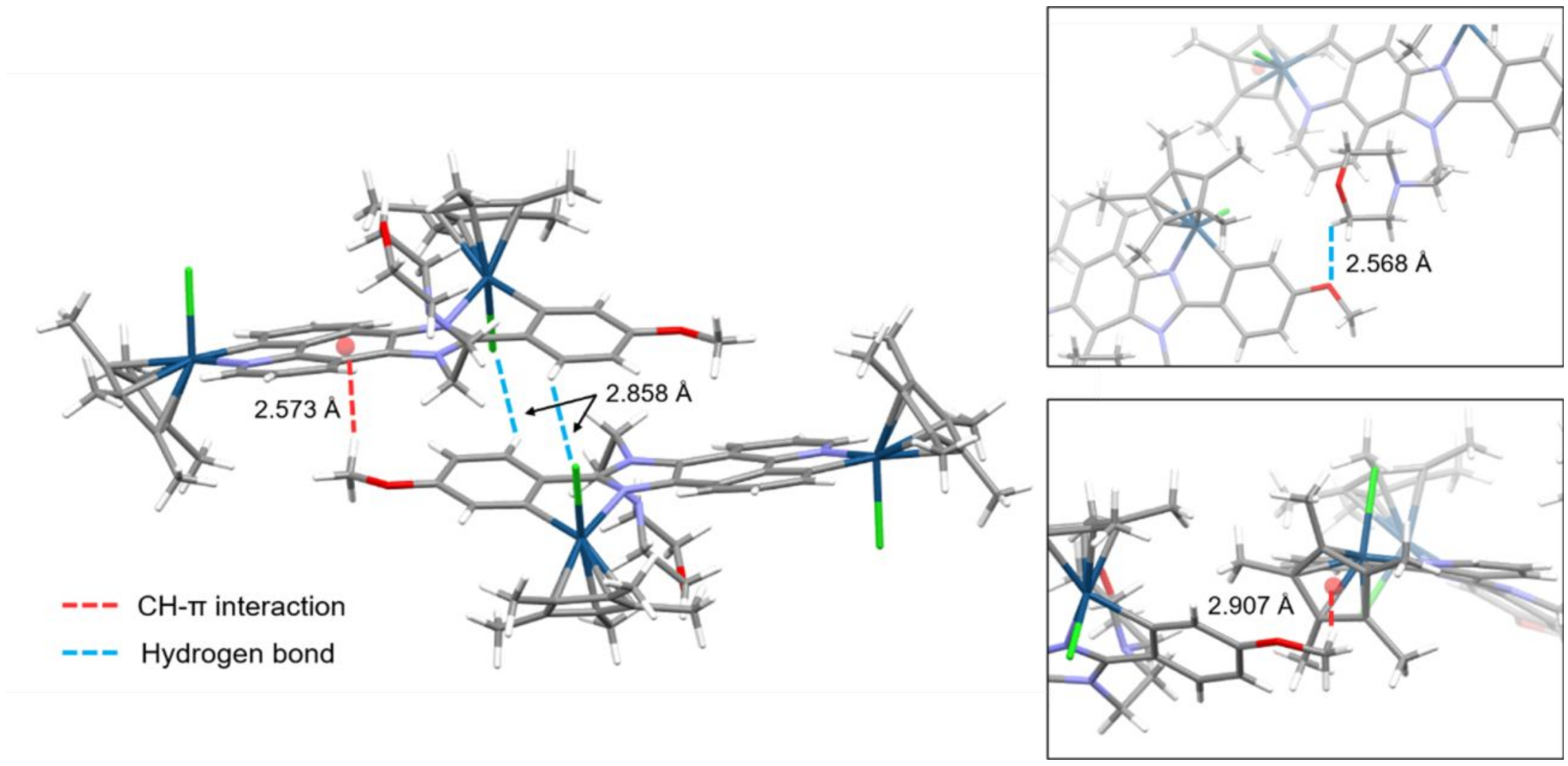

Figure S54. Intramolecular interactions of complex **C5** in the solid state. CH-π interactions (red, centroid–H distances indicated, Å) and hydrogen bonds (blue, H-X distances indicated, Å).

4. Stability in solution

Stability in DMSO-d_6:D_2O mixture.

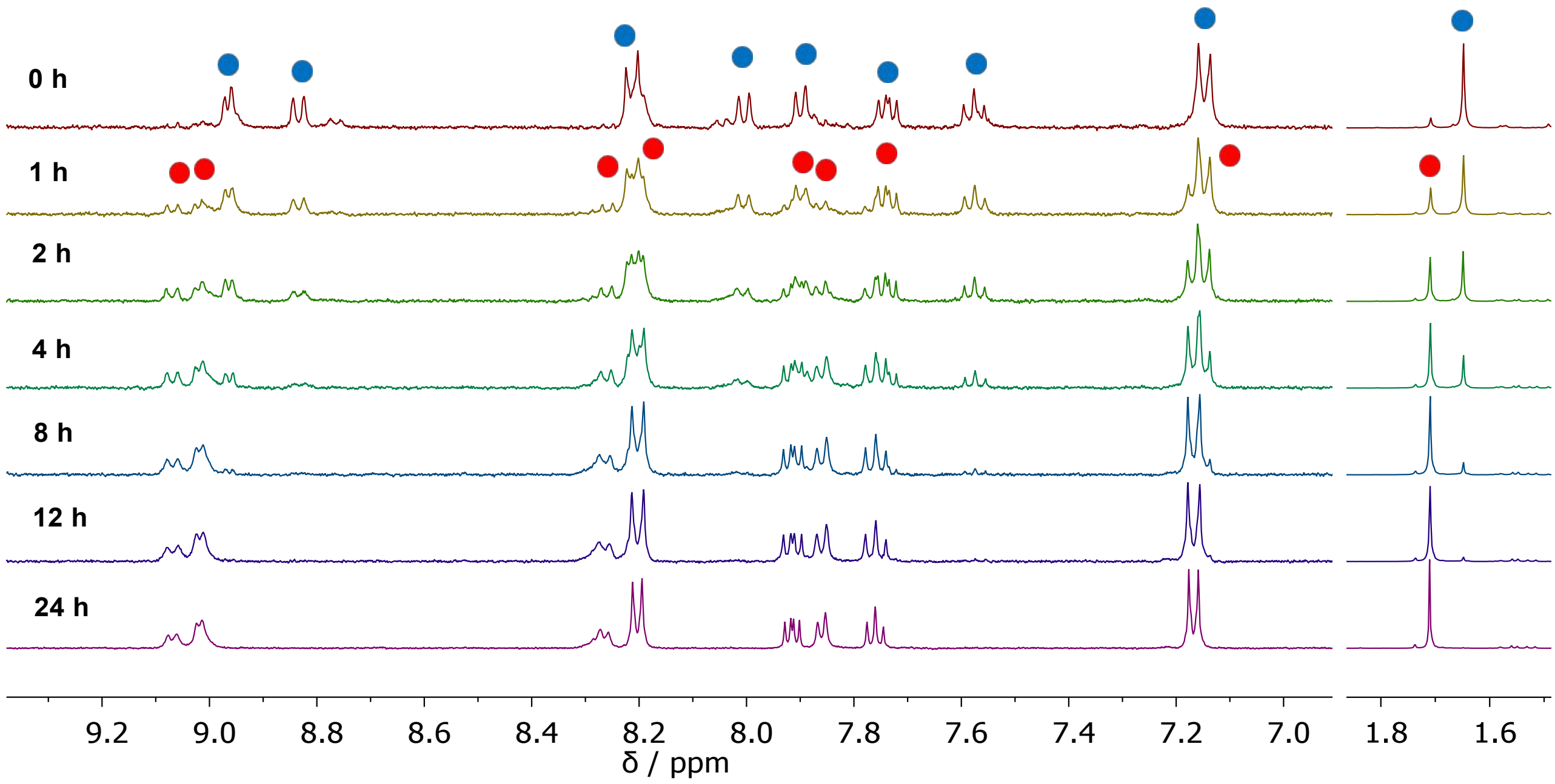

Figure S55. ^{1}H NMR evolution of complex **C1** in DMSO-d_6:D_2O (8:2). Blue circles correspond to the chlorido complex. Red circles correspond to the solvolysis product.

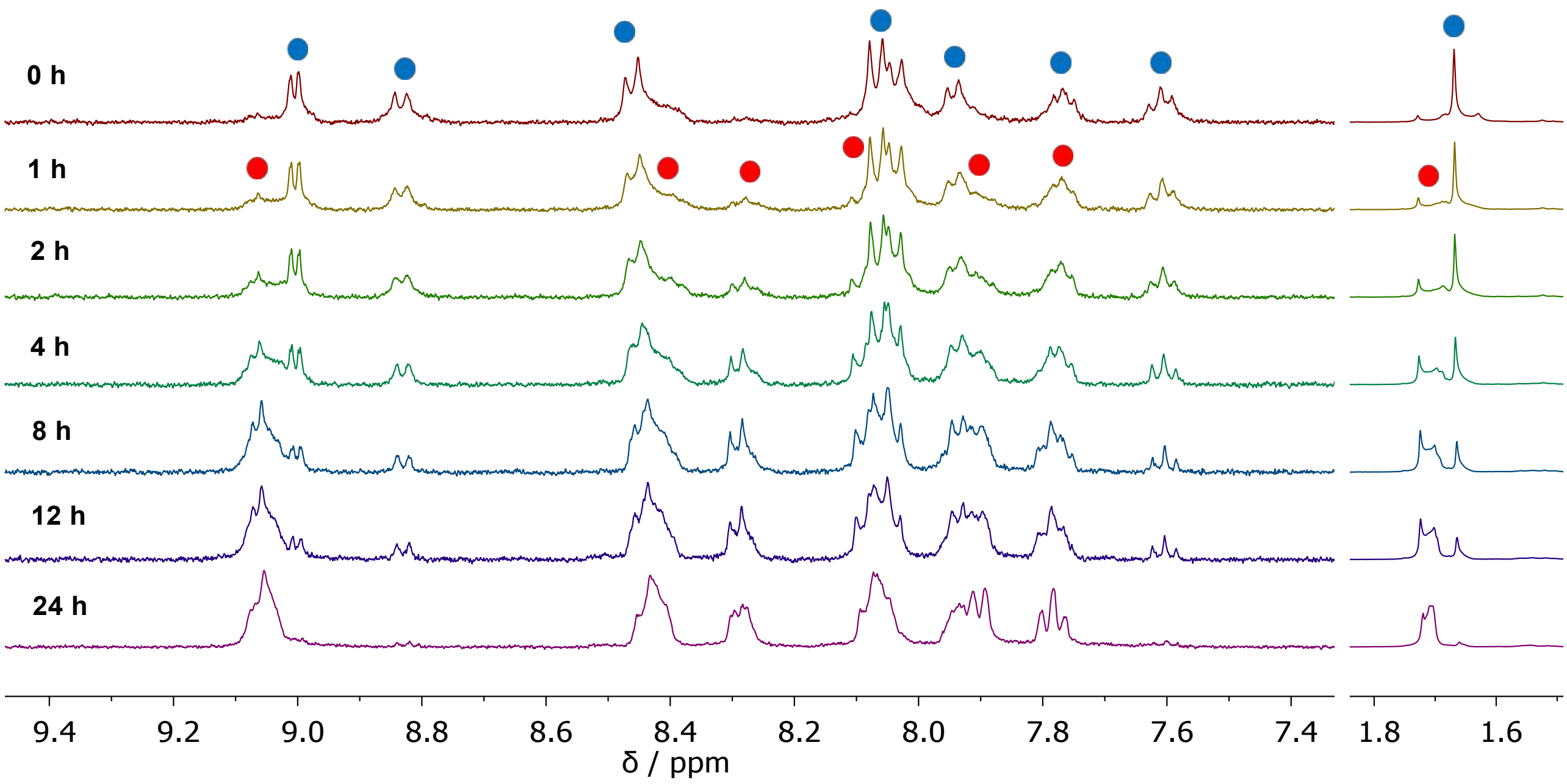

Figure S56. ^{1}H NMR evolution of complex **C2** in DMSO-d_6:D_2O (8:2). Blue circles correspond to the chlorido complex. Red circles correspond to the solvolysis product.

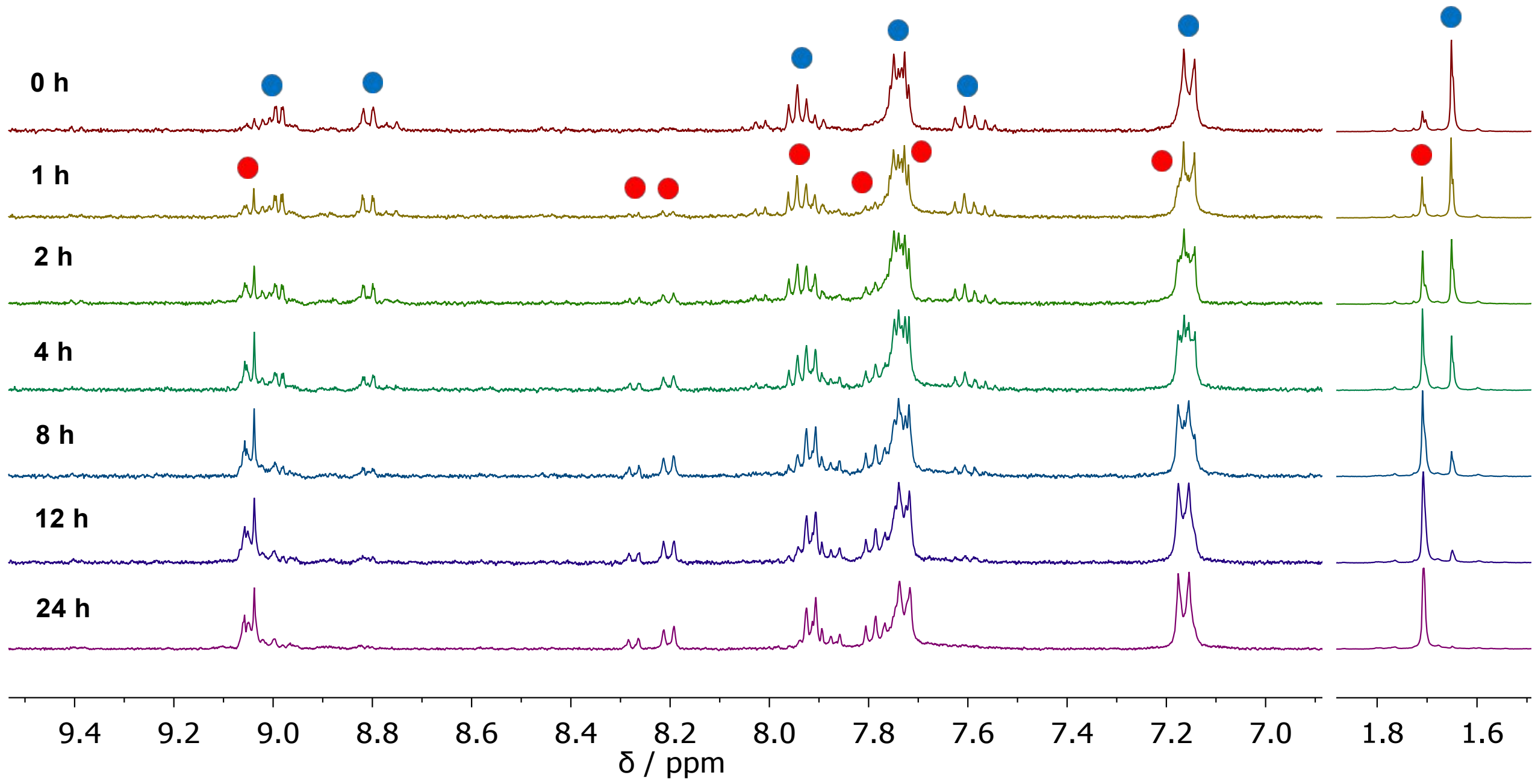

Figure S57. ^{1}H NMR evolution of complex **C3** in DMSO-d_6:D_2O (8:2). Blue circles correspond to the chlorido complex. Red circles correspond to the solvolysis product.

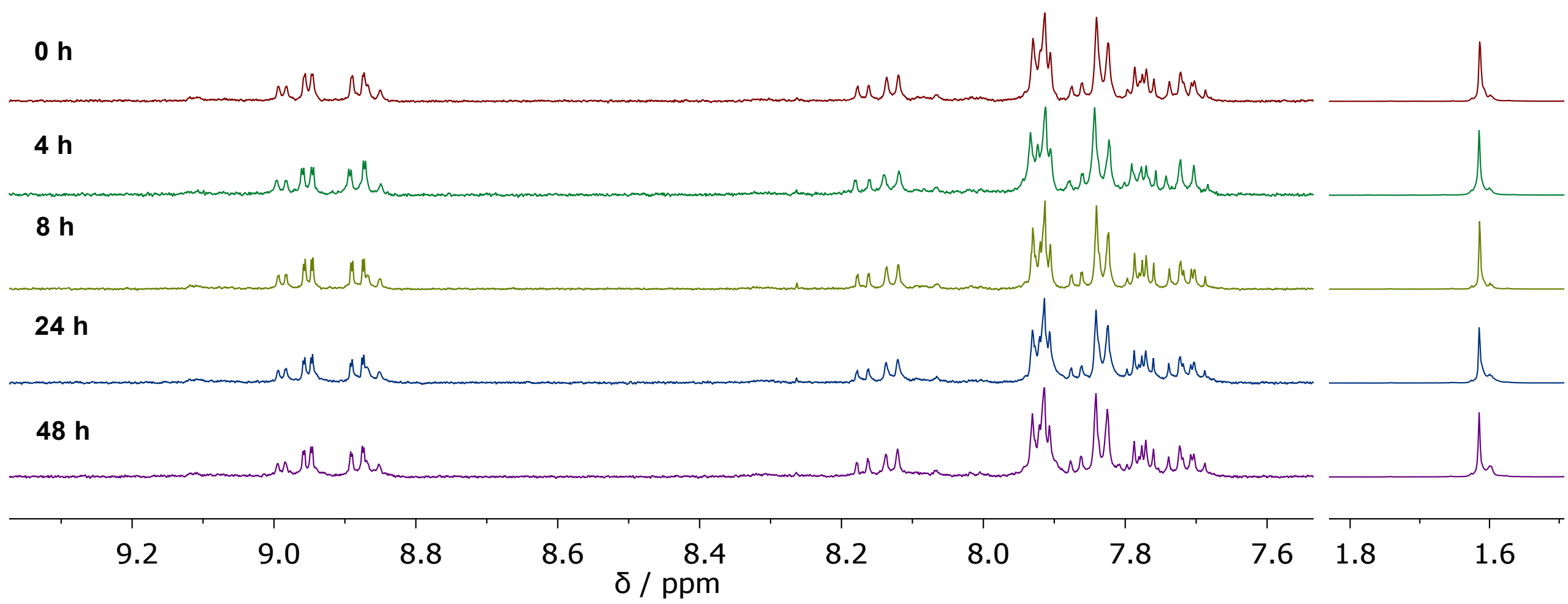

Figure S58. ^{1}H NMR evolution of complex **C4** in DMSO-d_6:D_2O (8:2).

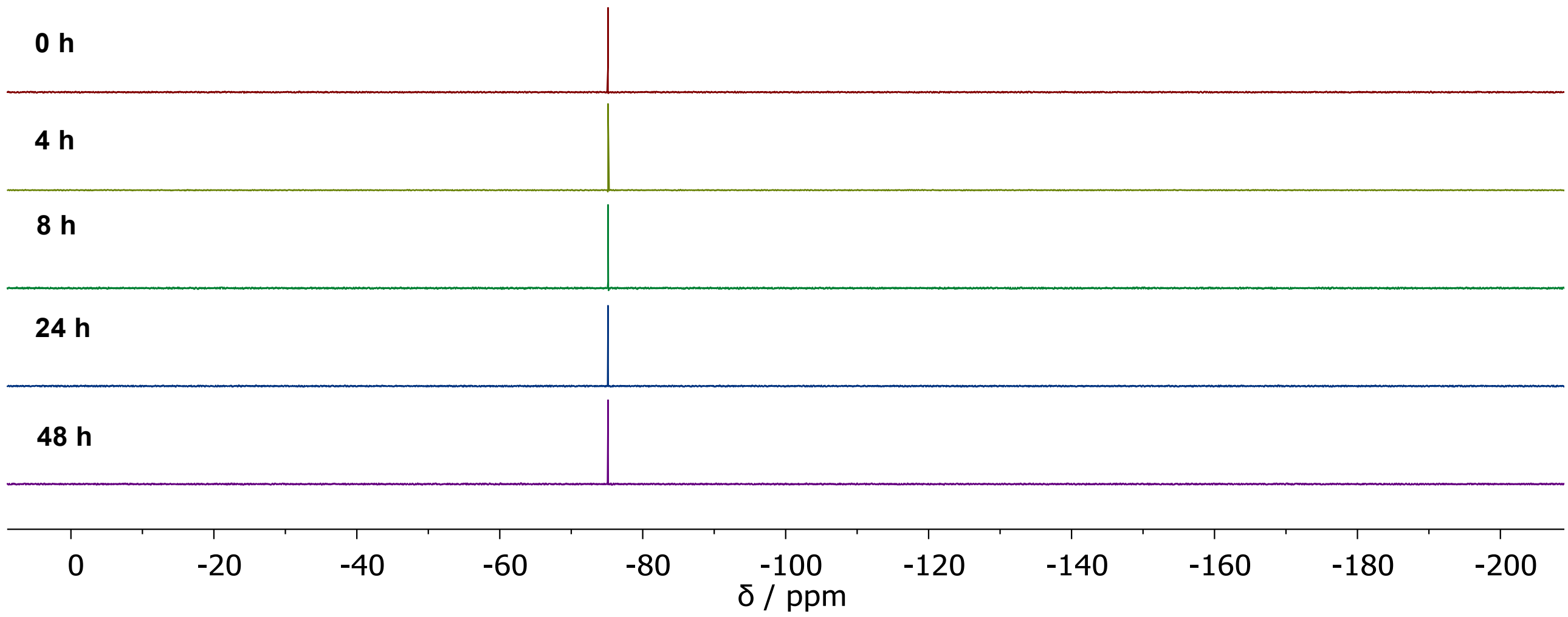

Figure S59. ^{19}F NMR evolution of complex **C4** in DMSO-*d*$_6$:D2O (8:2).

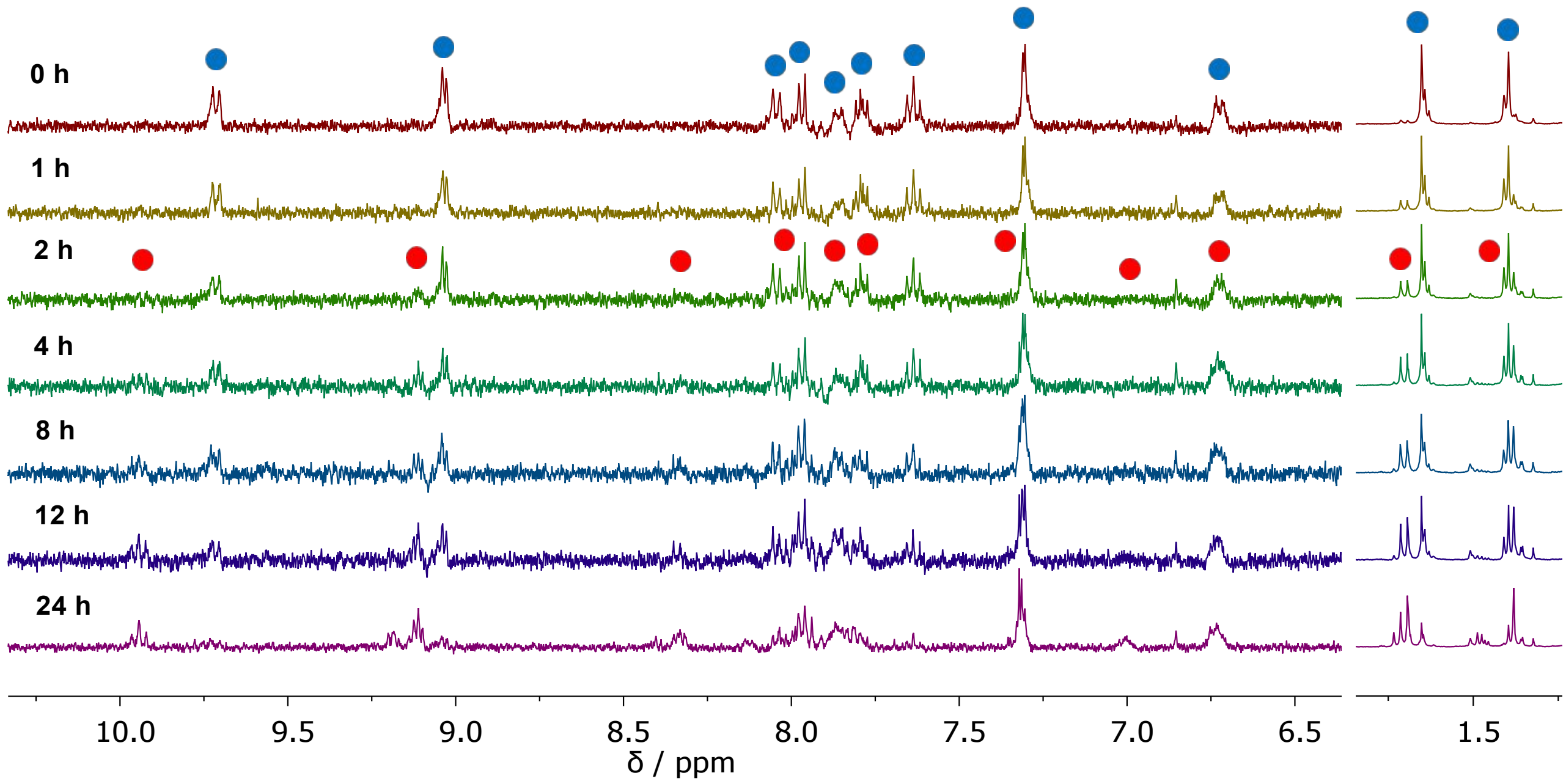

Figure S60.^{1}H NMR evolution of complex **C5** in DMSO-*d*$_6$:D_2O (8:2). Blue circles correspond to the chlorido complex. Red circles correspond to the solvolysis product.

Table S2. Solvolysis of the Ir(III) complexes in DMSO-d_6:D_2O (8:2) at room temperature.

	Starting product (%)				
**t (h)**	**C1**	**C2**	**C3**	**C4**	**C5**
0	94	97	84	100	100
0.5	80	90	78	100	99
1	69	84	73	100	95
1.5	59	78	66	100	91
2	53	71	59	100	89
4	31	50	45	100	72
8	12	26	22	100	48
12	0	17	0	100	36
24	0	6	0	100	14
48	0	0	0	100	0

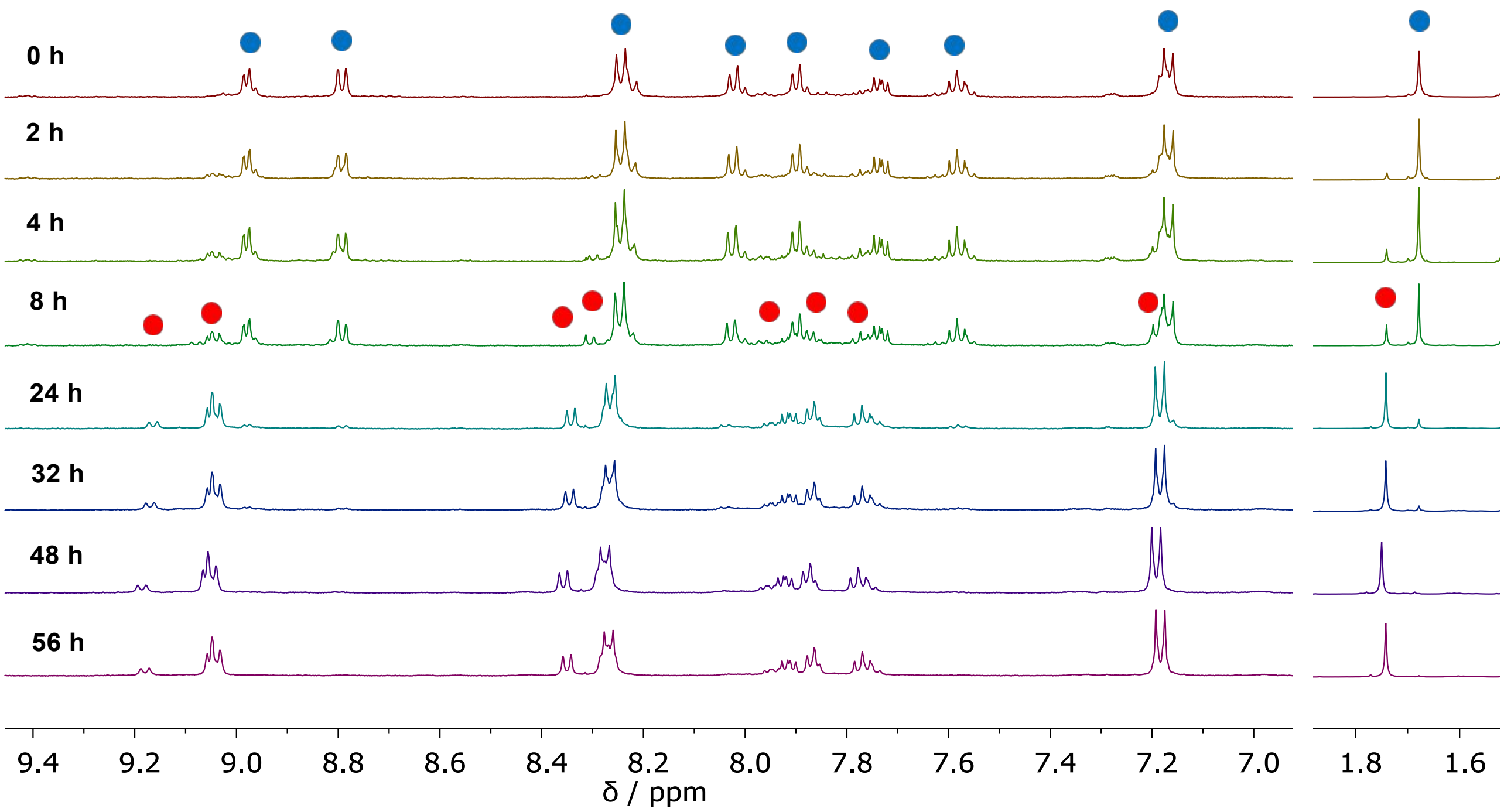

Figure S61. ^{1}H NMR evolution of complex **C1** in DMSO-d_6. Blue circles correspond to the chlorido complex. Red circles correspond to the solvolysis product.

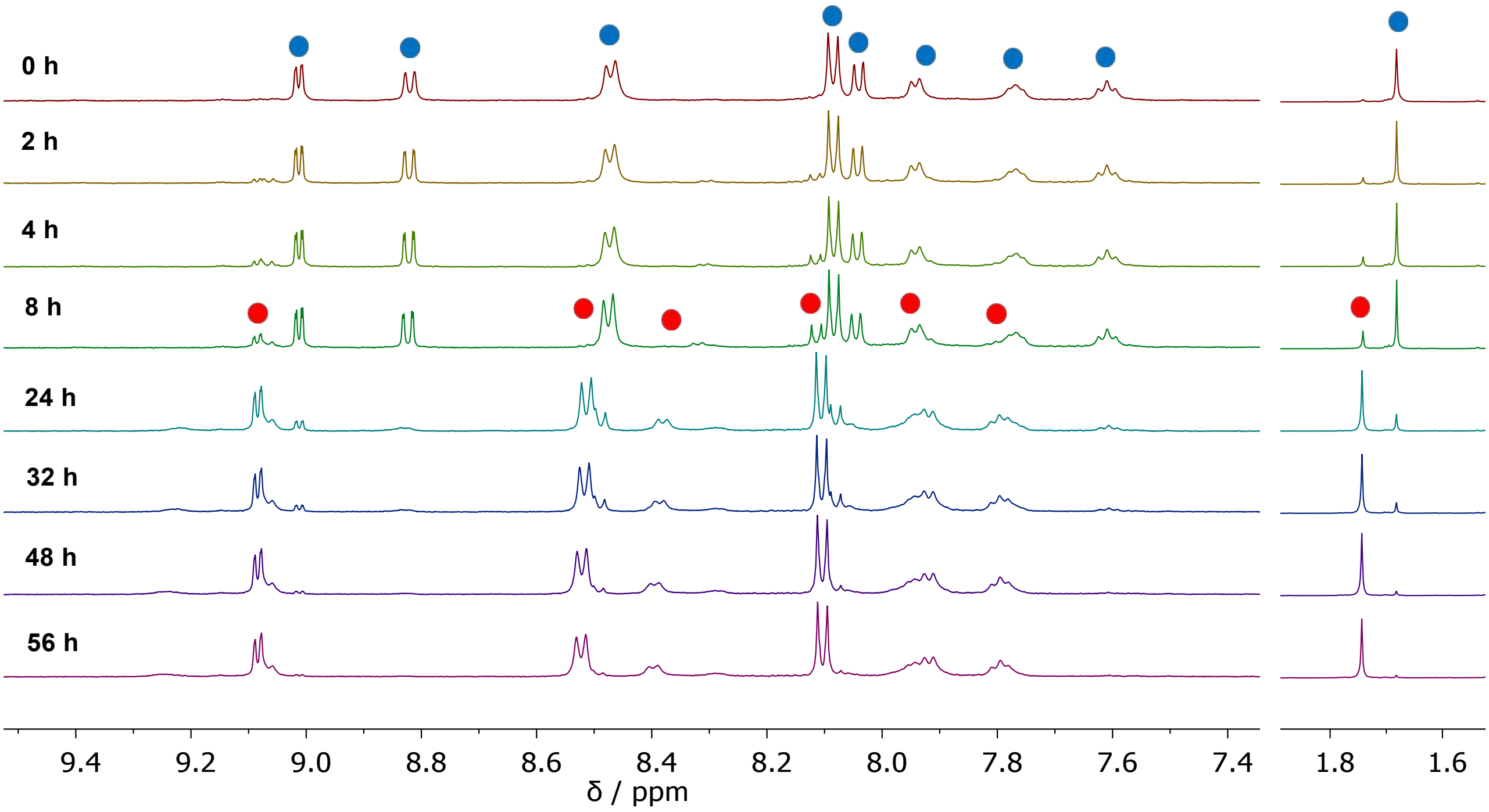

Figure S62. ^{1}H NMR evolution of complex **C2** in DMSO-d_6. Blue circles correspond to the chlorido complex. Red circles correspond to the solvolysis product.

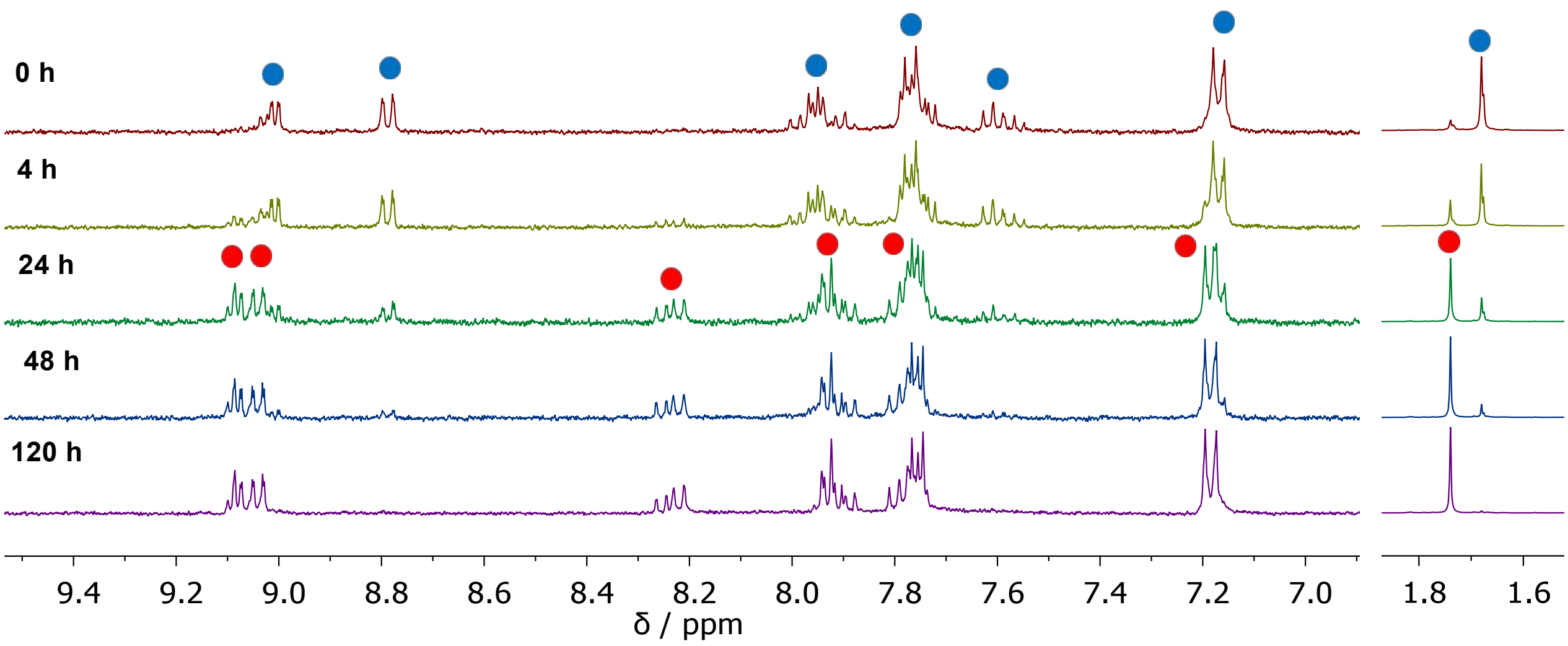

Figure S63. ^{1}H NMR evolution of complex **C3** in DMSO-d_6. Blue circles correspond to the chlorido complex. Red circles correspond to the solvolysis product.

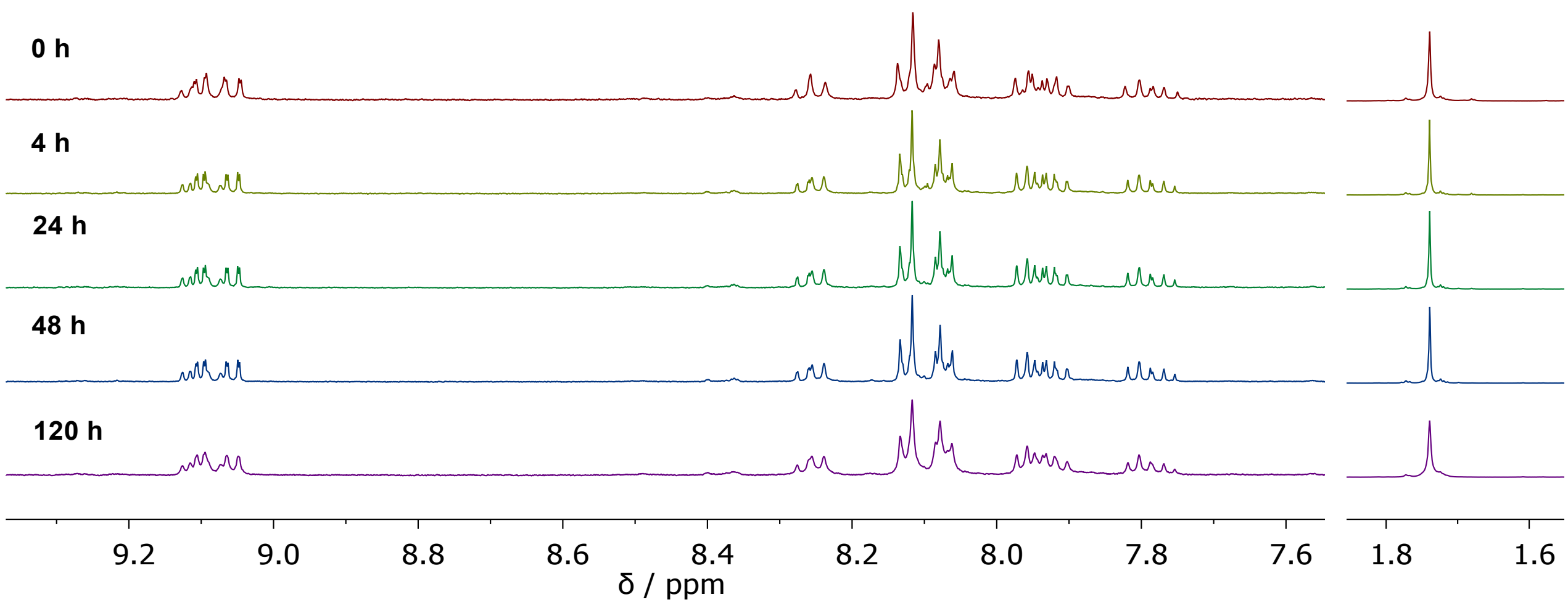

Figure S64. ^{1}H NMR evolution of complex **C4** in DMSO-*d*6.

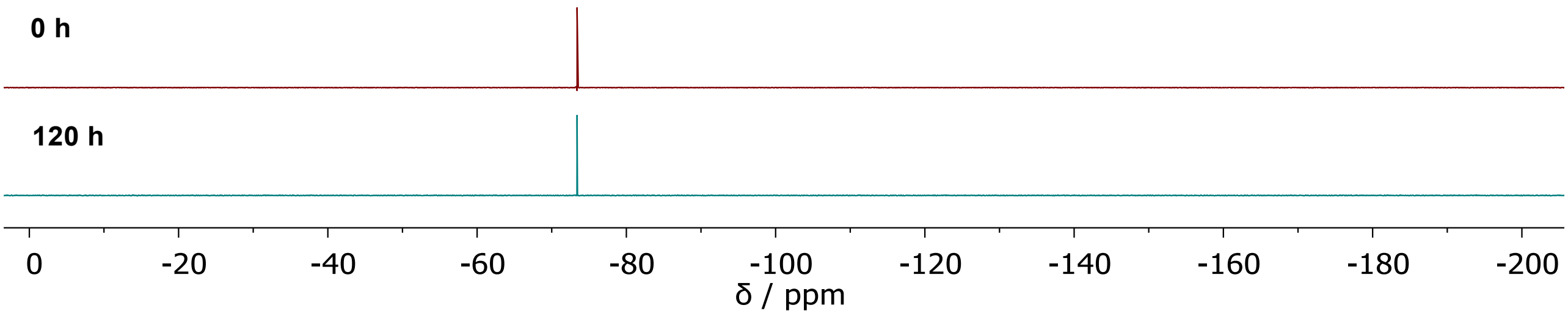

Figure S65. ^{19}F NMR evolution of complex **C4** in DMSO-*d*6.

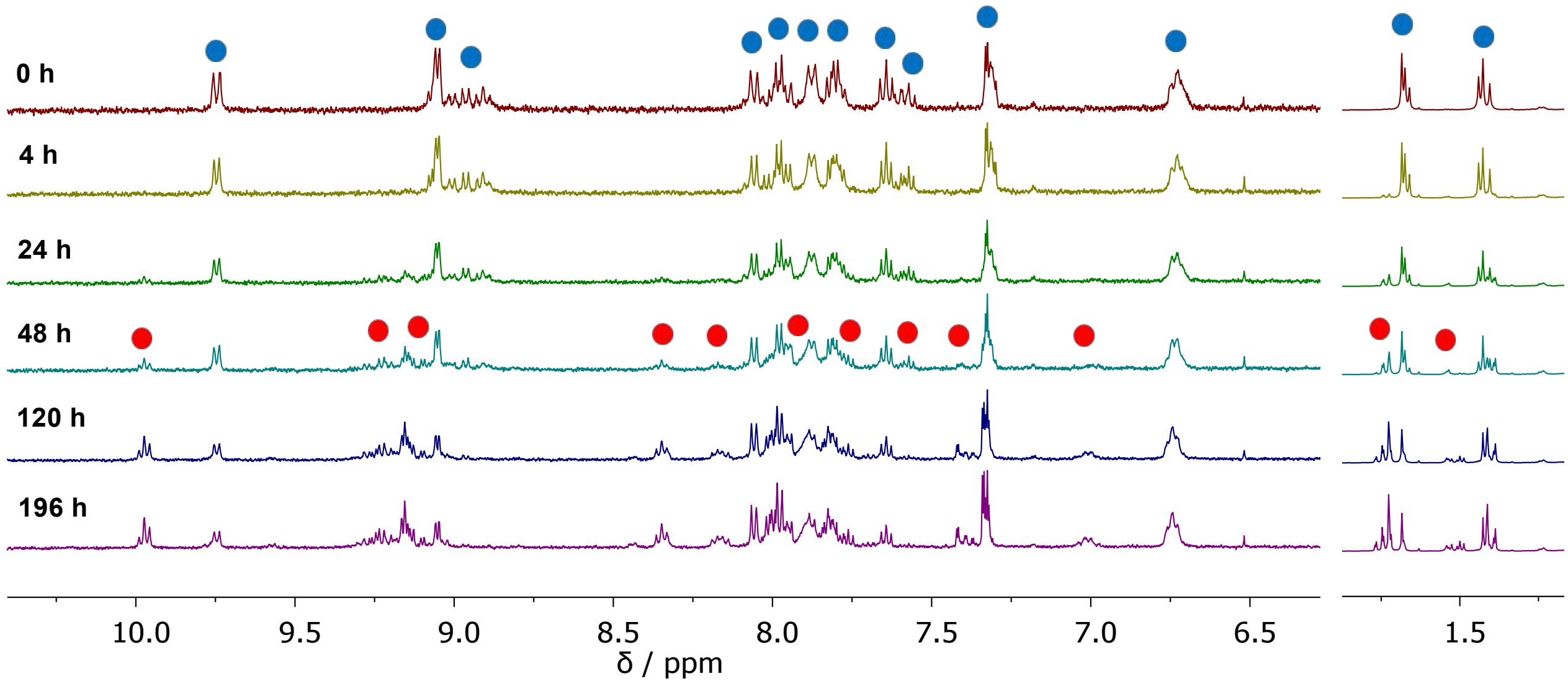

Figure S66. ^{1}H NMR evolution of complex **C5** in DMSO-*d*6. Blue circles correspond to the chlorido complex. Red circles correspond to the solvolysis product.

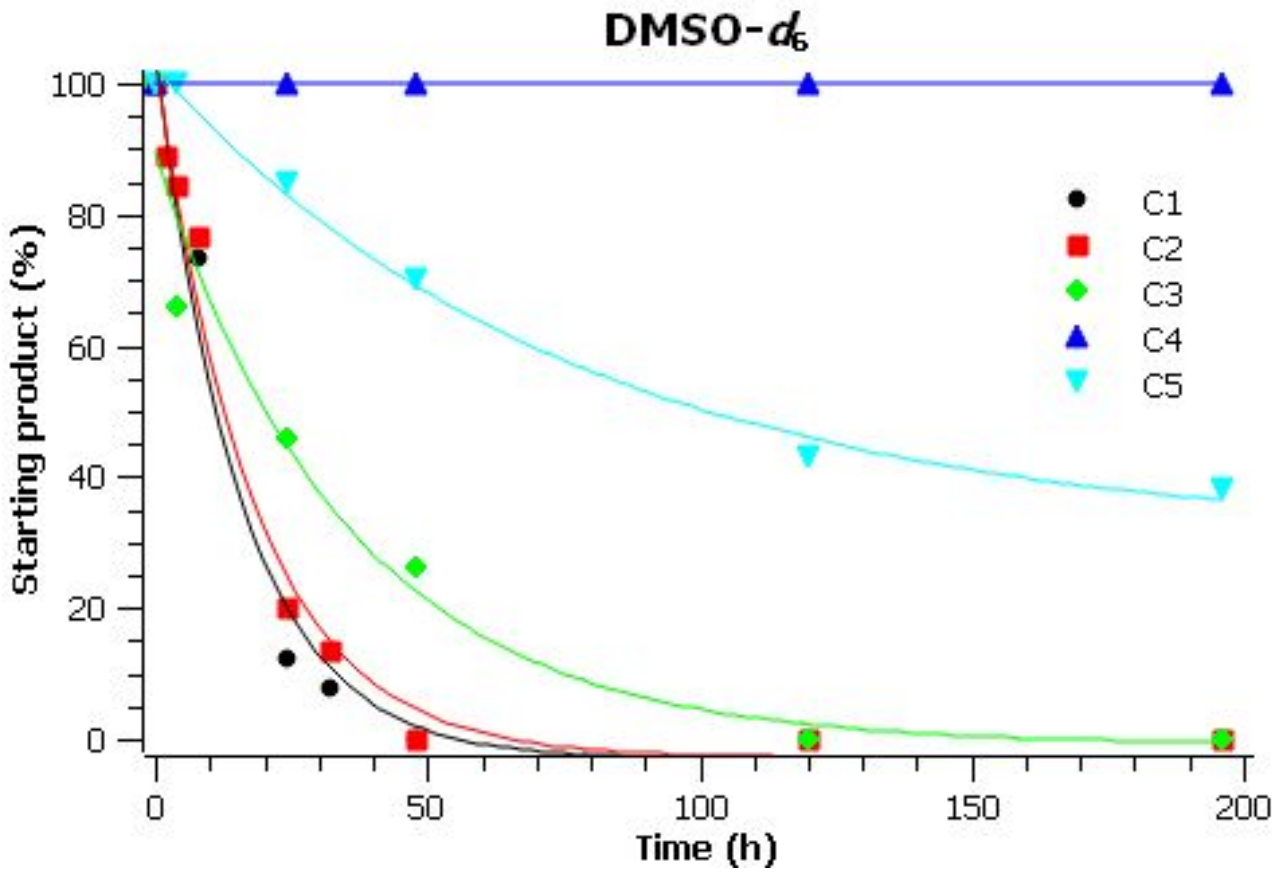

Figure S67. Hydrolysis of the Ir(III) complexes in DMSO-d_6 solution by ^{1}H NMR.

Table S3. Hydrolysis of Ir(III) complexes in DMSO-d_6 at room temperature.

	Starting product (%)				
**t (h)**	**C1**	**C2**	**C3**	**C4**	**C5**
0	100	100	100	100	100
2	89.1	89.1	-	-	-
4	83.9	84.5	65.8	100	100
8	73.3	76.5	-	-	-
24	12.3	20	46.1	100	84.7
32	7.9	13.7	-	-	-
48	0	0	26.2	100	69.9
120	-	-	0	100	42.9
196	-	-	0	100	38.2

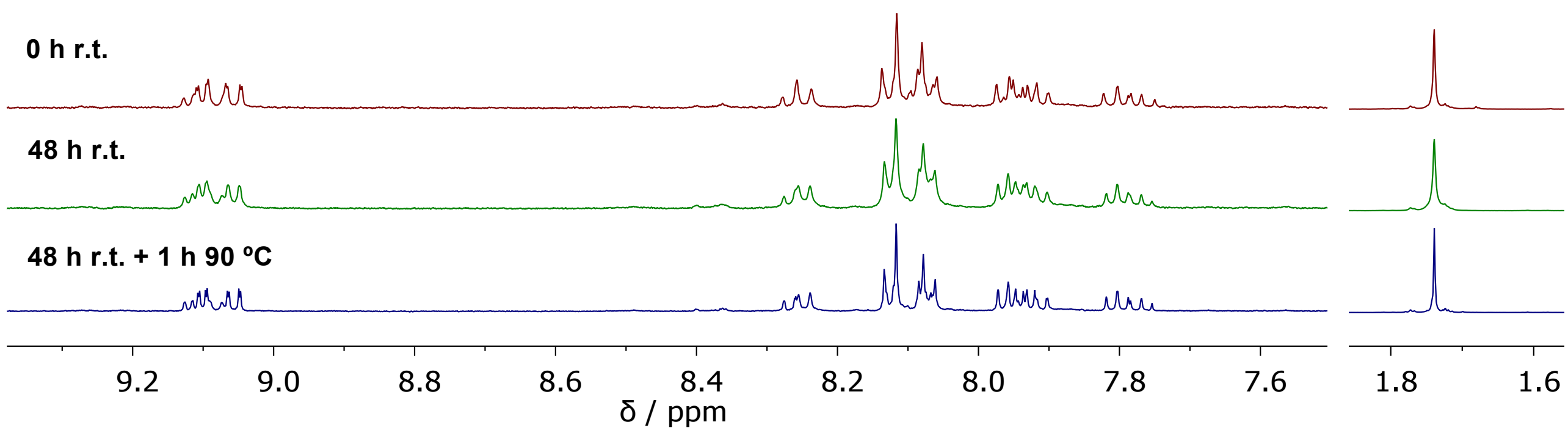

Figure S68. ^{1}H NMR evolution of complex **C4** in DMSO-d_6:D_2O (8:2) after 1 h of heating at 90 °C.

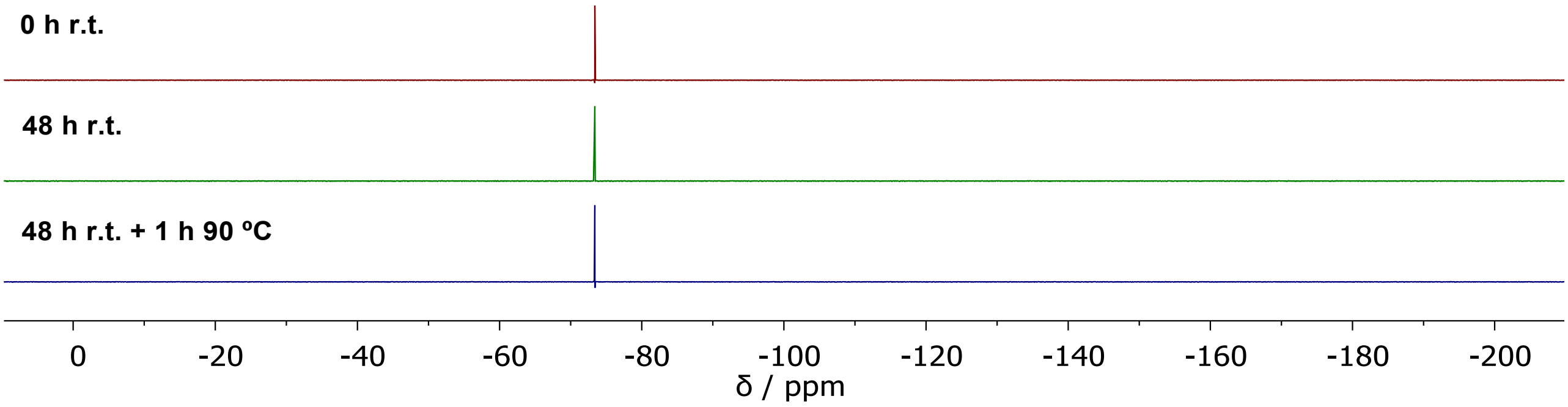

Figure S69. ^{19}F NMR evolution of complex **C4** in DMSO-d_6:D_2O (8:2) after 1 h of heating at 90 °C.

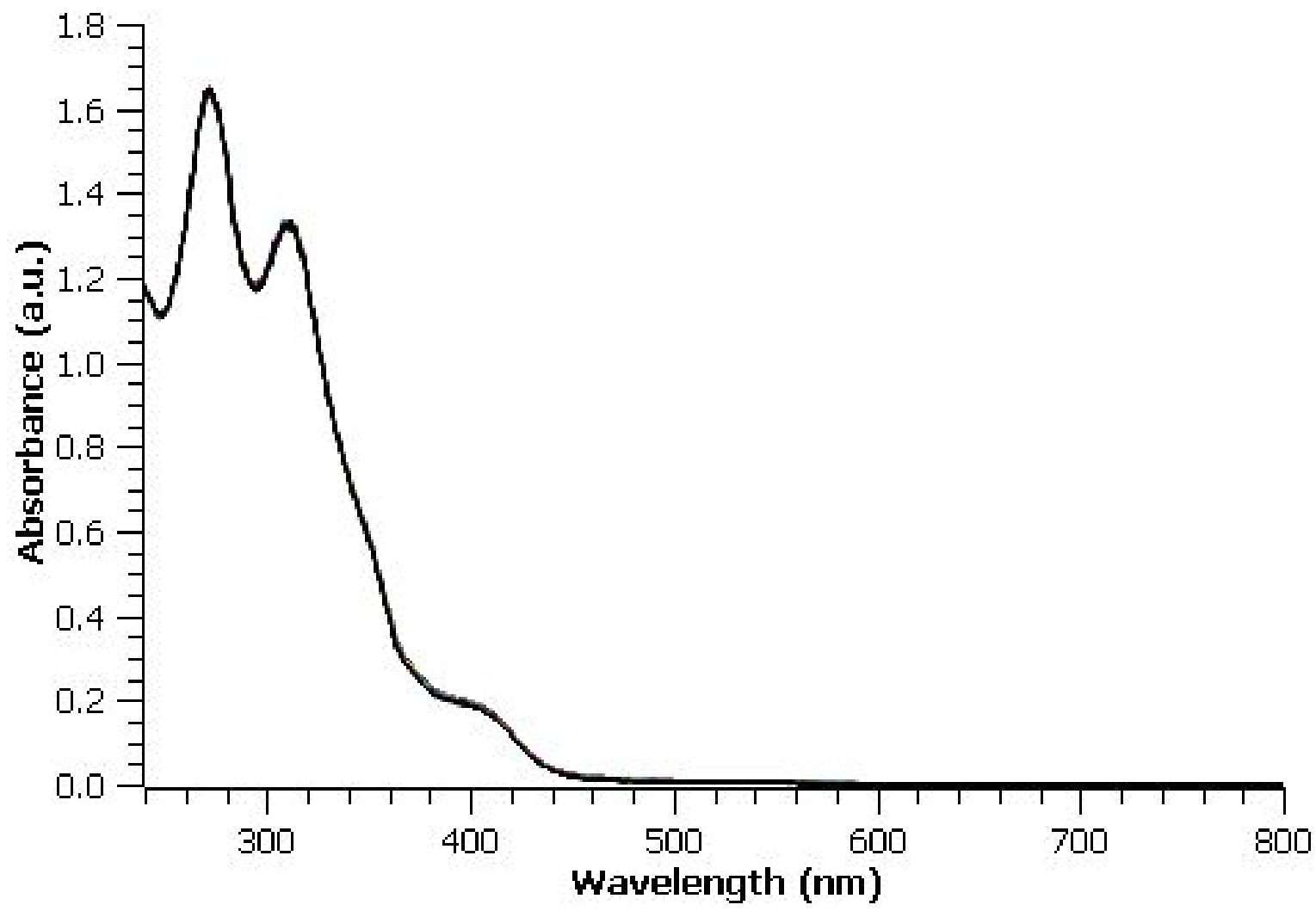

Figure S70. UV-vis spectrum of **C1w** in aqueous solution (4% DMSO) at 5.0×10^{-5} M recorded over 24 h at room temperature.

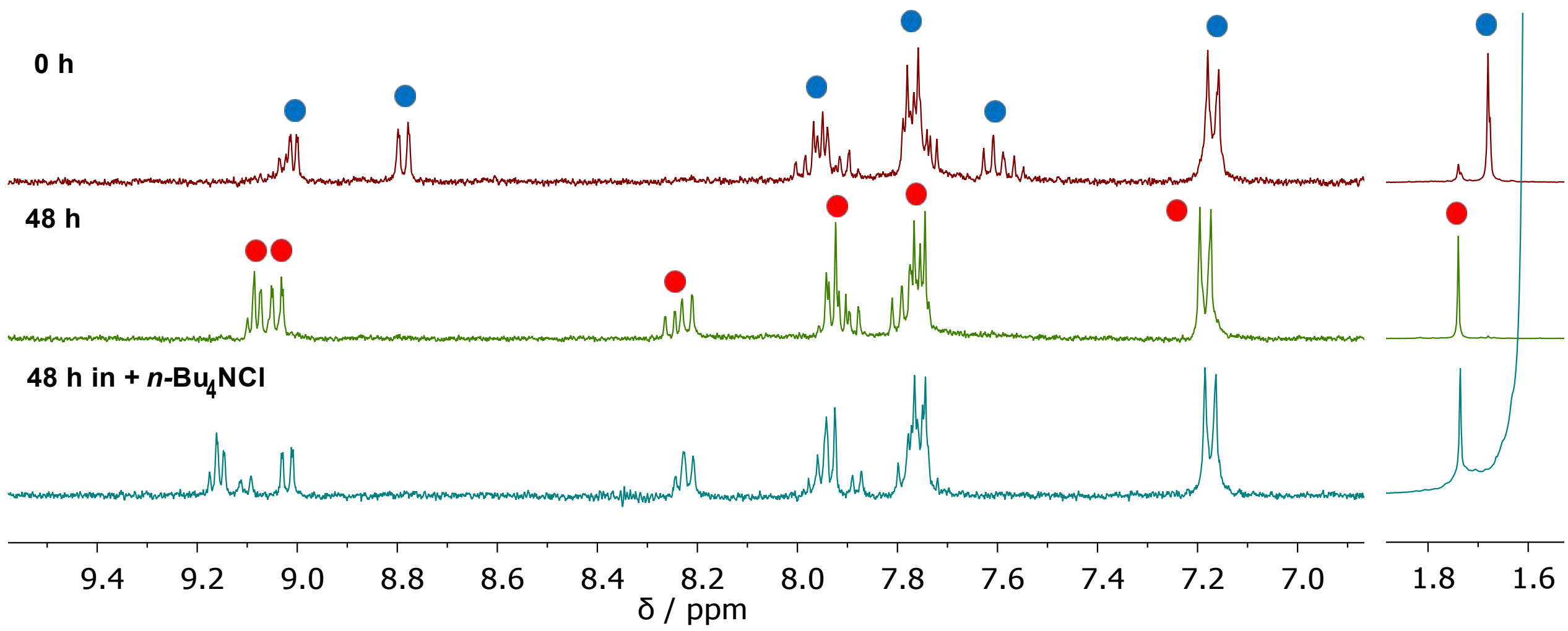

Figure S71. ^{1}H NMR evolution of the solvolysis product of **C3w** in DMSO-d_6:D_2O (8:2) after addition of 150 equiv. of *n*-Bu_4NCl at room temperature. Blue circles correspond to the chlorido complex **C3w**. Red circles correspond to the solvolysis product.

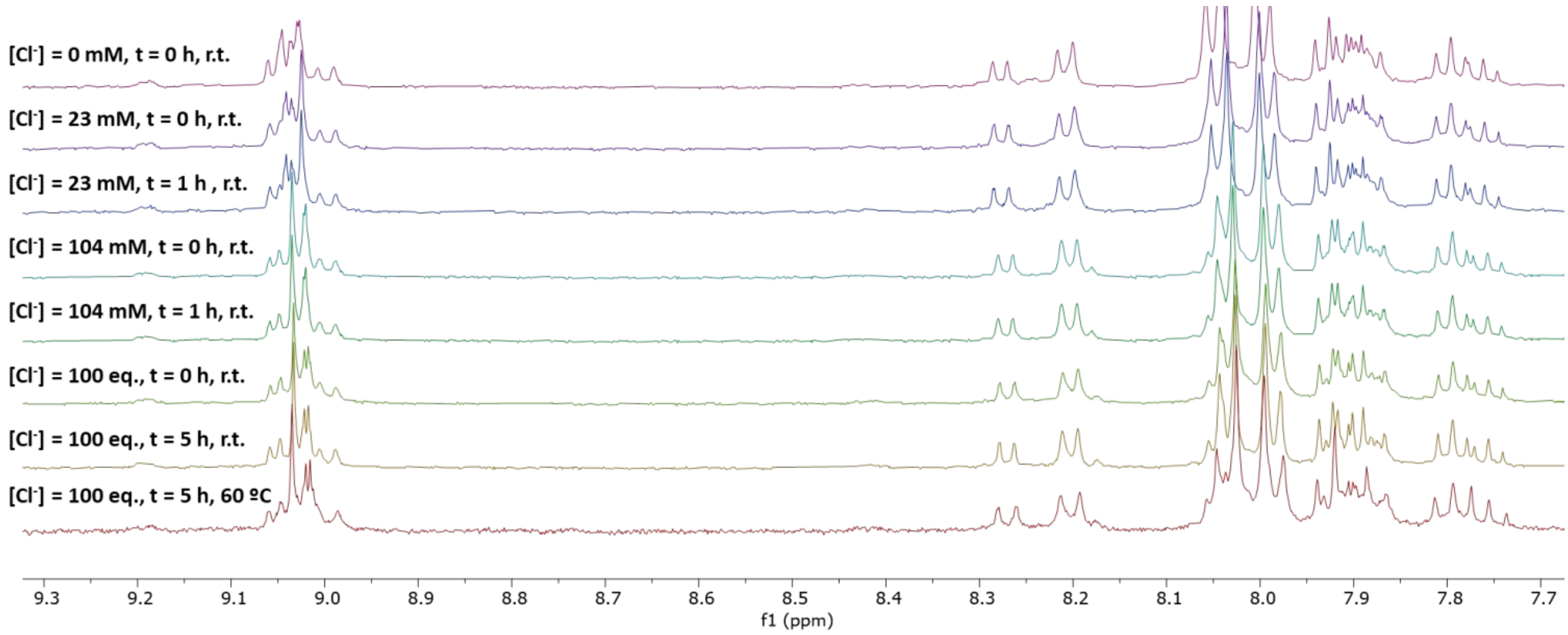

Figure S72. ^{1}H NMR spectra (500 MHz) of the aromatic region of complex **C4** recorded in the absence and in the presence of increasing chloride concentrations (23 mM, 104 mM, and 100 equivalents) in DMSO-d_6:D_2O (8:2).

5. <u>pKa determination</u>

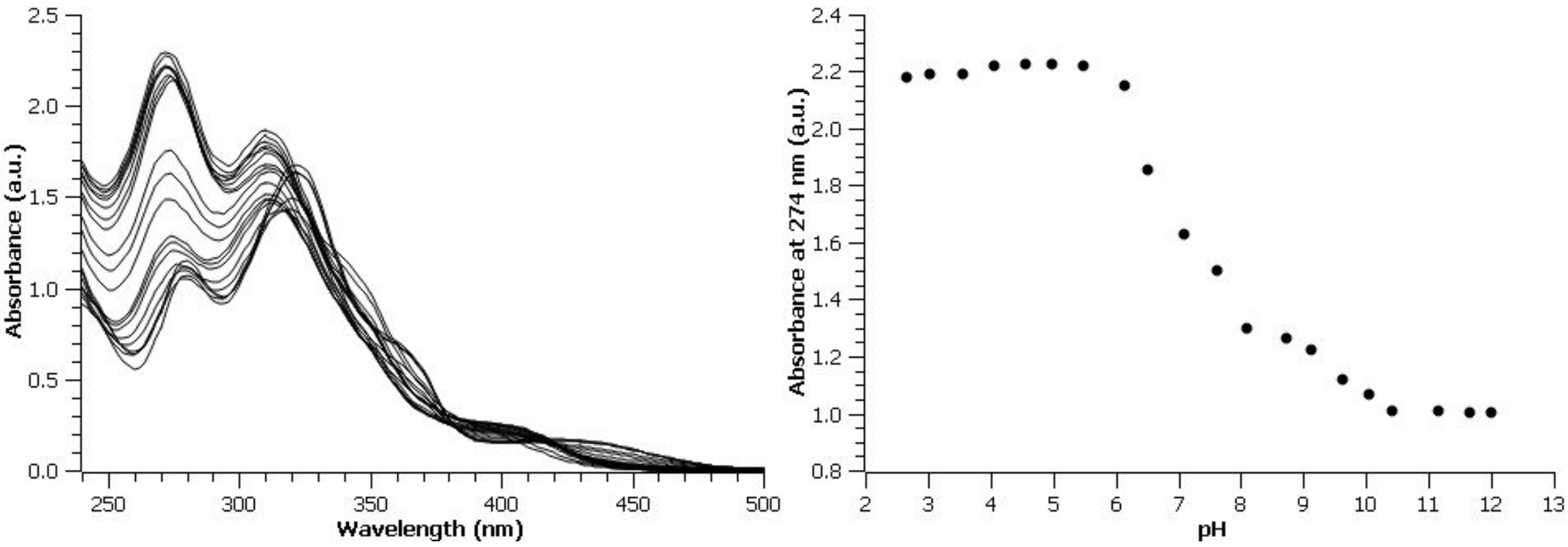

Figure S73. UV-vis spectrum of **C1w** at different pH in aqueous solution (4 % DMSO) (left) and variation of the absorbance at 274 nm vs. pH (right).

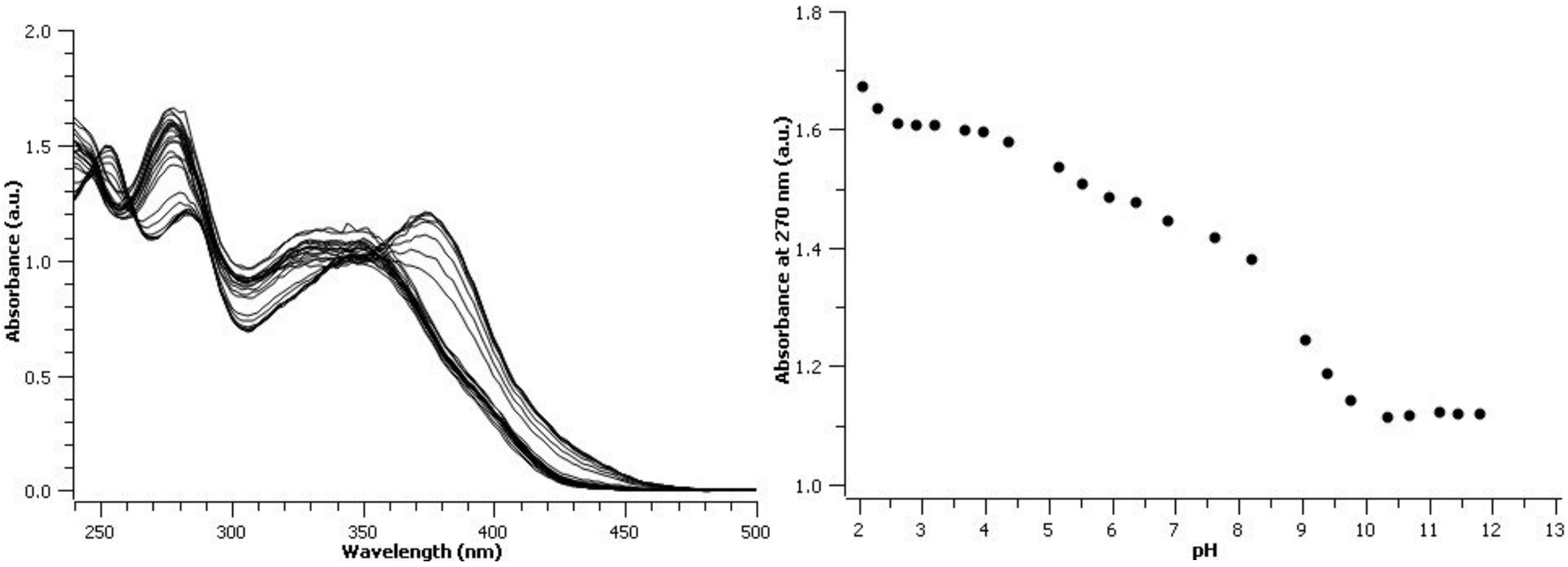

Figure S74. UV-vis spectrum of **C2w** at different pH in aqueous solution (4 % DMSO) (left) and variation of the absorbance at 270 nm vs. pH (right).

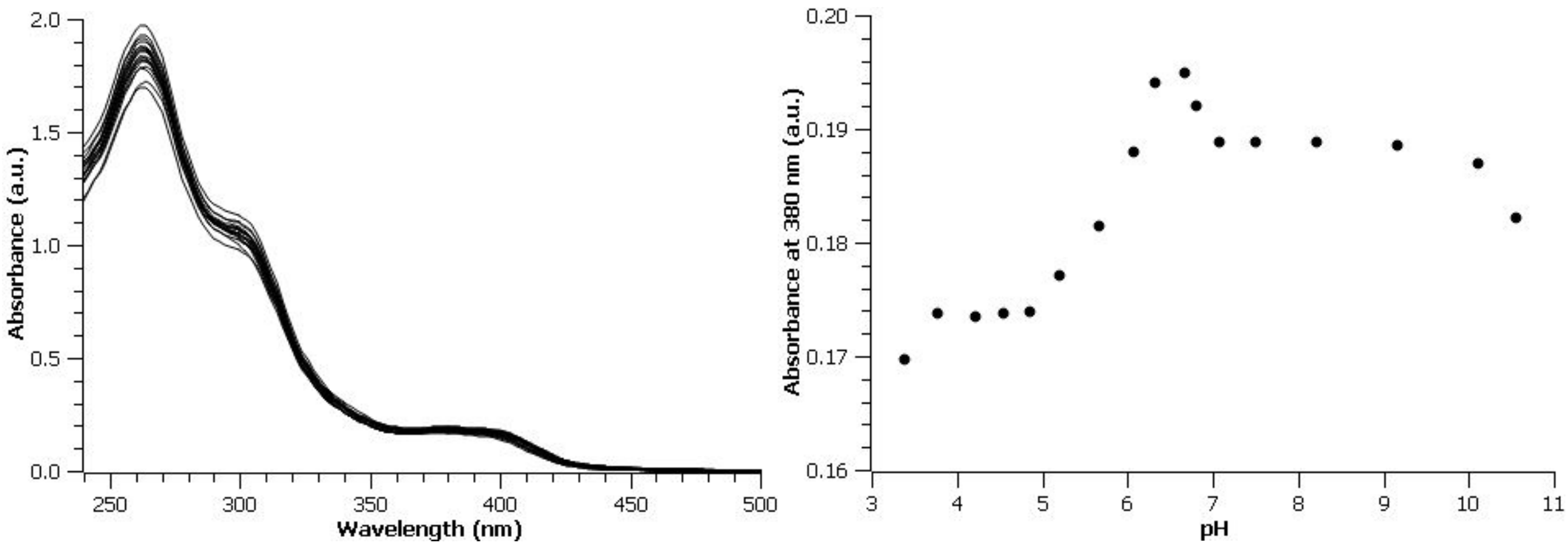

Figure S75. UV-vis spectrum of **C3w** at different pH in aqueous solution (4 % DMSO) (left) and variation of the absorbance at 380 nm vs. pH (right).

6. Photophysical properties

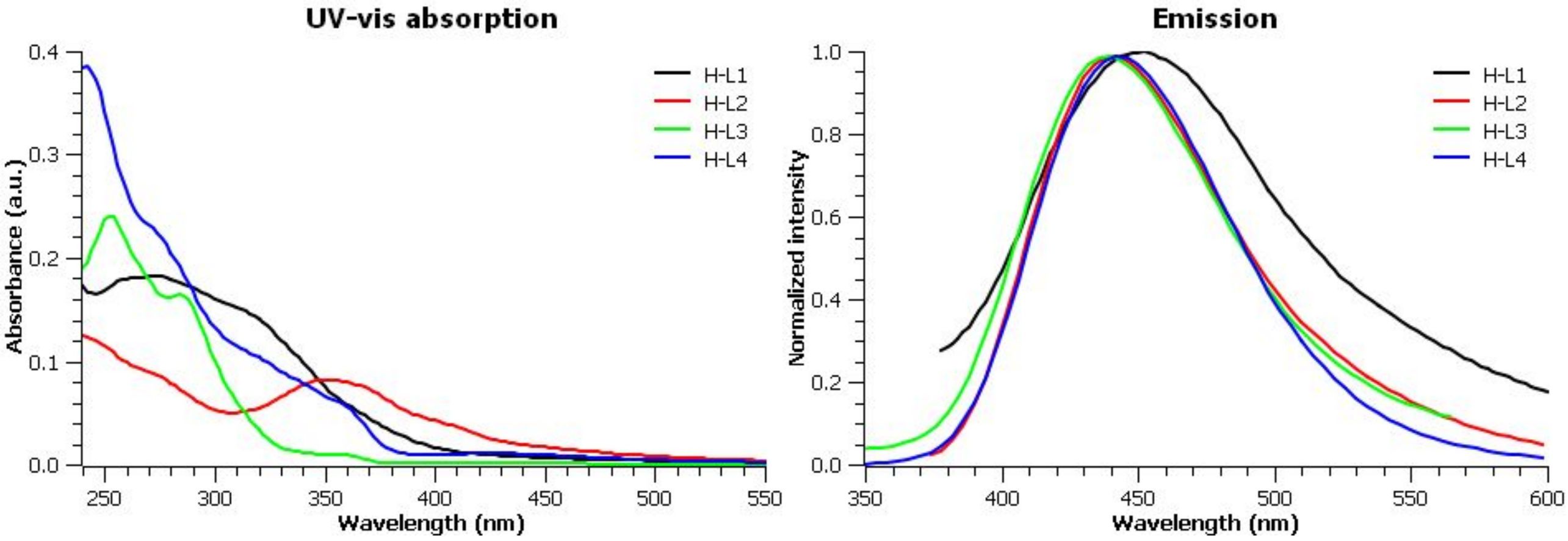

Figure S76. UV-vis absorption (left) and emission spectra (right) of proligands **H-L1**−**H-L4** in water (4 % DMSO, pH 5.5).

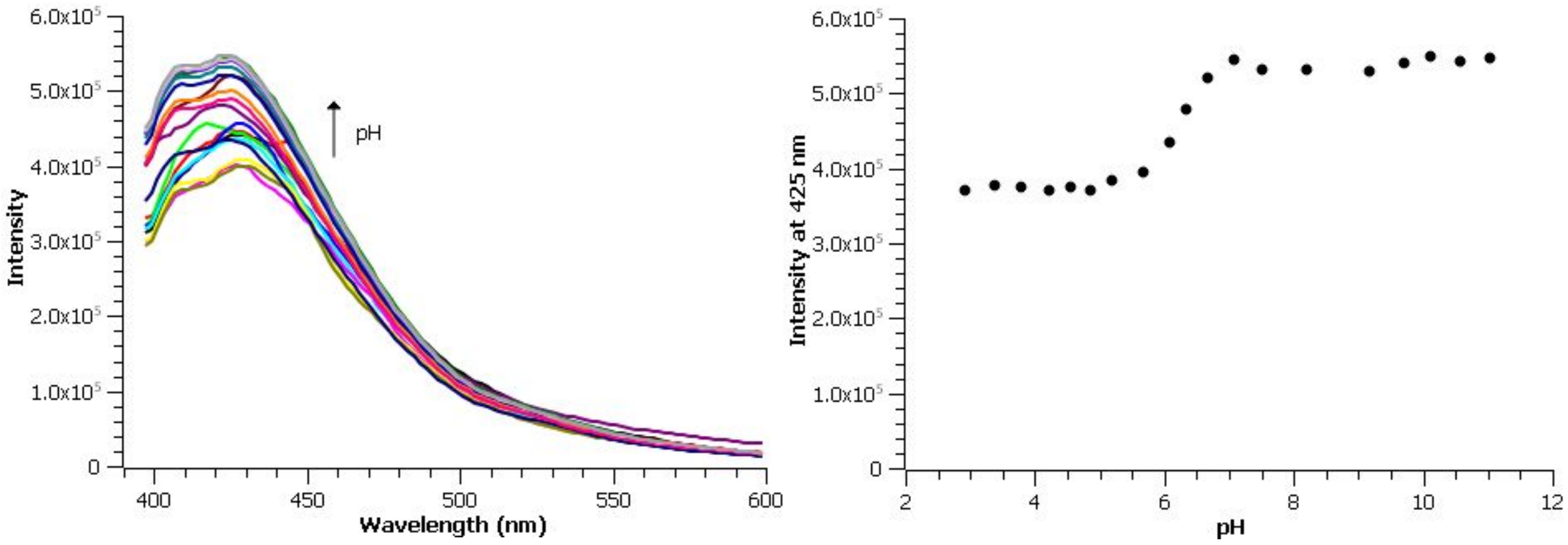

Figure S77. Emission spectrum of **C3w** at different pH in aqueous solution (4 % DMSO) (left) and variation of the emission at 425 nm vs. pH (right).

7. <u>NADH oxidation</u>

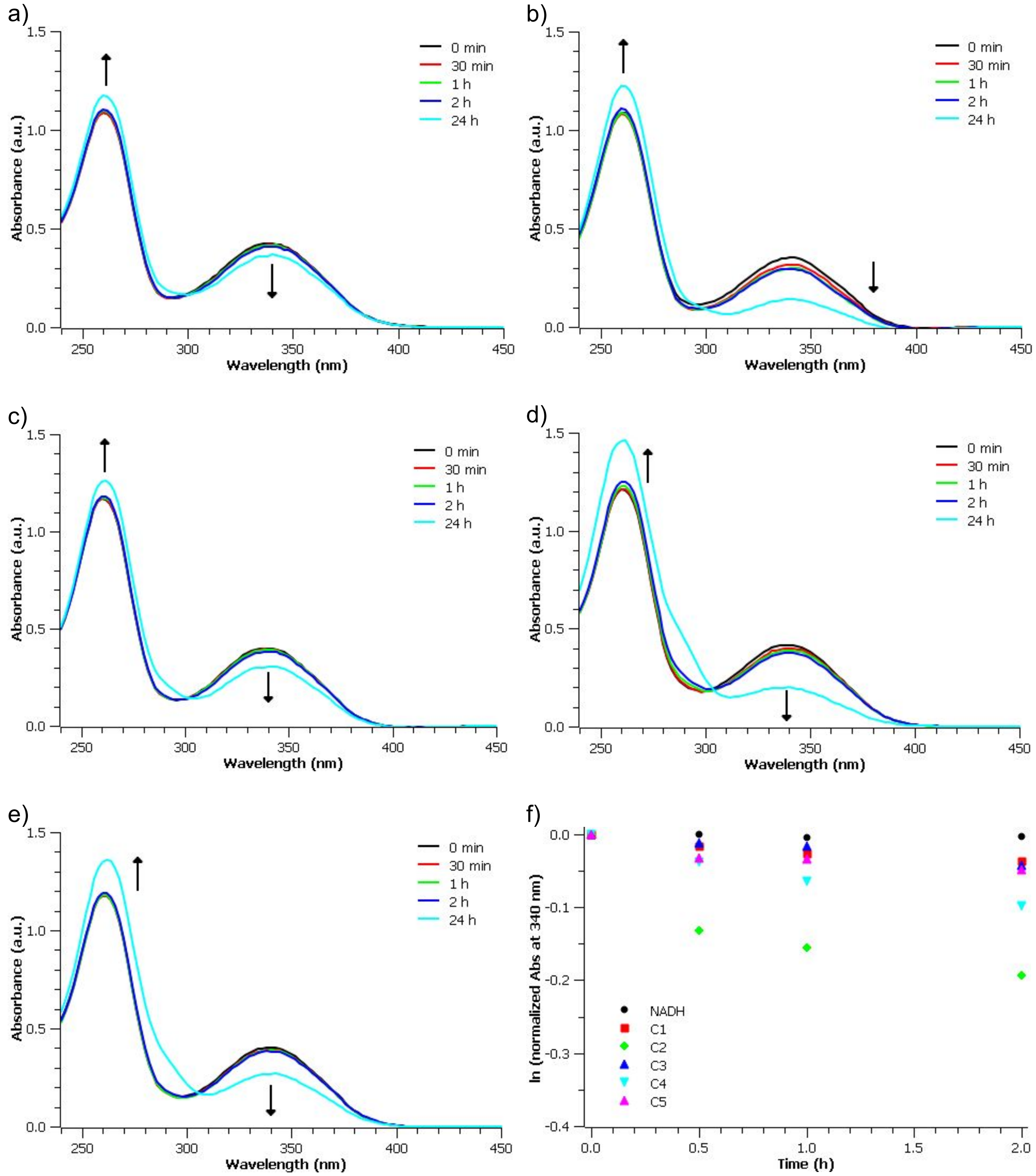

Figure S78. UV−vis spectra for the catalytic oxidation of NADH (100 µM) to NAD^+ in the presence of complexes **C1w** (a), **C2w** (b), **C3w** (c), **C4** (d) and **C5w** (e) at 2.5 µM in water (0.1 % DMSO). f) First-order kinetic plot of the oxidation of NADH with Ir(III) complexes.

Table S4. TON and TOF parameters of the catalytic oxidation of NADH (100 µM) in the presence of Ir(III) complexes (2.5 µM) in aqueous solution (complex/NADH ratio = 1/40) at room temperature.

	**C1w**	**C2w**	**C3w**	**C4**	**C5w**

**$TON_{24\ h}$**	4.4 ± 0.9	16 ± 3	5 ± 1	19 ± 3	8 ± 1
**$TOF_{30\ min}$ (h^{-1})**	1.1 ± 0.2	7 ± 1	1.7 ± 0.6	2.7 ± 0.5	3.1 ± 0.5

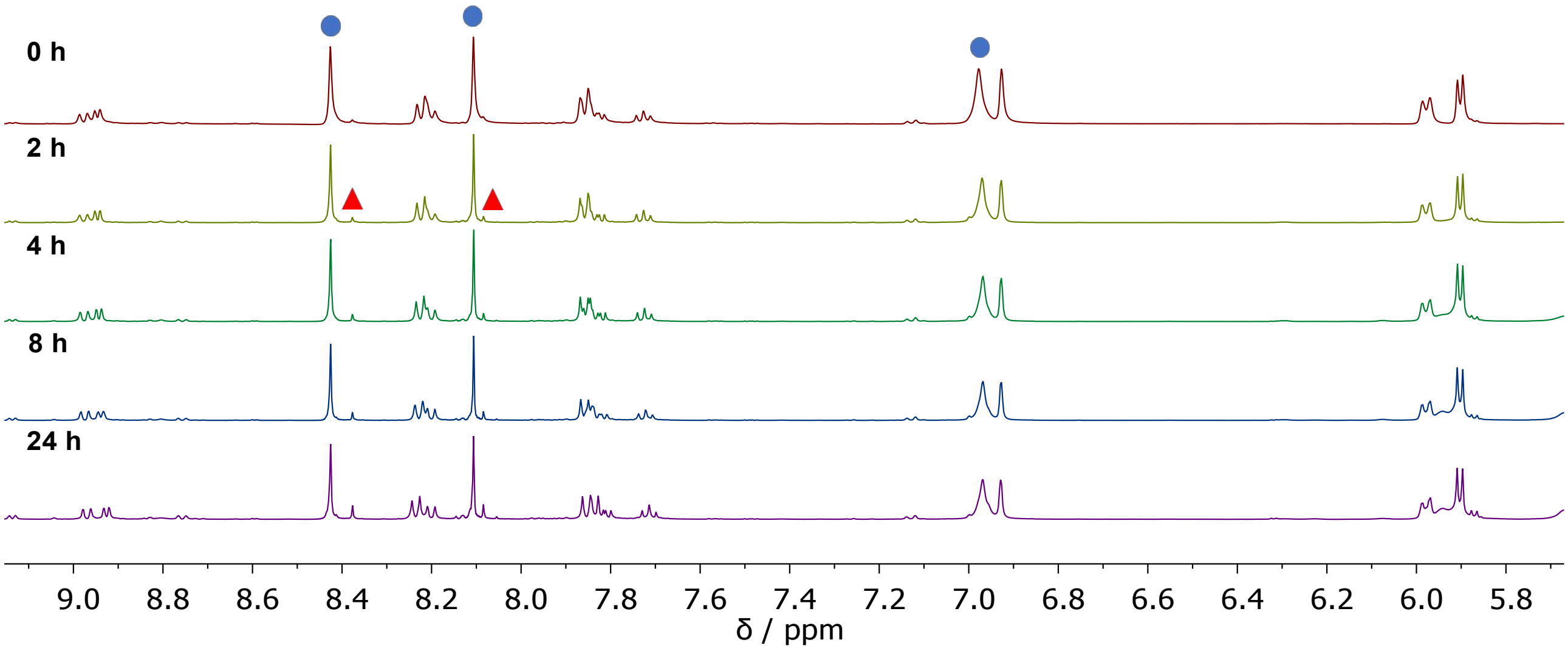

Figure S79. ^{1}H NMR spectra of the catalytic oxidation of NADH with complex **C2w** in DMSO-*d*$_6$:H_2O (1:1). Blue circles correspond to reduced NADH and red triangles correspond to oxidized NAD^+.

Table S5. Catalytic oxidation of NADH by representative half-sandwich Ir(III) complexes reported in literature.

Complex	TON / time (h)	TOF (h^{-1}) / time (h)	Reference
$[Cp^{xbiph}Ir(N^\wedge C_{NHC})Cl]^+$	85 / 8	10.6 / 8	a
$[Cp^*Ir(N^\wedge N)Cl]^+$	60.1 / 8	7.5 / 8	b
$[Cp^{xbiph}Ir(N^\wedge N)(OH_2)]^{2+}$	28.7 / 7.5	3.8 / 7.5	c
$[Cp^*Ir(C^\wedge N)(OH_2)]^+$	28 / 24	1.2 / 24	d
$[Cp^*Ir(N^\wedge N)(OH_2)]^{2+}$	20.2 / 7.5	2.7 / 7.5	c
$[Cp^{xph}Ir(N^\wedge N)Cl]^+$	18.8 / 8	2.4 / 8	e
$[Cp^*Ir(N^\wedge N)(OH_2)]^{2+}$	15.9 / 4	4.0 / 4	f
$[Cp^{xbiph}Ir(C_{NHC}{}^\wedge C_{NHC})(OH_2)]^{2+}$	15.3 / 8	1.9 / 8	g
$[Cp^{xph}Ir(Cl)_2(pyR)]$	15.28 / 8	1.9 / 8	h
$[Cp^*Ir(N^\wedge N)Cl]^+$	12.7 / 4	3.2 / 4	f
$[Cp^*Ir(N^\wedge N)Cl]^+$	9.3 / 8	1.2 / 8	e
$[Cp^{xbiph}Ir(C^\wedge N)(OH_2)]^+$	8.2 / 20	0.41 / 20	i
$[Cp^{xbiph}Ir(C^\wedge N)(py)]^+$	7.6 / 20	0.38 / 20	i
$[Cp^*Ir(C^\wedge N)(OH_2)]^+$	5.5 / 14	0.39 / 14	j
$[Cp^*Ir(C^\wedge N)(py\text{-}BODIPY)]^+$	3.6 / 14	0.26 / 14	j
$[Cp^*Ir(C^\wedge N)(DMSO)]^+$	3.3 / 2.5	1.3 / 2.5	k
$[Cp^*Ir(C^\wedge N)(Im\text{-}R)]^+$	0.94 / 1	0.94 / 1	l

a. Yang, Y.; Guo, L.; Ge, X.; Shi, S.; Gong, Y.; Xu, Z.; Zheng, X.; Liu, Z. Structure-activity relationships for highly potent half-sandwich organoiridium(III) anticancer complexes with C^N-chelated ligands, *J. Inorg. Biochem.* **2019**, *191*, 1-7. https://doi.org/10.1016/j.jinorgbio.2018.11.007

b. Lv, A.; Lia, G.; Zhang, P.; Tao, R.; Li, X.; Ren, X.; Li, P.; Liu, X.; Yuan, X.-A.; Liu, Z. Design and anticancer behaviour of cationic/neutral half-sandwich iridium (III) imidazole-phenanthroline/phenanthrene complexes, *J. Inorg. Biochem.* **2024**, *257*, 112612-112620. https://doi.org/10.1016/j.jinorgbio.2024.112612

c. Li, J.-J.; Guo, L.; Tian, Z.; Tian, M.; Zhang, S.; Xu, K.; Qian, Y.; Liu, Z. Novel half-sandwich iridium(III) imino-pyridyl complexes showing remarkable in vitro anticancer activity, *Dalton Trans.* **2017**, *46*, 15520–15534. https://doi.org/10.1039/c7dt03265j

d. Millet, A. J.; Habtemariam, A.; Romero-Canelón, I.; Clarkson, G. J.; Sadler, P. J. Contrasting anticancer activity of half-sandwich iridium(III) complexes bearing functionally diverse 2-phenylpyridine ligands, *Organometallics* **2015**, *34* (11), 2683–2694. https://doi.org/10.1021/acs.organomet.5b00097

e. Chen, S.; Liu, X.; Ge, X.; Wang, Q.; Xie, Y.; Hao, Y.; Zhang, Y.; Zhang, L.; Shang, W.; Liu, Z. Lysosome-targeted iridium(III) compounds with pyridine-triphenylamine Schiff base ligands: syntheses, antitumor applications and mechanisms, *Inorg. Chem. Front.* **2020**, 7, 91–100. https://doi.org/10.1039/c9qi01161g

f. Masaryk, L.; Orvoš, J.; Słoczyńska, K.; Herchel, R.; Moncol, J.; Milde, D.; Halaš, P.; Křikavová, R.; Koczurkiewicz-Adamczyk, P.; Pękala, E.; Fischer, R.; Šalitroš, I.; Nemec, I.; Štarha, P. Anticancer half-sandwich Ir(III) complex and its interaction with various biomolecules and their mixtures – a case study with ascorbic acid, *Inorg. Chem. Front.* **2022**, *9*, 3758–3770. https://doi.org/10.1039/D2QI00535B

g. Wang, C.; Liu, J.; Tian, Z.; Tian, M.; Tian, L.; Zhao, W.; Liu, Z. Half-sandwich iridium N-heterocyclic carbene anticancer complexes, *Dalton Trans.* **2017**, *46*, 6870-6883. https://doi.org/10.1039/C7DT00575J

h. Wang, L.; Liu, X.; Wu, Y.; He, X.; Guo, X.; Gao, W.; Tan, L.; Yuan, X.-A.; Liu, J.; Liu, Z. In vitro and in vivo antitumor assay of mitochondrially targeted fluorescent half-sandwich iridium(III) pyridine complexes, *Inorg. Chem.* **2023**, *62*, 3395−3408. https://doi.org/10.1021/acs.inorgchem.2c03333

i. Liu, Z.; Romero-Canelón, I.; Qamar, B.; Hearn, J. M.; Habtemariam, A.; Barry, N. P. E.; Pizarro, A. M.; Clarkson, G. J.; Sadler, P. J. The potent oxidant anticancer activity of organoiridium catalysts, *Angew. Chem. Int. Ed.* **2014**, *53*, 3941-3946. https://doi.org/10.1002/anie.201311161

j. Zimbron, J. M.; Passador, K.; Gatin-Fraudet, B.; Bachelet, C.-M.; Plażuk, D.; Chamoreau, L.-M.; Botuha, C.; Salmain, M. Synthesis, photophysical properties, and living cell imaging of theranostic half-sandwich iridium–4,4-difluoro-4-bora-3*a*,4*a*-diaza-*s*-indacene (BODIPY) dyads, *Organometallics* **2017**, *36* (18), 3435–3442. https://doi.org/10.1021/acs.organomet.7b00250

k. Ramos, R.; Zimbron, J. M.; Thorimbert, S.; Chamoreau, L.-M.; Munier, A.; Botuha, C.; Karaiskou, A.; Salmain, M.; Sobczak-Thépot, J. Insights into the antiproliferative mechanism of (C^N)-chelated half-sandwich iridium complexes, *Dalton Trans.* **2020**, *49*, 17635–17641. https://doi.org/10.1039/d0dt03414b

l. Gonzalo-Navarro, C.; Zafon, E.; Organero, J. A.; Jalón, F. A.; Lima, J. C.; Espino, G.; Rodríguez, A. M.; Santos, L.; Moro, A. J.; Barrabés, S.; Castro, J.; Camacho-Aguayo, J.; Massaguer, A.; Manzano, B. R.; Durá, G. Ir(III) Half-sandwich photosensitizers with a π-expansive ligand for efficient anticancer photodynamic therapy, *J. Med. Chem.* **2024**, *67* (3), 1783–1811. https://doi.org/10.1021/acs.jmedchem.3c01276

8. Depletion of GSH

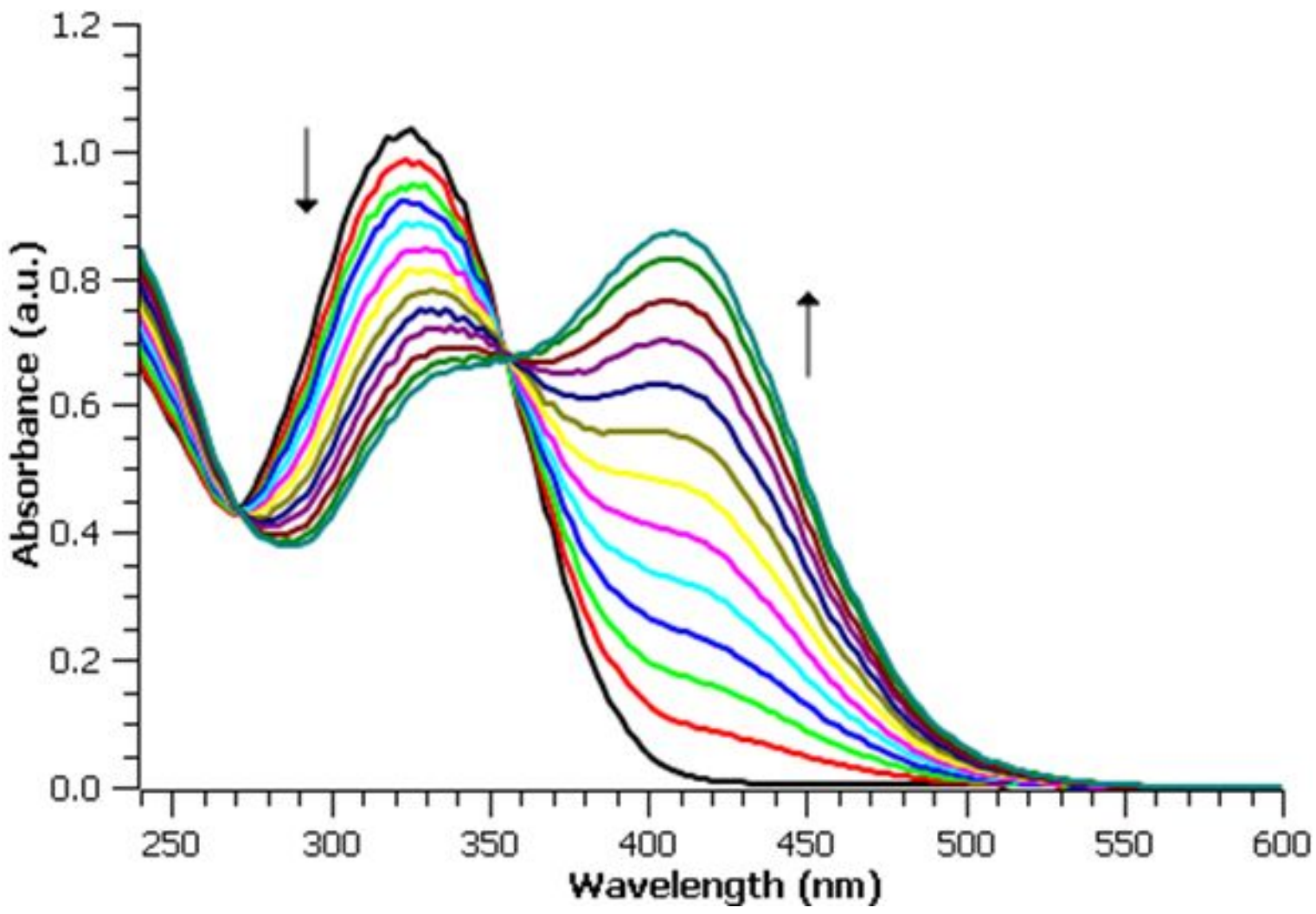

Figure S80. Evolution of the UV-vis spectrum of DTNB upon addition of increasing concentrations of GSH. The reaction between DTNB and GSH leads to a progressive decrease of the characteristic DTNB band at 326 nm and the concomitant appearance and increasing of the TNB band at 412 nm.

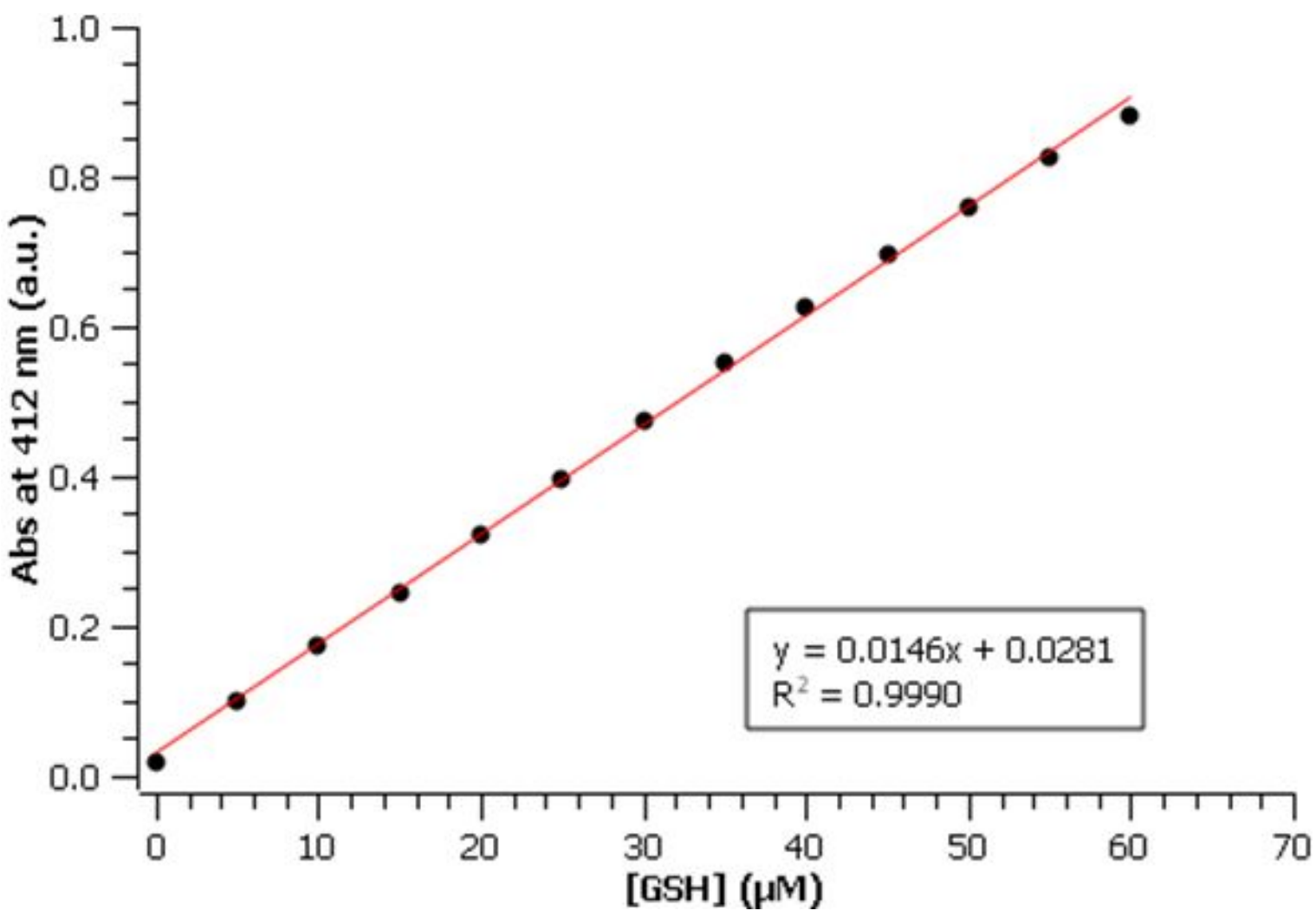

Figure S81. Calibration curve for the reaction of DTNB with GSH.

Table S6. GSH depletion by the Ir(III) complexes.[a]

Compound	[GSH] consumed (M)
**C1w**	24.5
**C2w**	18.9
**C3w**	19.8
**C4**	19.1
**C5w**	20.2

[a] GSH (50 μM) was exposed to the compounds (10 μM) and kept in the dark for 4 hours.

9. Interaction with BSA

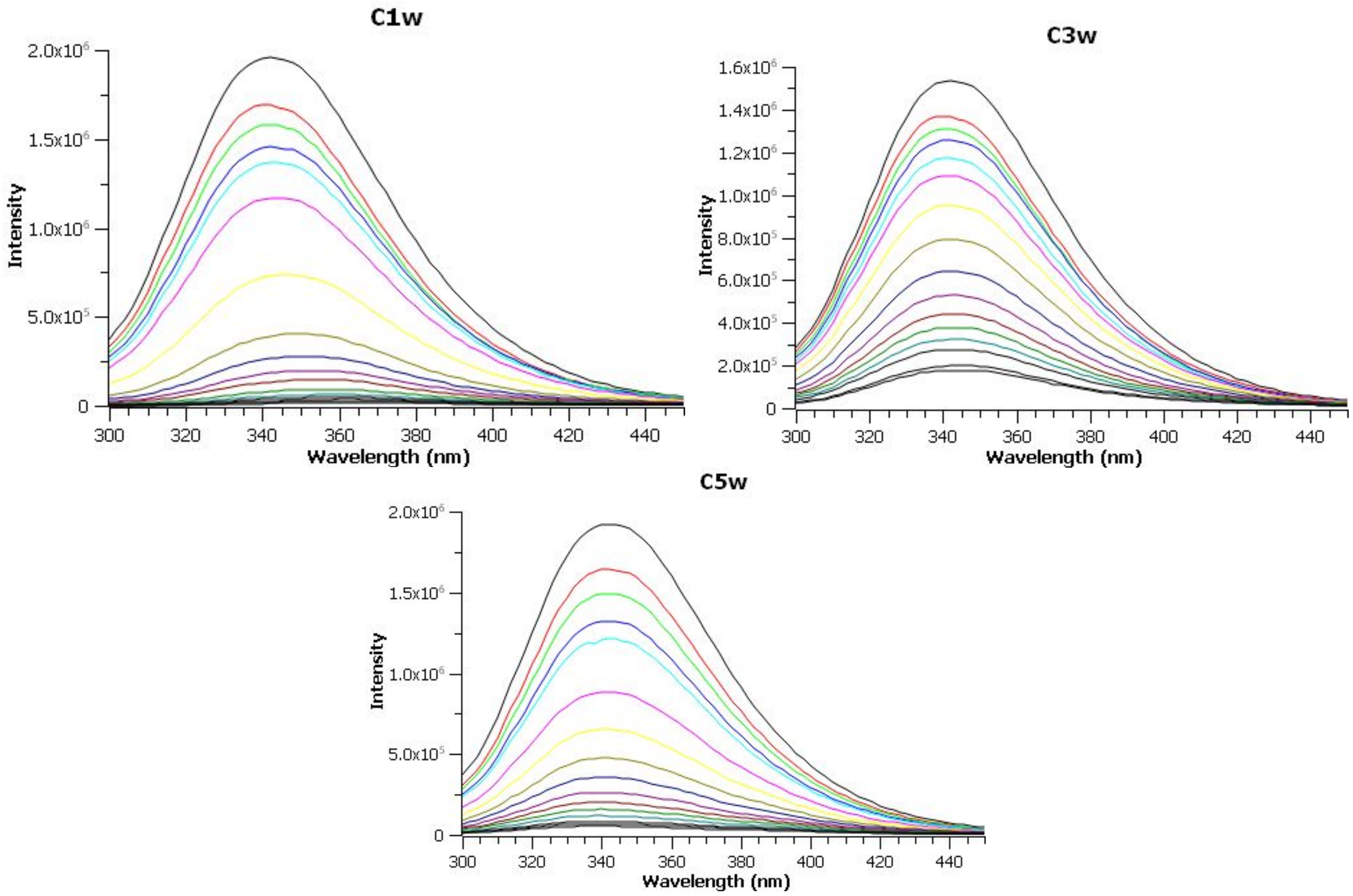

Figure S82. Evolution of the fluorescence emission spectrum of BSA with increasing concentrations of complexes **C1w**, **C3w** and **C5w** from 0 to 60 μM in water. The emission is quenched with the addition of the complexes.

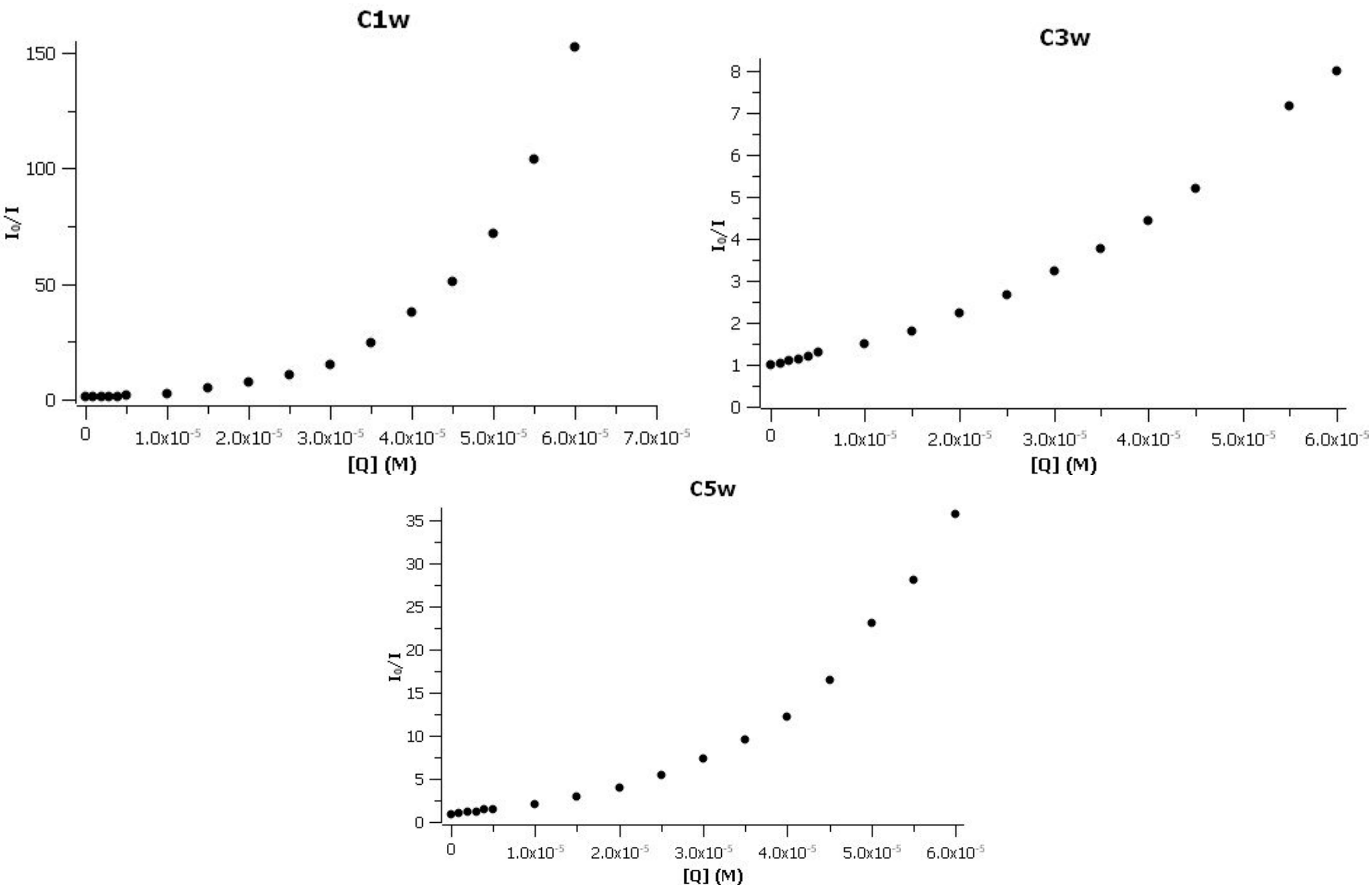

Figure S83. Stern-Volmer plots for the interaction of BSA with complexes **C1w**, **C3w** and **C5w** in water.

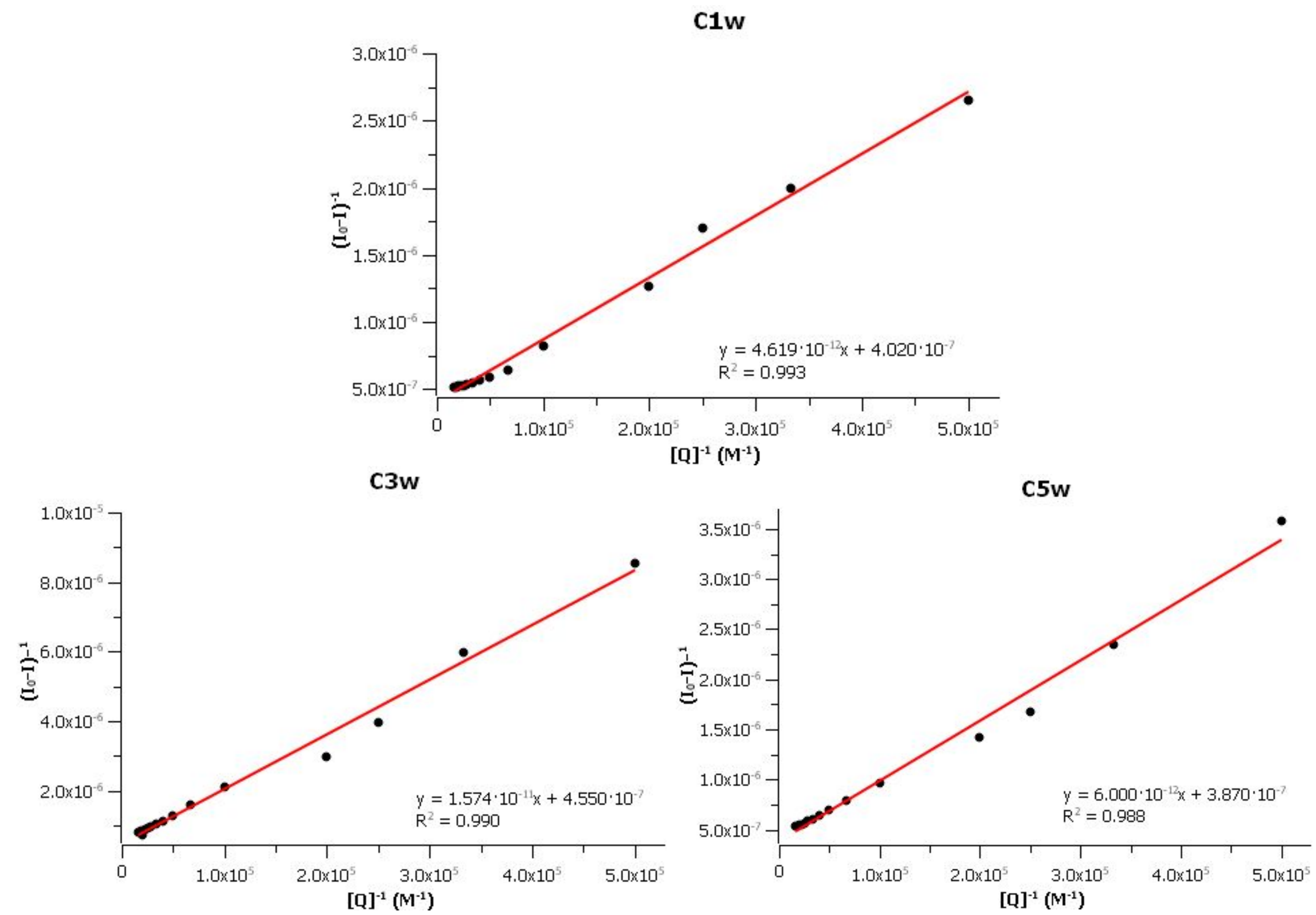

Figure S84. Lineweaver-Burk plots for the interaction of BSA with complexes **C1w**, **C3w** and **C5w** in water.

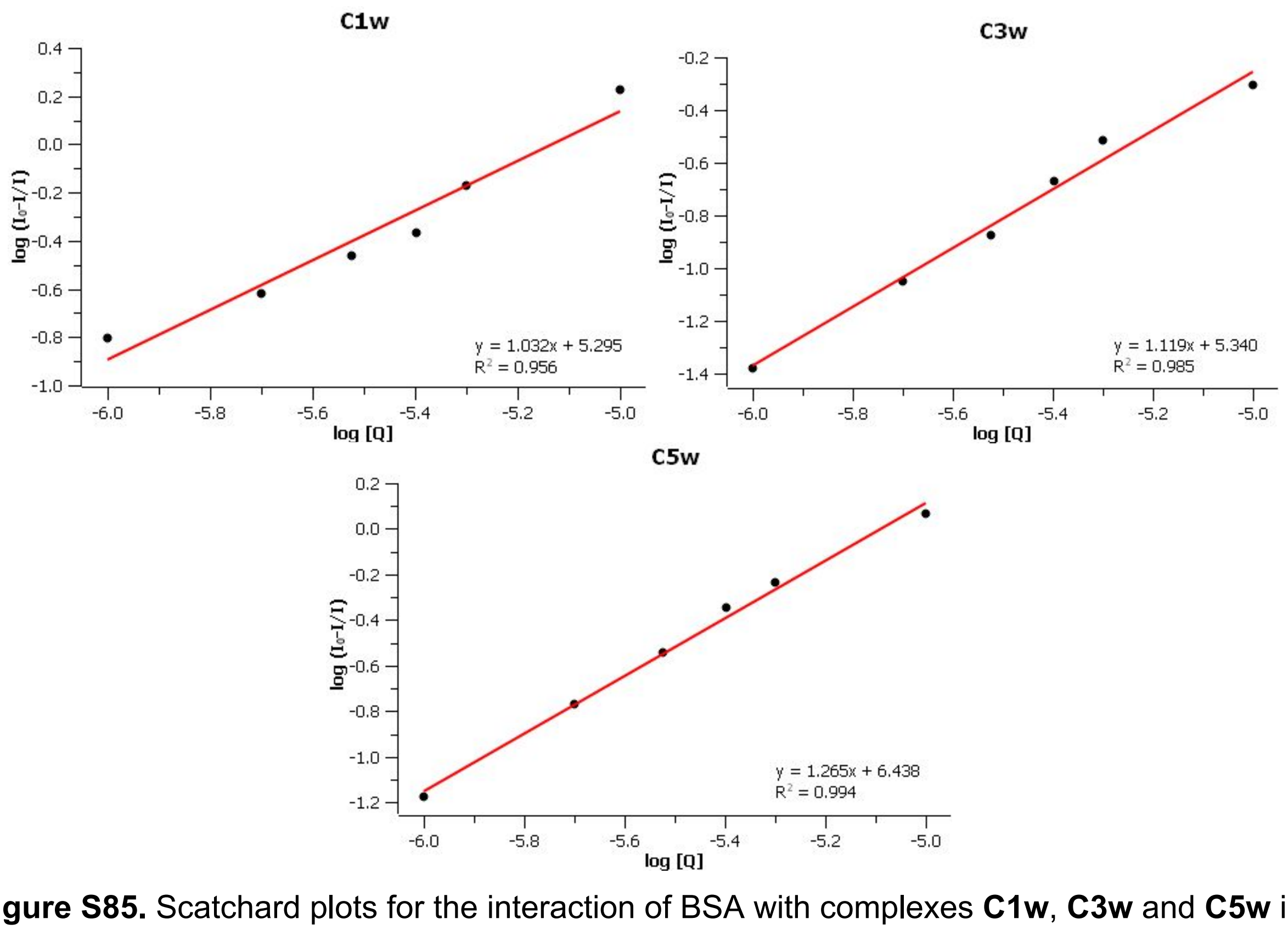

Figure S85. Scatchard plots for the interaction of BSA with complexes **C1w**, **C3w** and **C5w** in water.

10. Molecular docking

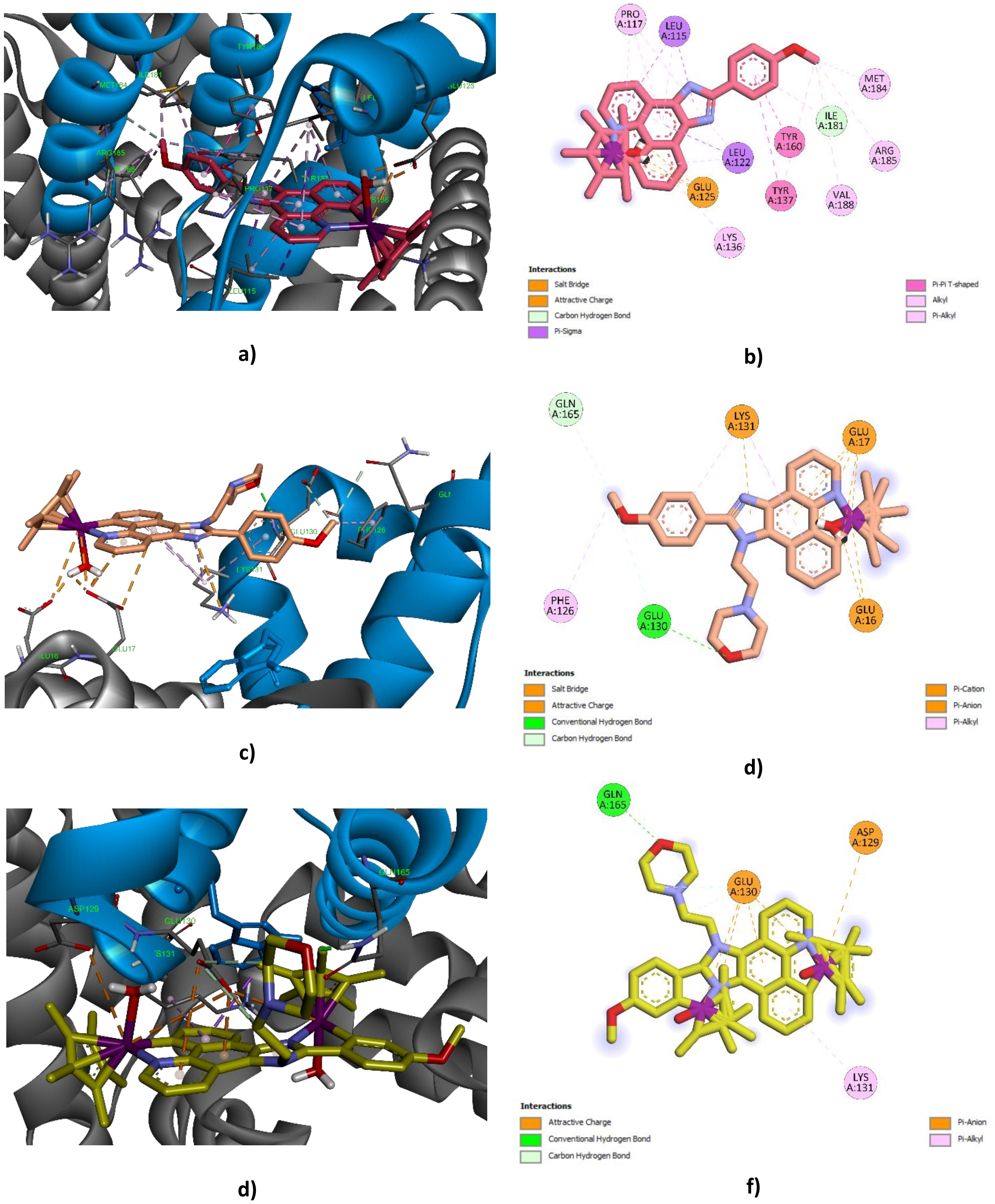

Figure S86. Three-dimensional (3D) and two-dimensional (2D) representations of the interactions of complexes **C1w** (3D: a, 2D: b), **C3w** (3D: c, 2D: d), and **C5w** (3D: e, 2D: f) with BSA (site III is highlighted in blue).

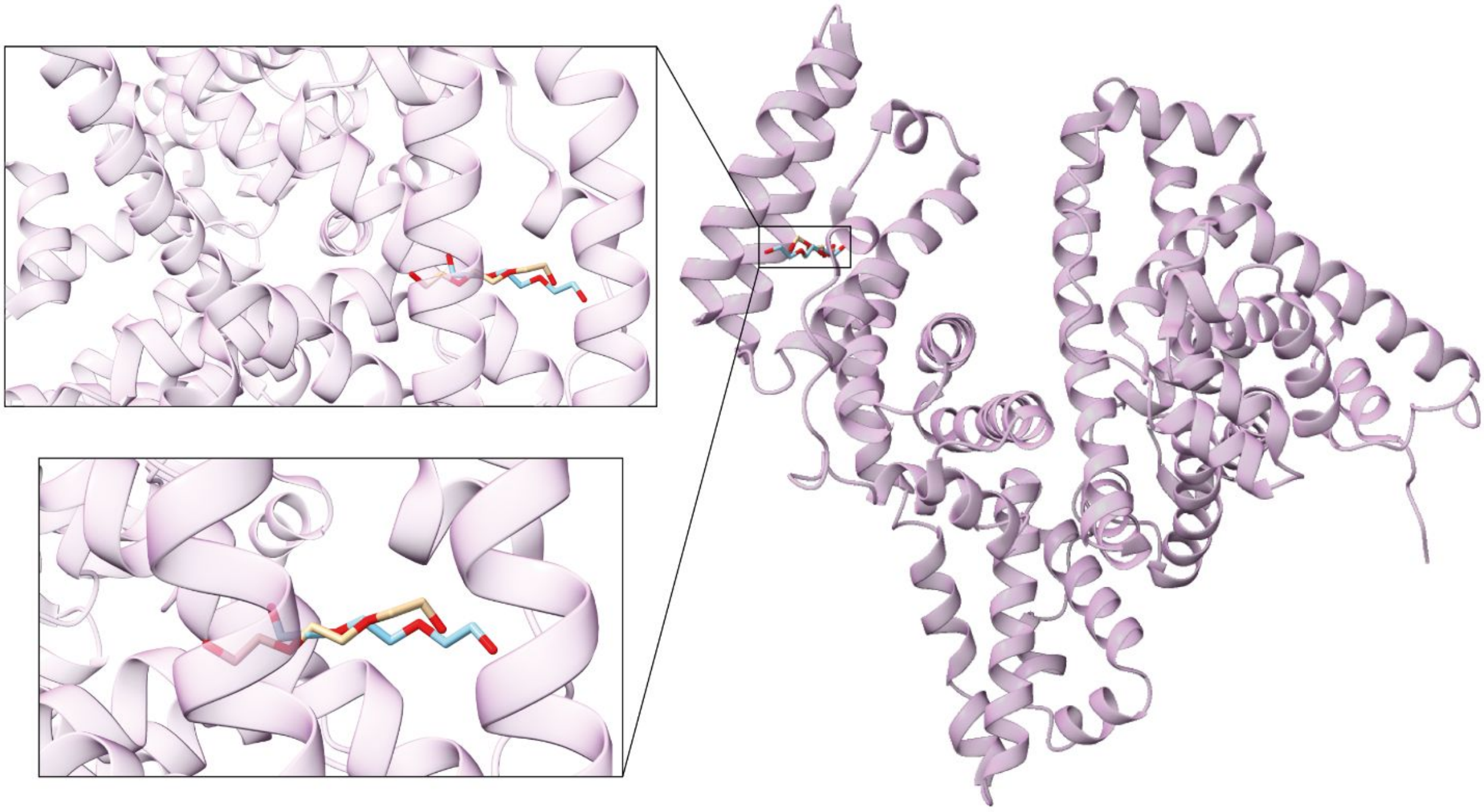

Figure S87. Superposition of the co-crystallized TEG molecule (yellow) and the redocked pose (blue) within BSA. Views are shown at increasing magnification: overall protein structure, binding region, and detailed view of the ligand arrangement.

Table S7. Predicted binding energies of complexes C1w, C3w, and C5w at the regions surrounding TRP134 and TRP213 in BSA.

**Complex**	**Predicted binding energy (kcal/mol)**	
	TRP134 region	TRP213 region
**C1w**	-8.34	-5.60
**C3w**	-5.68	-4.56
**C5w**	-5.73	-4.31

Table S8. Summary of interactions, binding distances to site III residues, and predicted binding energies of complexes **C1w**, **C3w**, and **C5w** with BSA.

Complex	Complex atom/group	Receptor group	Interaction type	Distance (Å)	Predicted binding energy (kcal/mol)
**C1w**	OH_2	GLU125	Hydrogen bond	1.83	-8.34
	$OMe_{anisole}$	ILE181		3.76	
	Ir1	GLU125	Electrostatic Attractive Charge	3.50	
	Biquem	LEU115	π-σ	3.76	
		LEU122		3.80	
	$Ph_{anisole}$	TYR160	π-π T-shaped	5.00	
		TYR137		5.06	
	Biquem	LEU115	π-alkyl	4.35	
		LYS136		4.72	
		PRO117		4.98	
		LEU122		5.24	
	$OMe_{anisole}$	TYR137		5.49	
	$Ph_{anisole}$	ILE181		5.41	
	$OMe_{anisole}$	MET184	Alkyl	3.21	
		ARG185		4.29	
		VAL188		4.77	
**C3w**	OH_2	GLU16	Hydrogen bond	1.84	-5.68
		GLU17		1.90	
	$O_{morpholine}$	GLU130		2.04	
	$OMe_{anisole}$	GLU130		3.40	
		GLN165		3.78	
	Ir1	GLU17	Electrostatic Attractive Charge	3.83	
		GLU16		4.35	
	Biquem	GLU17	π-anion	4.62	
		LYS131	π-cation	4.65	
	Biquem	LYS131	π-alkyl	4.57	
	$Ph_{anisole}$			5.14	
	$OMe_{anisole}$	PHE126		4.64	
**C5w**	$O_{morpholine}$	GLN165	Hydrogen bond	1.99	-5.73
	$CH_{2\ morpholine}$	GLU130		3.30	
	Ir1	GLU130	Electrostatic Attractive Charge	4.95	
		ASP129		5.42	
	Ir2	GLU130		4.74	
	Biquem	GLU130	π-anion	2.67	
		LYS131	π-alkyl	5.25	

Biquem = 4-(2-(3*H*-benzo[*h*]imidazo[4,5-*f*]quinolin-3-yl)ethyl)morpholine, GLU = glutamic acid, ILE = isoleucine, LEU = leucine, TYR = tyrosine, LYS = lysine, PRO = proline, MET = methionine, ARG = arginine, VAL = valine, GLN = glutamine, PHE = phenylalanine.

11. Biological studies

Table S9. Cytotoxicity of the Ir(III) complexes on A549 cancer cells in dark conditions and upon blue light irradiation.

Complex	IC_{50} (µM)		PI
	Dark	Blue light [a]	
**C1w**	19.8 ± 3.2	8.2 ± 3.7	2.4
**C2w**	28.1 ± 1.8	25.18 ± 0.24	1.1
**C3w**	13.0 ± 1.3	6.86 ± 0.56	1.9
**C4**	10.8 ± 3.2	18.0 ± 2.4	0.69
**C5w**	9.2 ± 1.8	16.51 ± 0.26	0.56
**Cisplatin**	5.9 ± 1.2	n.m.	n.m.

[a] Cells were incubated with the compounds at 37 °C for 48 hours in the dark (for dark experiments) or for 4 hours followed by 1 hour of blue light irradiation (460 nm, 24.1 J cm^{-2}) and an additional 43 hours of incubation (for light experiments). IC_{50} values were determined by MTT assays. Data represent the mean ± standard deviation of at least three independent experiments performed in triplicate. Phototoxic indexes were calculated as PI = $IC_{50,dark}$ / $IC_{50,light}$.
